# Genetic Structure of Capelin (*Mallotus villosus*) in the Northwest Atlantic Ocean

**DOI:** 10.1371/journal.pone.0122315

**Published:** 2015-03-30

**Authors:** Ellen L. Kenchington, Brian S. Nakashima, Christopher T. Taggart, Lorraine C. Hamilton

**Affiliations:** 1 Department of Fisheries and Oceans, Bedford Institute of Oceanography, Dartmouth, Nova Scotia, Canada; 2 Department of Fisheries and Oceans, Northwest Atlantic Fisheries Centre, St. John’s, Newfoundland and Labrador, Canada; 3 Oceanography Department, Dalhousie University, Halifax, Nova Scotia, Canada; The Ohio State University, UNITED STATES

## Abstract

Capelin (*Mallotus villosus*) is a commercially exploited, key forage-fish species found in the boreal waters of the North Pacific and North Atlantic Oceans. We examined the population structure of capelin throughout their range in the Canadian northwest Atlantic Ocean using genetic-based methods. Capelin collected at ten beach and five demersal spawning locations over the period 2002 through 2008 (*N* = 3,433 fish) were genotyped using six polymorphic microsatellite loci. Temporally distinct samples were identified at three beach spawning locations: Chance Cove, Little Lawn and Straitsview, Newfoundland. Four capelin stocks are assumed for fisheries management in the northwest Atlantic Ocean based on meristics, morphometrics, tag returns, and seasonal distribution patterns. Our results suggested groupings that were somewhat different than the assumed structure, and indicate at least seven genetically defined populations arising from two ancestral populations. The spatial mosaic of capelin from each of the two basal cluster groups explains much of the observed geographic variability amongst neighbouring samples. The genetic-defined populations were resolved at Jost’s *D*_est_ ≥ 0.01 and were composed of fish collected 1) in the Gulf of St. Lawrence, 2) along the south and east coasts of Newfoundland, 3) along coastal northern Newfoundland and southern Labrador, 4) along coastal northern Labrador, 5) near the Saguenay River, and at two nearshore demersal spawning sites, 6) one at Grebes Nest off Bellevue Beach on the east coast of Newfoundland, and 7) one off the coast of Labrador at Domino Run. Moreover, the offshore demersal spawners on the Scotian Shelf and Southeast Shoal appeared to be related to the inshore demersal spawners at Grebes Nest and in Domino Run and to beach spawners from the Gulf of St. Lawrence.

## Introduction

Capelin (*Mallotus villosus* Müller) is an energy-rich, small forage-fish for a suite of fish, seabird and marine mammal species throughout their circumpolar range, and their extensive feeding migrations serve to transfer energy from one location and time to another [1–5]. As a planktivorous species they are an essential part of the food web, with only Arctic cod (*Arctogadus glacialis*) and sandlance (*Ammodytes* sp.) fulfilling a similar role, though they are less abundant and not as widespread.

Capelin spawn on the bottom where their eggs adhere to the sediments and most capelin in the northwest Atlantic Ocean (NWA) spawn in the intertidal zone of gravel beaches, though some spawn on the sea bed at depths of 4 to 50 m; notably on the Southeast Shoal located on the Grand Bank [6] and in some coastal regions [7–9]. In contrast, capelin in the northeast Atlantic Ocean (NEA) are primarily demersal spawners [3]. The persistent use of spawning locations in the NWA, at least in the core of the documented species range, has led to hypotheses concerning spawning-site fidelity and thus a mechanism for population structuring. It is well known that prior to spawning, sex-specific schools of mature fish form near spawning locations [7] and that males are mostly semelparous, while females are often iteroparous and may spawn in two or more years [10, 11]. Males remain in the intertidal area of beaches during spawning and spawn multiple times before dying. When females are ready to spawn they arrive in the intertidal area where they are accompanied by one or two males. They release their eggs in one spawning effort, and then move off into deeper water [7].

Capelin in the NWA are considered to be a population complex representing one of four phylogenetic clades [12, 13] that ranges from coastal Labrador in the north, to the Scotian Shelf in the south, and into the Gulf of St. Lawrence to the west. Climate change influenced capelin distribution during the Pliocene and Pleistocene epochs when they underwent trans-Arctic migrations [12]. More recently (post-1990) the offshore capelin distribution in the NWA has shifted southward from Labrador to the northern Grand Banks [2, 14] and from the relatively shallow waters of the Grand Bank eastward to the shelf break and onto the Flemish Cap [15].

Circumpolar basin-scale analyses of microsatellite DNA data have shown that capelin collected in the Newfoundland and Labrador region are genetically distinct from those collected off west Greenland, in the NEA, the Barents Sea and in the eastern and western Pacific Ocean [16, 17]. The above genetic differentiation is consistent with similar studies based on mitochondrial DNA polymorphism [18–20]. While these different studies, using different DNA metrics, demonstrate large-scale genetic differentiation among capelin populations, no microsatellite DNA analyses have addressed the NWA stock complex despite evidence for sub-basin scale population structure inferred from meristics [21], morphometrics [22, 23], allozyme markers [23], seasonal patterns in distribution [24], and recurrent wide-spread spawning on specific beaches [25] and at specific nearshore [8, 9] and offshore [6] locations. Fine-scale microsatellite analyses of capelin in the NEA, documented by Præbel et al. [17] off northern Norway, showed no population differentiation though it is unclear how the sample populations were associated with spawning condition and in particular how they were associated with recurrently used spawning grounds. Furthermore, NWA capelin populations appear to be dominated by beach spawners while those in the NEA appear to be primarily offshore demersal spawners.

**Fig 1:**
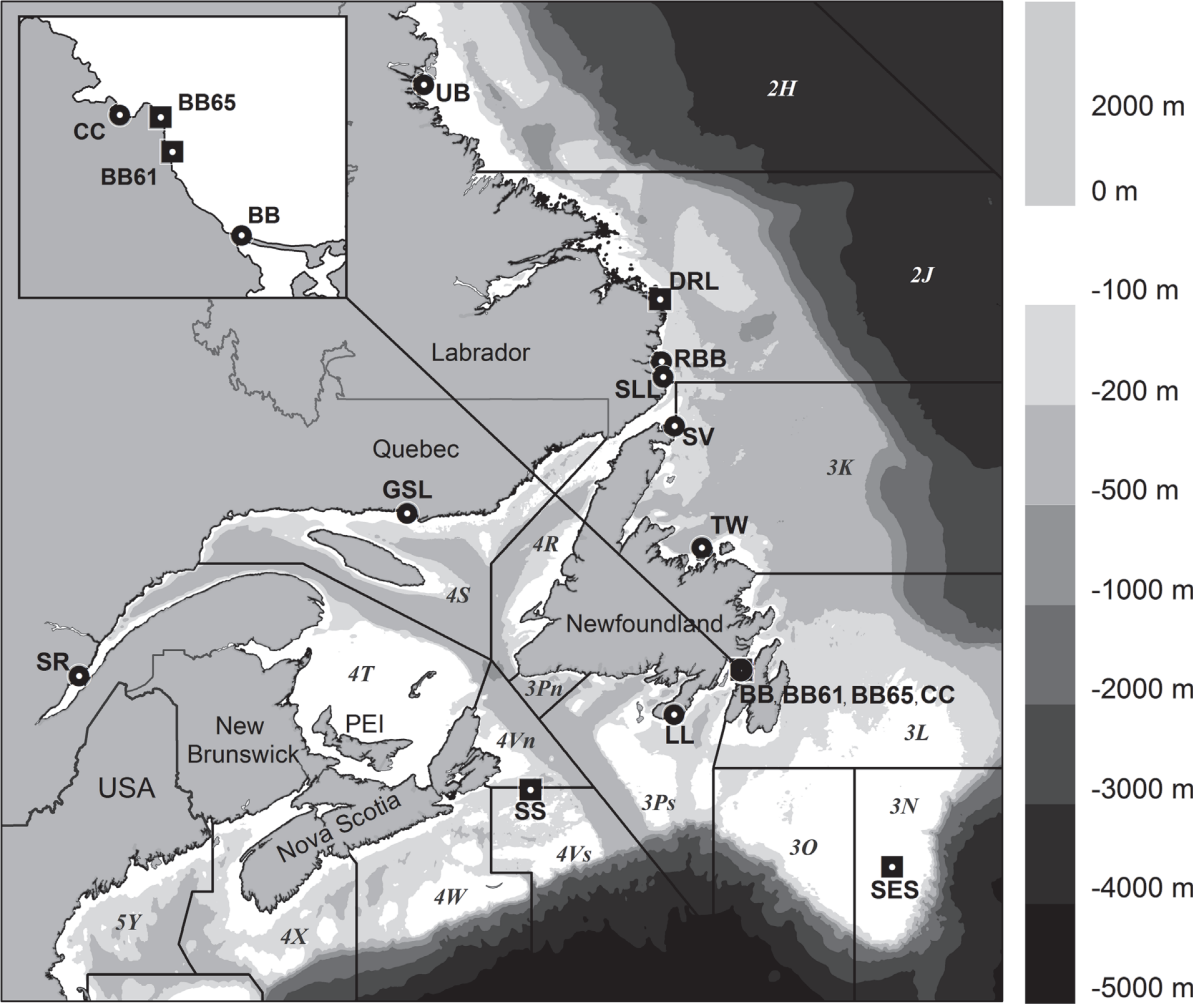
Bathymetric chart of the northwest Atlantic Ocean and adjacent Canadian-Atlantic provinces. Northwest Atlantic Fisheries Organisation (NAFO) statistical divisions (solid black line boundaries) and identifiers (italic font), and the 15 spawning capelin sampling locations at beach (solid circle) and demersal sites (solid square) along with their sample code identifiers (bold block font), as detailed in Table 1, are indicated.

Management of the capelin fisheries in the NWA assumes a four-stock complex [2] based, in part, on the various studies cited above. These stocks are represented by the Northwest Atlantic Fisheries Organization (NAFO) statistical Divisions 2J+3KL (east of Newfoundland and Labrador), 3NO (Southeast Shoal of the Grand Bank), 3Ps (south of Newfoundland), and 4RST (Gulf of St. Lawrence) (Fig 1). Given the importance of NWA capelin as a primary forage fish, and as a valuable commercial fishery, Carscadden and Vilhjalmsson [3] recommended that genetic evidence for population structure in the NWA be examined. Here we address population structure of capelin throughout the NWA using microsatellite DNA loci to evaluate the genetic evidence for the assumed contemporary stock structure. We identify genetic structuring that suggests population groupings somewhat different than the assumed stock structure.

**Table 1:** Details of Samples.

Sample descriptors		Geographic coordinates			Collection years	Sex		Total length (mm)			
Code	Location	Latitude	Longitude	N		N ♂	N ♀	Mean	S.E.	Min	Max
BB	Bellevue Beach	47.637085	-53.777626	654	2002–2008	355	299	157.43	0.49	121	187
BB61^1^	Gull Rock	47.663610	-53.806111	128	2004, 2006	94	34	158.69	1.06	128	184
BB65^1^	Grebes Nest	47.672990	-53.802196	98	2005	49	49	161.51	1.23	130	185
CC	Chance Cove	47.668330	-53.816667	530	2002–2008	265	265	160.44	0.58	122	191
DRL^1^	Domino Run	53.493333	-55.813333	88	2006	42	46	159.28	0.96	135	178
GSL	Aguanus River	50.216600	-62.083300	122	2006–2008	78	44	158.64	1.19	125	183
LL	Little Lawn	46.918330	-55.468890	392	2003, 2004, 2006–2008	194	198	160.27	0.68	120	191
RBB	Red Bay Beach	52.566670	-55.777780	186	2004, 2007	95	91	159.00	0.82	127	181
SES^1^	Southeast Shoal	44.273333	-50.068333	165	2006, 2007	80	85	155.44	0.97	127	183
SLL	St. Lewis	52.330000	-55.736666	98	2006	50	48	161.72	1.16	128	189
SR	Saguenay River	47.565000	-70.204666	62	2007	47	15	161.48	1.31	137	187
SS^1^	Scotian Shelf	45.627354	-59.025908	109	2006, 2007	64	45	135.29	1.86	99	170
SV	Straitsview	51.583889	-55.452500	299	2003–2006	134	165	155.72	0.66	124	178
TW	Twillingate	49.668056	-54.783330	272	2002, 2006–2008	129	143	158.64	0.75	120	186
UB	Unity Bay	56.543611	-61.666660	230	2004, 2005, 2007	131	99	159.38	1.14	108	190
Totals				3,433		1,807	1,626				

Capelin sample identifier code, location name and geographic coordinates (decimal degrees) and summary information for each sample including number of fish collected (*N*), minimum and maximum collection year of collection, number of males and females in each collection and the average, standard error (S.E.), minimum and maximum of total fish-length among the 15 sampling locations used in this study.

^1^Demersal spawning location.

## Materials and Methods

### Sampling

#### Ethics statement

All capelin samples for this field study were collected by the Canadian Department of Fisheries and Oceans, the regulatory authority for this species. Capelin are not a protected species and no specific permissions were required. Whole fish were sacrificed by exposing them to freezing temperatures (-20°C or -70°C) or killing them with a scalpel or sharp knife; most fish had a short life expectancy at the time of sampling due to mortality associated with spawning.

#### Field and tissue collections

A total of 3,433 individual capelin tissue samples were collected across several well-known beach and demersal spawning locations in the NWA at or near spawning time (Fig 1, Table 1) over the period 2002 through 2008. Collection dates ranged from 13 May to 25 August. Tissue samples were collected from capelin actively spawning on beaches at Little Lawn (LL), Bellevue Beach (BB), Chance Cove (CC), Twillingate (TW) and Straitsview (SV) on the island of Newfoundland; near the mouth of the Saguenay (SR) River in the St. Lawrence River estuary; at the Aguanas River (GSL) in the northern Gulf of St. Lawrence; and at Red Bay Beach (RBB), St. Lewis (SLL), and Unity Bay (UB) along the coast of Labrador. Samples were also collected from capelin aggregations at two nearshore demersal spawning sites at Gull Rock (BB61) and Grebes Nest (BB65) located at depths of 8 to 14 m and adjacent to Bellevue Beach [8]. One sample was collected at a demersal (20 m depth) spawning site in Domino Run, Labrador (DRL) in 2006. Tissues were also collected in 2006 and 2007 from post-spawning fish captured near demersal spawning sites on the Southeast Shoal (SES) and from immature fish on the Scotian Shelf (SS). With the exception of the immature capelin collected on the Scotian Shelf, all tissues were from fully mature and about to spawn, actively spawning, or partially spent males and females. This sampling scheme covered all NWA stock management areas and introduced a temporal sampling scheme not previously employed for capelin.

In most cases, individuals were collected using castnets and dipnets on beaches when the fish were actively spawning. In two exceptions, mature pre-spawning fish were collected using a capelin trap near Straitsview in 2006 and a tuck seine off Twillingate in 2008; each adjacent to a spawning beach. Samples collected from demersal spawning sites relied on a midwater trawl on the Southeast Shoal, a bottom trawl at Domino Run, and a modified crab-pot with a castnet-type closure that was lowered onto spawning aggregations at the Gull Rock and Grebes Nest locations. Immature capelin from the Scotian Shelf were collected during the annual Canadian Department of Fisheries and Oceans summer bottom-trawl survey.

Muscle tissue located below the dorsal fin was removed at the time of collection and preserved in 95% ethanol, or entire individuals were frozen and similar tissue was later excised and preserved as above. Each fish was measured for total length (± 1 mm), sex identified, and otoliths were collected for later age determination (± 1 year).

### DNA extraction, amplification and visualization

For DNA extraction, ~25 mg of the ethanol-preserved tissue was re-hydrated in one mL of DNase free water for 30 to 60 minutes. The water was removed and the DNA was extracted using a DNeasy 96 Blood and Tissue kit (Qiagen, Mississauga, Canada) following manufacturer instructions, without the optional RNase treatment. Purified DNA was collected from two elutions of 100 μL from the column and stored separately. The eluted DNA was quantified using the QuantIT PicoGreen assay (Invitrogen, Burlington, Canada) according to manufacturer instructions and fluorescence was measured using a FLUOStar OPTIMA (BMG Labtech, Offenburg, Germany) fluorescent-plate reader. All samples were normalized to ten ng/μL using ten mM Tris pH 8.0 prior to PCR (polymerase chain reaction) amplification.

Each DNA sample was amplified at six microsatellite loci: *Mvi*2, *Mvi*3, *Mvi*5, *Mvi*9, *Mvi*10 and *Mvi*16 selected from the twelve isolated by Gordos et al. [26]. The remaining six loci (see below) were not used in our analyses for the following reasons: 1) the allele range for each of *Mvi*1 and *Mvi*31 could not be resolved on the platform we used; 2) preliminary analyses of 149 samples collected in 2002 indicated that the *Mvi*14 and *Mvi*33 loci did not amplify reliably resulting in too many missing data; and 3) the *Mvi*12 and *Mvi*22 loci showed extreme deviations from Hardy-Weinberg equilibrium (HWE). Of the six loci we used, only the *Mvi*5 and *Mvi*10 loci showed allele sizes that followed a predominantly tetranucleotide pattern; the *Mvi*3 locus had alleles at two- and four-bp (base-pair) intervals, and the *Mvi*2, *Mvi*9 and *Mvi*16 loci contained interrupted repeats with alleles at one-, two- and four-bp intervals.

The PCR reactions were performed in a ten μL volume containing approximately 25 ng DNA, 1X KCl PCR buffer (MBI Fermentas, Burlington Canada) (10mM Tris pH 8.8, 50 mM KCl and 0.08% (v/v) Nonidet P40), 1.5 mM MgCl_2_, 0.2 mM dNTP; 0.25 μM each primer and 0.5U Taq (MBI Fermentas). The PCR cycles consisted of three minutes at 94°C for one cycle; three cycles of one minute each at 94°C, annealing temperature (AT) for 30 seconds, 45 seconds at 72°C; then X cycles of 30 seconds at 94°C, AT for 30 seconds and 45 seconds at 72°C; followed by one cycle of ten minutes at 72°C and a holding temperature of 10°C. Annealing temperatures (AT) and cycles (X) for each locus respectively were: *Mvi*2 53°C, 28 cycles; *Mvi*3 54°C, 28 cycles; *Mvi*5 56°C, 28 cycles; *Mvi*9 62°C, 30 cycles; *Mvi*10 55°C, 30 cycles and *Mvi*16 62°C, 35 cycles.

PCR products were size-separated on a 6% Long Ranger acrylamide (Lonza, Walkersville, MD USA) gel containing seven M urea (Sigma Aldrich, Oakville, Canada) using 1X TBE buffer (Sigma Aldrich) (89 mM Tris-borate, 2mM EDTA pH 8.3). The fragments were detected using a FMBIOII or FMBIOIII (Hitachi Software Engineering America, Ltd., Miraibio Division, San Francisco, CA USA) as used in Gordos et al. [26] for these loci. For FMBIOII detection all loci were labelled in HEX and two μL of the PCR product was combined with eight μL loading dye (6.6 mM EDTA, ten mg bromophenol blue; Sigma Aldrich, in deionized formamide; Sigma Aldrich). For FMBIOIII detection the *Mvi*9, *Mvi*10 and *Mvi*16 loci were labelled in HEX and the *Mvi*2, *Mvi*3 and *Mvi*5 loci were labelled in FAM, and the loci were combined based on similar allele ranges [26] to allow two loci to be run on each gel. One μL of each PCR product was combined with eight μL loading dye (6.6 mM EDTA, ten mg Bromophenol blue in deionized formamide) as follows: *Mvi*9 and *Mvi*2; *Mvi*5 and *Mvi*10 and *Mvi*3 and *Mvi*16. For either detection platform two μL of the sample in loading dye was run on a gel. Relative allele sizes were manually assigned (scored) to each allele against a ladder of known-size fragments and control samples were run on each gel (inter- and intra-gel controls). Further details of the quality control steps that we applied to our data are presented as Supplementary Material (S1 Text).

### Analytical Approach

Complete genotypes across all six microsatellite loci were obtained from 3,433 individuals among 45 collections and 15 locations (Table 1, Fig 1) represented by 1,807 (53%) male and 1,626 (47%) female capelin. As no sex effect was detected in preliminary analyses, the males and females were not separately analyzed. In our analyses, permutation and exact tests were used to estimate the probability value of departure from a given null hypothesis. When interpreting our results we evaluated the probabilities (*P*) using *α* = 0.05 with subsequent Bonferroni corrections for the number of tests [27]. We additionally used the upper 95% confidence interval around the statistic to identify tests that have large differences relative to the sample distribution [28–30] in order to assess biological relevance. Bootstrapping and jack-knifing provided an estimate of the confidence interval around the observed value.

#### Genetic Analyses

We conducted preliminary analyses on the 45 samples to assess linkage disequilibrium (LD) and to determine whether temporal samples from each location could be pooled. Details of those analyses are presented as Supplementary Material (S2 Text, S1 Table). Following the pooling of similar within-location collections sampled in different years, the new set of 18 samples from the 15 locations were re-evaluated (S2 Table) following the methods described in the Supplementary Material (S2 Text). Genepop version 4.0.10 [31] was used to calculate exact tests of departure from HWE [32]. Comparative measures of genetic diversity for each sample were calculated for each locus and where applicable, over all loci: allelic richness (*N*_A_), the number of alleles (*R*_S_), the number of private alleles (*P*_S_) for a standard sample size of 38 individuals, observed (*H*_O_) and expected number of heterozygotes (*H*_E_), and Weir and Cockerham’s [33] inbreeding coefficient (*F*_IS_) using Genetix version 4.05.2 [34] and Adze version 1.0 [35]. The number of alleles for a standard sample size was calculated using a rarefaction approach using Adze; allele frequencies for each locus were produced with Genetix. Weir and Cockerham’s [33] *F*-statistics (θ) were also re-calculated to examine pair-wise genetic differentiation and to test the null hypothesis of panmixia. Samples outside the upper 95% confidence interval for θ were considered to be highly differentiated.

The Præbel et al. study of genetic variation among NEA capelin [17] used assignment tests performed with Geneclass version 2.0 [36] to evaluate the fidelity of individuals to their group of origin. We undertook the same Bayesian individual assignment analyses, following Rannala and Mountain [37]; the details of which are provided as Supplementary Material (S3 Text).

Fixation indices systematically underestimate genetic differentiation when evaluated with highly polymorphic markers [38]. We used the harmonic mean of Jost's *D*_est_ [39] as a measure of heterozygosity-based relative differentiation of allele frequencies among samples (actual differentiation) adjusted for sample size. The *D*_est_ parameter is based on allele identities and estimates allelic differentiation by partitioning heterozygosity within and among population components. This improves the estimates of population genetic divergence by not confounding within-group heterozygosity with divergence [39]. *D*_est_ is not rooted in *F*-statistic-based metrics and performs consistently under different modelled levels of haplotype diversity and genetic distance between populations [40]. It is also considered preferable for estimating genetic differentiation from high diversity loci (such as those used here) relative to *F*_ST_ or *G*_ST_ that may underestimate genetic divergence [39, 41, 42]. Both *D*_est_ and *F*-statistics weight the common alleles more heavily than the rare alleles [39, 40, 43]. *D*_est_ was calculated with the online program Smogd version 1.2.5 (http://www.ngcrawford.com/django/jost) [44] separately for each locus and as the harmonic mean across loci. Bootstrapped 95% confidence intervals were calculated through resampling of individuals over 1,000 iterations. To test for the effect of the rare alleles in our data on *D*_est_ we used the program SPADE (updated 2009) [45] which allows for allele frequency input and hence manipulation. We estimated *D*_est_ for each locus after removing all alleles with an average frequency across samples of less than 0.05. For *Mvi*9 and *Mvi*16, no average allele frequencies met this criterion and so 0.01 was used as the threshold. These levels have been used to define rare allele thresholds for examining the effect of rare alleles on estimates of effective population size [46].

Jost’s *D*_est_ pair-wise distance matrix was imported into Primer version 6.1.5 (PRIMER-E Ltd, Plymouth, UK). Samples within this matrix were visualized on a non-metric (no units) multidimensional scaling plot (nMDS) based on Euclidean distances, such that the ranked differences among samples were preserved. The degree of correspondence between the distances among points implied by the nMDS scatterplot and the Jost *D*_est_ matrix input was evaluated using the Kruskal stress formula 1 implemented in Primer. A complete linkage hierarchical cluster analysis was performed on this matrix. In complete linkage hierarchical clustering, the two clusters with the smallest pair-wise distance are merged in each step. This method was chosen as it is sensitive to outliers.

The nMDS ordination considers the genetic distance between all possible pairs of samples and highlights those that are most differentiated from the others in Euclidean space. The expression of population differentiation in geographic space and the location of “barriers”, or areas of reduced gene flow among neighbouring samples, were also assessed using Barrier version 2.2 [47]. Geographical latitude and longitude co-ordinates, by sample, were used along with the genetic data (Jost’s *D*_est_ excluding temporally differentiated samples from the same location) to generate a connectivity network of genetic distances based on Delaunay triangulation and Voronoï tessellation. Automatically generated virtual points prevented long edges forming in the Delaunay triangles. Monmonier’s Maximum Difference algorithm [48] was then used to identify putative genetic barriers across the geographic landscape. In this analysis the user specifies the numbers of barriers; the first being positioned along the axis of greatest genetic differentiation, with subsequent barriers decreasing in order of importance and representing diminishing levels of differentiation. We computed barriers using a genetic matrix based on the harmonic mean of *D*_est_ to represent genetic differentiation among capelin from the 15 locations. We ran the analyses excluding the three temporally distinct samples (2005CC, 2005SV, 2004LL) and by replacing CC, SV and LL samples with the temporally distinct samples to assess the stability of the results. Barrier robustness was evaluated by examining “consensus barriers”, that is the number of loci supporting the individual sections of the barriers constructed from single locus matrices of *D*_est_ analyzed as above in the multiple matrix mode of Barrier. We sequentially considered both the consensus support for each barrier and the genetic distance between sample pairs at those barriers. We concluded our assessment (rejection of additional barriers) when a barrier was supported by fewer than four of the six loci (67%) in any segment over its length and (or) when average *D*_est_ over all segments forming the barrier between adjacent samples connected through the oceanic features, was less than 0.01.

Isolation by distance (IbD) describes the tendency of individuals to mate with others that are nearby rather than with individuals from some distant population and if present can cause genetic diversity to be clinal over the sample area. Mantel tests [49] were used to test for congruence between genetic (Jost’s *D*_est_) and geographic (shortest sea route; km) distances among the 15 locations (excluding temporally differentiated samples) with the web-service program Isolation By Distance (Ibdws) [50]. Ibdws was used to calculate the slope and intercept of the IbD relation using reduced major axis regression [51].

We further investigated the genetic population structure of capelin using the Bayesian clustering program in Structure (version 2.3.4) [52]; the details of which can be found as Supplementary Material (S4 Text).

## Results

Tissue samples used for microsatellite DNA loci assessment were collected from fish ranging in age from one to six years (44% age-two, 47% age-three) representing the 1996 through 2006 year-classes, with the majority (99%) in the 1999 through 2006 year-class range. The total length of individuals ranged between 99 and 191 mm and averaged 158.03 mm (0.24 standard error; S.E.). There were marginal differences in average and median lengths among the 15 site locations with the exception of the Scotian Shelf (SS) samples that consisted of immature fish of age-one and-two that averaged 135.29 mm and had the greatest variation (1.86 S.E.) in total length (Table 1). Although eleven year-classes were represented, the majority of sampling sites had collections associated with the 2001 through 2005 year-classes and the 2004 year-class was represented at all sampling sites with the exception of Grebes Nest (BB65) near Bellevue Beach (BB). The median age ranged from two to three years for the 15 locations with age-two and-three fish predominating except for the Scotian Shelf (SS) where immature age-one and-two fish were collected.

The microsatellite loci varied widely in the observed number of alleles and illustrated the high level of variability typically associated with marine fish species. The *Mvi*2, *Mvi*9 and *Mvi*16 loci had 137, 153 and 158 alleles respectively, while the *Mvi*3, *Mvi*5 and *Mvi*10 loci were less variable with 36, 25 and 42 alleles respectively. This complexity in allelic variation reflects the one, two and four base pair (bp) variation associated with loci *Mvi*2, *Mvi*9, *Mvi*16 and to a lesser degree *Mvi*3 and our large sample sizes which enabled the detection of rare alleles. In all of these loci the most common alleles differed in size by four bp consistent with the gain or loss of one or more repeat unit and varied amongst samples. However one and two bp variations were spread throughout the allelic size range, suggestive of interrupted repeats in these loci.

Our preliminary genetic analyses (S2 Text) indicated that collections 2005CC, 2004LL, and 2005SV were genetically different from other temporal collections from their respective locations (i.e., CC, LL and SV) and most subsequent analyses were performed on 18 samples (15 locations + 2005CC, 2004LL, 2005SV) (S2 Table).

Allelic richness per locus, by standard sample (*R*_S_), was similar among samples, with no sample particularly allelic-rich or-poor (S3 Table). The allelic richness and the number of private alleles in a standard sample (*P*_S_) were similar among samples within loci and over all loci. Observed heterozygosity was high and ranged from 0.880 in the Aguanus River (GSL) sample to 0.927 in the St. Lewis, Labrador (SLL) sample. Re-evaluation of the HWE proportions in the 18 samples identified three of the 108 combinations of samples and loci to be significant, after correcting for multiple tests (21 were significant prior to correction). All three were associated with a large *F*_IS_ (2005 SV-*Mvi*9; BB and GSL- *Mvi*16) outside the 95% C.I. for the statistic (*F*_IS_ estimated at 0.0039; 95% C.I. 0.0184–0.0626).

Genetic differentiation among the 18 samples based on θ was small (mean ± standard deviation; S.D.: 0.0007 ± 0.0004; 95% C.I. 0.0002–0.0014) and ranged from < 0 to 0.0045 (Table 2). Samples showing a significant departure from HWE proportions in the above analyses (2005 SV-*Mvi*9; BB and GSL-*Mvi*16) did not exhibit a significant effect on θ as determined by jack-knifing across samples at each locus (all values within 95% C.I. of θ). Therefore, we did not consider it necessary to exclude them from subsequent analyses. Locus *Mvi*9 had the most influence on the value of multilocus θ (S4 Table).

**Table 2:** Genetic Differentiation.

	BB	BB61	BB65	CC	2005CC	DRL	GSL	LL	2004LL	RBB	SES	SLL	SR	SS	SV	2005SV	TW	UB
**BB**		0.0015	0.0139	0.0012	0.0234	0.0110	0.0078	0.0026	0.0149	0.0028	0.0065	0.0044	0.0261	0.0095	0.0090	0.0311	0.0005	0.0058
**BB61**	-0.0000		0.0152	0.0007	0.0434	0.0314	0.0069	0.0000	0.0113	0.0001	0.0139	0.0121	0.0275	0.0202	0.0066	0.0222	0.0023	0.0060
**BB65**	0.0012	0.0012		0.0118	0.0409	0.0103	0.0111	0.0119	0.0283	0.0128	0.0226	-0.0023	0.0115	0.0084	0.0160	0.0487	0.0152	0.0144
**CC**	0.0001	0.0000	0.0011		0.0377	0.0151	0.0067	0.0034	0.0080	0.0002	0.0078	0.0005	0.0246	0.0121	0.0022	0.0271	0.0063	0.0058
**2005CC**	**0.0022**	**0.0036**	**0.0021**	**0.0027**		0.0380	0.0173	0.0253	0.0413	0.0225	0.0328	0.0306	0.0531	0.0179	0.0241	0.0240	0.0317	0.0321
**DRL**	0.0014	**0.0022**	0.0008	**0.0016**	**0.0019**		0.0147	0.0209	0.0393	0.0139	0.0147	0.0003	0.0404	0.0013	0.0201	0.0150	0.0104	0.0134
**GSL**	0.0004	0.0003	0.0007	0.0002	0.0009	0.0007		0.0018	0.0277	0.0104	-0.0002	-0.0019	0.0148	0.0019	0.0125	0.0074	0.0043	0.0120
**LL**	0.0002	-0.0006	0.0012	0.0002	**0.0021**	**0.0020**	-0.0001		0.0171	0.0019	0.0035	0.0021	0.0084	0.0113	0.0105	0.0246	0.0007	0.0052
**2004LL**	**0.0015**	**0.0019**	**0.0031**	0.0012	**0.0043**	**0.0045**	**0.0027**	**0.0020**		0.0034	0.0227	0.0200	0.0332	0.0204	0.0181	0.0439	0.0120	0.0190
**RBB**	0.0003	-0.0002	0.0011	-0.0002	**0.0020**	**0.0017**	0.0002	-0.0001	0.0003		0.0091	0.0078	0.0377	0.0047	0.0000	0.0234	0.0022	0.0051
**SES**	0.0006	0.0010	0.0012	0.0006	**0.0018**	0.0009	-0.0011	0.0002	**0.0031**	0.0006		-0.0022	0.0147	0.0087	0.0122	0.0164	0.0032	0.0091
**SLL**	0.0001	0.0006	-0.0006	-0.0002	**0.0023**	-0.0002	-0.0012	0.0002	**0.0022**	0.0005	-0.0007		0.0149	0.0022	0.0117	0.0242	0.0017	0.0037
**SR**	**0.0015**	0.0012	0.0007	0.0013	**0.0030**	**0.0020**	0.0004	0.0007	**0.0029**	**0.0020**	0.0006	0.0006		0.0234	0.0275	0.0505	0.0209	0.0435
**SS**	0.0013	**0.0016**	0.0014	0.0013	0.0009	0.0006	0.0004	0.0007	**0.0025**	0.0004	0.0006	0.0007	**0.0019**		0.0169	0.0144	0.0085	0.0075
**SV**	0.0005	0.0005	0.0014	0.0002	**0.0021**	**0.0019**	0.0008	0.0008	0.0013	-0.0003	0.0009	0.0008	**0.0015**	**0.0015**		0.0391	0.0089	0.0091
**2005SV**	**0.0026**	**0.0018**	**0.0037**	**0.0025**	**0.0021**	0.0005	0.0003	**0.0019**	**0.0044**	**0.0025**	**0.0017**	**0.0016**	**0.0033**	0.0009	**0.0033**		0.0323	0.0429
**TW**	-0.0001	0.0002	0.0014	0.0004	**0.0025**	0.0012	0.0003	0.0002	**0.0017**	0.0004	0.0004	0.0000	0.0010	0.0013	0.0007	**0.0023**		0.0047
**UB**	0.0003	0.0003	0.0014	0.0004	**0.0025**	0.0014	0.0004	0.0004	**0.0018**	0.0003	0.0008	0.0004	**0.0021**	0.0012	0.0004	**0.0030**	0.0003	

Weir and Cockerham’s *F*-statistics (θ) for sample pairs (below diagonal) and the harmonic mean across loci of Jost’s *D*_est_ of actual differentiation (above diagonal). Sample codes are as in Table 1. BB61, BB65, DRL, SES and SS are demersal spawning locations; all others are beach spawning locations. Values outside the upper 95% confidence interval of θ are indicated in bold-type. Values of θ not significant at *α* = 0.05 in exact tests are underlined.

Exact test of the 153 sample pairs found 124 significant at *P* ≤ 0.05 and 91 significant after correcting for multiple tests. In producing those results the MC appeared to mix well with small standard error and the number of switches always greater than 10,000. Fifty-three of the 153 pair-wise combinations of samples produced estimates of multilocus θ greater than the upper limit of the 95% C.I. indicating that some sample pairs were very highly differentiated (Table 2). Forty of those results included in the tested sample pair one of the three single-year samples that were significantly differentiated in the analysis of temporal variation (2005CC, 2004LL, 2005SV). Of the remaining 13 sample pairs, nine included one or more of the five demersal spawning sites as one of the tested pairs (Table 2), indicating that at least some of the genetic structure in these data may be attributable to spawning behaviour. Also of note is the significant difference between the Saguenay River (SR) sample and the others in nine of 17 pair-wise comparisons (Table 2).

Comparison of θ with Jost’s *D*_est_ (Table 2 and S4 Table) showed the latter to be higher and more variable among loci, as expected. The harmonic mean of *D*_est_ was 0.012 in contrast to a multilocus θ of 0.0006. The loci *Mvi*2, *Mvi*9 and *Mvi*16 produced greater estimates of differentiation among samples in *D*_est_ than the other loci and ranged from 0.026 to 0.197 with Mvi9 having the largest values of *D*_est_ (S4 Table). These loci also had the largest number of alleles and largest apparent difference in allelic frequencies among samples. *D*_est_ through its formulation gives greater weight to the common alleles and the effect of low frequency (rare and diverse) alleles on the value of *D*_est_ in our data was minimal (S5 Table) demonstrating that these data can be used to determine genetic differentiation between populations.

**Fig 2:**
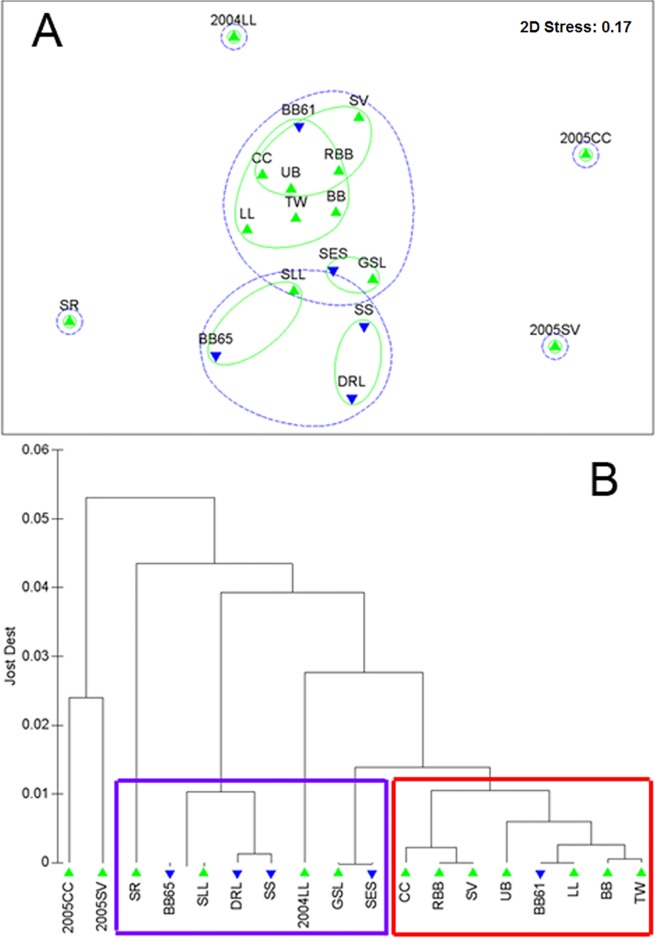
Visual relationships amongst samples based on Jost’s *D*_est_ measure of genetic differentiation. A. Non-metric multidimensional scaling (nMDS) plot of the 18 capelin samples using Jost’s *D*_est_ measure of genetic differentiation. Circles encompassing samples represent the 0.01 (green) and 0.02 (blue dash) Jost’s *D*_est_ distance from the cluster analysis. B. The complete linkage hierarchical cluster analysis of the same data. Basal genetic populations identified through Bayesian clustering with the program Structure under the optimal number of populations (*K* = 2) are indicated on the dendrogram: samples with highest proportional membership to Cluster I (blue box); samples with highest proportional membership to Cluster II (red box). Note that the temporal sample 2004LL was not included in the Structure analyses. In both plots (A and B), samples from demersal and beach spawning locations are distinguished by symbols (blue inverted triangle: demersal spawning site; green triangle: beach spawning site).

The relationship among sample sites based on pair-wise *D*_est_, as visualized through nMDS, produced a stress level of 0.17 in the two-dimensional presentation (Fig 2A). The samples did not reflect any strong geographic organization. In both the complete linkage cluster analysis and the nMDS, the three temporal samples were very different from the others as observed in the analyses of temporal stability using multilocus θ. Their level of differentiation was greater than most other pair-wise combinations (Table 2) and the data for these samples revealed distinctive allele frequency distributions. The sample from the Saguenay River (SR) was also separated from the other samples in the nMDS and showed a large distance from the other samples in the cluster analysis (Fig 2B).

**Fig 3:**
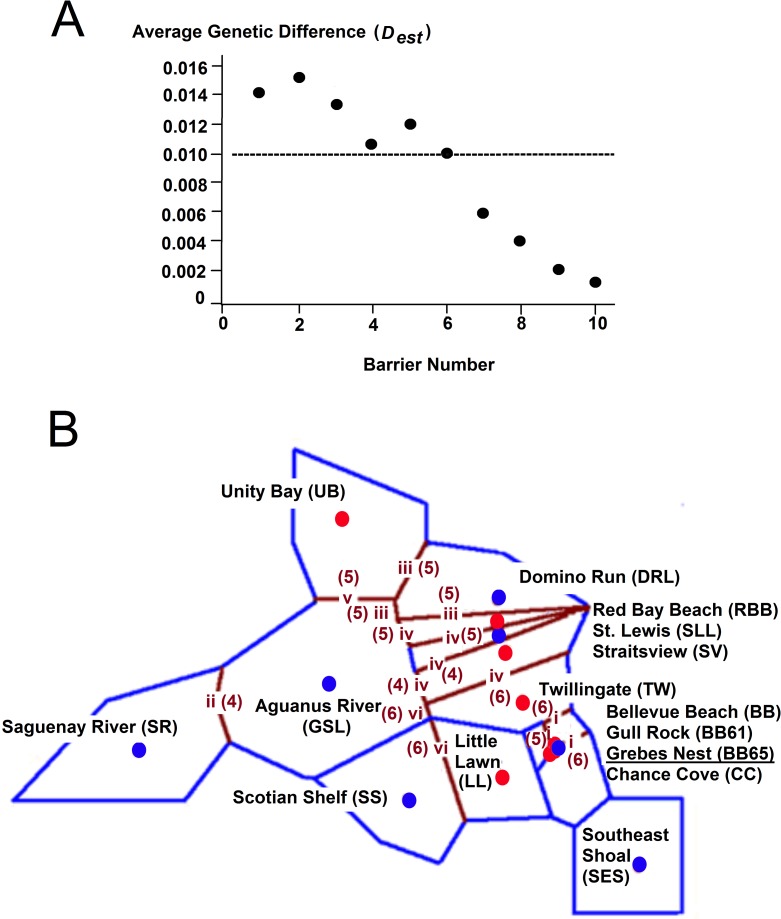
Spatial Genetic Structure. A. The average genetic difference (Jost’s *D*_est_; Table 2) between the samples identified by each of 10 successive barriers. B. Genetic “barriers” to gene flow (numbered brown lines) among capelin calculated using the Monmonier algorithm from the overall Jost’s *D*_est_ matrix (Table 2) based on samples from 15 locations. Blue lines illustrate the Voronoï tessellation. Filled circles represent the geographic position of samples identified through Bayesian clustering with Structure as belonging to Cluster I (blue) or Cluster II (red). Barriers are numbered sequentially in order of strength using Roman numerals. Six barriers are shown. Bracketed numbers represent the number of loci supporting the barrier (maximum 6). Barrier i surrounds the BB65 location.

The geographic expression of genetic variation as measured by *D*_est_ and the location of areas of higher differentiation among neighbouring samples (referred to hereafter as barriers) among the 15 sample locations are illustrated in Fig 3. The number of loci used to support the barriers through sub-replicate tests can be used to make decisions on how many barriers to consider. However, in our data this was not a useful tool as the first ten barriers (the limit of our analysis) were supported by four or more loci. Consequently we used the average genetic distance (Table 2, Fig 3A) between adjacent samples connected through the oceanic features to evaluate the number of barriers to interpret—retaining those with an average *D*_est_ across segments ≥ 0.01 as average genetic distance dropped to 0.006 in the next iterations for barriers 6 to 10 (Fig 3A).

Six barriers were evaluated using our *a priori* decision tree, all supported by four or more of the six loci. Monmonier’s Maximum Difference algorithm, in its first iteration (barrier-i), identified the area with the highest genetic differentiation to occur between capelin collected at the demersal spawning site Grebes Nest (BB65) and those from the two nearby spawning beaches at Bellevue Beach (BB) and Chance Cove (CC) and the demersal spawning site at Gull Rock (BB61) (Fig 3B). This barrier was well supported by six, five, and six loci in each of its three borders. Barrier-ii isolated the Saguenay River (SR) capelin in the St. Lawrence River estuary from all other samples. This barrier was also well supported by four of the six loci. Barrier-iii isolated the demersal spawning location in Domino Run, Labrador (DRL) from neighbouring samples (UB, GSL, and RBB) with support from five loci on each border. These first three barriers isolated the three samples that were separated from the others on the nMDS visualization of genetic differentiation (Fig 2A). Barrier-iv identified a distinct group of beach spawners collected at Straitsview (SV) and Red Bay Beach (RBB) with support of five, five, four, four, and six loci, while the fifth barrier separated the Unity Bay (UB) sample from the others with support of five loci. The sixth barrier separated the St. Lewis (SLL), Aguanus River (GSL) and Scotian Shelf (SS) capelin from those collected on spawning sites at Twillingate (TW), Little Lawn (LL), Bellevue Beach (BB), Gull Rock (BB61), Chance Cove (CC), and the Southeast Shoal (SES) supported by six loci. These samples showed similar groupings in the nMDS visualization (Fig 2A). Replacing the CC, LL and SV samples with the temporally distinct samples from those locations (2005CC, 2004LL and 2005SV) produced barriers around those samples in the first three iterations as expected (Table 2), and thereafter proceeded to reproduce the same major groupingsabove with the exception of TW which remained with the GSL, SS and SLL group after ten barriers (after which the average genetic distance was < 0.01).

There was a significant correlation between geographic distance and Jost’s *D*_est_ measure of genetic distance (*Z* = 844.6565, *r* = 0.4242, *P* = 0.0202). However, this correlation was driven by a single datum (Saguenay River/Unity Bay pair). Removing that datum and removing the Saguenay River sample both produced insignificant results.

Bayesian clustering inferred from the Structure program recovered mean values of *Ln P(D)* through different *K* values from 1–15 (S4 Text, S6 Table) under running conditions indicative of good mixing of the MCMC. Use of sampling location in the Locprior model was uninformative for most *K* values with the exception of *K* = 2 where the *r* statistic was closest to 1 (S1 Fig); we interpret *K* = 2 as the optimal solution (S4 Text).

At *K* = 2 the assignments reflected the major relationships identified in the cluster analysis (Figs 2B and S1). Individuals from the demersal spawning sites at Grebes Nest (BB65) and Domino Run, Labrador (DRL), the Saguenay River (SR) capelin in the St. Lawrence River estuary, those from the St. Lewis (SLL), Aguanus River (GSL) and Scotian Shelf (SS), and capelin from the Southeast Shoal (SES) were all strongly associated with one of the genetic groups (Cluster I). Within this group, mean membership probability by sample location was greater than 90% in all cases (individual range 71–100%). The second group (Cluster II) was not well resolved with all individuals and sampling locations admixed with Cluster I. On average sample locations in this second group displayed 60% proportional membership to Cluster II and 40% proportional membership to Cluster I. Samples with their highest proportional membership in each of Cluster I and Cluster II exactly correspond to the sample locations separated in the MDS and in the complete linkage dendrogram produced from Jost’s *D*_est_ (Fig 2B).

## Discussion

Our analyses of six microsatellite DNA loci, among 3,433 fish, collected across as many as 15 different spawning locations in the NWA, over seven years, and representing eleven year-classes, confirm high levels of genetic diversity in common with other marine species, and provide genetic evidence for population structuring of capelin in this area.

Allelic range and the average number of alleles per locus were comparable with those of Gordos et al. [26], especially when our much larger sample size and greater geographic coverage are taken into consideration. Three of our tetra-nucleotide loci were highly polymorphic overall due to their one, two, and four bp variations as reported previously [26]. Differences in allelic sizes are most commonly caused by the gain or loss of one or more of the microsatellite repeat units resulting in a uniform allelic size difference (e.g., four bp in perfect tetranucleotide loci), and are believed to arise through slipped-strand mispairing during DNA replication [53]. Many alleles and genotypes reflected this pattern in our study. However the one- and two-bp differences in sizes produced rare alleles in all samples, greatly increasing allelic diversity at such loci. Bull et al. [54] discuss three mechanisms that can produce complexity in interallelic variation such as observed here: 1) small insertions-deletions or single bp mutations in the flanking sequences (producing null alleles), 2) small insertions-deletions of one or two bp DNA motifs within the repeat unit (interrupted microsatellites), and 3) small insertion-deletions within all or some of the repeat units of compound loci. Without sequence information for each individual variant, we cannot specify which of these mechanisms may have produced the high number of alleles in loci *Mvi*2, *Mvi*9 and *Mvi*16, although of these, only *Mvi*2 is a compound locus. However, we speculate that those loci likely represent interrupted microsatellites. Interrupted microsatellites are characterized by a higher variance in repeat number and considered useful for population genetic studies [55], although they are more commonly used for phylogenetic analyses when they are highly conserved [56, 57]. Very rare alleles such as observed in this study are not very informative for assessing genetic structure among populations [58] and both *F*_ST_ and *D*_est_ are most heavily influenced by the frequency of the common alleles [39, 59]. This was shown to be the case here, where estimates of genetic differentiation as measured by *D*_est_ were largely unaffected by removing rare alleles from the analyses.

The patterns that emerged from our analyses suggest subtle population structure within this phylogenetic clade, as opposed to a single panmictic population. This is evidenced by pair-wise tests of significance of the null hypothesis of panmixia that revealed genetic differentiation between demersal and beach spawning capelin, even when samples from the two spawning groups were in close proximity at two nearshore demersal spawning locations. Spatial genetic variation from the Barrier analyses showed relative isolation of the capelin in the Saguenay River, from Unity Bay, Labrador and from Domino Run, Labrador, and distinguished capelin from the Gulf of St. Lawrence and Scotian Shelf (GSL, SLL, SS) from those in south and eastern Newfoundland-Grand Bank (BB,BB61,CC, LL, SES, TW). Despite low levels of differentiation, we were able to detect structuring with Bayesian analyses which supported two basal groups corresponding to samples which formed clusters based on Jost’s *D*_est_. Given that the Bayesian analyses fit a model of genetic data based upon assumptions in populations mating at random, and Jost’s *D*_est_ is based on allele identities, the mutual reinforcement of the genetic patterns that each analysis provides increases our confidence in our conclusions. At least some of the spatial variation revealed by the Barrier analyses can be explained by the sample mosaic pattern produced through interspersion of fish with ancestors from the two basal groups.

Temporal variation in allele frequencies within locations occurred, and our results highlight three strongly differentiated temporal samples from three of the sample locations. This could be due to generational effects produced by the short life span of capelin, occasional incursion of capelin of a different basal population, or other unknown factors.

The spatial and temporal differentiation results were evident with both multilocus θ, and when using Jost’s *D*_est_. Our values of multilocus θ were less than that observed amongst capelin samples using comparable statistics in the NEA where *F*_ST_ ranged from -0.001 to 0.027. However, the degree of correct self-assignment (~13%, S3 Text) is within the range reported previously [17] for non-spawning fish from locations in the NEA (11–40%).

Capelin stock structure in the NWA used by fisheries management is based on meristic and morphometric data and on distribution and migration patterns. Capelin inhabiting Divisions 4RST (Fig 1), spawn on beaches throughout the Gulf of St. Lawrence including the St. Lawrence River estuary [22] and are considered a management stock. Two capelin stocks are assumed to inhabit the Grand Banks: one distributed to the north across Divisions 2GHJ and 3KL (see Fig 1) that forages offshore and migrates inshore to spawn predominantly on beaches in Newfoundland and Labrador [24, 60] and one distributed to the south across Divisions 3NO that also forages offshore and spawns offshore at depths of ~50 m on the Southeast Shoal of the Grand Bank [6]. A fourth stock inhabiting Division 3Ps is presumed to reside on St. Pierre Bank and to spawn on beaches in southern Newfoundland [22]. Our study is the first to relate this biologically-based stock structure to population genetic structure.

Our genetic population structure results show general agreement with the stock structure in Divisions 4RST but extends the stock influence to include those fish on the Scotian Shelf (SS) and St. Lewis, Labrador (SLL) near the northern entrance to the Strait of Belle Isle. This result is strongly supported by the Bayesian clustering method implemented in Structure which assigned individuals from these locations (SR, SS, GSL, SLL) to the same basal unit with high probability, and by pairwise sample differentiation based on θ and Jost’s *D*_est_, as well as the nMDS and complete linkage clustering of samples based on *D*_est_. Capelin have been recruiting to the Scotian Shelf in recent years [2, 15] where they are known to spawn on Western Bank based on larval fish collections [61]. This putative ‘stock’ has been increasing in abundance since 1987 coincident with shifts in distribution [15]. Our genetic results indicate that the origin of the capelin spawning on the Scotian Shelf is likely the result of immigration of capelin from the Gulf of St. Lawrence. To the north, evidence of genetic similarity among some beach spawning locations within the northern Gulf of St. Lawrence (GSL) and southern Labrador (SLL) is not surprising considering the close proximity of the two conventional stock complexes via the Strait of Belle Isle (Fig 1). Bayesian clustering analyses further suggest that capelin from the Gulf of St. Lawrence may share ancestry with capelin from the demersal spawning site in Domino Run, Labrador (DRL).

Roby et al. [23] reported that capelin in the northern Gulf of St. Lawrence were different from capelin found elsewhere in the Gulf which differs from Colbeck et al. [13] who report that capelin throughout the Gulf of St. Lawrence, including the estuary, were similar except for fish from the Saguenay Fjord. Our samples are not sufficiently representative of the region to validate either of these previous studies, but our results show that relatively high genetic similarity occurs among capelin spawning in the northern Gulf and some spawning beaches adjacent to the Newfoundland and Labrador shelves, and distinguishes capelin spawning in the Saguenay River. Further the sample from the Saguenay River in the St. Lawrence estuary separated from the other samples in the nMDS and showed a large distance from the other samples in the cluster analysis (Fig 2B). Spatial analyses distinguished capelin spawning in the Saguenay River from those spawning at the Aguanus River at 4 of 6 loci, consistent with Colbeck et al. [13] and Bayesian assignment patterns at *K* > 2 (S2 Fig) are somewhat suggestive of weak hierarchical clustering within the basal population assignment that might be elucidated with a greater number of loci and/or samples from the area.

Genetic data from this study indicate four population complexes in the area of Divisions 2J and 3KL (Fig 1). The analyses collectively indicate a south and east coast Newfoundland stock including capelin in Division 3L (BB, CC, BB61), 3P_S_ (LL), extending at least as far north as Twillingate (TW) in 3K. Tag-return data indicate that pre-spawning aggregations of capelin on the east coast of Newfoundland represent a mixture of capelin distributed across several coastal embayments along the south and east coast of Newfoundland [60].

The demersal capelin spawning on the Southeast Shoal in NAFO Div. 3NO (SES) has been differentiated previously from beach spawners collected in coastal Newfoundland based on meristic and morphometric data [21, 22] and our results support this interpretation. Capelin are known to spawn demersally on the Southeast Shoal [6] and they are managed as a separate stock. Our spatial analyses indicate that there is genetic similarity between these offshore demersal spawners and those fish spawning on the beach at Bellevue on the east coast of Newfoundland. However, the Southeast Shoal capelin are on the periphery of the sampling area, and their spatial relationship with the samples from the south and east coast of Newfoundland was only tested at a single barrier due to the spatial configuration of the Delauney triangulation. Pairwise *D*_est_ shows relatively high genetic differentiation (*D*_est_ ≥ 0.01) between the Southeast Shoal and two of the south and east coast of Newfoundland samples (demersal spawning sites at Grebe’s Nest and Gull Rock, Newfoundland) from within that genetic population. We also observed a relatively low level of genetic differentiation between the Southeast Shoal and south and east coast of Newfoundland samples based on θ that was statistically significant but not outside the 95% confidence interval of θ. Lower genetic differentiation could be due to a number of factors, including large population size, recent separation or incursion, occasional homogenization, etc. Immature capelin originating from southern and eastern Newfoundland beaches and from demersal spawning grounds on the Southeast Shoal co-habit the northern Grand Bank [62]. When mature, pre-spawners separate and either migrate inshore in the spring towards beaches along the south and east coast of Newfoundland or remain offshore to spawn on the Southeast Shoal [18], which could facilitate gene flow between these areas and contribute to the observed level of differentiation. It may also be that recent shifts in capelin distribution beginning in the early 1990s [2] have changed spawning migrations such that capelin from the same stock complex are now spawning on the Southeast Shoal and in coastal areas of Newfoundland, similar to the southerly movement of capelin from the Gulf of St. Lawrence onto the Scotian Shelf that we alluded to above reducing the genetic distinction between offshore and inshore capelin in this region. Bayesian clustering clearly placed the Southeast Shoal capelin in a basal population with capelin from the Gulf of St. Lawrence, a result that also emerged from Jost’s *D*_est_ when visualized in the nMDS and complete linkage clustering analyses. Pairwise analyses of differentiation in θ support are consistent with the above in that no differences were found between SES and those samples associated with the genetic population centered in the Gulf of St. Lawrence (SLL, SS, BB65 and GSL). The spatial mosaic pattern of basal populations would distinguish the Southeast Shoal capelin spatially from the beach spawners collected in coastal Newfoundland, consistent with results based on meristic and morphometric data [21, 22]. Therefore collectively our analyses suggest that the capelin from the Southeast Shoal are genetically differentiated from those on the south and east coast of Newfoundland, however the differences may be in the process of changing. It may be useful to collect capelin samples from the Southeast Shoal and from coastal Newfoundland to compare more recent meristic and morphometric data to the results from earlier studies [21, 22].

To the north, a third population is formed by the Red Bay Beach (RBB; Division 2J) and Straitsview (SV; Division 3K) beach spawning samples which group together in both Bayesian clustering and nMDS based on Jost’s *D*_est_. The separation of these two samples in the spatial analysis is explained by the St. Lewis (SLL) sample, with its affinities to the Gulf of St. Lawrence, situated between the two. Further north, capelin from the demersal spawning location at Domino Run, Labrador (DRL) were genetically differentiated from beach spawning capelin samples collected to the north (Unity Bay, Labrador; UB) and south (RBB) creating two populations in this area; a result consistent with the spatial organization of the two basal populations identified in the Bayesian analyses. However this was one of the locations for which we were unable to analyze temporal variability and so this uniqueness (and that of other single collection samples i.e., BB65, SR and SLL) may not be representative of the genetic diversity of these fish if our collection in that year was atypical. The capelin collected over three years from Unity Bay have relatively high levels of genetic differentiation from neighbouring samples to the south which are apparent in five of the six loci in barrier-iii in barrier-v of the Barrier analyses. Further, the three temporal replicates from this location were undifferentiated. It is possible that capelin from UB may be more related to capelin found further north and west as they are adjacent to the region associated with the Arctic phylogenetic clade [12]. Capelin from the Arctic may have migrated south and mixed with capelin off northern Labrador or perhaps *vice versa*. It is unlikely that Unity Bay capelin are mixing with those from west Greenland as the latter have been genetically differentiated from NWA capelin [12, 13, 17].

Colbeck et al. [13] also detected genetic structure in NWA capelin using amplified fragment length polymorphism (AFLP) nuclear markers thought to be under selective pressure. In their study one group was located in the St. Lawrence River Estuary and Gulf and another included samples from the Saguenay Fjord, Newfoundland and Hudson Bay. Our analyses with “neutral” microsatellite DNA markers aligned with their first group in the Gulf of St. Lawrence in as far as a comparison with different sampling locations can be made. We found a high degree of genetic differentiation amongst locations at the mouth of the Saguenay River (SR) and those on the south and east coast of Newfoundland and Labrador as discussed above, and we further differentiated four distinct populations on the south and east coast of Newfoundland and coastal Labrador in contrast to Colbeck et al. [13]. However, if the geographic context of the Colbeck et al. [13] samples is reviewed, their Saguenay Fjord sample was significantly different from three of its four nearest neighbours (Sept-Îles, Saint-Irénée and Newport, Quebec, Canada). Although our closest Gulf sample to this location was on the north shore of the Gulf of St. Lawrence at Aguanus River (GSL), we also found it to be significantly differentiated from our Saguenay River (SR) sample. Therefore it is possible that the Saguenay River Fjord represents a locally adapted population of capelin with the environment selecting against migrants, although Colbeck et al. [13] were not able to attribute AFLP banding patterns to either temperature or salinity. In northern Norway, beach spawning capelin in Balsfjorden complete their life cycle within the fjord [63] presenting a possible mechanism for the high degree of differentiation observed between this population and the others, including neighbouring fish in the Gulf of St. Lawrence from whence they are likely derived according to our analyses.

An intriguing result of our analyses is that capelin spawning on one of the nearshore demersal spawning sites demonstrated relatively high genetic differentiation from capelin spawning on neighbouring beaches, although they are geographically separated by less than five km; i.e., Grebes Nest (BB65) with Bellevue Beach (BB) and with Chance Cove (CC). The genetic differentiation of BB65 was very well supported by four, five and six of the six loci in each of its three barrier segments in the first barrier. The nearby Gull Rock (BB61) demersal spawning site is 2.5 km from Bellevue Beach (BB) and was not differentiated from the same neighbouring spawning beaches and was differentiated from BB65 in the barrier analyses, Bayesian clustering and in pair-wise population differentiation. We may speculate if capelin are unable to spawn on Bellevue Beach because of warming water temperatures [8], these fish may be preferentially spawning demersally on Gull Rock (BB61) rather than on Grebes Nest (BB65). Genetic sampling over several years at Grebes Nest (BB65) and adjacent sites (BB61, BB) would facilitate a test for temporal variability. Bayesian clustering indicates that the capelin spawning at Grebe’s Nest are from the same basal population as those from the Gulf of St. Lawrence, and the demersal spawning capelin from the Scotian Shelf, Southeast Shoal, and Domino Run, Labrador sample sites.

Our other demersal spawning locations were far offshore (SES, SS) or not in the vicinity of sampled spawning beaches (DRL) and so we were unable to directly compare those locations to nearby beaches. We note that collectively, demersally spawning fish were genetically distinct from beach spawners in pair-wise tests of differentiation, but those test results were not as compelling as the direct comparison of fish in the vicinity of Bellevue Beach. Præbel et al. [17] were unable to differentiate capelin spawning on beaches in Balsfjorden in northern Norway from demersal spawners collected at two locations in the Barents Sea even though Balsfjorden capelin apparently do not leave the fjord [63]. In contrast, larval capelin from beach and demersal spawning sites along coastal Newfoundland migrate offshore where they mix, grow, and mature before migrating inshore to spawn [24, 60, 62]. Identification of other coastal demersal spawning sites (e.g., [9]) and comparable genetic studies could elucidate a more extensive pattern of genetically differentiated demersal spawning sites and more importantly how they are maintained [64].

The results of our study provide genetically-based evidence that capelin in the NWA comprise several populations that is consistent, to varying extents, with other studies using different data and metrics. Some are composed of multiple beach and demersal spawning locations such as the Gulf of St. Lawrence and the Scotian Shelf, and the south and east coasts of Newfoundland with capelin spawning at Gull Rock, Newfoundland. Others consist of one beach (Saguenay River, Unity Bay) or one demersal (Domino Run, Grebes Nest, Southeast Shoal) spawning location. Our interpretation, especially for those sites that were sampled in a single year (Saguenay River, Domino Run, Grebes Nest, St. Lewis Labrador), is tempered by our inability to test for temporal variability at all locations for all analyses due to our asymmetrical sampling of years and locations, which we did find at three sampling locations (Chance Cove, Little Lawn, Straitsview). Nevertheless, the identification of genetic differentiation among capelin collections in the northwest Atlantic Ocean, likely arising from two ancestral populations, adds to the growing literature demonstrating spatial genetic heterogeneity in ecologically similar marine fish species such as herring, *Clupea harengus* [35, 65–67].

## Supporting Information

S1 Fig Trends in parameters of Bayesian clustering models under simulations of *K* (1–5). Mean log likelihood of the data (*Ln P(D)*), admixture coefficient (*α*) and model fit (*r*) (± 95% confidence interval) for each *K* value.
(TIF)

S2 Fig Structure bar plots of posterior probabilities of group assignment. Bar plots displaying the genetic clustering relationships of capelin from 15 sample locations (Table 1 in the publication) under the Locprior model. *K* is the number of hypothetical clusters each represented by a different colour. The best alignment of the results of individual computer runs for each *K* is presented. Proportional genetic assignment for each capelin is represented by a vertical bar and shown under simulations for *K* = 2, 3, 4, 5, 6, 7, 10 and 12. The plots are organized by grouping individuals by sample location.
(TIF)

S1 Table Sample Summary Statistics. Nei’s unbiased expected heterozygosity (*H*_*E*_), observed heterozygosity (*H*_*O*_) and the inbreeding coefficient (*F*_*IS*_) for each of 45 capelin samples at each of 6 loci and overall loci. Codes for samples are found in Table 1 of the publication. Bold text indicates a significant deviation from HWE (*P* ≤ 0.05). * Indicates significant deviation from HWE after correcting for multiple tests (*P* ≤ 0.0002).
(DOCX)

S2 Table Capelin Microsatellite DNA Allelic Size Data Set. Allelic sizes (xxx base pairs) at six microsatellite loci in capelin collected at 15 locations (sample codes in Table 1 of the publication) including samples from 3 locations that were found to be temporally distinct.
(DOCX)

S3 Table Summary Statistics of Capelin at 6 Loci across 18 Samples Used for Genetic Analyses. Details of capelin at 6 loci (*Mvi*) across 18 samples (see Table 1 of the publication for codes) with varying number of individuals in a sample (*N*) and associated genetic diversity summary statistics that include the number of alleles observed (*NA*) for each locus, allelic richness (*RS*) for each locus, the number of private alleles (*PS*) based on a minimum sample size of 38 individuals, expected heterozygosity (*H*_*E*_), observed heterozygosity (*H*_*O*_) and the inbreeding coefficient (*F*_*IS*_). *SD* = standard deviation.
(DOCX)

S4 Table Jack-knifed Weir and Cockerham Fixation Indices and Jost’s *D*est. Re-sampled indices using a jack-knife on the six loci and 18 samples to produce variance estimates and to evaluate the influence of the selection of loci and/or samples on the mean values of parameters. *F*_IS_ expresses the variation between individuals within populations; *F*_IT_ expresses the variation within individuals; *F*_ST_ θ expresses the variation among populations. Jost’s *D*_est_ is estimated for each locus (no jack-knife) and overall (Chao’s harmonic mean). 95% confidence intervals were constructed through 1,000 bootstraps for each locus.
(DOCX)

S5 Table Effect of Rare Alleles on *D*_est_. *D*_est_ for six microsatellite loci applied to 18 capelin samples. Values calculated using all allele frequencies are compared with those calculated after removing rare alleles (alleles with average frequency across samples of less than 0.05% or 0.01% dependent on the frequency distribution). Confidence intervals were generated through 500 permutations of the data.
(DOCX)

S6 Table Performance of Bayesian Clustering Models. Summary statistics of *N* replicates (mean, standard deviation) for the log likelihood of the data (*Ln P(D)*), the degree of admixture (*α*) and model fit (*r*) from Markov chain Monte Carlo (MCMC) data collection chains of 500,000 steps each, run under 1 to 15 assumed populations (*K*).
(DOCX)

S1 Text Quality Control of Data. (DOCX)

S2 Text Preliminary Genetic Analyses: Methods and Results. (DOCX)

S3 Text Individual Assignment Tests: Methods and Results. (DOCX)

S4 Text Bayesian Clustering of Individuals with Structure: Methods and Results. (DOCX)
