# Supplementary figures and images for: Genetic Structure of Capelin (Mallotus villosus) in the Northwest Atlantic Ocean
Source: PLoS One. 2015 Mar 30;10(3):e0122315. doi: 10.1371/journal.pone.0122315 (PMC4378951; doi:10.1371/journal.pone.0122315)

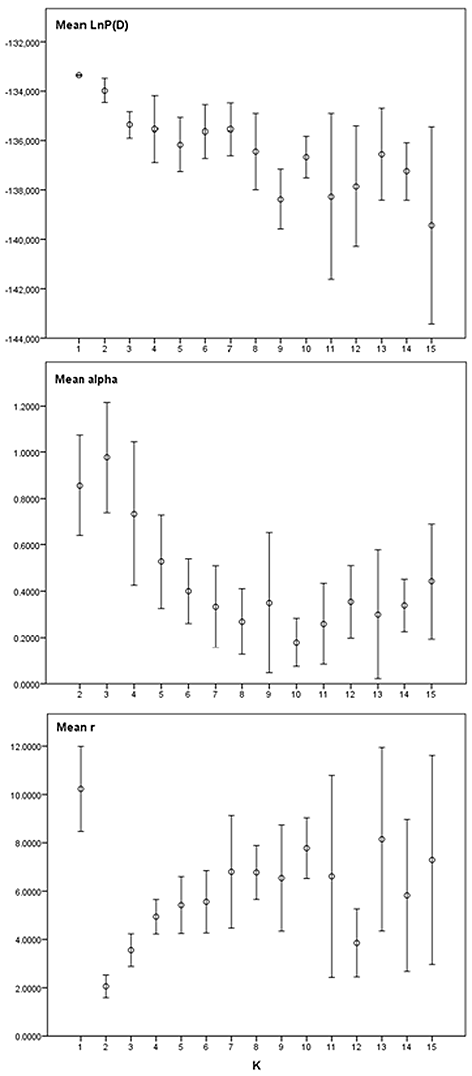

Supplement: S1 Fig — Mean log likelihood of the data (Ln P(D)), admixture coefficient (α) and model fit (r) (± 95% confidence interval) for each K value. (TIF) [file pone.0122315.s001.tif]

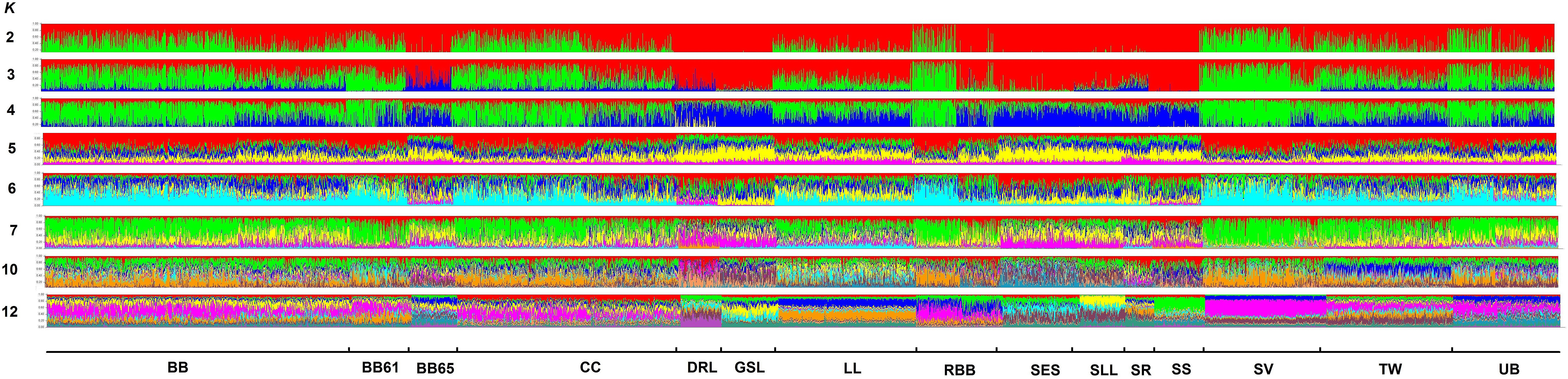

Supplement: S2 Fig — Bar plots displaying the genetic clustering relationships of capelin from 15 sample locations (Table 1 in the publication) under the Locprior model. K is the number of hypothetical clusters each represented by a different colour. The best alignment of the results of individual computer runs for each K is presented. Proportional genetic assignment for each capelin is represented by a vertical bar and shown under simulations for K = 2, 3, 4, 5, 6, 7, 10 and 12. The plots are organized by grouping individuals by sample location. (TIF) [file pone.0122315.s002.tif]
