# Supplementary material for: Genetic Structure of Capelin (Mallotus villosus) in the Northwest Atlantic Ocean
Source: PLoS One. 2015 Mar 30;10(3):e0122315. doi: 10.1371/journal.pone.0122315 (PMC4378951; doi:10.1371/journal.pone.0122315)
Supplement: S1 Table — Nei’s unbiased expected heterozygosity (H E), observed heterozygosity (H O) and the inbreeding coefficient (F IS) for each of 45 capelin samples at each of 6 loci and overall loci. Codes for samples are found in Table 1 of the publication. Bold text indicates a significant deviation from HWE (P ≤ 0.05). * Indicates significant deviation from HWE after correcting for multiple tests (P ≤ 0.0002). (DOCX) [file pone.0122315.s003.docx]

**S1 Table.** **Sample Summary Statistics**.

| **Sample Code (YYYYCode)** | **N** | ***Mvi*2** | | | ***Mvi*3** | | | ***Mvi*5** | | | ***Mvi*9** | | |
| --- | --- | --- | --- | --- | --- | --- | --- | --- | --- | --- | --- | --- | --- |
|  |  | ***H_E_*** | ***H_O_*** | ***F_IS_*** | ***H_E_*** | ***H_O_*** | ***F_IS_*** | ***H_E_*** | ***H_O_*** | ***F_IS_*** | ***H_E_*** | ***H_O_*** | ***F_IS_*** |
| 2002BB | 93 | 0.961 | 0.925 | 0.038 | 0.922 | 0.914 | 0.009 | 0.873 | 0.828 | 0.051 | **0.979** | **0.828** | 0.155 |
| 2003BB | 97 | 0.964 | 0.918 | 0.048 | 0.921 | 0.918 | 0.004 | **0.868** | **0.814** | *0.062 | 0.981 | 0.928 | 0.054 |
| 2004BB | 225 | **0.959** | **0.916** | 0.045 | 0.928 | 0.924 | 0.004 | 0.876 | 0.858 | 0.021 | 0.977 | 0.916 | 0.063 |
| 2005BB | 47 | 0.966 | 0.936 | 0.031 | 0.926 | 0.894 | 0.035 | 0.875 | 0.851 | 0.027 | 0.984 | 0.957 | 0.028 |
| 2006BB | 72 | 0.960 | 0.931 | 0.031 | **0.927** | **0.875** | 0.057 | **0.880** | **0.861** | 0.022 | 0.980 | 0.944 | 0.036 |
| 2007BB | 48 | 0.963 | 0.938 | 0.027 | 0.926 | 0.896 | 0.033 | 0.874 | 0.813 | 0.071 | 0.977 | 0.938 | 0.041 |
| 2008BB | 72 | 0.966 | 0.903 | 0.066 | 0.927 | 0.958 | -0.034 | 0.894 | 0.889 | 0.006 | **0.982** | **0.944** | 0.039 |
| 2004BB61 | 63 | 0.965 | 0.937 | 0.030 | 0.936 | 0.905 | 0.034 | 0.884 | 0.921 | -0.041 | **0.980** | **0.825** | *0.158 |
| 2006BB61 | 65 | 0.959 | 0.939 | 0.021 | **0.925** | **0.862** | 0.069 | 0.885 | 0.892 | -0.008 | 0.980 | 0.877 | 0.106 |
| 2005BB65 | 98 | **0.968** | **0.929** | 0.041 | 0.923 | 0.888 | 0.039 | 0.861 | 0.867 | -0.008 | 0.980 | 0.929 | 0.053 |
| 2002CC | 94 | 0.963 | 0.926 | 0.039 | 0.930 | 0.883 | 0.050 | 0.886 | 0.915 | -0.033 | 0.980 | 0.936 | 0.045 |
| 2003CC | 98 | 0.956 | 0.959 | -0.004 | 0.922 | 0.929 | -0.007 | 0.876 | 0.888 | -0.013 | 0.980 | 0.939 | 0.043 |
| 2004CC | 95 | 0.964 | 0.916 | 0.050 | **0.926** | **0.905** | 0.023 | 0.863 | 0.863 | -0.001 | 0.977 | 0.947 | 0.031 |
| 2005CC | 50 | 0.959 | 0.940 | 0.020 | 0.923 | 0.920 | 0.004 | 0.870 | 0.940 | -0.081 | **0.971** | **0.900** | 0.074 |
| 2006CC | 70 | 0.966 | 0.929 | 0.039 | 0.925 | 0.986 | -0.067 | 0.869 | 0.800 | 0.080 | 0.982 | 0.943 | 0.040 |
| 2007CC | 65 | 0.954 | 0.923 | 0.032 | 0.921 | 0.923 | -0.002 | 0.889 | 0.862 | 0.031 | **0.980** | **0.892** | 0.090 |
| 2008CC | 58 | 0.943 | 0.914 | 0.031 | 0.928 | 0.897 | 0.034 | 0.873 | 0.845 | 0.033 | 0.979 | 0.914 | 0.067 |
| 2006DRL | 88 | 0.965 | 0.921 | 0.047 | 0.918 | 0.898 | 0.023 | 0.875 | 0.830 | 0.052 | **0.980** | **0.898** | 0.084 |
| 2006GSL | 57 | 0.955 | 0.930 | 0.026 | 0.929 | 0.912 | 0.018 | 0.870 | 0.860 | 0.012 | 0.980 | 0.965 | 0.016 |
| 2007GSL | 28 | **0.958** | **0.893** | 0.070 | 0.924 | 0.893 | 0.034 | 0.881 | 0.821 | 0.069 | **0.984** | **0.857** | *0.131 |
| 2008GSL | 37 | **0.949** | **0.838** | *0.119 | 0.927 | 0.946 | -0.020 | 0.874 | 0.919 | -0.052 | 0.982 | 0.973 | 0.009 |
| 2003LL | 96 | 0.960 | 0.896 | 0.067 | 0.929 | 0.958 | -0.032 | 0.869 | 0.792 | 0.089 | 0.982 | 0.979 | 0.003 |
| 2004LL | 97 | **0.965** | **0.928** | 0.038 | 0.927 | 0.918 | 0.011 | 0.867 | 0.825 | 0.049 | **0.977** | **0.897** | 0.082 |
| 2006LL | 69 | 0.971 | 0.942 | 0.030 | 0.920 | 0.928 | -0.008 | 0.872 | 0.870 | 0.002 | 0.986 | 0.928 | 0.059 |
| 2007LL | 62 | 0.955 | 0.952 | 0.004 | 0.915 | 0.839 | 0.084 | 0.866 | 0.871 | -0.006 | 0.983 | 0.984 | -0.001 |
| 2008LL | 68 | 0.961 | 0.971 | -0.010 | 0.933 | 0.882 | 0.054 | 0.884 | 0.838 | 0.052 | 0.980 | 0.912 | 0.070 |
| 2004RBB | 99 | 0.961 | 0.909 | 0.054 | 0.929 | 0.899 | 0.032 | 0.877 | 0.849 | 0.032 | 0.978 | 0.950 | 0.029 |
| 2007RBB | 87 | 0.964 | 0.977 | -0.013 | 0.930 | 0.908 | 0.024 | 0.857 | 0.885 | -0.034 | 0.984 | 0.931 | 0.054 |
| 2006SES | 101 | 0.965 | 0.941 | 0.026 | 0.925 | 0.901 | 0.026 | 0.879 | 0.891 | -0.014 | **0.977** | **0.931** | 0.048 |
| 2007SES | 64 | **0.962** | **0.969** | -0.007 | 0.925 | 0.953 | -0.031 | 0.866 | 0.875 | -0.010 | 0.975 | 0.922 | 0.055 |
| 2006SLL | 98 | 0.956 | 0.929 | 0.028 | 0.922 | 0.949 | -0.029 | 0.882 | 0.867 | 0.016 | 0.981 | 0.959 | 0.023 |
| 2007SR | 62 | 0.957 | 0.919 | 0.040 | 0.927 | 0.903 | 0.025 | 0.867 | 0.839 | 0.033 | 0.984 | 0.919 | 0.066 |
| 2006SS | 55 | 0.966 | 0.946 | 0.022 | 0.929 | 0.946 | -0.018 | 0.878 | 0.855 | 0.027 | 0.978 | 0.927 | 0.052 |
| 2007SS | 54 | 0.966 | 0.982 | -0.017 | 0.932 | 0.889 | 0.046 | 0.858 | 0.852 | 0.007 | **0.983** | **0.907** | 0.077 |
| 2003SV | 99 | 0.958 | 0.950 | 0.009 | 0.927 | 0.909 | 0.020 | 0.870 | 0.859 | 0.013 | **0.980** | **0.939** | 0.042 |
| 2004SV | 97 | 0.960 | 0.918 | 0.044 | 0.923 | 0.887 | 0.039 | 0.848 | 0.876 | -0.034 | 0.977 | 0.928 | 0.050 |
| 2005SV | 39 | 0.972 | 0.923 | 0.051 | **0.933** | **0.897** | 0.039 | 0.883 | 0.872 | 0.013 | 0.971 | 0.872 | 0.104 |
| 2006SV | 64 | 0.964 | 0.875 | 0.093 | 0.931 | 0.922 | 0.009 | 0.871 | 0.859 | 0.014 | 0.980 | 0.922 | 0.060 |
| 2002TW | 98 | 0.959 | 0.929 | 0.032 | 0.928 | 0.888 | 0.043 | 0.877 | 0.837 | 0.046 | 0.979 | 0.918 | 0.062 |
| 2006TW | 59 | 0.955 | 0.983 | -0.029 | 0.929 | 0.949 | -0.022 | 0.878 | 0.881 | -0.004 | **0.979** | **0.915** | 0.066 |
| 2007TW | 54 | 0.974 | 0.926 | 0.050 | 0.921 | 0.944 | -0.025 | 0.867 | 0.907 | -0.047 | 0.983 | 0.907 | 0.077 |
| 2008TW | 61 | **0.963** | **0.902** | 0.064 | 0.932 | 0.902 | 0.032 | 0.889 | 0.934 | -0.051 | 0.981 | 0.918 | 0.064 |
| 2004UB | 95 | 0.961 | 0.905 | 0.058 | **0.925** | **0.874** | 0.056 | 0.869 | 0.853 | 0.019 | **0.977** | **0.937** | 0.042 |
| 2005UB | 88 | 0.961 | 0.955 | 0.006 | 0.928 | 0.886 | 0.045 | 0.882 | 0.875 | 0.008 | **0.983** | **0.898** | 0.087 |
| 2007UB | 47 | 0.961 | 0.915 | 0.048 | 0.924 | 0.851 | 0.080 | 0.889 | 0.894 | -0.005 | 0.983 | 1.000 | -0.017 |

**S1 Table.** Continued.

| **Sample Code (YYYYCode)** | **N** | ***Mvi*10** | | | ***Mvi*16** | | | **Overall Loci** | | |
| --- | --- | --- | --- | --- | --- | --- | --- | --- | --- | --- |
|  |  | ***H_E_*** | ***H_O_*** | ***F_IS_*** | ***H_E_*** | ***H_O_*** | ***F_IS_*** | ***H_E_*** | ***H_O_*** | **Average No. of Alleles/Locus** |
| 2002BB | 93 | 0.948 | 0.989 | -0.044 | **0.973** | **0.882** | *0.094 | 0.943 | 0.894 | 36.83 |
| 2003BB | 97 | 0.951 | 0.897 | 0.057 | 0.978 | 0.907 | 0.072 | 0.944 | 0.897 | 38.50 |
| 2004BB | 225 | 0.943 | 0.920 | 0.025 | **0.978** | **0.880** | 0.101 | 0.944 | 0.902 | 48.50 |
| 2005BB | 47 | 0.948 | 0.957 | -0.010 | **0.976** | **0.851** | *0.129 | 0.946 | 0.908 | 31.50 |
| 2006BB | 72 | **0.952** | **0.931** | *0.023 | 0.980 | 0.847 | 0.136 | 0.946 | 0.898 | 35.33 |
| 2007BB | 48 | 0.946 | 0.979 | -0.036 | 0.983 | 0.938 | 0.047 | 0.945 | 0.917 | 31.33 |
| 2008BB | 72 | **0.946** | **0.944** | 0.001 | 0.980 | 0.944 | 0.037 | 0.949 | 0.931 | 36.50 |
| 2004BB61 | 63 | 0.946 | 0.937 | 0.010 | **0.978** | **0.937** | 0.042 | 0.948 | 0.910 | 32.83 |
| 2006BB61 | 65 | 0.945 | 0.954 | -0.010 | **0.977** | **0.831** | 0.150 | 0.945 | 0.892 | 34.67 |
| 2005BB65 | 98 | 0.942 | 0.980 | -0.040 | 0.982 | 0.867 | 0.118 | 0.943 | 0.910 | 42.17 |
| 2002CC | 94 | 0.944 | 0.979 | -0.037 | 0.978 | 0.957 | 0.021 | 0.947 | 0.933 | 38.17 |
| 2003CC | 98 | 0.943 | 0.939 | 0.004 | **0.983** | **0.908** | *0.077 | 0.943 | 0.927 | 38.17 |
| 2004CC | 95 | 0.945 | 0.895 | 0.053 | **0.973** | **0.895** | *0.081 | 0.941 | 0.904 | 37.50 |
| 2005CC | 50 | 0.943 | 0.900 | 0.046 | **0.974** | **0.940** | 0.035 | 0.940 | 0.923 | 28.50 |
| 2006CC | 70 | 0.947 | 0.900 | 0.050 | 0.981 | 0.900 | 0.083 | 0.945 | 0.910 | 36.50 |
| 2007CC | 65 | 0.947 | 0.923 | 0.025 | 0.985 | 0.969 | 0.016 | 0.946 | 0.915 | 37.17 |
| 2008CC | 58 | 0.942 | 0.931 | 0.012 | 0.982 | 0.966 | 0.017 | 0.941 | 0.911 | 32.00 |
| 2006DRL | 88 | 0.939 | 0.932 | 0.008 | 0.984 | 0.830 | 0.158 | 0.944 | 0.885 | 39.00 |
| 2006GSL | 57 | 0.948 | 0.895 | 0.056 | 0.984 | 0.895 | 0.091 | 0.944 | 0.909 | 33.33 |
| 2007GSL | 28 | 0.960 | 0.857 | 0.109 | **0.979** | **0.821** | 0.164 | 0.948 | 0.857 | 24.67 |
| 2008GSL | 37 | 0.932 | 0.865 | 0.073 | **0.978** | **0.568** | *0.423 | 0.940 | 0.851 | 25.33 |
| 2003LL | 96 | 0.954 | 0.906 | 0.050 | **0.981** | **0.896** | 0.087 | 0.946 | 0.905 | 38.50 |
| 2004LL | 97 | 0.941 | 0.938 | 0.003 | 0.977 | 0.928 | 0.050 | 0.942 | 0.906 | 35.83 |
| 2006LL | 69 | 0.948 | 0.971 | -0.025 | 0.983 | 0.913 | 0.072 | 0.947 | 0.925 | 37.67 |
| 2007LL | 62 | 0.943 | 0.919 | 0.025 | **0.980** | **0.823** | 0.161 | 0.940 | 0.898 | 32.50 |
| 2008LL | 68 | 0.942 | 0.868 | 0.080 | 0.983 | 0.941 | 0.043 | 0.947 | 0.902 | 34.67 |
| 2004RBB | 99 | 0.945 | 0.899 | 0.049 | 0.979 | 0.919 | 0.061 | 0.945 | 0.904 | 37.00 |
| 2007RBB | 87 | **0.943** | **0.908** | 0.038 | 0.979 | 0.954 | 0.026 | 0.943 | 0.927 | 40.17 |
| 2006SES | 101 | 0.951 | 0.921 | 0.032 | 0.983 | 0.901 | 0.083 | 0.947 | 0.914 | 41.83 |
| 2007SES | 64 | 0.949 | 0.938 | 0.012 | 0.980 | 0.922 | 0.059 | 0.943 | 0.930 | 33.33 |
| 2006SLL | 98 | 0.944 | 0.939 | 0.006 | **0.985** | **0.918** | 0.068 | 0.945 | 0.927 | 42.33 |
| 2007SR | 62 | 0.939 | 0.839 | 0.108 | **0.978** | **0.936** | *0.044 | 0.942 | 0.893 | 34.17 |
| 2006SS | 55 | 0.942 | 0.927 | 0.015 | 0.982 | 0.800 | 0.186 | 0.946 | 0.900 | 33.00 |
| 2007SS | 54 | 0.948 | 0.944 | 0.004 | **0.981** | **0.852** | 0.133 | 0.945 | 0.904 | 33.33 |
| 2003SV | 99 | 0.942 | 0.919 | 0.024 | 0.980 | 0.869 | 0.114 | 0.943 | 0.907 | 38.50 |
| 2004SV | 97 | 0.945 | 0.938 | 0.007 | 0.974 | 0.938 | 0.037 | 0.938 | 0.914 | 34.33 |
| 2005SV | 39 | 0.941 | 0.974 | -0.035 | **0.974** | **0.718** | 0.265 | 0.946 | 0.876 | 27.00 |
| 2006SV | 64 | 0.944 | 0.953 | -0.010 | 0.981 | 0.891 | 0.093 | 0.945 | 0.904 | 34.83 |
| 2002TW | 98 | 0.940 | 0.929 | 0.012 | **0.976** | **0.929** | 0.049 | 0.943 | 0.905 | 38.83 |
| 2006TW | 59 | 0.951 | 0.898 | 0.056 | 0.982 | 0.898 | 0.085 | 0.946 | 0.921 | 34.83 |
| 2007TW | 54 | 0.946 | 0.907 | 0.042 | **0.980** | **0.870** | 0.113 | 0.945 | 0.911 | 32.67 |
| 2008TW | 61 | 0.949 | 0.934 | 0.015 | 0.978 | 0.902 | 0.079 | 0.949 | 0.915 | 34.00 |
| 2004UB | 95 | 0.949 | 0.905 | 0.046 | 0.980 | 0.958 | 0.022 | 0.944 | 0.905 | 35.67 |
| 2005UB | 88 | 0.946 | 0.966 | -0.021 | **0.982** | **0.909** | 0.075 | 0.947 | 0.915 | 39.67 |
| 2007UB | 47 | 0.950 | 0.957 | -0.008 | 0.978 | 0.936 | 0.043 | 0.948 | 0.926 | 30.83 |

Nei’s unbiased expected heterozygosity (*H_E_*), observed heterozygosity (*H_O_*) and the inbreeding coefficient (*F_IS_*) for each of 45 capelin samples at each of 6 loci and overall loci. Codes for samples are found in Table 1 of the publication. Bold text indicates a significant deviation from HWE (*P* ≤ 0.05). * Indicates significant deviation from HWE after correcting for multiple tests (*P* ≤ 0.0002).
