# Supplementary material for: Genetic Structure of Capelin (Mallotus villosus) in the Northwest Atlantic Ocean
Source: PLoS One. 2015 Mar 30;10(3):e0122315. doi: 10.1371/journal.pone.0122315 (PMC4378951; doi:10.1371/journal.pone.0122315)
Supplement: S2 Table — Allelic sizes (xxx base pairs) at six microsatellite loci in capelin collected at 15 locations (sample codes in Table 1 of the publication) including samples from 3 locations that were found to be temporally distinct. (DOCX) [file pone.0122315.s004.docx]

**S2 Table. Capelin** **Microsatellite DNA Allelic Size Data Set.**

| **Sample Code** | **Loci** |  |  |  |  |  |
| --- | --- | --- | --- | --- | --- | --- |
|  | ***Mvi*2** | **Mvi3** | ***Mvi*5** | ***Mvi*9** | ***Mvi*10** | ***Mvi*16** |
| BB | 204208 | 162186 | 105125 | 108108 | 143159 | 160160 |
| BB | 190198 | 166202 | 105113 | 135164 | 143159 | 160160 |
| BB | 116204 | 174194 | 113117 | 142150 | 131139 | 172184 |
| BB | 172176 | 170206 | 105105 | 118126 | 159183 | 184184 |
| BB | 160218 | 162166 | 109117 | 113151 | 163179 | 184192 |
| BB | 148168 | 162202 | 109109 | 100188 | 167195 | 160200 |
| BB | 116158 | 178210 | 109121 | 130156 | 139167 | 180204 |
| BB | 152210 | 166190 | 097109 | 130130 | 151159 | 212212 |
| BB | 153228 | 198202 | 101105 | 114114 | 163179 | 180212 |
| BB | 156174 | 194194 | 105109 | 156197 | 147183 | 212212 |
| BB | 144160 | 182202 | 097125 | 114134 | 167183 | 212212 |
| BB | 156160 | 186194 | 109117 | 121135 | 151171 | 180216 |
| BB | 156168 | 162174 | 109113 | 138160 | 151175 | 184216 |
| BB | 186190 | 162238 | 117117 | 114114 | 151187 | 216216 |
| BB | 144156 | 178182 | 113125 | 156168 | 155195 | 212216 |
| BB | 132170 | 194206 | 101113 | 130171 | 159171 | 160220 |
| BB | 148152 | 170174 | 125133 | 109188 | 163191 | 216220 |
| BB | 140152 | 186198 | 105113 | 132140 | 139175 | 216224 |
| BB | 116156 | 178194 | 117121 | 109179 | 143195 | 180224 |
| BB | 172206 | 166186 | 097129 | 140155 | 115151 | 228228 |
| BB | 160190 | 170178 | 105121 | 175188 | 139167 | 160228 |
| BB | 156228 | 162190 | 117121 | 140148 | 159167 | 216228 |
| BB | 114194 | 166202 | 109109 | 192227 | 147179 | 172228 |
| BB | 148176 | 166166 | 105125 | 113172 | 147183 | 204229 |
| BB | 152164 | 170178 | 113125 | 129196 | 159179 | 208230 |
| BB | 176180 | 158166 | 109109 | 125151 | 163167 | 228231 |
| BB | 148148 | 162206 | 109113 | 105130 | 143155 | 180232 |
| BB | 148166 | 158174 | 117121 | 148148 | 147159 | 212232 |
| BB | 116144 | 182186 | 109129 | 113126 | 159179 | 230234 |
| BB | 165165 | 182190 | 109113 | 117125 | 187187 | 180234 |
| BB | 172188 | 178182 | 101117 | 122160 | 139151 | 216236 |
| BB | 186194 | 158174 | 097113 | 130138 | 171175 | 208236 |
| BB | 152160 | 166182 | 113121 | 171224 | 155175 | 236236 |
| BB | 144164 | 178182 | 105129 | 108134 | 151191 | 212238 |
| BB | 148214 | 182198 | 105137 | 114164 | 115143 | 208240 |
| BB | 124156 | 182182 | 113121 | 113152 | 135159 | 224240 |
| BB | 168220 | 166202 | 109121 | 138156 | 167171 | 200244 |
| BB | 144172 | 170198 | 109121 | 104165 | 171183 | 192244 |
| BB | 144206 | 194202 | 117125 | 140140 | 147155 | 225248 |
| BB | 148172 | 174182 | 109121 | 97126 | 171175 | 236248 |
| BB | 140164 | 206230 | 113133 | 135135 | 135179 | 160252 |
| BB | 168184 | 182218 | 109117 | 160223 | 139155 | 225258 |
| BB | 168172 | 178198 | 113117 | 100181 | 139159 | 225260 |
| BB | 116116 | 178186 | 105109 | 110156 | 123187 | 192260 |
| BB | 160227 | 170206 | 101113 | 140144 | 163195 | 160260 |
| BB | 188188 | 158162 | 113121 | 85142 | 131195 | 208260 |
| BB | 124160 | 162182 | 097113 | 141180 | 171223 | 224264 |
| BB | 116168 | 166190 | 113117 | 134188 | 131163 | 229268 |
| BB | 172196 | 190206 | 113113 | 131131 | 151183 | 232268 |
| BB | 140176 | 158166 | 097113 | 144144 | 131151 | 200272 |
| BB | 160220 | 166166 | 121125 | 144202 | 175179 | 160276 |
| BB | 156156 | 190190 | 109117 | 118122 | 151191 | 258276 |
| BB | 136144 | 166170 | 101129 | 101152 | 135167 | 248280 |
| BB | 168168 | 162166 | 105105 | 131229 | 167171 | 268280 |
| BB | 116136 | 182202 | 105125 | 156156 | 171175 | 220280 |
| BB | 164172 | 190202 | 105109 | 156212 | 139187 | 212280 |
| BB | 152164 | 174202 | 109113 | 135196 | 187207 | 276280 |
| BB | 164194 | 182182 | 105121 | 144144 | 183215 | 272280 |
| BB | 148200 | 162162 | 105133 | 132172 | 183215 | 280280 |
| BB | 156164 | 166174 | 117117 | 117148 | 143179 | 228282 |
| BB | 144202 | 178210 | 105117 | 113136 | 131155 | 158284 |
| BB | 164168 | 174206 | 105121 | 100112 | 155211 | 200284 |
| BB | 168172 | 182182 | 117117 | 110175 | 167171 | 232288 |
| BB | 176194 | 162170 | 105129 | 121160 | 167175 | 215288 |
| BB | 128194 | 166202 | 105125 | 130130 | 151175 | 244288 |
| BB | 156172 | 198202 | 117129 | 109131 | 207223 | 212288 |
| BB | 148160 | 190210 | 105113 | 126140 | 151155 | 216292 |
| BB | 180188 | 162186 | 101125 | 148176 | 155167 | 180292 |
| BB | 144176 | 166198 | 109113 | 138144 | 147183 | 284292 |
| BB | 140156 | 190194 | 117121 | 148192 | 183191 | 180292 |
| BB | 136198 | 166182 | 089101 | 148148 | 127159 | 236296 |
| BB | 168210 | 174178 | 105109 | 108122 | 151167 | 232296 |
| BB | 156160 | 190198 | 117121 | 135168 | 167175 | 188296 |
| BB | 136152 | 162182 | 105121 | 108193 | 175179 | 160296 |
| BB | 160164 | 166174 | 109129 | 126168 | 175179 | 288296 |
| BB | 172174 | 186190 | 109109 | 130160 | 179203 | 292296 |
| BB | 120160 | 174182 | 121133 | 131134 | 143183 | 208300 |
| BB | 160172 | 174190 | 101109 | 97126 | 159187 | 296300 |
| BB | 163192 | 166202 | 125125 | 117147 | 147167 | 252302 |
| BB | 164180 | 186190 | 105105 | 177242 | 119155 | 280304 |
| BB | 148156 | 166190 | 101117 | 117117 | 163183 | 212304 |
| BB | 166180 | 182186 | 105121 | 148156 | 151191 | 224312 |
| BB | 144194 | 190198 | 109109 | 155155 | 163195 | 276312 |
| BB | 164176 | 162166 | 097109 | 098184 | 167203 | 300312 |
| BB | 192222 | 178182 | 117117 | 144161 | 139163 | 188316 |
| BB | 168210 | 162202 | 105105 | 148148 | 159163 | 316316 |
| BB | 172182 | 186194 | 101105 | 136164 | 147159 | 244318 |
| BB | 136192 | 170178 | 109113 | 157175 | 143147 | 224320 |
| BB | 184226 | 158170 | 105121 | 126127 | 163175 | 224320 |
| BB | 168232 | 162178 | 109109 | 125156 | 135151 | 216332 |
| BB | 152156 | 162186 | 101105 | 98125 | 155171 | 260332 |
| BB | 152152 | 190210 | 109121 | 148152 | 151159 | 216380 |
| BB | 116144 | 170186 | 101105 | 108143 | 143199 | 228384 |
| BB | 148148 | 170182 | 105113 | 143148 | 139147 | 172172 |
| BB | 116144 | 162170 | 109137 | 097140 | 147179 | 172172 |
| BB | 188206 | 186194 | 101109 | 104152 | 151179 | 172172 |
| BB | 136168 | 178178 | 113125 | 126156 | 139171 | 148180 |
| BB | 176198 | 170182 | 101109 | 121196 | 147151 | 186186 |
| BB | 116166 | 162170 | 105117 | 98144 | 163171 | 180192 |
| BB | 160160 | 174186 | 105117 | 113200 | 151159 | 176200 |
| BB | 156202 | 186194 | 105109 | 147155 | 163179 | 200200 |
| BB | 116160 | 170186 | 109129 | 097127 | 175175 | 204204 |
| BB | 163164 | 170182 | 109109 | 097108 | 155163 | 192212 |
| BB | 144152 | 170174 | 113121 | 118134 | 171203 | 188212 |
| BB | 132160 | 182202 | 105109 | 152152 | 123131 | 162216 |
| BB | 180262 | 182198 | 117117 | 129168 | 139143 | 168216 |
| BB | 116168 | 178182 | 113125 | 144144 | 163179 | 212216 |
| BB | 164210 | 198202 | 101113 | 131137 | 183203 | 204216 |
| BB | 152186 | 186190 | 105105 | 136192 | 151187 | 179220 |
| BB | 144206 | 166182 | 109113 | 110172 | 167167 | 220224 |
| BB | 136156 | 166182 | 109109 | 136140 | 167179 | 188224 |
| BB | 136214 | 186186 | 93113 | 150158 | 171219 | 180226 |
| BB | 180234 | 162174 | 105121 | 158194 | 155187 | 224227 |
| BB | 152202 | 182210 | 105121 | 118158 | 147147 | 176228 |
| BB | 144170 | 194210 | 101105 | 097150 | 147155 | 184228 |
| BB | 144205 | 166198 | 101109 | 146209 | 155163 | 215228 |
| BB | 172180 | 166170 | 109113 | 138139 | 183183 | 200228 |
| BB | 156160 | 186198 | 113117 | 122206 | 179203 | 218228 |
| BB | 136140 | 174186 | 101125 | 152168 | 159163 | 232232 |
| BB | 124132 | 166170 | 101113 | 112122 | 115167 | 212232 |
| BB | 148256 | 186186 | 105125 | 131142 | 167195 | 228232 |
| BB | 172180 | 178194 | 109109 | 113130 | 171179 | 180234 |
| BB | 116136 | 158166 | 113125 | 112128 | 139155 | 204236 |
| BB | 186186 | 166194 | 105121 | 100176 | 135191 | 172236 |
| BB | 164200 | 166194 | 113117 | 118147 | 139191 | 232236 |
| BB | 156172 | 162190 | 113121 | 110129 | 187195 | 212236 |
| BB | 160206 | 174198 | 113117 | 110146 | 155207 | 232236 |
| BB | 144156 | 166170 | 109109 | 121134 | 135175 | 176244 |
| BB | 156214 | 162186 | 105117 | 136146 | 171179 | 220244 |
| BB | 120190 | 178214 | 113121 | 127133 | 171199 | 244244 |
| BB | 136144 | 190198 | 113125 | 110164 | 135135 | 182252 |
| BB | 198206 | 178182 | 109109 | 126156 | 139167 | 195252 |
| BB | 160198 | 166178 | 113117 | 122149 | 155187 | 192252 |
| BB | 140172 | 166202 | 105121 | 108172 | 131199 | 212252 |
| BB | 164170 | 198202 | 121125 | 135165 | 123151 | 252256 |
| BB | 124184 | 166202 | 109121 | 108121 | 159159 | 162256 |
| BB | 166198 | 178218 | 125149 | 136136 | 127175 | 172260 |
| BB | 160190 | 174190 | 113133 | 118134 | 179183 | 216260 |
| BB | 166166 | 182186 | 101101 | 130164 | 163167 | 236264 |
| BB | 167202 | 174190 | 101113 | 122144 | 151163 | 234268 |
| BB | 140166 | 202206 | 113113 | 105122 | 131199 | 216268 |
| BB | 136180 | 186190 | 105117 | 152152 | 131139 | 200272 |
| BB | 148152 | 190190 | 125129 | 122126 | 163171 | 244272 |
| BB | 172218 | 178202 | 109125 | 148156 | 139179 | 224272 |
| BB | 156156 | 158222 | 113117 | 113172 | 179199 | 220272 |
| BB | 190198 | 182186 | 109121 | 130202 | 183211 | 176272 |
| BB | 116156 | 166178 | 109117 | 136200 | 167171 | 172276 |
| BB | 172176 | 202210 | 117121 | 148168 | 175179 | 232276 |
| BB | 180208 | 182198 | 113117 | 122122 | 155159 | 228280 |
| BB | 176176 | 174218 | 113125 | 106142 | 127195 | 220280 |
| BB | 164168 | 174182 | 101113 | 117127 | 159195 | 228280 |
| BB | 156198 | 166186 | 117117 | 140156 | 175243 | 272280 |
| BB | 156164 | 174178 | 101113 | 110118 | 143167 | 168282 |
| BB | 144152 | 170202 | 105121 | 133138 | 143167 | 160284 |
| BB | 116168 | 174178 | 109129 | 113121 | 147175 | 216284 |
| BB | 184190 | 194194 | 105105 | 117140 | 131155 | 252286 |
| BB | 152204 | 174174 | 109117 | 113192 | 151155 | 172288 |
| BB | 180190 | 170186 | 113125 | 156240 | 127191 | 204288 |
| BB | 176264 | 166186 | 097113 | 100168 | 163235 | 224288 |
| BB | 136140 | 158186 | 113117 | 100162 | 143151 | 288292 |
| BB | 172214 | 166190 | 101113 | 212212 | 155159 | 212292 |
| BB | 164206 | 162182 | 105121 | 109144 | 147159 | 232292 |
| BB | 144164 | 166178 | 117133 | 149154 | 159179 | 168292 |
| BB | 170206 | 174182 | 101101 | 152172 | 151167 | 256296 |
| BB | 160160 | 162194 | 113129 | 109109 | 147183 | 180296 |
| BB | 144162 | 178234 | 105125 | 99122 | 191199 | 236296 |
| BB | 152160 | 162194 | 113117 | 146147 | 135147 | 192300 |
| BB | 152164 | 174194 | 101109 | 121188 | 143151 | 228300 |
| BB | 144160 | 170194 | 117125 | 122131 | 143151 | 264300 |
| BB | 139210 | 158174 | 109121 | 148177 | 155155 | 220300 |
| BB | 176198 | 194194 | 109133 | 142143 | 135179 | 180300 |
| BB | 164174 | 170186 | 117117 | 118149 | 163183 | 252300 |
| BB | 156164 | 178190 | 129145 | 130136 | 151159 | 174304 |
| BB | 140166 | 166202 | 109109 | 109130 | 179179 | 179304 |
| BB | 135160 | 158190 | 109109 | 131218 | 175187 | 236304 |
| BB | 134140 | 162186 | 105113 | 136168 | 167231 | 262304 |
| BB | 176190 | 178182 | 113117 | 168214 | 171171 | 212308 |
| BB | 156160 | 162198 | 109121 | 135184 | 151179 | 180308 |
| BB | 120148 | 190222 | 101101 | 104126 | 139191 | 172308 |
| BB | 152176 | 194198 | 113117 | 125146 | 159163 | 160312 |
| BB | 144156 | 186190 | 117125 | 118210 | 115183 | 308312 |
| BB | 151164 | 166166 | 101113 | 126165 | 187187 | 208312 |
| BB | 170170 | 186206 | 121125 | 126160 | 147151 | 162320 |
| BB | 162206 | 166182 | 101113 | 123140 | 127191 | 252328 |
| BB | 172192 | 170178 | 109109 | 101147 | 151195 | 184328 |
| BB | 168198 | 166170 | 109109 | 108113 | 139183 | 176380 |
| BB | 156184 | 174182 | 117121 | 152172 | 163179 | 308388 |
| BB | 156163 | 186194 | 101125 | 144147 | 167187 | 228388 |
| BB | 148180 | 182194 | 109117 | 122144 | 171195 | 230392 |
| BB | 176180 | 184194 | 105137 | 122172 | 139195 | 394394 |
| BB | 164190 | 162190 | 109117 | 192192 | 139227 | 160160 |
| BB | 116184 | 178194 | 113117 | 124160 | 131163 | 160164 |
| BB | 172198 | 174194 | 109129 | 113113 | 167175 | 160164 |
| BB | 158160 | 166182 | 121125 | 136172 | 131167 | 168168 |
| BB | 152198 | 162210 | 105121 | 109148 | 175239 | 160168 |
| BB | 132172 | 166170 | 105105 | 110136 | 175175 | 168176 |
| BB | 170180 | 166166 | 105121 | 156216 | 155195 | 168180 |
| BB | 152210 | 186186 | 105117 | 151151 | 127155 | 184184 |
| BB | 136172 | 182202 | 105117 | 126179 | 151159 | 164184 |
| BB | 82176 | 174206 | 105129 | 113146 | 163183 | 160188 |
| BB | 160214 | 166190 | 125125 | 148176 | 143175 | 192192 |
| BB | 168192 | 174182 | 101113 | 122192 | 175187 | 156192 |
| BB | 148164 | 162162 | 109125 | 105200 | 135187 | 184192 |
| BB | 144186 | 178198 | 101105 | 200200 | 143159 | 168200 |
| BB | 154160 | 162166 | 101109 | 122164 | 147159 | 192204 |
| BB | 206222 | 170190 | 117117 | 149168 | 131163 | 204204 |
| BB | 160182 | 170182 | 113117 | 136160 | 159187 | 204204 |
| BB | 144196 | 162206 | 109117 | 130147 | 147211 | 160204 |
| BB | 112194 | 178178 | 117125 | 126156 | 131187 | 192206 |
| BB | 154168 | 166178 | 113113 | 121142 | 163163 | 204208 |
| BB | 160168 | 174206 | 109125 | 122134 | 147179 | 204208 |
| BB | 148202 | 206210 | 101105 | 136180 | 139159 | 180210 |
| BB | 148156 | 166190 | 117117 | 113135 | 143155 | 192212 |
| BB | 152168 | 186238 | 105109 | 156160 | 147159 | 192212 |
| BB | 116116 | 170194 | 105145 | 125226 | 163163 | 212212 |
| BB | 152174 | 166202 | 109121 | 146146 | 163167 | 212212 |
| BB | 148172 | 182186 | 113113 | 126160 | 155171 | 212212 |
| BB | 148168 | 186210 | 117121 | 126162 | 123179 | 176212 |
| BB | 172172 | 174182 | 109113 | 101109 | 179183 | 188212 |
| BB | 172172 | 162206 | 117133 | 114152 | 147163 | 164216 |
| BB | 152168 | 174202 | 105105 | 122130 | 159163 | 172216 |
| BB | 152216 | 158198 | 105113 | 134138 | 139167 | 180216 |
| BB | 156206 | 158178 | 105131 | 135164 | 135167 | 188216 |
| BB | 176210 | 166202 | 105113 | 144184 | 147167 | 216216 |
| BB | 132202 | 174190 | 109129 | 126200 | 163175 | 196216 |
| BB | 156224 | 174182 | 109117 | 98118 | 159175 | 212216 |
| BB | 186206 | 186198 | 109117 | 106160 | 163179 | 216216 |
| BB | 156198 | 158186 | 121129 | 166234 | 175183 | 188216 |
| BB | 152160 | 182182 | 101113 | 112134 | 179183 | 216216 |
| BB | 136176 | 166210 | 097117 | 142175 | 151155 | 192217 |
| BB | 152178 | 186194 | 101109 | 128132 | 187199 | 164218 |
| BB | 140152 | 166178 | 101129 | 142156 | 155159 | 204220 |
| BB | 148176 | 166206 | 121125 | 112118 | 147163 | 220220 |
| BB | 148160 | 178190 | 121133 | 113155 | 167171 | 172220 |
| BB | 136148 | 162190 | 105113 | 118152 | 167175 | 212220 |
| BB | 156156 | 166206 | 113117 | 122126 | 163203 | 221221 |
| BB | 156164 | 170178 | 121121 | 113170 | 139171 | 216222 |
| BB | 148148 | 202202 | 117129 | 122122 | 151151 | 180224 |
| BB | 144178 | 178190 | 109121 | 152152 | 143155 | 224224 |
| BB | 140164 | 174210 | 117137 | 106142 | 143167 | 180224 |
| BB | 152160 | 190194 | 101109 | 156176 | 139175 | 212224 |
| BB | 160168 | 174186 | 117117 | 109148 | 171183 | 216224 |
| BB | 144180 | 174194 | 113125 | 130155 | 159183 | 220224 |
| BB | 136160 | 178198 | 105109 | 138152 | 143187 | 224224 |
| BB | 168190 | 178194 | 113117 | 102164 | 135207 | 172224 |
| BB | 116156 | 178182 | 113113 | 116117 | 123183 | 172226 |
| BB | 164214 | 162174 | 109113 | 142156 | 159187 | 219226 |
| BB | 190228 | 190190 | 109113 | 109123 | 135143 | 216228 |
| BB | 148148 | 162194 | 105117 | 113129 | 155159 | 184228 |
| BB | 148164 | 170190 | 109121 | 117117 | 175191 | 216228 |
| BB | 156220 | 162186 | 109117 | 105142 | 163191 | 228228 |
| BB | 148160 | 162178 | 125137 | 128156 | 151199 | 228228 |
| BB | 204206 | 178186 | 117125 | 114144 | 143203 | 224228 |
| BB | 164244 | 166218 | 117129 | 146180 | 139223 | 208228 |
| BB | 206210 | 166190 | 105125 | 093138 | 155159 | 200229 |
| BB | 160172 | 174182 | 109113 | 138138 | 135171 | 200229 |
| BB | 171188 | 174194 | 109121 | 160184 | 151159 | 156230 |
| BB | 168202 | 170198 | 97109 | 118160 | 155167 | 202230 |
| BB | 116156 | 170218 | 109109 | 136174 | 135175 | 212230 |
| BB | 132156 | 174186 | 109121 | 121188 | 151175 | 222230 |
| BB | 164182 | 162182 | 105109 | 168184 | 143187 | 172230 |
| BB | 116206 | 170186 | 117125 | 106117 | 135147 | 172232 |
| BB | 148210 | 170198 | 105125 | 138164 | 143163 | 160232 |
| BB | 148154 | 198226 | 105117 | 113152 | 163167 | 224232 |
| BB | 148180 | 178182 | 113121 | 118156 | 179183 | 224232 |
| BB | 176210 | 166198 | 117125 | 144180 | 187187 | 212232 |
| BB | 148158 | 166202 | 97101 | 113178 | 171195 | 232232 |
| BB | 160198 | 166210 | 97117 | 142156 | 167223 | 232232 |
| BB | 156198 | 194214 | 105129 | 116131 | 159159 | 220234 |
| BB | 156180 | 162194 | 109129 | 122122 | 135163 | 196234 |
| BB | 170204 | 186218 | 125131 | 145212 | 147167 | 188234 |
| BB | 166166 | 186190 | 113117 | 118180 | 139171 | 234234 |
| BB | 136202 | 162234 | 109117 | 144144 | 175231 | 230234 |
| BB | 116152 | 178214 | 109125 | 139168 | 135159 | 232236 |
| BB | 116116 | 166202 | 117129 | 138176 | 159163 | 180236 |
| BB | 172248 | 194198 | 109125 | 121180 | 135175 | 172236 |
| BB | 178218 | 158162 | 113121 | 140180 | 123195 | 184236 |
| BB | 158206 | 174194 | 117121 | 121136 | 163163 | 230238 |
| BB | 120164 | 178198 | 121129 | 136152 | 167167 | 176238 |
| BB | 116160 | 162170 | 105117 | 113138 | 155175 | 164238 |
| BB | 144164 | 190194 | 117121 | 105152 | 175199 | 238238 |
| BB | 150150 | 202214 | 117117 | 108168 | 171235 | 225238 |
| BB | 140156 | 174174 | 109125 | 132198 | 163167 | 192240 |
| BB | 160168 | 158186 | 101105 | 126146 | 155167 | 216240 |
| BB | 172186 | 194202 | 113117 | 126163 | 167191 | 180240 |
| BB | 128156 | 182186 | 117121 | 113130 | 163239 | 236240 |
| BB | 124190 | 198198 | 121125 | 148172 | 159179 | 202241 |
| BB | 160218 | 158198 | 109113 | 156232 | 151175 | 242242 |
| BB | 140160 | 158178 | 105113 | 113180 | 147187 | 160242 |
| BB | 148204 | 162190 | 109121 | 132152 | 171179 | 236244 |
| BB | 148216 | 178182 | 109109 | 109162 | 151199 | 184244 |
| BB | 160160 | 186238 | 105113 | 143198 | 139167 | 160246 |
| BB | 188190 | 170186 | 109109 | 101264 | 163179 | 200248 |
| BB | 152168 | 178202 | 121125 | 136170 | 155179 | 212248 |
| BB | 168172 | 182194 | 117129 | 118140 | 143183 | 188250 |
| BB | 160202 | 166174 | 109129 | 113168 | 163171 | 180252 |
| BB | 152152 | 162174 | 113121 | 113160 | 139163 | 220254 |
| BB | 144168 | 174182 | 109109 | 130136 | 159211 | 214254 |
| BB | 152194 | 166174 | 105133 | 144148 | 175175 | 229256 |
| BB | 156316 | 178186 | 101131 | 164175 | 151159 | 216260 |
| BB | 116144 | 174174 | 121121 | 121172 | 139159 | 250262 |
| BB | 190206 | 174210 | 101109 | 148160 | 155155 | 224264 |
| BB | 160168 | 198206 | 101117 | 118176 | 135167 | 228264 |
| BB | 148180 | 178202 | 109121 | 164182 | 123163 | 184266 |
| BB | 202206 | 166174 | 105113 | 125144 | 147183 | 230266 |
| BB | 148210 | 186198 | 105105 | 122122 | 147151 | 176268 |
| BB | 164222 | 190194 | 101121 | 151174 | 167171 | 212268 |
| BB | 136152 | 178198 | 109125 | 109122 | 167175 | 264268 |
| BB | 160220 | 186190 | 113113 | 146176 | 163179 | 234268 |
| BB | 132156 | 178198 | 113129 | 105117 | 163199 | 208268 |
| BB | 116182 | 162210 | 105121 | 113126 | 119143 | 197272 |
| BB | 148156 | 170190 | 101109 | 143172 | 147155 | 234272 |
| BB | 116144 | 202202 | 97113 | 184188 | 131155 | 246272 |
| BB | 160160 | 202202 | 101125 | 104113 | 147171 | 192272 |
| BB | 172180 | 166170 | 109133 | 113143 | 135191 | 212272 |
| BB | 136198 | 178206 | 113117 | 134170 | 191199 | 240272 |
| BB | 156160 | 178190 | 113117 | 109152 | 131151 | 224273 |
| BB | 152156 | 178202 | 109113 | 180180 | 139147 | 226276 |
| BB | 152164 | 166194 | 105109 | 126164 | 135159 | 212276 |
| BB | 156194 | 182194 | 101109 | 130186 | 163167 | 212276 |
| BB | 140148 | 194198 | 113113 | 102126 | 159171 | 180276 |
| BB | 164172 | 162162 | 105133 | 108134 | 159175 | 212276 |
| BB | 128156 | 194206 | 117121 | 105126 | 159179 | 212276 |
| BB | 168184 | 178186 | 109125 | 130144 | 163179 | 246276 |
| BB | 152180 | 166170 | 101117 | 144200 | 151195 | 168276 |
| BB | 148180 | 170206 | 085109 | 138160 | 127207 | 212276 |
| BB | 140144 | 170186 | 101105 | 104130 | 159159 | 176280 |
| BB | 156172 | 182198 | 113113 | 148168 | 159159 | 176280 |
| BB | 168230 | 170174 | 105109 | 134160 | 163167 | 250280 |
| BB | 156222 | 190198 | 105121 | 156178 | 155175 | 224280 |
| BB | 160176 | 174202 | 117129 | 143152 | 159191 | 276280 |
| BB | 116152 | 190198 | 105121 | 130168 | 163227 | 188280 |
| BB | 164171 | 190202 | 101137 | 178180 | 147159 | 164284 |
| BB | 176188 | 178182 | 105125 | 172172 | 155163 | 224284 |
| BB | 132168 | 174186 | 105113 | 98196 | 143167 | 238284 |
| BB | 176198 | 174214 | 113117 | 160164 | 139171 | 224284 |
| BB | 116160 | 166222 | 113121 | 138150 | 143175 | 280284 |
| BB | 132176 | 166182 | 101121 | 105113 | 187199 | 230284 |
| BB | 82148 | 170174 | 109109 | 156156 | 143203 | 176284 |
| BB | 178220 | 166186 | 105113 | 117125 | 151171 | 260288 |
| BB | 116186 | 182182 | 105109 | 100126 | 147175 | 212288 |
| BB | 172176 | 178190 | 113133 | 109133 | 147175 | 256288 |
| BB | 152180 | 174190 | 121121 | 121121 | 159179 | 216288 |
| BB | 172190 | 174194 | 109133 | 126212 | 135179 | 276288 |
| BB | 147198 | 170186 | 101117 | 144152 | 147183 | 256288 |
| BB | 144156 | 178206 | 105105 | 140152 | 163183 | 260288 |
| BB | 152168 | 198210 | 121129 | 102148 | 175211 | 180288 |
| BB | 120192 | 166206 | 109125 | 148169 | 155159 | 290290 |
| BB | 160198 | 182198 | 093113 | 116154 | 131163 | 192292 |
| BB | 116200 | 182214 | 105117 | 152194 | 151187 | 256292 |
| BB | 152214 | 210210 | 109109 | 113165 | 159215 | 200292 |
| BB | 140168 | 174182 | 113121 | 138150 | 123131 | 220296 |
| BB | 144192 | 166174 | 109113 | 152168 | 163163 | 280296 |
| BB | 156166 | 174186 | 109113 | 148156 | 167167 | 168296 |
| BB | 152192 | 190194 | 121121 | 112162 | 155167 | 260296 |
| BB | 156164 | 166178 | 105121 | 113126 | 131167 | 296296 |
| BB | 160168 | 162186 | 105109 | 109152 | 175175 | 192296 |
| BB | 152152 | 166194 | 101117 | 109113 | 163179 | 296296 |
| BB | 140196 | 166174 | 105113 | 117121 | 159183 | 184296 |
| BB | 148202 | 182198 | 113145 | 112156 | 151187 | 160296 |
| BB | 156156 | 162170 | 117129 | 110152 | 163195 | 276296 |
| BB | 160232 | 162186 | 109113 | 155163 | 159207 | 252296 |
| BB | 168182 | 190194 | 101121 | 143170 | 187223 | 224296 |
| BB | 168218 | 190218 | 117121 | 113130 | 227227 | 212296 |
| BB | 128206 | 166194 | 113113 | 105140 | 151155 | 204300 |
| BB | 148148 | 182198 | 109125 | 122126 | 167171 | 188300 |
| BB | 144180 | 162170 | 109121 | 114192 | 163175 | 196300 |
| BB | 160186 | 170186 | 101113 | 148159 | 155183 | 284300 |
| BB | 168202 | 174194 | 125133 | 117146 | 155187 | 228300 |
| BB | 172188 | 182186 | 117117 | 101156 | 163187 | 276300 |
| BB | 116188 | 182190 | 109113 | 126140 | 163191 | 296300 |
| BB | 136182 | 178182 | 109121 | 140151 | 167199 | 296300 |
| BB | 148176 | 170184 | 109113 | 160168 | 171207 | 276300 |
| BB | 116160 | 178198 | 109113 | 122196 | 139211 | 264300 |
| BB | 156216 | 182186 | 109117 | 117146 | 139155 | 260304 |
| BB | 168178 | 162178 | 109117 | 149164 | 143163 | 248304 |
| BB | 180180 | 174202 | 097113 | 152156 | 123167 | 200304 |
| BB | 139206 | 210226 | 125129 | 109188 | 167171 | 168304 |
| BB | 155156 | 174222 | 113133 | 175156 | 147187 | 200304 |
| BB | 140144 | 170182 | 101117 | 126155 | 207219 | 260304 |
| BB | 176224 | 182190 | 101113 | 125164 | 127147 | 230308 |
| BB | 170172 | 174182 | 113121 | 144152 | 135163 | 304308 |
| BB | 148148 | 166174 | 101121 | 112122 | 155171 | 296308 |
| BB | 152160 | 170206 | 121125 | 134152 | 167175 | 164308 |
| BB | 148178 | 190210 | 121121 | 140164 | 159175 | 212308 |
| BB | 140168 | 186186 | 097117 | 162200 | 163183 | 256308 |
| BB | 160168 | 174186 | 097105 | 130152 | 143187 | 216308 |
| BB | 166176 | 190194 | 109141 | 114134 | 183223 | 296308 |
| BB | 168206 | 210218 | 105109 | 105160 | 135171 | 240312 |
| BB | 152196 | 174174 | 101109 | 113188 | 135171 | 242312 |
| BB | 166184 | 198210 | 105105 | 139180 | 155175 | 240312 |
| BB | 148164 | 186198 | 117129 | 152204 | 147195 | 172312 |
| BB | 156198 | 162186 | 105105 | 184208 | 167211 | 300312 |
| BB | 148152 | 182186 | 109117 | 130152 | 143175 | 212316 |
| BB | 140164 | 174178 | 105109 | 130156 | 123123 | 220320 |
| BB | 140140 | 170190 | 105133 | 105148 | 159163 | 320320 |
| BB | 160176 | 186190 | 113117 | 110147 | 179195 | 224320 |
| BB | 152202 | 170210 | 117129 | 136144 | 159195 | 276320 |
| BB | 152218 | 190202 | 109109 | 136148 | 147147 | 226324 |
| BB | 136156 | 158206 | 105121 | 126144 | 143159 | 214324 |
| BB | 156180 | 178182 | 105113 | 111113 | 163167 | 184324 |
| BB | 164192 | 158190 | 129137 | 156171 | 123187 | 288324 |
| BB | 148160 | 162202 | 117117 | 126134 | 171219 | 180328 |
| BB | 128152 | 162190 | 101113 | 118192 | 139175 | 320332 |
| BB | 116180 | 166170 | 105109 | 117160 | 151199 | 280340 |
| BB | 160170 | 182202 | 113117 | 156156 | 155179 | 246342 |
| BB | 148170 | 162178 | 129133 | 122138 | 159167 | 230348 |
| BB | 160168 | 186198 | 121125 | 116116 | 179215 | 196352 |
| BB | 132176 | 170198 | 105109 | 113130 | 147155 | 380384 |
| BB | 212212 | 174198 | 109125 | 144156 | 151159 | 212384 |
| BB | 132148 | 162202 | 105121 | 113179 | 163171 | 284384 |
| BB | 164190 | 170194 | 105109 | 122239 | 139191 | 236384 |
| BB | 196238 | 158186 | 105129 | 113126 | 147183 | 216388 |
| BB | 186202 | 158198 | 109113 | 126126 | 155219 | 308392 |
| BB | 156222 | 166182 | 117129 | 172184 | 143147 | 200400 |
| BB | 164242 | 170194 | 105117 | 155166 | 155175 | 160160 |
| BB | 132152 | 170186 | 121129 | 111175 | 127159 | 164164 |
| BB | 136186 | 170182 | 101105 | 099131 | 163167 | 174174 |
| BB | 216228 | 186186 | 105109 | 115196 | 143171 | 184184 |
| BB | 160188 | 166198 | 109109 | 122168 | 135191 | 200200 |
| BB | 200218 | 178198 | 129129 | 146151 | 163167 | 208208 |
| BB | 116116 | 182210 | 105125 | 110131 | 139171 | 160208 |
| BB | 116152 | 166174 | 117117 | 143151 | 147171 | 188212 |
| BB | 160174 | 162178 | 113121 | 115160 | 179183 | 208212 |
| BB | 164216 | 178190 | 105125 | 150163 | 171215 | 200212 |
| BB | 164206 | 174190 | 109117 | 127163 | 175179 | 206215 |
| BB | 148156 | 218218 | 113121 | 131180 | 131155 | 212216 |
| BB | 192208 | 166186 | 101105 | 163163 | 155183 | 158216 |
| BB | 148160 | 178186 | 101125 | 122145 | 155191 | 186216 |
| BB | 164188 | 162174 | 97137 | 155200 | 155171 | 208220 |
| BB | 186188 | 154158 | 105121 | 123135 | 175179 | 192220 |
| BB | 148212 | 174186 | 105133 | 123198 | 191211 | 220220 |
| BB | 150164 | 174194 | 117121 | 131214 | 179191 | 188222 |
| BB | 140140 | 162166 | 113125 | 135159 | 139151 | 192224 |
| BB | 160166 | 194198 | 109137 | 130145 | 163167 | 224224 |
| BB | 116152 | 214222 | 105109 | 115164 | 115151 | 176228 |
| BB | 136184 | 182186 | 121125 | 115123 | 123175 | 216228 |
| BB | 144152 | 174178 | 109125 | 141145 | 159195 | 230230 |
| BB | 196200 | 162178 | 113121 | 107194 | 139147 | 160238 |
| BB | 120164 | 166178 | 113121 | 105110 | 139151 | 228244 |
| BB | 156196 | 158186 | 109113 | 115123 | 159191 | 196250 |
| BB | 144148 | 166178 | 105109 | 115118 | 175183 | 172252 |
| BB | 132164 | 162198 | 113133 | 176184 | 167207 | 196252 |
| BB | 148168 | 182202 | 101109 | 136147 | 187187 | 220254 |
| BB | 156163 | 186186 | 105133 | 119119 | 139159 | 164256 |
| BB | 216220 | 182198 | 109109 | 127147 | 167179 | 252256 |
| BB | 140168 | 158162 | 109109 | 147147 | 131151 | 200274 |
| BB | 156186 | 178190 | 113121 | 126174 | 143179 | 228274 |
| BB | 156194 | 162186 | 101101 | 131176 | 139151 | 252276 |
| BB | 164174 | 178202 | 105113 | 153207 | 163207 | 276276 |
| BB | 148186 | 174226 | 101109 | 123128 | 147159 | 252280 |
| BB | 148212 | 186210 | 105121 | 114134 | 143171 | 246282 |
| BB | 176180 | 166166 | 109117 | 139151 | 179195 | 186282 |
| BB | 172180 | 170210 | 101105 | 132183 | 147159 | 228284 |
| BB | 148204 | 186190 | 097117 | 134151 | 135143 | 264292 |
| BB | 136136 | 174194 | 105133 | 102127 | 159167 | 212292 |
| BB | 160160 | 162178 | 113117 | 111143 | 171175 | 292292 |
| BB | 148156 | 162178 | 113113 | 123166 | 167183 | 216292 |
| BB | 164172 | 202202 | 109121 | 143182 | 187207 | 158292 |
| BB | 140193 | 170198 | 105125 | 118130 | 147151 | 212296 |
| BB | 148156 | 162178 | 105109 | 126139 | 155175 | 166296 |
| BB | 152176 | 162186 | 109121 | 127130 | 167167 | 215300 |
| BB | 172184 | 158162 | 101105 | 111115 | 163171 | 278300 |
| BB | 116140 | 166194 | 97109 | 118136 | 163179 | 292300 |
| BB | 156178 | 186186 | 93109 | 131167 | 127183 | 224300 |
| BB | 172172 | 174218 | 117117 | 139175 | 179183 | 240300 |
| BB | 148160 | 166202 | 105105 | 139151 | 171187 | 192300 |
| BB | 152164 | 170182 | 113121 | 114132 | 155195 | 170300 |
| BB | 140144 | 170194 | 101105 | 107123 | 143195 | 212300 |
| BB | 160176 | 194194 | 109121 | 194194 | 159199 | 206300 |
| BB | 132192 | 206206 | 105117 | 139164 | 115163 | 216304 |
| BB | 168180 | 166186 | 109113 | 113131 | 147171 | 228304 |
| BB | 148168 | 210214 | 113125 | 119151 | 167183 | 214304 |
| BB | 160208 | 178202 | 105109 | 126214 | 163243 | 188304 |
| BB | 116197 | 186190 | 117149 | 147155 | 151155 | 192308 |
| BB | 156176 | 170182 | 113125 | 119167 | 163167 | 200308 |
| BB | 160204 | 174178 | 109117 | 109159 | 159171 | 254312 |
| BB | 148156 | 194194 | 113121 | 106151 | 151175 | 284312 |
| BB | 148164 | 170178 | 113125 | 102143 | 143171 | 198316 |
| BB | 152202 | 178190 | 105109 | 142152 | 175183 | 170316 |
| BB | 152164 | 170202 | 109117 | 147194 | 207207 | 230316 |
| BB | 148200 | 194218 | 113117 | 136147 | 147159 | 170324 |
| BB | 172178 | 174198 | 109121 | 110114 | 127215 | 182324 |
| BB | 136152 | 174210 | 101125 | 147194 | 159159 | 238336 |
| BB | 140172 | 166194 | 117125 | 135165 | 135135 | 296358 |
| BB | 176200 | 178186 | 133133 | 148188 | 175199 | 219358 |
| BB | 138181 | 182190 | 101117 | 107115 | 155187 | 168394 |
| BB | 140182 | 166186 | 105125 | 107151 | 171207 | 174178 |
| BB | 148160 | 166194 | 101121 | 127206 | 179187 | 192192 |
| BB | 152189 | 162186 | 105113 | 139171 | 123147 | 188204 |
| BB | 116148 | 174202 | 113113 | 107130 | 151199 | 198206 |
| BB | 182224 | 158182 | 101113 | 131244 | 155179 | 160216 |
| BB | 163236 | 194202 | 105117 | 159167 | 155199 | 188220 |
| BB | 190234 | 170198 | 125125 | 142142 | 147151 | 180222 |
| BB | 140244 | 186202 | 105113 | 121142 | 135187 | 214226 |
| BB | 144182 | 166206 | 113121 | 104117 | 171171 | 198227 |
| BB | 164196 | 158162 | 101105 | 115143 | 123151 | 206228 |
| BB | 156204 | 174194 | 105113 | 114166 | 151171 | 213228 |
| BB | 152164 | 166198 | 105117 | 130147 | 171191 | 178228 |
| BB | 160179 | 174186 | 105129 | 130159 | 147159 | 224232 |
| BB | 148152 | 166178 | 109121 | 134173 | 143171 | 228232 |
| BB | 116156 | 174210 | 117117 | 130138 | 167191 | 228232 |
| BB | 156206 | 178182 | 109137 | 109127 | 195207 | 219234 |
| BB | 181187 | 178218 | 117121 | 140147 | 155167 | 188236 |
| BB | 136168 | 194194 | 113121 | 130143 | 135167 | 192236 |
| BB | 148148 | 186190 | 97125 | 105150 | 131151 | 180242 |
| BB | 148200 | 162162 | 109109 | 123123 | 167175 | 180246 |
| BB | 116204 | 186190 | 109125 | 126173 | 147183 | 194248 |
| BB | 116164 | 178194 | 113117 | 166234 | 171175 | 242254 |
| BB | 84162 | 190202 | 109121 | 167232 | 131167 | 216258 |
| BB | 148152 | 170206 | 105121 | 113155 | 171175 | 223262 |
| BB | 148168 | 170182 | 113117 | 127196 | 171195 | 210262 |
| BB | 156156 | 166178 | 113137 | 105135 | 167203 | 190262 |
| BB | 155200 | 178186 | 93113 | 130177 | 155183 | 232270 |
| BB | 116136 | 166166 | 121125 | 115123 | 131167 | 230276 |
| BB | 160180 | 190218 | 113113 | 131172 | 159175 | 192276 |
| BB | 152164 | 162170 | 105133 | 139150 | 151167 | 228280 |
| BB | 140160 | 186194 | 109109 | 131139 | 171179 | 240284 |
| BB | 144148 | 162162 | 105129 | 126142 | 131155 | 288288 |
| BB | 160216 | 178190 | 105121 | 155171 | 159175 | 284288 |
| BB | 144190 | 162162 | 109121 | 109139 | 155179 | 284288 |
| BB | 176176 | 170174 | 109121 | 113118 | 135183 | 248292 |
| BB | 180196 | 174194 | 113121 | 135142 | 143187 | 296296 |
| BB | 148163 | 162238 | 109121 | 118126 | 147171 | 220300 |
| BB | 147168 | 162170 | 113121 | 136159 | 159171 | 264300 |
| BB | 184224 | 162178 | 117133 | 110131 | 135175 | 288300 |
| BB | 148216 | 170198 | 113121 | 114138 | 131151 | 212304 |
| BB | 120168 | 198226 | 101109 | 144144 | 143151 | 258304 |
| BB | 168192 | 190202 | 105105 | 131139 | 143167 | 272304 |
| BB | 140168 | 170182 | 101101 | 154170 | 175191 | 232304 |
| BB | 160216 | 174202 | 121125 | 127148 | 143163 | 172308 |
| BB | 156172 | 166178 | 109113 | 123131 | 159203 | 216312 |
| BB | 198204 | 162190 | 105105 | 142218 | 175215 | 176316 |
| BB | 160176 | 162194 | 117121 | 131150 | 163183 | 296332 |
| BB | 156176 | 178226 | 109141 | 143150 | 139183 | 217388 |
| BB | 140140 | 170190 | 97109 | 114118 | 155187 | 196196 |
| BB | 136184 | 190202 | 105105 | 98111 | 167195 | 180196 |
| BB | 148148 | 174194 | 109121 | 115142 | 155215 | 192204 |
| BB | 152192 | 166170 | 121121 | 130188 | 143175 | 184212 |
| BB | 168188 | 166194 | 105117 | 126159 | 179231 | 168212 |
| BB | 136156 | 162170 | 101117 | 123135 | 163171 | 184214 |
| BB | 180180 | 182190 | 109129 | 119141 | 163175 | 212216 |
| BB | 144188 | 190198 | 101133 | 108131 | 167211 | 168216 |
| BB | 156174 | 182194 | 105109 | 115127 | 159167 | 206218 |
| BB | 182220 | 162186 | 97105 | 112131 | 183203 | 214218 |
| BB | 116170 | 182198 | 125125 | 155156 | 155167 | 180222 |
| BB | 198220 | 186210 | 109113 | 141163 | 167183 | 222222 |
| BB | 148240 | 170170 | 97105 | 138165 | 159219 | 188224 |
| BB | 116216 | 190234 | 101105 | 123138 | 183219 | 204224 |
| BB | 128156 | 170190 | 113129 | 122143 | 127147 | 222226 |
| BB | 188196 | 186206 | 101125 | 122152 | 151203 | 210226 |
| BB | 152234 | 178202 | 97121 | 184204 | 167175 | 160227 |
| BB | 116204 | 178194 | 117129 | 123206 | 115191 | 168227 |
| BB | 144156 | 174182 | 129129 | 115155 | 163175 | 196228 |
| BB | 152254 | 182186 | 105109 | 110191 | 143223 | 188228 |
| BB | 162168 | 170178 | 105109 | 107111 | 139139 | 224230 |
| BB | 116170 | 166202 | 105145 | 115178 | 143171 | 230230 |
| BB | 160160 | 170198 | 113117 | 111111 | 171199 | 196234 |
| BB | 156176 | 166186 | 109113 | 93131 | 143199 | 206234 |
| BB | 208216 | 166194 | 101105 | 137163 | 143191 | 236236 |
| BB | 172216 | 166190 | 101109 | 115159 | 171207 | 208236 |
| BB | 156200 | 190194 | 109109 | 133151 | 155175 | 220237 |
| BB | 134148 | 182210 | 109117 | 139194 | 139163 | 210252 |
| BB | 130212 | 162198 | 105113 | 142154 | 115171 | 180260 |
| BB | 148212 | 182182 | 113117 | 118130 | 131171 | 196264 |
| BB | 164180 | 166186 | 117125 | 114151 | 155183 | 226264 |
| BB | 148204 | 174186 | 121125 | 113159 | 159175 | 212272 |
| BB | 148164 | 178186 | 117121 | 111147 | 171175 | 226272 |
| BB | 148204 | 186198 | 109121 | 133151 | 159171 | 212276 |
| BB | 152164 | 174198 | 117161 | 163172 | 167203 | 218276 |
| BB | 184200 | 166174 | 117117 | 135145 | 171207 | 222276 |
| BB | 157208 | 194198 | 117121 | 135135 | 131147 | 214280 |
| BB | 144168 | 178214 | 101113 | 139143 | 139159 | 226280 |
| BB | 216246 | 162182 | 105137 | 106133 | 123163 | 256280 |
| BB | 156188 | 162174 | 105113 | 97154 | 175179 | 280284 |
| BB | 156176 | 182206 | 105117 | 103221 | 151183 | 252284 |
| BB | 156210 | 186190 | 105133 | 140142 | 155167 | 240288 |
| BB | 160210 | 166178 | 133141 | 119159 | 167171 | 225288 |
| BB | 172228 | 166194 | 133137 | 151204 | 139143 | 220292 |
| BB | 164194 | 170178 | 121125 | 110186 | 159167 | 284292 |
| BB | 156160 | 186198 | 121125 | 107151 | 143167 | 288292 |
| BB | 160160 | 170186 | 117117 | 149166 | 163179 | 248292 |
| BB | 148168 | 170190 | 109109 | 110114 | 195199 | 230292 |
| BB | 160234 | 190206 | 101109 | 105115 | 155163 | 180296 |
| BB | 136140 | 174186 | 97129 | 111169 | 167167 | 188296 |
| BB | 156164 | 174194 | 101121 | 129139 | 155167 | 292296 |
| BB | 116180 | 178182 | 105113 | 112115 | 139179 | 218296 |
| BB | 140204 | 162198 | 117121 | 111139 | 139179 | 226296 |
| BB | 160176 | 182202 | 113121 | 143154 | 167187 | 212296 |
| BB | 152160 | 174190 | 109113 | 102143 | 175191 | 220296 |
| BB | 178218 | 174190 | 109121 | 103126 | 167175 | 272300 |
| BB | 136230 | 158194 | 109129 | 111158 | 151183 | 284300 |
| BB | 144172 | 206206 | 109121 | 148155 | 151183 | 296300 |
| BB | 208216 | 198210 | 109113 | 151174 | 155203 | 208300 |
| BB | 144148 | 158186 | 117129 | 114162 | 163219 | 260300 |
| BB | 152208 | 158186 | 105125 | 138139 | 139151 | 276304 |
| BB | 148168 | 162226 | 113121 | 122143 | 139163 | 188304 |
| BB | 116172 | 186202 | 117121 | 111137 | 163175 | 220304 |
| BB | 166220 | 174190 | 121129 | 151232 | 151175 | 232304 |
| BB | 168232 | 186214 | 117121 | 118147 | 123187 | 256312 |
| BB | 156262 | 190202 | 101109 | 119119 | 139155 | 268316 |
| BB | 120156 | 202214 | 101109 | 114126 | 159179 | 316320 |
| BB | 156160 | 166182 | 105125 | 107141 | 151183 | 240320 |
| BB | 116151 | 170194 | 117121 | 102102 | 159159 | 246324 |
| BB | 164164 | 170178 | 129145 | 150166 | 195195 | 244324 |
| BB | 220220 | 162186 | 105133 | 107171 | 159175 | 288332 |
| BB | 120200 | 162166 | 105125 | 122152 | 163175 | 280387 |
| 2005BB | 144164 | 174222 | 121141 | 118208 | 151155 | 184184 |
| 2005BB | 156160 | 166190 | 93113 | 139147 | 163167 | 160200 |
| 2005BB | 184204 | 174194 | 105117 | 102124 | 147159 | 192216 |
| 2005BB | 152180 | 166170 | 109121 | 117146 | 131171 | 118220 |
| 2005BB | 168172 | 186198 | 105117 | 121160 | 159175 | 180220 |
| 2005BB | 136232 | 186198 | 105117 | 152194 | 163191 | 184222 |
| 2005BB | 140160 | 178194 | 121125 | 142149 | 135159 | 228228 |
| 2005BB | 154208 | 158202 | 121121 | 130172 | 175187 | 170228 |
| 2005BB | 196196 | 182186 | 109113 | 112180 | 175203 | 180228 |
| 2005BB | 168190 | 186202 | 109129 | 170180 | 175175 | 212236 |
| 2005BB | 168196 | 158238 | 105109 | 112159 | 123159 | 192240 |
| 2005BB | 80168 | 170194 | 121125 | 139156 | 131179 | 224240 |
| 2005BB | 136148 | 170210 | 105129 | 120159 | 151175 | 184244 |
| 2005BB | 136200 | 178194 | 113125 | 138155 | 171183 | 244244 |
| 2005BB | 136180 | 186198 | 113125 | 127146 | 183143 | 227252 |
| 2005BB | 156156 | 178198 | 105125 | 156180 | 155179 | 216256 |
| 2005BB | 114232 | 162182 | 109121 | 134184 | 167187 | 214256 |
| 2005BB | 216220 | 194202 | 109113 | 112168 | 155163 | 208260 |
| 2005BB | 114208 | 170174 | 105125 | 116167 | 135155 | 228266 |
| 2005BB | 168192 | 166166 | 105117 | 124124 | 135139 | 228268 |
| 2005BB | 184228 | 170202 | 109109 | 126129 | 155163 | 256268 |
| 2005BB | 156226 | 178194 | 109125 | 134142 | 131175 | 184272 |
| 2005BB | 136170 | 182218 | 109109 | 93168 | 179199 | 192272 |
| 2005BB | 114180 | 174226 | 109109 | 125137 | 151167 | 216280 |
| 2005BB | 152222 | 174202 | 117133 | 159162 | 143183 | 228280 |
| 2005BB | 136156 | 182206 | 97117 | 155178 | 155167 | 238284 |
| 2005BB | 144160 | 166178 | 117121 | 114133 | 171179 | 208284 |
| 2005BB | 148168 | 174174 | 113129 | 131168 | 151187 | 288288 |
| 2005BB | 172216 | 166170 | 109109 | 117160 | 159179 | 220292 |
| 2005BB | 144188 | 166198 | 109113 | 133176 | 147203 | 192292 |
| 2005BB | 148204 | 166174 | 105105 | 124176 | 155175 | 208300 |
| 2005BB | 114136 | 170170 | 101113 | 104116 | 151187 | 300300 |
| 2005BB | 156220 | 170198 | 121141 | 121175 | 183215 | 174300 |
| 2005BB | 176240 | 198218 | 105133 | 108138 | 151235 | 196300 |
| 2005BB | 148198 | 182186 | 109113 | 117140 | 139151 | 304304 |
| 2005BB | 164208 | 162210 | 109121 | 129175 | 162227 | 272304 |
| 2005BB | 148148 | 162194 | 101101 | 125166 | 167171 | 208308 |
| 2005BB | 116132 | 174194 | 109113 | 114134 | 135171 | 228308 |
| 2005BB | 136160 | 158170 | 109117 | 163184 | 147155 | 262312 |
| 2005BB | 114196 | 178218 | 113117 | 118151 | 167179 | 232312 |
| 2005BB | 172222 | 198202 | 113117 | 124130 | 139167 | 284328 |
| 2005BB | 156184 | 174214 | 125145 | 125182 | 139163 | 184332 |
| 2005BB | 156178 | 174222 | 121129 | 147155 | 147159 | 272336 |
| 2005BB | 190212 | 166182 | 97109 | 138138 | 167187 | 300354 |
| 2005BB | 82176 | 174174 | 109141 | 155158 | 155219 | 288370 |
| 2005BB | 156228 | 166174 | 105117 | 129175 | 159159 | 320390 |
| 2005BB | 148152 | 194194 | 109117 | 112146 | 167183 | 394394 |
| BB61 | 164166 | 162198 | 109113 | 121192 | 171187 | 160160 |
| BB61 | 116156 | 174194 | 97129 | 122171 | 159187 | 160188 |
| BB61 | 148163 | 170202 | 105109 | 136168 | 163163 | 180200 |
| BB61 | 116136 | 186190 | 101105 | 122176 | 151155 | 204208 |
| BB61 | 156160 | 162214 | 101101 | 102160 | 143155 | 208212 |
| BB61 | 144156 | 166206 | 109125 | 168172 | 147159 | 172212 |
| BB61 | 116160 | 162178 | 121129 | 132132 | 131167 | 212212 |
| BB61 | 164170 | 162182 | 109117 | 113158 | 135171 | 208212 |
| BB61 | 148148 | 162210 | 105125 | 128155 | 155167 | 216216 |
| BB61 | 140206 | 174202 | 109125 | 121160 | 151187 | 212216 |
| BB61 | 168202 | 166178 | 101109 | 142154 | 139179 | 172220 |
| BB61 | 160160 | 202218 | 109125 | 113145 | 135191 | 208223 |
| BB61 | 152159 | 174214 | 109109 | 149153 | 143199 | 208224 |
| BB61 | 160176 | 198206 | 109137 | 113164 | 123167 | 226226 |
| BB61 | 168176 | 166174 | 121133 | 188188 | 155199 | 176228 |
| BB61 | 116184 | 170186 | 97101 | 112112 | 147147 | 216232 |
| BB61 | 132140 | 170174 | 113121 | 130145 | 159171 | 220232 |
| BB61 | 156190 | 186206 | 113121 | 138183 | 151175 | 172234 |
| BB61 | 176218 | 162178 | 113121 | 113126 | 159183 | 200238 |
| BB61 | 154168 | 170186 | 121125 | 128164 | 163187 | 160240 |
| BB61 | 168210 | 170206 | 105125 | 117138 | 159175 | 176248 |
| BB61 | 162162 | 174186 | 109121 | 134138 | 191191 | 230248 |
| BB61 | 152234 | 166190 | 113125 | 114158 | 147175 | 180252 |
| BB61 | 152172 | 170194 | 117121 | 126146 | 163175 | 200252 |
| BB61 | 152182 | 162194 | 105113 | 113122 | 143147 | 180254 |
| BB61 | 168172 | 218218 | 109117 | 122122 | 171179 | 236256 |
| BB61 | 156222 | 162170 | 113125 | 132132 | 175183 | 216256 |
| BB61 | 144202 | 210210 | 121125 | 122200 | 131155 | 216260 |
| BB61 | 114206 | 178182 | 109125 | 115140 | 159183 | 236260 |
| BB61 | 164176 | 206206 | 97125 | 109172 | 167171 | 228268 |
| BB61 | 116198 | 182206 | 117137 | 150172 | 147151 | 268272 |
| BB61 | 164190 | 174190 | 105113 | 151156 | 159171 | 240272 |
| BB61 | 136164 | 170202 | 137141 | 140140 | 163183 | 196272 |
| BB61 | 128234 | 170174 | 101105 | 97144 | 115155 | 172276 |
| BB61 | 82160 | 162166 | 121125 | 145164 | 155163 | 208276 |
| BB61 | 144155 | 206226 | 101117 | 105122 | 171207 | 172276 |
| BB61 | 151202 | 178194 | 101109 | 184184 | 163175 | 196280 |
| BB61 | 164216 | 158190 | 117121 | 118134 | 167203 | 172280 |
| BB61 | 156156 | 158170 | 105117 | 90168 | 155179 | 200284 |
| BB61 | 144206 | 166210 | 121125 | 126144 | 175179 | 240284 |
| BB61 | 168182 | 186190 | 105113 | 148148 | 119175 | 252292 |
| BB61 | 152166 | 166206 | 109113 | 164164 | 179183 | 276292 |
| BB61 | 132148 | 162182 | 117117 | 113167 | 151151 | 196296 |
| BB61 | 156198 | 174174 | 117121 | 134147 | 135151 | 220296 |
| BB61 | 164190 | 162194 | 117117 | 144156 | 155171 | 196296 |
| BB61 | 164180 | 186190 | 109117 | 118130 | 171187 | 292296 |
| BB61 | 156160 | 162170 | 109109 | 94126 | 139159 | 288300 |
| BB61 | 140156 | 178210 | 121137 | 113156 | 131163 | 204300 |
| BB61 | 160194 | 182186 | 109117 | 106118 | 151187 | 220300 |
| BB61 | 172194 | 182198 | 109117 | 117200 | 143199 | 208300 |
| BB61 | 160194 | 166166 | 101129 | 110168 | 159175 | 220304 |
| BB61 | 152170 | 182210 | 105121 | 104146 | 175187 | 252304 |
| BB61 | 164168 | 178206 | 97105 | 113144 | 151195 | 276304 |
| BB61 | 156232 | 178178 | 113117 | 164182 | 151175 | 238308 |
| BB61 | 152172 | 194202 | 109121 | 130138 | 151187 | 304308 |
| BB61 | 176194 | 194222 | 105109 | 136144 | 155191 | 172312 |
| BB61 | 120226 | 178194 | 105117 | 136136 | 167247 | 215312 |
| BB61 | 166228 | 186218 | 109117 | 117161 | 159167 | 224314 |
| BB61 | 152180 | 182190 | 105113 | 126144 | 155171 | 160316 |
| BB61 | 172202 | 190194 | 109125 | 152152 | 147151 | 300322 |
| BB61 | 166190 | 194202 | 101105 | 128216 | 179199 | 232328 |
| BB61 | 148180 | 162186 | 101105 | 126134 | 143191 | 312332 |
| BB61 | 164194 | 166214 | 113129 | 118131 | 195199 | 312388 |
| BB61 | 160234 | 166166 | 109109 | 148175 | 171175 | 160172 |
| BB61 | 116172 | 190198 | 113121 | 106107 | 155183 | 180180 |
| BB61 | 140166 | 166182 | 105109 | 126153 | 139159 | 208208 |
| BB61 | 156216 | 186246 | 109117 | 140161 | 155155 | 212212 |
| BB61 | 184214 | 170178 | 105105 | 110115 | 139159 | 201216 |
| BB61 | 184194 | 158194 | 121125 | 142160 | 139167 | 216216 |
| BB61 | 139144 | 170202 | 125141 | 127150 | 163171 | 216216 |
| BB61 | 198210 | 186218 | 105117 | 150150 | 171191 | 204216 |
| BB61 | 140152 | 166190 | 125137 | 151154 | 131135 | 188224 |
| BB61 | 168180 | 174202 | 101121 | 115123 | 159175 | 224224 |
| BB61 | 152232 | 170190 | 101113 | 125152 | 127167 | 178226 |
| BB61 | 148168 | 186190 | 101129 | 143208 | 131143 | 216228 |
| BB61 | 140164 | 178218 | 121125 | 127151 | 159195 | 204228 |
| BB61 | 164168 | 174194 | 105105 | 163176 | 159167 | 220230 |
| BB61 | 156170 | 206206 | 101113 | 147176 | 147171 | 204230 |
| BB61 | 116168 | 178246 | 109121 | 192192 | 155187 | 230230 |
| BB61 | 156182 | 186194 | 109121 | 126180 | 155219 | 182230 |
| BB61 | 144206 | 178194 | 109113 | 135147 | 143159 | 192232 |
| BB61 | 148200 | 170198 | 109109 | 125176 | 143163 | 208232 |
| BB61 | 116144 | 182198 | 121125 | 119184 | 159167 | 216232 |
| BB61 | 152190 | 202206 | 97121 | 119150 | 159187 | 228232 |
| BB61 | 140172 | 162170 | 101125 | 113147 | 123147 | 220234 |
| BB61 | 152152 | 170190 | 109125 | 106147 | 127151 | 228236 |
| BB61 | 124220 | 182198 | 105109 | 110110 | 155175 | 236236 |
| BB61 | 152168 | 166186 | 101113 | 111122 | 139183 | 174236 |
| BB61 | 162176 | 178186 | 105121 | 127127 | 155159 | 230238 |
| BB61 | 140198 | 170190 | 97105 | 155159 | 163195 | 192238 |
| BB61 | 132152 | 186214 | 109117 | 126144 | 147151 | 216242 |
| BB61 | 148220 | 166190 | 109125 | 111111 | 139151 | 188248 |
| BB61 | 152172 | 178198 | 117145 | 103160 | 139147 | 237249 |
| BB61 | 164170 | 202214 | 109117 | 111151 | 159171 | 158256 |
| BB61 | 160160 | 170214 | 105109 | 135139 | 171191 | 250256 |
| BB61 | 160172 | 166186 | 105117 | 159192 | 155195 | 212256 |
| BB61 | 164164 | 162182 | 97105 | 127129 | 167191 | 228260 |
| BB61 | 114152 | 182194 | 113117 | 123127 | 131139 | 264264 |
| BB61 | 152158 | 158198 | 105121 | 143143 | 163187 | 220268 |
| BB61 | 152224 | 166166 | 117133 | 111114 | 115191 | 200272 |
| BB61 | 148178 | 166194 | 109113 | 114151 | 175175 | 208274 |
| BB61 | 156158 | 166166 | 109117 | 113143 | 187187 | 160276 |
| BB61 | 144160 | 170170 | 113121 | 160180 | 147179 | 212280 |
| BB61 | 168170 | 190194 | 121125 | 118134 | 163167 | 282282 |
| BB61 | 152156 | 166182 | 93097 | 111111 | 123171 | 216284 |
| BB61 | 166192 | 182194 | 109121 | 147151 | 155183 | 226284 |
| BB61 | 172228 | 162206 | 105105 | 146164 | 135151 | 230288 |
| BB61 | 156180 | 162166 | 125133 | 115172 | 147167 | 208292 |
| BB61 | 144160 | 166182 | 105109 | 110147 | 159175 | 284292 |
| BB61 | 168198 | 162194 | 105113 | 122150 | 159179 | 216292 |
| BB61 | 148180 | 170186 | 121149 | 91126 | 139159 | 284296 |
| BB61 | 152174 | 178178 | 109125 | 106130 | 167191 | 288296 |
| BB61 | 136140 | 186186 | 101113 | 134156 | 131155 | 182300 |
| BB61 | 144206 | 174174 | 117121 | 126138 | 127151 | 280304 |
| BB61 | 156161 | 166182 | 129133 | 135146 | 159187 | 192304 |
| BB61 | 140148 | 194198 | 117129 | 122130 | 131187 | 216304 |
| BB61 | 148210 | 186194 | 101105 | 102126 | 139199 | 220304 |
| BB61 | 168168 | 158206 | 113121 | 126150 | 127135 | 180308 |
| BB61 | 152198 | 170190 | 109113 | 99130 | 139179 | 312312 |
| BB61 | 140166 | 170190 | 109125 | 188188 | 159183 | 182312 |
| BB61 | 152180 | 198222 | 113129 | 134142 | 131191 | 230316 |
| BB61 | 140156 | 166206 | 109109 | 119169 | 135167 | 234320 |
| BB61 | 132208 | 182234 | 113125 | 135157 | 147171 | 260320 |
| BB61 | 132210 | 182206 | 113117 | 84146 | 163187 | 240332 |
| BB61 | 156198 | 166166 | 117121 | 107133 | 155183 | 292348 |
| BB61 | 152168 | 162166 | 117117 | 129148 | 135195 | 296390 |
| BB61 | 192222 | 178186 | 101105 | 103126 | 155167 | 176394 |
| BB61 | 176210 | 174202 | 105109 | 131146 | 167171 | 228402 |
| BB65 | 116200 | 182190 | 113117 | 117204 | 119183 | 166166 |
| BB65 | 116128 | 162170 | 109117 | 119166 | 155171 | 160168 |
| BB65 | 151156 | 170206 | 109121 | 126130 | 139187 | 168176 |
| BB65 | 170204 | 178202 | 117125 | 131155 | 151167 | 184184 |
| BB65 | 144208 | 166206 | 105109 | 114123 | 159227 | 186186 |
| BB65 | 156160 | 186190 | 109113 | 127155 | 163187 | 188188 |
| BB65 | 160168 | 182210 | 117121 | 140151 | 143155 | 176196 |
| BB65 | 124124 | 166194 | 133145 | 114153 | 151195 | 188196 |
| BB65 | 160186 | 174214 | 105137 | 123168 | 131159 | 188203 |
| BB65 | 160171 | 190210 | 101129 | 133135 | 135159 | 162206 |
| BB65 | 160164 | 174178 | 109117 | 114143 | 155171 | 206206 |
| BB65 | 148152 | 174178 | 97113 | 112159 | 159187 | 200208 |
| BB65 | 166182 | 206210 | 105113 | 144146 | 171215 | 180208 |
| BB65 | 186208 | 178182 | 117121 | 130147 | 143179 | 202210 |
| BB65 | 172200 | 170182 | 101113 | 155155 | 135183 | 194210 |
| BB65 | 168178 | 162182 | 113121 | 122147 | 131139 | 204212 |
| BB65 | 156188 | 214214 | 109113 | 142147 | 159167 | 216216 |
| BB65 | 156160 | 166186 | 105109 | 127179 | 143179 | 200216 |
| BB65 | 164196 | 166186 | 109117 | 131151 | 139155 | 212224 |
| BB65 | 152158 | 186198 | 113141 | 118151 | 171175 | 176224 |
| BB65 | 156160 | 174178 | 109145 | 155143 | 167207 | 174224 |
| BB65 | 116164 | 182190 | 117125 | 106106 | 135139 | 200228 |
| BB65 | 157172 | 186194 | 101117 | 206210 | 159159 | 188228 |
| BB65 | 164170 | 182182 | 105117 | 126180 | 147159 | 228228 |
| BB65 | 164212 | 190190 | 93109 | 114114 | 119167 | 170228 |
| BB65 | 136166 | 186202 | 105109 | 236240 | 159175 | 200228 |
| BB65 | 152156 | 178182 | 101105 | 115151 | 167187 | 176228 |
| BB65 | 176220 | 202214 | 109109 | 107140 | 139195 | 206228 |
| BB65 | 144176 | 174202 | 117121 | 133147 | 139143 | 216230 |
| BB65 | 148190 | 166166 | 109125 | 115162 | 155163 | 208230 |
| BB65 | 116180 | 186202 | 105113 | 176228 | 135151 | 228232 |
| BB65 | 168190 | 174194 | 113125 | 127148 | 167171 | 168234 |
| BB65 | 155156 | 162186 | 109113 | 115134 | 123163 | 160236 |
| BB65 | 158194 | 166186 | 113125 | 127155 | 163167 | 192236 |
| BB65 | 152168 | 186198 | 105129 | 126126 | 135163 | 184238 |
| BB65 | 168172 | 166190 | 117117 | 105148 | 159179 | 162240 |
| BB65 | 152186 | 174194 | 101109 | 162174 | 155179 | 216242 |
| BB65 | 116182 | 170186 | 109129 | 151166 | 175183 | 172242 |
| BB65 | 168184 | 170170 | 109109 | 143147 | 151155 | 214244 |
| BB65 | 116156 | 162170 | 101109 | 118122 | 143159 | 208244 |
| BB65 | 151174 | 170194 | 109125 | 102115 | 163167 | 160248 |
| BB65 | 116148 | 166182 | 105105 | 127135 | 159179 | 216250 |
| BB65 | 198210 | 170194 | 101113 | 126172 | 139159 | 220254 |
| BB65 | 140156 | 174182 | 109117 | 142155 | 131163 | 218256 |
| BB65 | 130144 | 158166 | 101117 | 126151 | 195223 | 232256 |
| BB65 | 166188 | 170174 | 113117 | 122155 | 159175 | 156264 |
| BB65 | 136172 | 178186 | 105117 | 155176 | 187203 | 232264 |
| BB65 | 120152 | 170194 | 109109 | 135200 | 155211 | 176264 |
| BB65 | 172185 | 166174 | 113113 | 119126 | 143147 | 234266 |
| BB65 | 156176 | 178194 | 109133 | 155171 | 127159 | 180266 |
| BB65 | 178190 | 174174 | 105113 | 116122 | 155171 | 208268 |
| BB65 | 148220 | 186194 | 97105 | 119140 | 131155 | 272272 |
| BB65 | 156192 | 198214 | 105113 | 123139 | 155175 | 170272 |
| BB65 | 140174 | 182186 | 97101 | 120123 | 159195 | 272272 |
| BB65 | 207207 | 182194 | 101101 | 149171 | 155167 | 204276 |
| BB65 | 164186 | 162166 | 113117 | 152186 | 155175 | 220276 |
| BB65 | 116116 | 166166 | 105121 | 107158 | 155179 | 212276 |
| BB65 | 144170 | 162194 | 117125 | 107123 | 151199 | 206276 |
| BB65 | 164210 | 166218 | 117121 | 193193 | 159163 | 228280 |
| BB65 | 172222 | 162174 | 113117 | 110127 | 159179 | 188280 |
| BB65 | 140144 | 162198 | 105113 | 142146 | 175203 | 235280 |
| BB65 | 144208 | 170190 | 109113 | 102129 | 155167 | 170284 |
| BB65 | 148165 | 182186 | 101109 | 99139 | 159175 | 198284 |
| BB65 | 152176 | 158210 | 97105 | 102155 | 155171 | 176288 |
| BB65 | 186212 | 174206 | 105121 | 138138 | 167179 | 170288 |
| BB65 | 140234 | 182186 | 105109 | 146147 | 135155 | 280292 |
| BB65 | 116152 | 170186 | 109113 | 143155 | 135163 | 292292 |
| BB65 | 164164 | 194194 | 109113 | 110129 | 131183 | 292292 |
| BB65 | 132164 | 166198 | 117117 | 127158 | 159187 | 222292 |
| BB65 | 116165 | 166198 | 113117 | 98127 | 123191 | 234292 |
| BB65 | 156162 | 166166 | 113113 | 126135 | 167191 | 240292 |
| BB65 | 116116 | 162178 | 105125 | 115126 | 143195 | 241292 |
| BB65 | 180312 | 162182 | 109117 | 152154 | 131151 | 196296 |
| BB65 | 161184 | 182198 | 105113 | 135144 | 159171 | 224296 |
| BB65 | 148180 | 182186 | 93113 | 107136 | 159195 | 236296 |
| BB65 | 116158 | 170226 | 109145 | 134151 | 199227 | 280296 |
| BB65 | 156156 | 170182 | 113121 | 119210 | 119179 | 216300 |
| BB65 | 148186 | 166190 | 105105 | 126136 | 151183 | 288300 |
| BB65 | 116202 | 186190 | 105113 | 144144 | 135187 | 296300 |
| BB65 | 158188 | 166206 | 105121 | 135156 | 139151 | 204304 |
| BB65 | 120156 | 174186 | 105129 | 114140 | 171171 | 236304 |
| BB65 | 132160 | 178206 | 105129 | 122174 | 155171 | 256304 |
| BB65 | 180206 | 162186 | 113129 | 123176 | 143187 | 211304 |
| BB65 | 144216 | 170198 | 109109 | 151178 | 167187 | 218304 |
| BB65 | 168176 | 170194 | 105113 | 127160 | 139215 | 224304 |
| BB65 | 148199 | 182206 | 109117 | 168194 | 155171 | 188308 |
| BB65 | 136184 | 178198 | 109121 | 123220 | 167175 | 292308 |
| BB65 | 132202 | 162218 | 105113 | 127180 | 175183 | 300308 |
| BB65 | 116116 | 158174 | 117125 | 119146 | 155191 | 300308 |
| BB65 | 164180 | 158158 | 113125 | 143171 | 151155 | 292316 |
| BB65 | 144194 | 162162 | 109113 | 123148 | 147159 | 212316 |
| BB65 | 168186 | 170182 | 97109 | 107167 | 159199 | 164318 |
| BB65 | 120158 | 162190 | 117117 | 126163 | 131139 | 300320 |
| BB65 | 152180 | 174190 | 113125 | 109118 | 179195 | 226320 |
| BB65 | 164200 | 162194 | 109117 | 143170 | 167179 | 226324 |
| BB65 | 148186 | 186202 | 105105 | 131175 | 147155 | 280328 |
| BB65 | 136172 | 166186 | 109113 | 157170 | 155179 | 332332 |
| BB65 | 172200 | 170182 | 109141 | 109133 | 151163 | 344344 |
| CC | 152156 | 166178 | 109125 | 111113 | 163171 | 160160 |
| CC | 152168 | 174190 | 105113 | 144202 | 151199 | 164168 |
| CC | 164190 | 178178 | 109125 | 104184 | 167171 | 160176 |
| CC | 148160 | 178198 | 109113 | 144196 | 155175 | 164180 |
| CC | 152204 | 162190 | 113117 | 130159 | 135183 | 168180 |
| CC | 168172 | 170186 | 105109 | 113147 | 123151 | 180188 |
| CC | 148148 | 174202 | 109121 | 150176 | 179183 | 168196 |
| CC | 148164 | 178210 | 105109 | 100148 | 155159 | 168200 |
| CC | 148172 | 162182 | 105117 | 160164 | 127163 | 172200 |
| CC | 184222 | 174210 | 113121 | 122134 | 143175 | 176200 |
| CC | 156160 | 178182 | 113117 | 130168 | 139163 | 196204 |
| CC | 164232 | 170198 | 113133 | 125142 | 139203 | 184208 |
| CC | 136152 | 162178 | 121125 | 112126 | 167171 | 168209 |
| CC | 170202 | 174190 | 121125 | 112156 | 143159 | 212216 |
| CC | 152205 | 162202 | 109113 | 109134 | 151167 | 184216 |
| CC | 136160 | 174190 | 105117 | 118125 | 159175 | 216216 |
| CC | 116148 | 170198 | 109121 | 126164 | 171191 | 208216 |
| CC | 80172 | 170194 | 117117 | 121122 | 143211 | 204216 |
| CC | 136174 | 174194 | 105129 | 155160 | 167183 | 176220 |
| CC | 172234 | 170202 | 101117 | 111160 | 179195 | 215220 |
| CC | 172206 | 166170 | 105109 | 146168 | 171179 | 176223 |
| CC | 144176 | 202210 | 113117 | 151163 | 115183 | 204223 |
| CC | 164164 | 174210 | 105129 | 117190 | 151159 | 184228 |
| CC | 152216 | 194206 | 113117 | 156156 | 159163 | 188228 |
| CC | 152214 | 174190 | 117141 | 155188 | 155167 | 196228 |
| CC | 144172 | 198202 | 121133 | 164168 | 147167 | 208228 |
| CC | 152226 | 190190 | 109109 | 122220 | 159179 | 216228 |
| CC | 136226 | 178202 | 101121 | 130180 | 155187 | 212228 |
| CC | 160168 | 166202 | 113121 | 135171 | 155199 | 168228 |
| CC | 116164 | 166194 | 101109 | 125156 | 155167 | 228231 |
| CC | 132238 | 162190 | 105145 | 134140 | 123155 | 188232 |
| CC | 148164 | 190214 | 105117 | 119125 | 159163 | 228232 |
| CC | 166230 | 206222 | 109121 | 105136 | 151167 | 208232 |
| CC | 120190 | 162162 | 109117 | 118143 | 151171 | 224232 |
| CC | 160168 | 166166 | 105113 | 137200 | 171179 | 208232 |
| CC | 180180 | 166174 | 105105 | 118148 | 183187 | 216232 |
| CC | 180204 | 182190 | 117121 | 130167 | 127195 | 184232 |
| CC | 156160 | 158174 | 117129 | 148164 | 151195 | 232232 |
| CC | 148176 | 178202 | 109125 | 108138 | 171199 | 224232 |
| CC | 144164 | 174234 | 113117 | 130172 | 135183 | 164236 |
| CC | 124164 | 202210 | 117141 | 144200 | 123199 | 224236 |
| CC | 156210 | 158166 | 125133 | 97138 | 135163 | 236239 |
| CC | 168170 | 182198 | 105105 | 121130 | 171171 | 220240 |
| CC | 140210 | 162198 | 105113 | 164164 | 159191 | 222244 |
| CC | 148164 | 198210 | 101117 | 105108 | 187199 | 216244 |
| CC | 80180 | 174202 | 109121 | 105168 | 159163 | 152248 |
| CC | 148202 | 174202 | 109121 | 149239 | 151163 | 228248 |
| CC | 116172 | 190202 | 105125 | 113156 | 147151 | 218252 |
| CC | 148178 | 162170 | 105125 | 126192 | 155179 | 212252 |
| CC | 168198 | 166182 | 101113 | 113125 | 191191 | 212256 |
| CC | 152232 | 186194 | 109145 | 113113 | 167207 | 180256 |
| CC | 154158 | 162178 | 109129 | 152160 | 155171 | 228260 |
| CC | 152202 | 170190 | 109109 | 125142 | 179183 | 216260 |
| CC | 156202 | 170222 | 105105 | 119119 | 143155 | 192268 |
| CC | 152156 | 178186 | 113113 | 135139 | 159167 | 168268 |
| CC | 152152 | 170182 | 105121 | 121136 | 159183 | 204272 |
| CC | 194194 | 186206 | 105109 | 121176 | 139187 | 236274 |
| CC | 156156 | 162162 | 113117 | 126150 | 119135 | 204276 |
| CC | 127132 | 166174 | 105117 | 156156 | 147163 | 252280 |
| CC | 148184 | 174206 | 101133 | 125152 | 159171 | 276280 |
| CC | 144152 | 182182 | 101133 | 117126 | 163167 | 180284 |
| CC | 160192 | 174186 | 113129 | 118163 | 143199 | 240284 |
| CC | 172172 | 182198 | 113117 | 100131 | 119147 | 172288 |
| CC | 164178 | 182186 | 113117 | 122184 | 183187 | 212288 |
| CC | 240256 | 170202 | 105121 | 126131 | 151175 | 188292 |
| CC | 140180 | 170186 | 117125 | 126127 | 115151 | 280296 |
| CC | 172206 | 178186 | 109117 | 130155 | 151155 | 240296 |
| CC | 120132 | 170202 | 121133 | 114161 | 167179 | 212296 |
| CC | 128188 | 178186 | 105105 | 164184 | 167187 | 224296 |
| CC | 152156 | 162178 | 117125 | 141142 | 123171 | 244298 |
| CC | 164172 | 182182 | 117133 | 155190 | 143147 | 252300 |
| CC | 116160 | 158186 | 101117 | 113160 | 127155 | 180300 |
| CC | 120164 | 178186 | 109117 | 140152 | 155167 | 260300 |
| CC | 184198 | 186194 | 113125 | 152171 | 139171 | 264300 |
| CC | 116200 | 186214 | 97121 | 138143 | 155187 | 296300 |
| CC | 116148 | 198202 | 101121 | 121148 | 147167 | 188304 |
| CC | 140182 | 166166 | 109125 | 164164 | 179183 | 192304 |
| CC | 144156 | 162178 | 93129 | 147156 | 143191 | 180304 |
| CC | 148194 | 186198 | 101113 | 132136 | 151155 | 208308 |
| CC | 176178 | 178194 | 105133 | 122148 | 155159 | 292308 |
| CC | 128176 | 182206 | 109125 | 113126 | 143195 | 272308 |
| CC | 168169 | 174178 | 101129 | 122180 | 115143 | 228316 |
| CC | 152212 | 166250 | 113129 | 104168 | 151187 | 200316 |
| CC | 160168 | 166170 | 101133 | 108142 | 159171 | 316320 |
| CC | 132198 | 170190 | 109117 | 118172 | 167183 | 229320 |
| CC | 136186 | 206206 | 93113 | 136138 | 147183 | 248320 |
| CC | 168202 | 162170 | 117121 | 104155 | 151171 | 328328 |
| CC | 152164 | 166202 | 101121 | 126188 | 123167 | 284332 |
| CC | 132168 | 190190 | 109141 | 136140 | 155215 | 192332 |
| CC | 164222 | 182202 | 109125 | 109147 | 139171 | 232336 |
| CC | 148156 | 190194 | 125141 | 105110 | 155187 | 284368 |
| CC | 166198 | 178178 | 109113 | 113144 | 131171 | 300380 |
| CC | 176214 | 190210 | 113121 | 131148 | 143163 | 216388 |
| CC | 172210 | 198228 | 105125 | 121130 | 147179 | 324388 |
| CC | 156160 | 194198 | 101125 | 113130 | 151155 | 156156 |
| CC | 152188 | 162202 | 105125 | 117142 | 163199 | 178178 |
| CC | 144170 | 162174 | 117117 | 113144 | 163163 | 160182 |
| CC | 152152 | 162170 | 109125 | 92160 | 159163 | 178182 |
| CC | 132148 | 174182 | 125133 | 122129 | 163207 | 164184 |
| CC | 156160 | 186222 | 113125 | 108125 | 155167 | 174206 |
| CC | 148214 | 186186 | 109113 | 130156 | 147175 | 166208 |
| CC | 116152 | 190222 | 105121 | 168198 | 171179 | 160208 |
| CC | 116168 | 158182 | 117121 | 100122 | 135183 | 184208 |
| CC | 116160 | 190230 | 117117 | 113168 | 195195 | 202210 |
| CC | 164164 | 178186 | 101117 | 163164 | 163167 | 208212 |
| CC | 140148 | 210222 | 101113 | 126148 | 155171 | 200212 |
| CC | 172194 | 186194 | 117141 | 146147 | 159179 | 214214 |
| CC | 144206 | 166178 | 113129 | 148160 | 131151 | 216216 |
| CC | 168198 | 174182 | 121121 | 156164 | 163167 | 176216 |
| CC | 184214 | 174182 | 125125 | 134144 | 147151 | 218218 |
| CC | 136180 | 166198 | 101109 | 130170 | 159163 | 160218 |
| CC | 152170 | 166174 | 113113 | 122151 | 123155 | 200224 |
| CC | 176193 | 166186 | 117121 | 116144 | 123159 | 182224 |
| CC | 166214 | 198210 | 121133 | 140180 | 155159 | 178228 |
| CC | 168202 | 190190 | 105117 | 102174 | 159163 | 228228 |
| CC | 152156 | 174202 | 109113 | 157168 | 135175 | 224228 |
| CC | 144172 | 166182 | 109133 | 133134 | 151179 | 160228 |
| CC | 148160 | 166174 | 113117 | 135152 | 131151 | 166229 |
| CC | 176192 | 190194 | 109125 | 102106 | 175215 | 230230 |
| CC | 116210 | 170182 | 109125 | 130175 | 131167 | 216232 |
| CC | 144148 | 166182 | 101117 | 130130 | 167187 | 218232 |
| CC | 156176 | 186186 | 105117 | 125130 | 147151 | 212244 |
| CC | 140176 | 178182 | 121125 | 152156 | 135171 | 202244 |
| CC | 160170 | 174202 | 105133 | 133134 | 167183 | 216244 |
| CC | 152176 | 162162 | 121125 | 118160 | 159183 | 237244 |
| CC | 164168 | 178194 | 97105 | 134142 | 167207 | 192244 |
| CC | 116144 | 158166 | 109109 | 125160 | 163207 | 214244 |
| CC | 160166 | 158178 | 105113 | 109155 | 119143 | 158248 |
| CC | 152172 | 166190 | 105105 | 112126 | 171179 | 248248 |
| CC | 202214 | 186198 | 117125 | 102155 | 143147 | 226252 |
| CC | 164176 | 162194 | 113117 | 101126 | 147155 | 180252 |
| CC | 144152 | 178194 | 109133 | 144170 | 135191 | 232252 |
| CC | 152164 | 170198 | 101117 | 143176 | 175199 | 220252 |
| CC | 152162 | 170178 | 113121 | 148152 | 163183 | 204256 |
| CC | 116202 | 186198 | 113125 | 102180 | 123175 | 208264 |
| CC | 168194 | 170182 | 105105 | 164164 | 191191 | 186264 |
| CC | 148160 | 186198 | 109129 | 150164 | 131151 | 160268 |
| CC | 144210 | 186194 | 109113 | 143180 | 167171 | 232268 |
| CC | 144164 | 166190 | 125141 | 101131 | 167171 | 236268 |
| CC | 140152 | 178182 | 101117 | 117130 | 179207 | 204268 |
| CC | 164180 | 170170 | 97113 | 131156 | 139207 | 230268 |
| CC | 148176 | 166170 | 117117 | 114164 | 167175 | 220272 |
| CC | 140210 | 170178 | 105109 | 117179 | 159239 | 260272 |
| CC | 148155 | 186194 | 117129 | 133152 | 139151 | 236276 |
| CC | 164202 | 194206 | 105109 | 100190 | 155167 | 186276 |
| CC | 160180 | 166190 | 105113 | 109160 | 139167 | 272276 |
| CC | 148188 | 166186 | 101105 | 113156 | 135179 | 214276 |
| CC | 164172 | 166194 | 109137 | 162192 | 155159 | 184280 |
| CC | 144160 | 166186 | 109121 | 92112 | 139163 | 232280 |
| CC | 182190 | 178206 | 117121 | 126148 | 155163 | 248280 |
| CC | 152192 | 170202 | 109117 | 152159 | 155187 | 224280 |
| CC | 136164 | 166178 | 113121 | 126145 | 135187 | 158282 |
| CC | 148156 | 170214 | 113113 | 117118 | 147151 | 244284 |
| CC | 152210 | 182186 | 105117 | 134163 | 139163 | 276284 |
| CC | 178222 | 182194 | 97109 | 112121 | 159183 | 212284 |
| CC | 140166 | 166222 | 109121 | 126154 | 171247 | 238284 |
| CC | 152152 | 170178 | 109117 | 151151 | 167179 | 216288 |
| CC | 116168 | 174186 | 105121 | 156159 | 171179 | 224288 |
| CC | 152156 | 182214 | 117121 | 126143 | 159215 | 220288 |
| CC | 190230 | 206226 | 105129 | 96163 | 151151 | 288292 |
| CC | 190230 | 206226 | 105129 | 96163 | 151151 | 292292 |
| CC | 128190 | 158162 | 105113 | 150172 | 135163 | 288292 |
| CC | 156202 | 178190 | 109117 | 160160 | 179215 | 264292 |
| CC | 136180 | 186186 | 109117 | 117130 | 131143 | 166296 |
| CC | 140156 | 170198 | 109109 | 99099 | 159171 | 292296 |
| CC | 144156 | 162178 | 113121 | 122188 | 159179 | 204296 |
| CC | 156186 | 170186 | 105125 | 168204 | 163171 | 272300 |
| CC | 148158 | 166190 | 117125 | 158175 | 163179 | 174300 |
| CC | 148230 | 170218 | 109125 | 130200 | 175179 | 272300 |
| CC | 128190 | 170190 | 101109 | 130160 | 159191 | 264300 |
| CC | 116194 | 186190 | 109113 | 84100 | 167219 | 280300 |
| CC | 152160 | 178186 | 113117 | 134214 | 123159 | 225304 |
| CC | 164170 | 166206 | 101105 | 112113 | 155159 | 232304 |
| CC | 136164 | 202206 | 101129 | 94103 | 163167 | 178304 |
| CC | 152152 | 158178 | 117121 | 105140 | 159171 | 204304 |
| CC | 172176 | 174198 | 109117 | 126164 | 171231 | 268304 |
| CC | 152214 | 174182 | 113129 | 122128 | 123143 | 244308 |
| CC | 168172 | 166170 | 101117 | 121156 | 143155 | 232308 |
| CC | 164176 | 174186 | 109113 | 109133 | 123155 | 264308 |
| CC | 188210 | 170190 | 109117 | 113164 | 167167 | 204312 |
| CC | 151172 | 166206 | 109117 | 134142 | 167183 | 160312 |
| CC | 116152 | 178210 | 109113 | 129133 | 151187 | 308312 |
| CC | 116182 | 166174 | 109113 | 142180 | 159179 | 180320 |
| CC | 164176 | 174186 | 97109 | 148188 | 187211 | 284320 |
| CC | 188210 | 178198 | 101105 | 114122 | 143175 | 234324 |
| CC | 156198 | 178194 | 113125 | 134150 | 159175 | 238380 |
| CC | 136191 | 174190 | 101121 | 156168 | 139147 | 344388 |
| CC | 176218 | 186186 | 105109 | 114148 | 159163 | 224388 |
| CC | 143156 | 158166 | 105113 | 186186 | 135159 | 196392 |
| CC | 116172 | 166198 | 105125 | 105134 | 147163 | 208392 |
| CC | 148160 | 166174 | 117121 | 122146 | 159179 | 388392 |
| CC | 144148 | 186210 | 117137 | 168172 | 131223 | 222400 |
| CC | 160180 | 166170 | 97101 | 122144 | 151179 | 156156 |
| CC | 178214 | 190190 | 109137 | 108129 | 143143 | 200204 |
| CC | 148160 | 190202 | 129129 | 96096 | 151167 | 168204 |
| CC | 164164 | 166186 | 105113 | 130158 | 171171 | 164204 |
| CC | 172226 | 162170 | 101121 | 118148 | 135203 | 152204 |
| CC | 140250 | 170182 | 113117 | 120139 | 147163 | 176208 |
| CC | 140184 | 162182 | 109125 | 108145 | 147163 | 204208 |
| CC | 168168 | 170170 | 101105 | 125144 | 167175 | 204208 |
| CC | 132152 | 190190 | 101137 | 113144 | 171199 | 208208 |
| CC | 140156 | 186202 | 105113 | 116153 | 167183 | 180212 |
| CC | 156206 | 178210 | 105129 | 152156 | 163191 | 212212 |
| CC | 160166 | 194194 | 101117 | 114184 | 183211 | 208212 |
| CC | 152156 | 158194 | 105117 | 109121 | 143147 | 168216 |
| CC | 156185 | 178186 | 117121 | 124161 | 147159 | 208220 |
| CC | 140148 | 182202 | 109125 | 120144 | 159163 | 160221 |
| CC | 160188 | 166182 | 101101 | 109144 | 163179 | 164222 |
| CC | 112140 | 166182 | 105105 | 117178 | 195211 | 209222 |
| CC | 144202 | 178198 | 117117 | 109248 | 147147 | 208224 |
| CC | 140152 | 162186 | 105109 | 145212 | 151155 | 192224 |
| CC | 132132 | 166194 | 105129 | 109148 | 147163 | 220224 |
| CC | 148182 | 162170 | 109117 | 130138 | 147191 | 216224 |
| CC | 136157 | 166174 | 109113 | 130159 | 183195 | 180225 |
| CC | 152190 | 178202 | 101121 | 108134 | 135139 | 228228 |
| CC | 148164 | 174186 | 113113 | 102140 | 159163 | 180228 |
| CC | 156164 | 174186 | 105117 | 112148 | 147179 | 208228 |
| CC | 140148 | 178186 | 105117 | 143156 | 151187 | 172228 |
| CC | 160194 | 166186 | 109117 | 152168 | 159211 | 160228 |
| CC | 166186 | 170174 | 109117 | 113146 | 123155 | 176231 |
| CC | 116168 | 166186 | 109117 | 121148 | 147159 | 156232 |
| CC | 164178 | 166202 | 109121 | 144156 | 159159 | 208232 |
| CC | 190206 | 158198 | 109109 | 131132 | 163195 | 228232 |
| CC | 172202 | 166170 | 109117 | 130158 | 167243 | 232232 |
| CC | 148168 | 174190 | 113121 | 120174 | 143147 | 228236 |
| CC | 152156 | 162182 | 105109 | 122160 | 143155 | 180236 |
| CC | 156178 | 178206 | 101109 | 101126 | 167167 | 236236 |
| CC | 152164 | 182210 | 117125 | 117126 | 179183 | 228236 |
| CC | 156168 | 170194 | 109117 | 130144 | 163167 | 208240 |
| CC | 176208 | 186190 | 105109 | 136176 | 159167 | 216240 |
| CC | 132168 | 170182 | 101109 | 156160 | 159191 | 188240 |
| CC | 186214 | 174178 | 105121 | 126176 | 155155 | 232246 |
| CC | 112164 | 170202 | 113117 | 109140 | 171179 | 208246 |
| CC | 116208 | 162162 | 121121 | 142164 | 143187 | 222252 |
| CC | 152182 | 166190 | 105129 | 104121 | 171191 | 248252 |
| CC | 162164 | 158194 | 109125 | 118162 | 139143 | 224256 |
| CC | 172172 | 182186 | 109121 | 113186 | 155195 | 224256 |
| CC | 136168 | 190210 | 101133 | 124168 | 127139 | 224260 |
| CC | 152186 | 170194 | 105121 | 126216 | 155195 | 232260 |
| CC | 156197 | 162194 | 109125 | 130130 | 171195 | 248260 |
| CC | 136180 | 178186 | 101113 | 144148 | 151155 | 196264 |
| CC | 132176 | 178190 | 109109 | 130180 | 155159 | 232264 |
| CC | 120230 | 174178 | 105109 | 117124 | 143187 | 264264 |
| CC | 136225 | 178190 | 109109 | 117164 | 127147 | 180272 |
| CC | 194222 | 174178 | 109117 | 104113 | 171179 | 224272 |
| CC | 168256 | 182230 | 109109 | 100152 | 123151 | 160276 |
| CC | 192200 | 170182 | 101109 | 118144 | 127199 | 276276 |
| CC | 116132 | 162166 | 113133 | 126172 | 171203 | 228276 |
| CC | 152174 | 174206 | 105113 | 94136 | 159171 | 220280 |
| CC | 156214 | 218222 | 117117 | 180180 | 135159 | 272284 |
| CC | 152152 | 162218 | 101105 | 134138 | 135191 | 184284 |
| CC | 168210 | 170194 | 113121 | 109144 | 167191 | 188284 |
| CC | 234234 | 170206 | 109113 | 124124 | 159191 | 232284 |
| CC | 152200 | 162166 | 109117 | 109152 | 139139 | 280288 |
| CC | 174210 | 190194 | 101109 | 150166 | 159159 | 160288 |
| CC | 140144 | 198198 | 105117 | 104129 | 147163 | 280288 |
| CC | 160160 | 170174 | 101113 | 160192 | 123175 | 204288 |
| CC | 172180 | 182182 | 117121 | 141152 | 127207 | 236288 |
| CC | 182190 | 178178 | 93105 | 156172 | 147159 | 260292 |
| CC | 166176 | 182226 | 97113 | 117147 | 159175 | 212292 |
| CC | 180214 | 170210 | 105125 | 138192 | 175179 | 236292 |
| CC | 148160 | 170202 | 105109 | 117128 | 183195 | 276292 |
| CC | 116160 | 194198 | 113117 | 113130 | 163195 | 292292 |
| CC | 120222 | 166202 | 121121 | 117130 | 139155 | 292296 |
| CC | 122156 | 174190 | 109109 | 156156 | 143163 | 292296 |
| CC | 156168 | 186194 | 105113 | 152164 | 163171 | 288296 |
| CC | 152226 | 178226 | 101139 | 126164 | 155183 | 208296 |
| CC | 162174 | 194214 | 105117 | 144156 | 187191 | 188296 |
| CC | 140152 | 178210 | 113121 | 130156 | 147191 | 296296 |
| CC | 128160 | 178186 | 109129 | 94175 | 175195 | 216296 |
| CC | 172188 | 178210 | 113117 | 130192 | 131151 | 240300 |
| CC | 148152 | 162170 | 113121 | 113121 | 155183 | 296300 |
| CC | 148160 | 174174 | 113121 | 97164 | 159195 | 288300 |
| CC | 152184 | 162222 | 109117 | 125148 | 163163 | 292304 |
| CC | 164192 | 170206 | 109113 | 113163 | 167171 | 184304 |
| CC | 156176 | 174186 | 97105 | 130138 | 155159 | 204308 |
| CC | 160164 | 166206 | 101117 | 143172 | 179195 | 246308 |
| CC | 164194 | 178214 | 113129 | 138156 | 171203 | 280316 |
| CC | 144152 | 182202 | 105121 | 105140 | 175175 | 224324 |
| CC | 172172 | 174178 | 105113 | 160176 | 147183 | 188324 |
| CC | 168178 | 170202 | 105109 | 126212 | 155175 | 244328 |
| CC | 116228 | 178210 | 89121 | 130168 | 163187 | 236336 |
| CC | 152156 | 178186 | 105117 | 134182 | 135171 | 172340 |
| CC | 128176 | 174178 | 113137 | 110180 | 139175 | 232340 |
| CC | 162172 | 166202 | 109117 | 101140 | 163175 | 288356 |
| CC | 156172 | 174194 | 109113 | 129191 | 163171 | 208368 |
| CC | 166176 | 162174 | 109117 | 104156 | 155187 | 376380 |
| CC | 148176 | 170206 | 105117 | 127128 | 159187 | 188192 |
| CC | 144256 | 170190 | 113117 | 107118 | 163175 | 170204 |
| CC | 172192 | 194198 | 109117 | 119134 | 135167 | 200216 |
| CC | 144152 | 186186 | 97117 | 107111 | 135171 | 216216 |
| CC | 140154 | 170174 | 101109 | 139147 | 147175 | 184216 |
| CC | 148164 | 158162 | 97133 | 104180 | 155179 | 172216 |
| CC | 156212 | 162178 | 109117 | 151151 | 163187 | 212216 |
| CC | 172208 | 166170 | 105113 | 139204 | 175203 | 216220 |
| CC | 156160 | 178194 | 105113 | 147220 | 151159 | 176224 |
| CC | 156196 | 166182 | 109125 | 107127 | 159175 | 224224 |
| CC | 136217 | 178210 | 113117 | 151165 | 163207 | 180224 |
| CC | 160164 | 170170 | 109121 | 151186 | 139183 | 184225 |
| CC | 148159 | 178202 | 97109 | 131184 | 175175 | 212236 |
| CC | 160204 | 182206 | 113117 | 143154 | 163183 | 228238 |
| CC | 164212 | 178182 | 125129 | 104104 | 175239 | 225238 |
| CC | 144204 | 194214 | 109113 | 143224 | 163171 | 236252 |
| CC | 164192 | 190210 | 101121 | 127139 | 159159 | 196258 |
| CC | 136164 | 178210 | 117125 | 118126 | 167187 | 196258 |
| CC | 160188 | 166178 | 101105 | 131135 | 147157 | 212260 |
| CC | 140164 | 162174 | 109113 | 147198 | 159183 | 232260 |
| CC | 164166 | 182186 | 109113 | 143169 | 155167 | 234264 |
| CC | 160194 | 166174 | 109117 | 108143 | 147199 | 196268 |
| CC | 144176 | 166170 | 93105 | 107118 | 147167 | 238276 |
| CC | 160208 | 178190 | 105117 | 97139 | 151203 | 176276 |
| CC | 160176 | 198198 | 113113 | 135194 | 135151 | 236280 |
| CC | 164178 | 178190 | 113117 | 103135 | 167167 | 184280 |
| CC | 200208 | 166198 | 101113 | 114173 | 151167 | 228280 |
| CC | 142156 | 178202 | 105121 | 115135 | 175175 | 236280 |
| CC | 186186 | 178198 | 101121 | 139147 | 123159 | 280288 |
| CC | 136160 | 202210 | 109113 | 122163 | 143171 | 220288 |
| CC | 208228 | 178182 | 105109 | 111151 | 147191 | 226288 |
| CC | 164186 | 170174 | 109121 | 134134 | 167195 | 224292 |
| CC | 176224 | 182186 | 109113 | 118154 | 131139 | 212296 |
| CC | 175192 | 178222 | 105137 | 139139 | 155163 | 160296 |
| CC | 140196 | 182198 | 109113 | 123151 | 171183 | 160296 |
| CC | 112112 | 170210 | 105109 | 130143 | 155183 | 210296 |
| CC | 156178 | 162182 | 117133 | 107127 | 179183 | 296296 |
| CC | 152160 | 162198 | 105133 | 131138 | 135203 | 276296 |
| CC | 172176 | 190198 | 113113 | 159250 | 151175 | 232300 |
| CC | 144186 | 174202 | 105121 | 111158 | 171171 | 212304 |
| CC | 140160 | 170202 | 109125 | 127194 | 135215 | 300304 |
| CC | 162204 | 162170 | 105109 | 85151 | 135159 | 280308 |
| CC | 164188 | 174198 | 101125 | 119159 | 147179 | 250308 |
| CC | 154156 | 162186 | 109109 | 123135 | 155187 | 296308 |
| CC | 172196 | 166178 | 105117 | 115138 | 159171 | 219312 |
| CC | 172172 | 182194 | 109121 | 130163 | 155171 | 224316 |
| CC | 152188 | 166218 | 113133 | 115135 | 147155 | 328332 |
| CC | 182188 | 174186 | 105113 | 131131 | 143155 | 252394 |
| CC | 164167 | 182182 | 117129 | 131135 | 147167 | 334394 |
| CC | 144200 | 170198 | 121133 | 114118 | 147183 | 180394 |
| CC | 152160 | 166174 | 109121 | 104132 | 163191 | 164164 |
| CC | 148168 | 190210 | 105121 | 120136 | 139163 | 158170 |
| CC | 128140 | 170194 | 113117 | 139150 | 147163 | 176176 |
| CC | 156156 | 162190 | 113113 | 114122 | 135195 | 176176 |
| CC | 148210 | 162170 | 97101 | 135139 | 159159 | 196196 |
| CC | 176198 | 162182 | 105105 | 141176 | 135159 | 174217 |
| CC | 156168 | 178186 | 113129 | 119136 | 139171 | 206218 |
| CC | 128210 | 174190 | 117117 | 101156 | 155183 | 196222 |
| CC | 168216 | 190198 | 113117 | 114151 | 147183 | 222222 |
| CC | 156194 | 174190 | 101117 | 160188 | 139139 | 154224 |
| CC | 156172 | 182226 | 105109 | 99124 | 151151 | 208224 |
| CC | 191222 | 158182 | 113113 | 133201 | 147171 | 220224 |
| CC | 128156 | 174198 | 113117 | 132164 | 163179 | 208224 |
| CC | 164180 | 178202 | 89105 | 123143 | 163163 | 174228 |
| CC | 140180 | 162170 | 105109 | 130131 | 171175 | 228228 |
| CC | 188252 | 178198 | 105117 | 171175 | 147251 | 220228 |
| CC | 180198 | 174194 | 89113 | 101131 | 135179 | 196229 |
| CC | 188224 | 190194 | 113121 | 147148 | 131147 | 229232 |
| CC | 156182 | 206210 | 113117 | 115135 | 179187 | 196232 |
| CC | 120180 | 206206 | 97105 | 114147 | 163223 | 215232 |
| CC | 148176 | 170174 | 101105 | 106120 | 147159 | 158233 |
| CC | 164164 | 166190 | 109129 | 118162 | 167175 | 158234 |
| CC | 143208 | 166186 | 109109 | 110147 | 151175 | 211235 |
| CC | 172260 | 158170 | 109109 | 95128 | 155183 | 178236 |
| CC | 172180 | 198202 | 109117 | 115120 | 171219 | 196236 |
| CC | 132151 | 166170 | 109137 | 127127 | 147183 | 218243 |
| CC | 148176 | 178190 | 121129 | 102184 | 159183 | 168244 |
| CC | 148164 | 186198 | 101113 | 97126 | 131175 | 184248 |
| CC | 172172 | 178190 | 105105 | 115118 | 127131 | 178250 |
| CC | 116186 | 186206 | 109125 | 99115 | 151183 | 166268 |
| CC | 144180 | 182184 | 109113 | 102164 | 151151 | 188276 |
| CC | 152186 | 194210 | 113121 | 114139 | 147151 | 210280 |
| CC | 132160 | 178198 | 101117 | 119135 | 151163 | 178280 |
| CC | 208216 | 178210 | 105113 | 135179 | 143187 | 216280 |
| CC | 204206 | 166186 | 113133 | 143160 | 171191 | 212280 |
| CC | 188204 | 166198 | 125125 | 119167 | 147195 | 158280 |
| CC | 148156 | 190206 | 105137 | 115126 | 139163 | 216284 |
| CC | 156168 | 166210 | 125125 | 114135 | 147167 | 220284 |
| CC | 164176 | 162170 | 101109 | 176176 | 139167 | 280284 |
| CC | 140214 | 166174 | 109117 | 118143 | 139179 | 225288 |
| CC | 140198 | 170174 | 105105 | 119128 | 179187 | 236288 |
| CC | 160181 | 174202 | 97113 | 158163 | 207215 | 216288 |
| CC | 116176 | 166190 | 109125 | 147155 | 187215 | 288288 |
| CC | 116200 | 166182 | 113121 | 110188 | 135147 | 220292 |
| CC | 132206 | 170190 | 109113 | 177181 | 139155 | 252292 |
| CC | 164164 | 174194 | 109113 | 155159 | 183183 | 218292 |
| CC | 116152 | 178206 | 113125 | 130135 | 155187 | 284292 |
| CC | 136176 | 186202 | 117125 | 151163 | 123191 | 192292 |
| CC | 116173 | 166170 | 109109 | 100128 | 155211 | 198292 |
| CC | 151192 | 166206 | 109113 | 115155 | 179219 | 280292 |
| CC | 136156 | 162186 | 117129 | 136167 | 147151 | 222296 |
| CC | 112216 | 170174 | 109129 | 119159 | 175195 | 174296 |
| CC | 160176 | 166178 | 101109 | 146146 | 155195 | 242296 |
| CC | 164176 | 182186 | 105105 | 123131 | 151183 | 228300 |
| CC | 152152 | 162182 | 109113 | 176199 | 159187 | 296300 |
| CC | 150172 | 186230 | 101113 | 141160 | 159167 | 300304 |
| CC | 160192 | 170186 | 109133 | 119130 | 159175 | 300304 |
| CC | 152194 | 174198 | 109133 | 123154 | 139199 | 240304 |
| CC | 156184 | 170198 | 121121 | 111194 | 151203 | 222304 |
| CC | 112148 | 170222 | 101133 | 151151 | 155187 | 216308 |
| CC | 140204 | 182198 | 121129 | 123131 | 131171 | 174310 |
| CC | 156188 | 166178 | 109113 | 114130 | 163187 | 230312 |
| CC | 148180 | 174178 | 101109 | 119179 | 147155 | 256316 |
| CC | 116198 | 170198 | 109109 | 106123 | 151167 | 188316 |
| CC | 136149 | 170198 | 109121 | 111124 | 139167 | 296316 |
| CC | 148178 | 174186 | 109133 | 147154 | 171171 | 232316 |
| CC | 160168 | 174190 | 109117 | 115159 | 159163 | 296328 |
| CC | 152186 | 186202 | 109117 | 131155 | 147199 | 200352 |
| CC | 160180 | 174190 | 109129 | 107111 | 139155 | 350390 |
| CC | 152160 | 186198 | 121133 | 124171 | 163179 | 300394 |
| CC | 152200 | 166174 | 109113 | 123130 | 131159 | 164164 |
| CC | 188204 | 174186 | 121125 | 119167 | 179187 | 160172 |
| CC | 143166 | 198202 | 101109 | 93119 | 167187 | 158188 |
| CC | 160230 | 178190 | 109117 | 118118 | 143187 | 172190 |
| CC | 164168 | 162166 | 101101 | 127155 | 131163 | 196196 |
| CC | 116152 | 174198 | 105121 | 123123 | 151223 | 172196 |
| CC | 129140 | 194206 | 127131 | 107117 | 155171 | 204212 |
| CC | 152226 | 158166 | 117117 | 143151 | 139211 | 168212 |
| CC | 148164 | 166194 | 101137 | 125158 | 175199 | 172214 |
| CC | 114148 | 166198 | 105117 | 146180 | 147151 | 204216 |
| CC | 160193 | 166178 | 101105 | 131131 | 147159 | 212216 |
| CC | 148160 | 174186 | 101113 | 129138 | 143199 | 172216 |
| CC | 152152 | 174182 | 117121 | 138148 | 163187 | 184218 |
| CC | 156156 | 186194 | 113121 | 194246 | 171183 | 196220 |
| CC | 136218 | 174198 | 121125 | 143175 | 171187 | 188224 |
| CC | 168194 | 166194 | 117125 | 107110 | 175179 | 216230 |
| CC | 172238 | 158158 | 109117 | 126159 | 175175 | 180232 |
| CC | 148160 | 174202 | 117137 | 118159 | 159175 | 220232 |
| CC | 148160 | 186198 | 105121 | 138159 | 155187 | 160234 |
| CC | 144160 | 194194 | 113113 | 106189 | 155235 | 180238 |
| CC | 116156 | 162182 | 105137 | 114139 | 171175 | 180239 |
| CC | 139164 | 186194 | 105109 | 148160 | 123163 | 225244 |
| CC | 116176 | 162178 | 109109 | 134168 | 147159 | 234246 |
| CC | 70172 | 182186 | 109137 | 138143 | 151175 | 176252 |
| CC | 144160 | 162214 | 109109 | 120152 | 163187 | 208252 |
| CC | 168202 | 190194 | 113113 | 120127 | 151179 | 227254 |
| CC | 148168 | 178206 | 105113 | 107155 | 171187 | 158256 |
| CC | 116152 | 166174 | 101125 | 127151 | 151163 | 238260 |
| CC | 168202 | 166178 | 101113 | 122130 | 159163 | 256260 |
| CC | 182222 | 162186 | 109113 | 118151 | 131139 | 184268 |
| CC | 116168 | 166186 | 117125 | 107130 | 127127 | 208272 |
| CC | 148186 | 162186 | 101113 | 111115 | 147171 | 228272 |
| CC | 116156 | 166182 | 97109 | 131159 | 159199 | 268272 |
| CC | 148160 | 166166 | 109117 | 119134 | 167167 | 236274 |
| CC | 116148 | 178190 | 105131 | 111184 | 163171 | 236280 |
| CC | 148206 | 166186 | 101113 | 111143 | 143175 | 208280 |
| CC | 112188 | 178190 | 109109 | 134147 | 151167 | 192284 |
| CC | 148176 | 162210 | 105117 | 109171 | 151183 | 250284 |
| CC | 164214 | 214226 | 113117 | 88147 | 139183 | 286290 |
| CC | 116156 | 174186 | 129141 | 146152 | 151159 | 250292 |
| CC | 148194 | 178186 | 109133 | 118150 | 151171 | 204292 |
| CC | 156160 | 202230 | 109125 | 159159 | 151183 | 272292 |
| CC | 168168 | 178184 | 105109 | 107148 | 167175 | 223294 |
| CC | 144198 | 182182 | 105109 | 147176 | 147163 | 288296 |
| CC | 135148 | 174178 | 105105 | 115156 | 179187 | 174296 |
| CC | 152206 | 186194 | 105121 | 130177 | 167195 | 292296 |
| CC | 152194 | 170182 | 101117 | 127177 | 123163 | 230300 |
| CC | 152196 | 162174 | 109117 | 126187 | 159167 | 216300 |
| CC | 152214 | 166198 | 117129 | 115143 | 139175 | 172300 |
| CC | 134148 | 174186 | 105117 | 126152 | 151183 | 270300 |
| CC | 160190 | 166170 | 109117 | 131131 | 127183 | 172304 |
| CC | 140244 | 174194 | 113137 | 123135 | 139139 | 216308 |
| CC | 156206 | 170198 | 109121 | 143162 | 159179 | 217308 |
| CC | 156174 | 174190 | 113117 | 123149 | 171187 | 268308 |
| CC | 140144 | 170198 | 113121 | 140140 | 123167 | 264312 |
| CC | 176180 | 166186 | 109117 | 101133 | 127175 | 300316 |
| CC | 140222 | 162162 | 125133 | 139151 | 123183 | 208316 |
| CC | 156196 | 170202 | 129137 | 134172 | 123191 | 296338 |
| CC | 148148 | 182198 | 105141 | 134160 | 155155 | 340344 |
| CC | 164172 | 166186 | 89129 | 139147 | 123147 | 230360 |
| CC | 130130 | 166194 | 97109 | 114139 | 139163 | 308376 |
| CC | 160168 | 198202 | 125125 | 126157 | 175215 | 248388 |
| CC | 152186 | 190210 | 101105 | 107107 | 139143 | 212392 |
| CC | 116144 | 190202 | 109129 | 118163 | 135155 | 234392 |
| CC | 164192 | 170190 | 105117 | 118147 | 123159 | 212392 |
| CC | 116148 | 170190 | 109117 | 131131 | 179183 | 168184 |
| CC | 152168 | 178198 | 117117 | 118139 | 123127 | 196200 |
| CC | 168168 | 162194 | 109129 | 109150 | 159159 | 176200 |
| CC | 140164 | 190206 | 105129 | 115182 | 147151 | 164208 |
| CC | 168202 | 162170 | 109125 | 123126 | 139159 | 176212 |
| CC | 148190 | 166166 | 117117 | 110159 | 163163 | 204212 |
| CC | 152174 | 166186 | 113145 | 150163 | 131159 | 168216 |
| CC | 152160 | 174178 | 109125 | 106115 | 163175 | 208220 |
| CC | 148180 | 162166 | 117117 | 130163 | 163195 | 212224 |
| CC | 166194 | 170182 | 113121 | 109175 | 179199 | 184228 |
| CC | 168186 | 194198 | 117121 | 171244 | 155215 | 212228 |
| CC | 164176 | 210218 | 93113 | 126139 | 143155 | 192230 |
| CC | 144198 | 190190 | 105125 | 127224 | 159199 | 212230 |
| CC | 152152 | 194194 | 117125 | 97192 | 159163 | 168234 |
| CC | 116152 | 182182 | 105113 | 151200 | 147155 | 230236 |
| CC | 168182 | 174206 | 113121 | 110127 | 171179 | 196236 |
| CC | 136160 | 174186 | 109117 | 106119 | 163183 | 184236 |
| CC | 116176 | 170182 | 109125 | 122188 | 151195 | 188236 |
| CC | 204208 | 178218 | 117117 | 138167 | 163183 | 242242 |
| CC | 160170 | 158198 | 105105 | 119126 | 135179 | 176246 |
| CC | 152212 | 170182 | 101117 | 106137 | 131139 | 160252 |
| CC | 138148 | 194198 | 109137 | 115179 | 159167 | 230256 |
| CC | 160172 | 194198 | 125125 | 114131 | 151187 | 204256 |
| CC | 130156 | 170182 | 117133 | 122161 | 143203 | 258258 |
| CC | 156162 | 166174 | 105113 | 122167 | 139151 | 220262 |
| CC | 152160 | 166166 | 105117 | 131142 | 159167 | 212262 |
| CC | 198200 | 158186 | 121129 | 114135 | 151171 | 244262 |
| CC | 152164 | 174182 | 109109 | 147175 | 143187 | 208264 |
| CC | 152152 | 170178 | 125129 | 131196 | 135151 | 258266 |
| CC | 152168 | 166198 | 109113 | 145155 | 123163 | 216268 |
| CC | 152156 | 170178 | 101105 | 126170 | 135159 | 250274 |
| CC | 148216 | 170202 | 101109 | 131163 | 155175 | 160276 |
| CC | 184224 | 162182 | 117117 | 130134 | 135147 | 232280 |
| CC | 156174 | 170198 | 101109 | 129146 | 151159 | 172284 |
| CC | 152180 | 166174 | 105133 | 147147 | 163167 | 232284 |
| CC | 180198 | 154194 | 109113 | 175216 | 159179 | 284288 |
| CC | 168202 | 190206 | 113149 | 127250 | 159195 | 196288 |
| CC | 148224 | 162190 | 101113 | 192192 | 143147 | 164292 |
| CC | 168216 | 182210 | 125125 | 154188 | 147147 | 288292 |
| CC | 152228 | 162194 | 117129 | 139154 | 135183 | 236292 |
| CC | 116156 | 182190 | 109113 | 131139 | 175183 | 224296 |
| CC | 182216 | 166218 | 105109 | 106151 | 151191 | 224296 |
| CC | 148236 | 182202 | 97109 | 126135 | 151163 | 208300 |
| CC | 148152 | 170194 | 105113 | 130148 | 143167 | 230300 |
| CC | 152160 | 154186 | 105109 | 127127 | 171175 | 292300 |
| CC | 152168 | 166170 | 109121 | 114151 | 159167 | 230304 |
| CC | 134200 | 178194 | 109113 | 119141 | 151171 | 300304 |
| CC | 164182 | 170186 | 109113 | 143192 | 139187 | 226308 |
| CC | 148148 | 166206 | 129137 | 122122 | 171191 | 228312 |
| CC | 216216 | 178202 | 109121 | 127147 | 151183 | 228316 |
| CC | 164188 | 182186 | 113121 | 121136 | 179187 | 312316 |
| CC | 156160 | 158198 | 109113 | 135159 | 183191 | 272316 |
| CC | 148196 | 194198 | 105109 | 137142 | 167171 | 168320 |
| CC | 158196 | 162206 | 97113 | 109141 | 143143 | 260387 |
| CC | 144180 | 162210 | 105109 | 131155 | 143159 | 238387 |
| CC | 148156 | 186190 | 117121 | 110118 | 179199 | 272387 |
| CC | 148184 | 178182 | 101109 | 107131 | 163203 | 296387 |
| CC | 152228 | 182182 | 109113 | 114143 | 159163 | 188391 |
| DRL | 144196 | 166166 | 101129 | 123167 | 187239 | 160164 |
| DRL | 155198 | 162166 | 109129 | 128163 | 155179 | 162166 |
| DRL | 185190 | 186194 | 113133 | 113176 | 151175 | 174174 |
| DRL | 160164 | 166166 | 113113 | 119151 | 155171 | 184184 |
| DRL | 172202 | 170190 | 113113 | 100162 | 179179 | 160200 |
| DRL | 78116 | 162190 | 105113 | 119147 | 155159 | 162212 |
| DRL | 176180 | 190210 | 101109 | 123123 | 147163 | 212212 |
| DRL | 165220 | 162166 | 105113 | 133196 | 139163 | 174214 |
| DRL | 148190 | 198206 | 109125 | 110134 | 147151 | 192216 |
| DRL | 156212 | 166170 | 109113 | 131142 | 143175 | 216216 |
| DRL | 164198 | 162186 | 105105 | 115142 | 171179 | 212219 |
| DRL | 152190 | 182186 | 109113 | 150182 | 155167 | 184220 |
| DRL | 155156 | 186214 | 113113 | 111124 | 163171 | 220220 |
| DRL | 170188 | 198214 | 121145 | 115119 | 167187 | 208220 |
| DRL | 128198 | 170170 | 109121 | 103146 | 155175 | 174224 |
| DRL | 164194 | 194194 | 101105 | 122130 | 163179 | 224224 |
| DRL | 148176 | 202222 | 105121 | 115170 | 167179 | 200228 |
| DRL | 148152 | 178226 | 109113 | 134134 | 155191 | 216228 |
| DRL | 116152 | 178210 | 113129 | 131173 | 147179 | 229229 |
| DRL | 156160 | 194194 | 109117 | 119151 | 163183 | 230230 |
| DRL | 172196 | 174186 | 101105 | 145145 | 159191 | 188232 |
| DRL | 116160 | 166186 | 109117 | 132150 | 139167 | 220234 |
| DRL | 200208 | 170178 | 101129 | 163163 | 123183 | 236236 |
| DRL | 168202 | 190190 | 105105 | 138143 | 147167 | 172238 |
| DRL | 170204 | 170178 | 105105 | 127151 | 159167 | 176240 |
| DRL | 148156 | 194206 | 105105 | 128208 | 163179 | 240240 |
| DRL | 172185 | 178210 | 113125 | 146154 | 135155 | 174242 |
| DRL | 116164 | 158194 | 101105 | 146146 | 171183 | 228243 |
| DRL | 156156 | 170186 | 97125 | 110163 | 147167 | 236244 |
| DRL | 116148 | 166190 | 125133 | 118188 | 163179 | 233244 |
| DRL | 152156 | 174194 | 113129 | 151159 | 167199 | 196244 |
| DRL | 155176 | 178194 | 105113 | 111119 | 151159 | 236246 |
| DRL | 174202 | 182198 | 105109 | 103103 | 151159 | 214249 |
| DRL | 144204 | 174190 | 101109 | 142214 | 147151 | 226252 |
| DRL | 152164 | 186190 | 113117 | 115115 | 139171 | 242252 |
| DRL | 148164 | 190202 | 109129 | 119150 | 179199 | 256256 |
| DRL | 164208 | 174182 | 101113 | 135147 | 151171 | 208260 |
| DRL | 116164 | 166178 | 117129 | 154159 | 155171 | 260260 |
| DRL | 176176 | 162194 | 109129 | 130190 | 163175 | 232264 |
| DRL | 162164 | 174190 | 105117 | 135175 | 187191 | 216264 |
| DRL | 153208 | 166178 | 97125 | 168179 | 167175 | 224266 |
| DRL | 116174 | 174186 | 113129 | 127142 | 143163 | 162268 |
| DRL | 128156 | 186186 | 125129 | 146188 | 163187 | 244268 |
| DRL | 164216 | 170210 | 105113 | 103155 | 143163 | 229270 |
| DRL | 149153 | 178182 | 113129 | 107163 | 155155 | 158272 |
| DRL | 156172 | 174190 | 117129 | 143175 | 135167 | 184272 |
| DRL | 196196 | 174186 | 117121 | 119166 | 163215 | 272272 |
| DRL | 136181 | 158194 | 105145 | 136167 | 175199 | 254274 |
| DRL | 140168 | 186190 | 105129 | 135167 | 155155 | 276276 |
| DRL | 176206 | 170178 | 105121 | 118142 | 179187 | 272280 |
| DRL | 148192 | 162162 | 109117 | 118139 | 147163 | 236284 |
| DRL | 148152 | 166174 | 121125 | 135155 | 139179 | 280284 |
| DRL | 192208 | 174206 | 113125 | 106146 | 163191 | 284284 |
| DRL | 140208 | 170194 | 113113 | 103155 | 127155 | 214288 |
| DRL | 185207 | 190198 | 117125 | 127143 | 159163 | 204288 |
| DRL | 174206 | 182194 | 113117 | 131186 | 135167 | 184288 |
| DRL | 175242 | 170186 | 101109 | 176202 | 143167 | 228292 |
| DRL | 140212 | 182202 | 109121 | 106131 | 167187 | 216292 |
| DRL | 148188 | 170198 | 113133 | 103147 | 135175 | 220296 |
| DRL | 184184 | 162166 | 109121 | 156194 | 139175 | 225296 |
| DRL | 116156 | 174178 | 109121 | 128136 | 143179 | 234296 |
| DRL | 116140 | 170194 | 113113 | 135136 | 151151 | 288300 |
| DRL | 112148 | 186186 | 105121 | 102126 | 143179 | 288300 |
| DRL | 156178 | 166210 | 109129 | 134166 | 139191 | 236300 |
| DRL | 144176 | 166202 | 101125 | 143180 | 127195 | 187300 |
| DRL | 144152 | 162166 | 109109 | 130135 | 175195 | 244300 |
| DRL | 160160 | 194222 | 117129 | 135178 | 199211 | 212300 |
| DRL | 148164 | 186194 | 117117 | 111126 | 131211 | 260300 |
| DRL | 152206 | 178182 | 109113 | 115156 | 163231 | 224300 |
| DRL | 160168 | 158174 | 105113 | 135150 | 159159 | 168308 |
| DRL | 148156 | 166170 | 105121 | 127130 | 155163 | 192308 |
| DRL | 116116 | 194226 | 101125 | 105111 | 155183 | 170308 |
| DRL | 160196 | 186190 | 101117 | 111155 | 155183 | 187308 |
| DRL | 147168 | 170186 | 113121 | 128131 | 179195 | 232308 |
| DRL | 144192 | 186194 | 117121 | 143167 | 139175 | 296312 |
| DRL | 172182 | 178194 | 97117 | 143159 | 155159 | 216316 |
| DRL | 180212 | 178182 | 101121 | 132132 | 155175 | 304316 |
| DRL | 176208 | 170186 | 109113 | 123175 | 147151 | 168320 |
| DRL | 144152 | 170178 | 121121 | 127163 | 123155 | 292324 |
| DRL | 160160 | 170186 | 105113 | 119167 | 135163 | 230328 |
| DRL | 212220 | 162178 | 109109 | 162162 | 159239 | 180330 |
| DRL | 148160 | 166178 | 109109 | 100119 | 167183 | 204332 |
| DRL | 155164 | 174202 | 105113 | 143180 | 143179 | 212348 |
| DRL | 144156 | 166178 | 109117 | 127201 | 163163 | 200390 |
| DRL | 152188 | 170190 | 109117 | 139155 | 167207 | 220390 |
| DRL | 72116 | 178182 | 101121 | 135169 | 139163 | 280392 |
| DRL | 185187 | 170182 | 105113 | 127131 | 143151 | 284394 |
| DRL | 116196 | 162222 | 125125 | 131167 | 147175 | 168396 |
| GSL | 162212 | 162186 | 109113 | 119119 | 171191 | 160164 |
| GSL | 164180 | 166182 | 109121 | 107155 | 159167 | 172184 |
| GSL | 140168 | 186206 | 109129 | 130184 | 175175 | 184184 |
| GSL | 152170 | 186190 | 109125 | 111135 | 155163 | 196200 |
| GSL | 148148 | 178190 | 109137 | 133170 | 155179 | 192208 |
| GSL | 116144 | 182198 | 101105 | 122188 | 171187 | 208208 |
| GSL | 152188 | 178210 | 101133 | 145203 | 159199 | 200208 |
| GSL | 148168 | 198198 | 113113 | 118119 | 159171 | 172211 |
| GSL | 144156 | 166174 | 121121 | 155201 | 127127 | 216216 |
| GSL | 148148 | 178182 | 113109 | 118122 | 139163 | 208216 |
| GSL | 84134 | 190198 | 109121 | 149150 | 159171 | 184216 |
| GSL | 116152 | 178202 | 109113 | 138166 | 139147 | 204220 |
| GSL | 156216 | 170170 | 105117 | 151159 | 167171 | 226226 |
| GSL | 148172 | 174190 | 101105 | 115139 | 167171 | 222228 |
| GSL | 152176 | 174214 | 101121 | 127139 | 131171 | 176229 |
| GSL | 156160 | 214218 | 105117 | 133134 | 159183 | 212230 |
| GSL | 148206 | 190206 | 113121 | 106111 | 143163 | 160232 |
| GSL | 152226 | 186190 | 117141 | 115130 | 131163 | 176236 |
| GSL | 172198 | 178198 | 113113 | 121142 | 151179 | 233237 |
| GSL | 148164 | 178194 | 97121 | 135151 | 143183 | 208240 |
| GSL | 132148 | 170182 | 97109 | 155170 | 131159 | 216242 |
| GSL | 184242 | 174186 | 97113 | 153168 | 139167 | 228242 |
| GSL | 156168 | 170202 | 117129 | 123167 | 163179 | 204244 |
| GSL | 162180 | 170202 | 101117 | 131194 | 147195 | 160244 |
| GSL | 140178 | 170182 | 109109 | 134171 | 147163 | 196246 |
| GSL | 148204 | 182214 | 117125 | 143184 | 175239 | 224252 |
| GSL | 156172 | 162190 | 113125 | 147147 | 151151 | 184256 |
| GSL | 152180 | 162182 | 117117 | 111134 | 155155 | 256256 |
| GSL | 156180 | 158174 | 109113 | 123204 | 155167 | 252260 |
| GSL | 156208 | 166166 | 105121 | 154155 | 143179 | 260260 |
| GSL | 156180 | 182194 | 109113 | 119147 | 175199 | 228260 |
| GSL | 160194 | 178198 | 117129 | 107200 | 127167 | 180264 |
| GSL | 148160 | 166174 | 109125 | 115147 | 167179 | 188264 |
| GSL | 116160 | 162178 | 113117 | 130182 | 179179 | 204268 |
| GSL | 156172 | 166178 | 105105 | 143159 | 155207 | 168272 |
| GSL | 172172 | 174186 | 109129 | 107155 | 139143 | 176276 |
| GSL | 124180 | 166178 | 101121 | 154170 | 147183 | 176276 |
| GSL | 160188 | 206210 | 113125 | 114167 | 131159 | 280284 |
| GSL | 198230 | 162170 | 105113 | 102147 | 139183 | 227284 |
| GSL | 156168 | 174190 | 109121 | 113167 | 151159 | 190288 |
| GSL | 168230 | 174198 | 105125 | 122147 | 163167 | 216288 |
| GSL | 194208 | 182198 | 121129 | 126171 | 135163 | 233292 |
| GSL | 204220 | 174202 | 105105 | 107118 | 171171 | 200296 |
| GSL | 140168 | 178210 | 105109 | 143172 | 155179 | 280296 |
| GSL | 152192 | 206206 | 109113 | 139184 | 143147 | 280300 |
| GSL | 156198 | 170194 | 113121 | 151175 | 139147 | 220304 |
| GSL | 140144 | 162198 | 109121 | 130159 | 151171 | 218304 |
| GSL | 156184 | 162186 | 101105 | 99202 | 163175 | 300304 |
| GSL | 148184 | 178214 | 117105 | 131134 | 183191 | 304308 |
| GSL | 160200 | 166170 | 105109 | 118146 | 127215 | 221308 |
| GSL | 156200 | 194198 | 113125 | 123172 | 139143 | 260312 |
| GSL | 136214 | 170214 | 105109 | 111151 | 135159 | 304320 |
| GSL | 120222 | 174174 | 109117 | 135147 | 127223 | 212324 |
| GSL | 148160 | 190198 | 109113 | 176201 | 159195 | 272328 |
| GSL | 152194 | 170202 | 101117 | 139212 | 151159 | 223390 |
| GSL | 156176 | 166206 | 109109 | 115155 | 167191 | 328398 |
| GSL | 218218 | 170182 | 113125 | 115159 | 183191 | 288410 |
| GSL | 178182 | 162178 | 101109 | 106127 | 159159 | 156156 |
| GSL | 200208 | 174218 | 109117 | 115126 | 155167 | 160160 |
| GSL | 144164 | 162186 | 105121 | 180192 | 143179 | 160160 |
| GSL | 188192 | 162218 | 105109 | 142166 | 147159 | 170204 |
| GSL | 140144 | 178186 | 109113 | 115145 | 163167 | 209209 |
| GSL | 128152 | 166186 | 121129 | 107119 | 147171 | 220224 |
| GSL | 160160 | 174178 | 129145 | 111137 | 163175 | 216224 |
| GSL | 136204 | 186206 | 101109 | 113118 | 119171 | 216226 |
| GSL | 144180 | 210210 | 97129 | 137181 | 139147 | 192234 |
| GSL | 166172 | 170178 | 109129 | 122160 | 139159 | 164234 |
| GSL | 176208 | 190194 | 109121 | 126154 | 167225 | 220234 |
| GSL | 136232 | 174182 | 105105 | 157158 | 171211 | 196256 |
| GSL | 152156 | 166170 | 109121 | 102118 | 135167 | 212264 |
| GSL | 148172 | 166202 | 105137 | 101126 | 139151 | 222272 |
| GSL | 148164 | 186202 | 101109 | 115139 | 151151 | 236272 |
| GSL | 188200 | 190190 | 105133 | 121176 | 199199 | 256276 |
| GSL | 160164 | 178198 | 117117 | 155155 | 123183 | 212284 |
| GSL | 160160 | 178194 | 109117 | 130177 | 143155 | 224288 |
| GSL | 204232 | 198202 | 101125 | 123127 | 155163 | 236288 |
| GSL | 124168 | 186202 | 109113 | 147188 | 143207 | 220288 |
| GSL | 144164 | 186190 | 125129 | 154154 | 171195 | 200292 |
| GSL | 170180 | 158182 | 105105 | 114126 | 175243 | 212296 |
| GSL | 144180 | 186202 | 117141 | 133212 | 127203 | 304304 |
| GSL | 144170 | 170210 | 121149 | 139218 | 159159 | 284308 |
| GSL | 148176 | 170186 | 109109 | 143173 | 175183 | 296312 |
| GSL | 148156 | 178178 | 101105 | 118139 | 139195 | 217312 |
| GSL | 156204 | 170190 | 113113 | 114114 | 163187 | 192314 |
| GSL | 148148 | 170194 | 105109 | 135135 | 163207 | 174316 |
| GSL | 144180 | 158174 | 117117 | 104158 | 159167 | 168172 |
| GSL | 144204 | 186202 | 105109 | 126138 | 163175 | 180180 |
| GSL | 132132 | 166182 | 117121 | 119159 | 115147 | 160188 |
| GSL | 152152 | 174194 | 105117 | 107120 | 175203 | 192192 |
| GSL | 148148 | 206206 | 113125 | 114126 | 179187 | 196196 |
| GSL | 116176 | 190198 | 113117 | 127152 | 191203 | 196196 |
| GSL | 152176 | 178186 | 105121 | 113131 | 175187 | 200200 |
| GSL | 140140 | 170182 | 105109 | 146167 | 147155 | 160204 |
| GSL | 160174 | 162190 | 101121 | 140155 | 155159 | 204204 |
| GSL | 160184 | 162186 | 109113 | 154200 | 175187 | 208208 |
| GSL | 148168 | 174198 | 109133 | 111151 | 155199 | 188208 |
| GSL | 152216 | 162174 | 105113 | 126139 | 143155 | 201212 |
| GSL | 152172 | 174194 | 105105 | 147171 | 175207 | 215215 |
| GSL | 152184 | 194194 | 113121 | 142143 | 167179 | 216216 |
| GSL | 158200 | 166182 | 109121 | 130173 | 155187 | 218218 |
| GSL | 164176 | 166210 | 101113 | 97127 | 123147 | 218222 |
| GSL | 140156 | 186194 | 117133 | 111119 | 119155 | 218222 |
| GSL | 152164 | 178198 | 109129 | 143150 | 143147 | 223223 |
| GSL | 126160 | 162182 | 109117 | 111150 | 159179 | 168226 |
| GSL | 152168 | 174202 | 109133 | 119138 | 179183 | 226226 |
| GSL | 172208 | 170194 | 105109 | 139180 | 155155 | 180236 |
| GSL | 156196 | 190198 | 113141 | 160162 | 147187 | 176240 |
| GSL | 168180 | 142186 | 117125 | 132169 | 143163 | 172250 |
| GSL | 160168 | 162190 | 101117 | 136146 | 139183 | 250250 |
| GSL | 136140 | 170178 | 113113 | 131146 | 139139 | 214254 |
| GSL | 160174 | 178230 | 97117 | 134151 | 139175 | 192258 |
| GSL | 144204 | 170202 | 105109 | 110171 | 139139 | 262262 |
| GSL | 140152 | 198242 | 101109 | 110154 | 155163 | 160262 |
| GSL | 144144 | 174182 | 97105 | 138144 | 155163 | 238280 |
| GSL | 148200 | 166186 | 109121 | 99190 | 167179 | 284284 |
| GSL | 148148 | 162174 | 109117 | 139160 | 135159 | 254292 |
| GSL | 156159 | 174190 | 113121 | 119119 | 159159 | 284292 |
| GSL | 148216 | 162174 | 109117 | 118151 | 151163 | 210292 |
| GSL | 164174 | 178198 | 109125 | 140151 | 155163 | 296296 |
| GSL | 152168 | 162194 | 109117 | 140182 | 123167 | 226304 |
| GSL | 168204 | 166194 | 101105 | 111138 | 159163 | 168320 |
| GSL | 144166 | 174190 | 125129 | 130135 | 163163 | 292391 |
| LL | 148148 | 162166 | 109117 | 146164 | 143163 | 160160 |
| LL | 156202 | 182206 | 105109 | 117130 | 183187 | 176176 |
| LL | 144202 | 166198 | 101101 | 145164 | 123127 | 160184 |
| LL | 116156 | 166174 | 101117 | 128140 | 143195 | 192192 |
| LL | 172174 | 162174 | 101105 | 114136 | 135155 | 196200 |
| LL | 164166 | 158162 | 109125 | 139168 | 171191 | 200200 |
| LL | 136202 | 166186 | 109113 | 158180 | 151207 | 200200 |
| LL | 132202 | 162170 | 105121 | 126138 | 151191 | 176204 |
| LL | 175198 | 210210 | 105109 | 168188 | 139163 | 208208 |
| LL | 160164 | 170174 | 105105 | 108132 | 151155 | 178210 |
| LL | 148194 | 170178 | 117125 | 108150 | 163171 | 204212 |
| LL | 164182 | 154182 | 101105 | 100121 | 147167 | 176216 |
| LL | 152166 | 174190 | 113117 | 152160 | 151191 | 160216 |
| LL | 156198 | 170186 | 101113 | 132196 | 163191 | 216216 |
| LL | 184206 | 190198 | 97097 | 130148 | 127143 | 158219 |
| LL | 148188 | 170190 | 105129 | 102202 | 155175 | 172220 |
| LL | 162206 | 178202 | 109113 | 113184 | 123179 | 168220 |
| LL | 152156 | 182190 | 97117 | 118134 | 171187 | 216220 |
| LL | 172172 | 182198 | 101137 | 109150 | 159191 | 212220 |
| LL | 132136 | 202214 | 113121 | 118128 | 143187 | 164222 |
| LL | 160190 | 166194 | 105113 | 156188 | 123175 | 204224 |
| LL | 164198 | 194202 | 121129 | 130152 | 167199 | 212224 |
| LL | 160216 | 194206 | 105113 | 122125 | 163183 | 180225 |
| LL | 120168 | 178202 | 109117 | 136184 | 179191 | 214226 |
| LL | 148186 | 158186 | 109121 | 109118 | 143147 | 228228 |
| LL | 144198 | 158158 | 109117 | 113140 | 147151 | 188228 |
| LL | 144176 | 162198 | 109121 | 96155 | 135155 | 164228 |
| LL | 156170 | 166178 | 117137 | 106167 | 131187 | 224228 |
| LL | 120172 | 186190 | 109109 | 159160 | 123203 | 224228 |
| LL | 156164 | 190202 | 105117 | 150151 | 179195 | 226229 |
| LL | 168172 | 166194 | 113129 | 114140 | 147159 | 230230 |
| LL | 148152 | 166202 | 109109 | 108168 | 147167 | 204230 |
| LL | 168198 | 194194 | 105129 | 112184 | 127183 | 172230 |
| LL | 116140 | 170186 | 129133 | 118154 | 139159 | 227232 |
| LL | 156188 | 174178 | 113117 | 118139 | 155167 | 220232 |
| LL | 164184 | 166238 | 109121 | 154177 | 155175 | 232232 |
| LL | 188188 | 186198 | 113125 | 122164 | 139151 | 224236 |
| LL | 116120 | 166170 | 101133 | 113142 | 163163 | 212236 |
| LL | 160168 | 170226 | 109129 | 166174 | 167191 | 196236 |
| LL | 156174 | 186194 | 109113 | 126126 | 195195 | 184236 |
| LL | 116218 | 170194 | 109121 | 125150 | 159167 | 196240 |
| LL | 156176 | 198206 | 101121 | 128196 | 171191 | 208240 |
| LL | 172172 | 190198 | 105113 | 117160 | 135187 | 204244 |
| LL | 160192 | 186194 | 105105 | 121150 | 147199 | 168244 |
| LL | 168204 | 178202 | 109109 | 143170 | 159159 | 216248 |
| LL | 116160 | 162174 | 125125 | 168171 | 163167 | 228252 |
| LL | 116152 | 174210 | 101129 | 104108 | 163171 | 220252 |
| LL | 152180 | 162162 | 101117 | 132156 | 151175 | 178252 |
| LL | 152196 | 178186 | 109109 | 164170 | 135167 | 216258 |
| LL | 116168 | 166182 | 133149 | 120140 | 115131 | 230260 |
| LL | 164184 | 158166 | 125125 | 117122 | 151155 | 196260 |
| LL | 156168 | 182198 | 105125 | 136167 | 143179 | 196260 |
| LL | 152152 | 174178 | 113113 | 138141 | 167179 | 204260 |
| LL | 172192 | 170194 | 121133 | 113188 | 135139 | 228264 |
| LL | 164198 | 178198 | 121125 | 121162 | 147171 | 260264 |
| LL | 148186 | 174198 | 109117 | 110146 | 151175 | 244264 |
| LL | 148206 | 202218 | 109109 | 126155 | 115139 | 244272 |
| LL | 152160 | 162198 | 117121 | 144148 | 171175 | 218272 |
| LL | 144156 | 190194 | 117121 | 130131 | 151183 | 208272 |
| LL | 140202 | 182194 | 105109 | 96126 | 203203 | 256272 |
| LL | 172202 | 194202 | 113113 | 114126 | 167191 | 230274 |
| LL | 148148 | 198206 | 101109 | 121128 | 163167 | 229276 |
| LL | 144156 | 174178 | 109109 | 156166 | 131131 | 276280 |
| LL | 144202 | 166198 | 105117 | 122130 | 179179 | 276280 |
| LL | 164198 | 170174 | 101113 | 122136 | 171203 | 238280 |
| LL | 152174 | 186198 | 109117 | 167167 | 135147 | 204284 |
| LL | 210210 | 174182 | 117121 | 109156 | 163163 | 244284 |
| LL | 152198 | 166214 | 113121 | 118139 | 155167 | 212288 |
| LL | 138190 | 182194 | 105109 | 118122 | 139171 | 230288 |
| LL | 160192 | 166178 | 109109 | 134154 | 171175 | 196288 |
| LL | 128164 | 166174 | 105121 | 133151 | 155159 | 232292 |
| LL | 152156 | 174194 | 113117 | 140152 | 143167 | 242292 |
| LL | 132132 | 170214 | 109133 | 98106 | 167223 | 160292 |
| LL | 148172 | 170186 | 113117 | 118152 | 155183 | 160296 |
| LL | 160206 | 182198 | 109121 | 117134 | 155203 | 288296 |
| LL | 144176 | 162182 | 89105 | 126226 | 147211 | 236296 |
| LL | 128194 | 186194 | 101105 | 114125 | 119131 | 288300 |
| LL | 156202 | 174214 | 109117 | 106131 | 135143 | 220300 |
| LL | 202214 | 162170 | 113121 | 136168 | 155163 | 216300 |
| LL | 120186 | 174178 | 97109 | 122136 | 147159 | 258304 |
| LL | 150238 | 158182 | 101121 | 160180 | 163167 | 224304 |
| LL | 116176 | 170186 | 105109 | 115154 | 199199 | 160304 |
| LL | 116192 | 186194 | 101113 | 130184 | 131151 | 280308 |
| LL | 198202 | 186194 | 109109 | 117184 | 131151 | 232310 |
| LL | 170194 | 178190 | 109129 | 146198 | 155175 | 296312 |
| LL | 152176 | 170186 | 97121 | 134136 | 171183 | 168320 |
| LL | 136152 | 178198 | 109109 | 132172 | 167183 | 316320 |
| LL | 152164 | 166170 | 105117 | 126142 | 191191 | 224320 |
| LL | 132172 | 166218 | 133133 | 113125 | 167199 | 288324 |
| LL | 168194 | 162198 | 109109 | 105170 | 159179 | 272328 |
| LL | 168168 | 162190 | 109113 | 113146 | 167227 | 232332 |
| LL | 168172 | 158166 | 101125 | 126134 | 127227 | 160334 |
| LL | 136182 | 178194 | 105109 | 105129 | 175203 | 272340 |
| LL | 152152 | 162174 | 105105 | 120130 | 159175 | 182388 |
| LL | 160176 | 166170 | 101105 | 126130 | 151219 | 200388 |
| LL | 156178 | 174214 | 109125 | 141168 | 147183 | 296392 |
| LL | 184204 | 186186 | 109121 | 111159 | 147147 | 180184 |
| LL | 160164 | 186206 | 109121 | 104135 | 187219 | 180184 |
| LL | 172212 | 166234 | 105117 | 123151 | 143147 | 176196 |
| LL | 176180 | 162166 | 101125 | 155167 | 119207 | 196196 |
| LL | 164208 | 166198 | 121121 | 100115 | 155207 | 172200 |
| LL | 176180 | 186198 | 113117 | 131193 | 187191 | 180206 |
| LL | 156202 | 190206 | 109133 | 109151 | 143155 | 196208 |
| LL | 148174 | 178198 | 109121 | 140148 | 171175 | 170208 |
| LL | 172210 | 194214 | 97105 | 127158 | 135175 | 192210 |
| LL | 156252 | 186202 | 125125 | 108151 | 175203 | 210210 |
| LL | 144148 | 178182 | 101109 | 123150 | 159203 | 170212 |
| LL | 148148 | 178182 | 113145 | 143179 | 131171 | 180214 |
| LL | 188192 | 166190 | 109117 | 96114 | 163195 | 164214 |
| LL | 152172 | 182214 | 129129 | 147151 | 163195 | 219219 |
| LL | 172198 | 174198 | 113121 | 122135 | 139167 | 218220 |
| LL | 136194 | 186206 | 113125 | 123140 | 143179 | 208220 |
| LL | 116189 | 174178 | 109113 | 110115 | 167171 | 219223 |
| LL | 143202 | 162178 | 105109 | 138163 | 139147 | 223226 |
| LL | 184200 | 198206 | 121125 | 126143 | 175191 | 222226 |
| LL | 152190 | 166190 | 109117 | 114122 | 147155 | 216230 |
| LL | 148186 | 162202 | 117125 | 126130 | 143159 | 208230 |
| LL | 170224 | 166178 | 113125 | 104163 | 155159 | 226230 |
| LL | 155164 | 178186 | 117121 | 154184 | 167191 | 172230 |
| LL | 160188 | 166198 | 101125 | 155170 | 147207 | 182231 |
| LL | 184232 | 170190 | 109121 | 111129 | 147151 | 231234 |
| LL | 120160 | 170190 | 117117 | 138159 | 139155 | 214234 |
| LL | 148168 | 194198 | 109121 | 130151 | 143211 | 208234 |
| LL | 164184 | 170174 | 105129 | 113160 | 163171 | 224236 |
| LL | 139160 | 166190 | 105125 | 119173 | 131139 | 210238 |
| LL | 156190 | 166170 | 101121 | 114242 | 163167 | 242242 |
| LL | 156202 | 166178 | 109121 | 116127 | 151159 | 184246 |
| LL | 165188 | 178194 | 117121 | 144180 | 155159 | 236246 |
| LL | 172176 | 182190 | 109109 | 109110 | 171223 | 246246 |
| LL | 152216 | 178254 | 101121 | 114127 | 167171 | 212248 |
| LL | 166172 | 198198 | 109121 | 122188 | 167199 | 230250 |
| LL | 148176 | 190206 | 101109 | 140144 | 167179 | 232254 |
| LL | 186194 | 182190 | 109109 | 159162 | 147159 | 182256 |
| LL | 170210 | 170246 | 97117 | 142158 | 147159 | 234256 |
| LL | 196196 | 178194 | 109133 | 106166 | 135171 | 230272 |
| LL | 144144 | 170198 | 109109 | 109109 | 135223 | 164276 |
| LL | 172186 | 174182 | 113117 | 132160 | 171171 | 264280 |
| LL | 158158 | 166202 | 109121 | 132140 | 171195 | 280280 |
| LL | 168194 | 166218 | 113121 | 105144 | 147151 | 182284 |
| LL | 154160 | 170190 | 125125 | 108118 | 155187 | 280284 |
| LL | 116166 | 166178 | 117121 | 127172 | 147159 | 246288 |
| LL | 136190 | 206210 | 101121 | 150150 | 167179 | 237292 |
| LL | 116144 | 178194 | 105113 | 117163 | 143159 | 288296 |
| LL | 148160 | 166174 | 105121 | 132181 | 171175 | 292296 |
| LL | 156172 | 182190 | 109141 | 129129 | 115187 | 216296 |
| LL | 152184 | 178202 | 109121 | 111138 | 155203 | 239296 |
| LL | 116168 | 166210 | 105125 | 118131 | 135151 | 238300 |
| LL | 168178 | 162170 | 121121 | 176176 | 151167 | 226300 |
| LL | 152190 | 174174 | 109117 | 172211 | 151167 | 252300 |
| LL | 156188 | 166174 | 113129 | 105105 | 167183 | 219300 |
| LL | 140180 | 182186 | 117133 | 112176 | 135195 | 192300 |
| LL | 116148 | 174210 | 97105 | 136144 | 123135 | 184304 |
| LL | 168176 | 170174 | 117125 | 122181 | 143159 | 192304 |
| LL | 116164 | 162194 | 109125 | 109151 | 155195 | 174304 |
| LL | 116162 | 162166 | 121125 | 110154 | 151195 | 216304 |
| LL | 156188 | 174178 | 105117 | 114117 | 191227 | 300304 |
| LL | 176210 | 174182 | 109125 | 125155 | 147187 | 235308 |
| LL | 169214 | 182182 | 113117 | 110174 | 167195 | 264310 |
| LL | 139208 | 174190 | 101105 | 100122 | 147183 | 216316 |
| LL | 144208 | 170170 | 97113 | 112207 | 131151 | 228320 |
| LL | 156172 | 166178 | 101113 | 176194 | 115159 | 274320 |
| LL | 140168 | 162166 | 109121 | 121180 | 159183 | 212320 |
| LL | 156164 | 190194 | 113117 | 125150 | 135147 | 230324 |
| LL | 148186 | 190194 | 105113 | 155168 | 147203 | 234328 |
| LL | 136166 | 166178 | 105109 | 114164 | 139159 | 212394 |
| LL | 146152 | 166194 | 101153 | 123126 | 155191 | 172172 |
| LL | 148154 | 166202 | 101109 | 97115 | 155179 | 188188 |
| LL | 144148 | 162190 | 105121 | 106114 | 151163 | 184192 |
| LL | 164164 | 182198 | 113117 | 122135 | 155187 | 188192 |
| LL | 168212 | 162198 | 101105 | 98178 | 139171 | 176196 |
| LL | 168228 | 186250 | 109117 | 107136 | 127219 | 176204 |
| LL | 140162 | 190190 | 109121 | 110122 | 167187 | 168207 |
| LL | 152160 | 154174 | 105109 | 110119 | 131151 | 208208 |
| LL | 160196 | 182186 | 113113 | 93117 | 151203 | 164212 |
| LL | 164166 | 174182 | 109117 | 110135 | 147151 | 212216 |
| LL | 160168 | 166182 | 109117 | 110146 | 131179 | 216216 |
| LL | 120160 | 174174 | 105113 | 110134 | 135171 | 176220 |
| LL | 156156 | 166178 | 105113 | 127158 | 127179 | 200222 |
| LL | 152184 | 174182 | 121125 | 122147 | 143155 | 196224 |
| LL | 148196 | 182190 | 113121 | 122157 | 175199 | 225232 |
| LL | 172188 | 170178 | 117117 | 111150 | 131147 | 200238 |
| LL | 140148 | 190190 | 109109 | 97146 | 159167 | 216238 |
| LL | 140180 | 170178 | 105137 | 135167 | 163171 | 212238 |
| LL | 164180 | 162178 | 117121 | 105139 | 159175 | 230238 |
| LL | 160161 | 166166 | 105121 | 138146 | 171187 | 238238 |
| LL | 176206 | 174210 | 101113 | 139180 | 123187 | 230240 |
| LL | 200200 | 202206 | 109117 | 154159 | 135163 | 230246 |
| LL | 140156 | 182182 | 101109 | 118118 | 159163 | 250250 |
| LL | 116176 | 174174 | 109113 | 119142 | 127171 | 208250 |
| LL | 148210 | 170206 | 105117 | 135138 | 159171 | 234254 |
| LL | 140152 | 162174 | 121125 | 115139 | 147163 | 240256 |
| LL | 156160 | 158190 | 109121 | 166190 | 159159 | 188258 |
| LL | 120200 | 186206 | 105109 | 139194 | 143167 | 180258 |
| LL | 140156 | 182186 | 117121 | 131135 | 135171 | 212258 |
| LL | 194206 | 174182 | 109117 | 165168 | 159199 | 184258 |
| LL | 148184 | 174182 | 121133 | 122159 | 143175 | 224260 |
| LL | 168184 | 174214 | 105109 | 136151 | 151151 | 268268 |
| LL | 152206 | 190198 | 89133 | 131196 | 143155 | 246276 |
| LL | 160178 | 178198 | 101105 | 97142 | 179199 | 276276 |
| LL | 116172 | 166166 | 113125 | 101111 | 135147 | 228280 |
| LL | 150170 | 182206 | 109113 | 127196 | 163167 | 222280 |
| LL | 140152 | 166214 | 101113 | 100119 | 139203 | 184282 |
| LL | 148156 | 170182 | 113129 | 147151 | 143175 | 164284 |
| LL | 156206 | 186230 | 101105 | 131154 | 191191 | 280284 |
| LL | 176180 | 178182 | 117125 | 136151 | 123151 | 254288 |
| LL | 168178 | 178182 | 109113 | 126131 | 159183 | 212288 |
| LL | 156168 | 174182 | 101121 | 106138 | 151171 | 188292 |
| LL | 164172 | 186194 | 113121 | 106150 | 159175 | 280292 |
| LL | 148156 | 166202 | 109109 | 122158 | 195195 | 242292 |
| LL | 172206 | 166190 | 101109 | 147172 | 159159 | 216296 |
| LL | 164168 | 186202 | 105109 | 123201 | 127171 | 184296 |
| LL | 160211 | 186206 | 113117 | 161176 | 155171 | 212296 |
| LL | 160170 | 190194 | 113113 | 139143 | 159179 | 292296 |
| LL | 160168 | 182190 | 109109 | 143146 | 135159 | 188300 |
| LL | 176214 | 182182 | 117125 | 139171 | 143183 | 230300 |
| LL | 148152 | 190198 | 101101 | 133164 | 147199 | 276300 |
| LL | 184206 | 162186 | 109117 | 110176 | 139155 | 304304 |
| LL | 148224 | 198202 | 129129 | 131134 | 151159 | 188304 |
| LL | 140144 | 174178 | 105121 | 117155 | 171175 | 172304 |
| LL | 196228 | 170170 | 109157 | 146177 | 143159 | 308308 |
| LL | 164202 | 170174 | 117121 | 114135 | 127167 | 192308 |
| LL | 160180 | 170190 | 105121 | 107119 | 167171 | 292320 |
| LL | 136156 | 170174 | 105113 | 137196 | 159195 | 320320 |
| LL | 148152 | 170170 | 109117 | 166238 | 147171 | 230332 |
| LL | 168178 | 174186 | 109117 | 103123 | 151159 | 224388 |
| LL | 156172 | 162210 | 101117 | 144160 | 167187 | 220392 |
| LL | 164194 | 174178 | 109125 | 130176 | 179203 | 226392 |
| LL | 172206 | 186194 | 105117 | 94113 | 163199 | 168168 |
| LL | 156172 | 206206 | 113117 | 97138 | 163195 | 184184 |
| LL | 152162 | 174206 | 117125 | 111115 | 159167 | 196208 |
| LL | 152246 | 166178 | 109125 | 146182 | 171175 | 200208 |
| LL | 132184 | 162166 | 113113 | 127158 | 123171 | 168212 |
| LL | 140160 | 170170 | 109113 | 155201 | 163187 | 200212 |
| LL | 156199 | 190194 | 117125 | 119159 | 123147 | 180216 |
| LL | 156214 | 186210 | 105121 | 115146 | 99171 | 196216 |
| LL | 206210 | 170186 | 105109 | 114135 | 159179 | 180216 |
| LL | 156160 | 182210 | 117141 | 127187 | 135151 | 204217 |
| LL | 144152 | 166182 | 109121 | 127150 | 159167 | 220221 |
| LL | 198214 | 186198 | 105105 | 110130 | 163219 | 176225 |
| LL | 168214 | 190194 | 117117 | 111129 | 131135 | 164228 |
| LL | 136144 | 178186 | 109117 | 114134 | 139159 | 178228 |
| LL | 116208 | 162166 | 109113 | 114146 | 155167 | 164228 |
| LL | 156226 | 186202 | 121125 | 119119 | 195211 | 212228 |
| LL | 144152 | 170186 | 105117 | 96142 | 135179 | 164229 |
| LL | 144144 | 198206 | 109113 | 110155 | 159159 | 220232 |
| LL | 152228 | 174186 | 109141 | 159162 | 167175 | 232234 |
| LL | 88116 | 206206 | 109113 | 119145 | 151159 | 184236 |
| LL | 168190 | 170186 | 117125 | 136162 | 155191 | 204238 |
| LL | 156186 | 162210 | 121137 | 159175 | 159195 | 216238 |
| LL | 160164 | 174190 | 101129 | 134148 | 159163 | 234240 |
| LL | 166166 | 174202 | 121121 | 122150 | 155175 | 232248 |
| LL | 148164 | 190202 | 109129 | 111119 | 139143 | 216250 |
| LL | 194210 | 186206 | 109121 | 131154 | 151151 | 250250 |
| LL | 156164 | 162178 | 109113 | 114136 | 199199 | 226254 |
| LL | 156168 | 210210 | 109137 | 122154 | 147155 | 216262 |
| LL | 160192 | 170202 | 113117 | 147175 | 151179 | 262264 |
| LL | 148182 | 178182 | 105105 | 162175 | 155195 | 188264 |
| LL | 116148 | 170230 | 105125 | 107110 | 167183 | 180268 |
| LL | 160208 | 166210 | 113117 | 106126 | 163163 | 268272 |
| LL | 152156 | 162174 | 105117 | 115144 | 143187 | 176272 |
| LL | 160216 | 162194 | 117125 | 107144 | 139143 | 236276 |
| LL | 156208 | 178202 | 105121 | 135198 | 147171 | 216276 |
| LL | 152160 | 170202 | 109109 | 150180 | 131183 | 200276 |
| LL | 158178 | 186198 | 113121 | 115179 | 159191 | 222276 |
| LL | 194210 | 202202 | 113125 | 118179 | 163175 | 260280 |
| LL | 148152 | 182182 | 113117 | 102115 | 123199 | 238280 |
| LL | 148184 | 158198 | 113125 | 146202 | 159207 | 276280 |
| LL | 138216 | 166178 | 121121 | 147147 | 147159 | 244284 |
| LL | 148171 | 166198 | 113121 | 146146 | 175175 | 222284 |
| LL | 182194 | 186190 | 109117 | 115115 | 123155 | 210288 |
| LL | 140202 | 162194 | 101125 | 131131 | 159159 | 192288 |
| LL | 164168 | 174218 | 109113 | 122159 | 155163 | 180296 |
| LL | 116154 | 170194 | 105109 | 103173 | 179195 | 258296 |
| LL | 180190 | 166174 | 89121 | 139153 | 123159 | 210300 |
| LL | 176190 | 186190 | 117121 | 131136 | 151159 | 216300 |
| LL | 152164 | 166178 | 109109 | 142142 | 159167 | 296300 |
| LL | 156172 | 158174 | 117121 | 131158 | 155155 | 176304 |
| LL | 148156 | 170182 | 101129 | 145184 | 155155 | 184304 |
| LL | 160234 | 178218 | 101129 | 110175 | 143191 | 280304 |
| LL | 160230 | 170170 | 125133 | 103134 | 131211 | 304304 |
| LL | 144152 | 170174 | 113121 | 102126 | 163175 | 232308 |
| LL | 116210 | 162194 | 97117 | 123155 | 135175 | 254308 |
| LL | 140180 | 178182 | 125125 | 118155 | 183203 | 225308 |
| LL | 116238 | 182206 | 101125 | 139171 | 183207 | 192308 |
| LL | 172226 | 190206 | 109121 | 139163 | 139167 | 220312 |
| LL | 152218 | 174214 | 105133 | 123131 | 163183 | 229312 |
| LL | 152214 | 166182 | 101101 | 131151 | 167171 | 212316 |
| LL | 148164 | 174206 | 97101 | 135150 | 163171 | 312316 |
| LL | 132170 | 170218 | 93121 | 105171 | 155195 | 168316 |
| LL | 144182 | 174198 | 101101 | 110175 | 167195 | 219320 |
| LL | 148176 | 174174 | 105117 | 119179 | 151151 | 180324 |
| LL | 144172 | 190218 | 113125 | 126175 | 167191 | 284324 |
| LL | 152216 | 178182 | 113117 | 162166 | 159179 | 192328 |
| LL | 152180 | 182202 | 101117 | 114155 | 155187 | 220332 |
| LL | 144160 | 186194 | 117121 | 110122 | 155167 | 218391 |
| 2004LL | 164206 | 166186 | 109117 | 172176 | 127147 | 160180 |
| 2004LL | 168188 | 186190 | 105109 | 130134 | 139171 | 180184 |
| 2004LL | 162178 | 182186 | 105109 | 129155 | 159199 | 192192 |
| 2004LL | 164218 | 178186 | 109145 | 156156 | 131187 | 172204 |
| 2004LL | 148160 | 162174 | 101101 | 109130 | 167183 | 196208 |
| 2004LL | 160160 | 178198 | 109117 | 125148 | 139163 | 208212 |
| 2004LL | 152174 | 154186 | 121133 | 113200 | 155167 | 176212 |
| 2004LL | 174196 | 170174 | 109121 | 105168 | 167171 | 184212 |
| 2004LL | 136156 | 186202 | 105113 | 108126 | 167179 | 188212 |
| 2004LL | 164168 | 174190 | 105113 | 116168 | 127151 | 216216 |
| 2004LL | 160202 | 178202 | 113113 | 125145 | 155159 | 216216 |
| 2004LL | 172176 | 170174 | 109117 | 126178 | 151171 | 208216 |
| 2004LL | 148166 | 170202 | 105121 | 139147 | 127183 | 180216 |
| 2004LL | 116188 | 186190 | 113113 | 188196 | 131179 | 218218 |
| 2004LL | 160238 | 190210 | 109125 | 113156 | 155159 | 220220 |
| 2004LL | 152198 | 178198 | 105129 | 94172 | 151175 | 200224 |
| 2004LL | 152188 | 178190 | 125125 | 144152 | 151155 | 180225 |
| 2004LL | 152156 | 182206 | 105109 | 134146 | 155187 | 208226 |
| 2004LL | 152152 | 162166 | 109109 | 113139 | 163183 | 188228 |
| 2004LL | 164214 | 178186 | 101105 | 126148 | 171191 | 208228 |
| 2004LL | 152202 | 190198 | 101113 | 109109 | 163199 | 224228 |
| 2004LL | 168168 | 186186 | 89133 | 117144 | 155159 | 200232 |
| 2004LL | 120218 | 182198 | 105121 | 122130 | 159159 | 200232 |
| 2004LL | 152160 | 186194 | 109109 | 101117 | 155159 | 215232 |
| 2004LL | 218218 | 210230 | 109149 | 101168 | 151179 | 216232 |
| 2004LL | 198218 | 206214 | 117129 | 130134 | 147151 | 232236 |
| 2004LL | 174194 | 186198 | 109113 | 121200 | 155171 | 228236 |
| 2004LL | 176194 | 190190 | 105109 | 133228 | 151175 | 204236 |
| 2004LL | 156202 | 178190 | 109121 | 113148 | 135199 | 184236 |
| 2004LL | 128152 | 170210 | 113117 | 117120 | 171215 | 236236 |
| 2004LL | 148201 | 158190 | 113117 | 138164 | 147179 | 204240 |
| 2004LL | 148194 | 178214 | 109121 | 117170 | 151159 | 188244 |
| 2004LL | 162214 | 178198 | 101109 | 121160 | 159183 | 226244 |
| 2004LL | 156174 | 190198 | 113121 | 109142 | 159183 | 220246 |
| 2004LL | 188202 | 170194 | 109109 | 168172 | 151167 | 180248 |
| 2004LL | 116152 | 182214 | 109121 | 101160 | 151171 | 236248 |
| 2004LL | 148176 | 162182 | 113117 | 134134 | 155191 | 212248 |
| 2004LL | 164314 | 158214 | 109125 | 142156 | 191211 | 248248 |
| 2004LL | 152168 | 194198 | 113141 | 109143 | 163171 | 232252 |
| 2004LL | 154190 | 170194 | 101121 | 142196 | 183187 | 228252 |
| 2004LL | 128180 | 170206 | 109121 | 125144 | 151159 | 234254 |
| 2004LL | 156182 | 194234 | 101121 | 172172 | 163171 | 192256 |
| 2004LL | 172206 | 178186 | 97117 | 100140 | 151183 | 224256 |
| 2004LL | 148160 | 194210 | 97109 | 135186 | 115191 | 226258 |
| 2004LL | 148180 | 170198 | 113129 | 140156 | 159163 | 180264 |
| 2004LL | 136160 | 166174 | 113113 | 152152 | 187215 | 232264 |
| 2004LL | 194202 | 158166 | 109117 | 117156 | 139195 | 240266 |
| 2004LL | 144170 | 178190 | 101113 | 121148 | 139171 | 204268 |
| 2004LL | 160160 | 158174 | 105121 | 147152 | 199215 | 220268 |
| 2004LL | 144214 | 186190 | 101109 | 97148 | 151167 | 264272 |
| 2004LL | 160176 | 178182 | 97121 | 113113 | 159191 | 168272 |
| 2004LL | 168184 | 194198 | 109113 | 146156 | 155159 | 184280 |
| 2004LL | 136168 | 170182 | 105109 | 125126 | 159175 | 228280 |
| 2004LL | 152164 | 182182 | 117125 | 104164 | 167183 | 280284 |
| 2004LL | 172184 | 198214 | 113121 | 130130 | 159159 | 240288 |
| 2004LL | 136160 | 174186 | 105109 | 117184 | 159171 | 212288 |
| 2004LL | 182194 | 170178 | 113113 | 124128 | 155199 | 248288 |
| 2004LL | 116172 | 166214 | 105125 | 132166 | 187207 | 284288 |
| 2004LL | 128174 | 186194 | 117117 | 130166 | 143155 | 180292 |
| 2004LL | 170172 | 182206 | 113113 | 140144 | 155159 | 232292 |
| 2004LL | 144148 | 162178 | 117125 | 146160 | 167175 | 288292 |
| 2004LL | 136178 | 170190 | 113117 | 101168 | 151187 | 236292 |
| 2004LL | 143160 | 170174 | 105121 | 121144 | 179207 | 220292 |
| 2004LL | 136164 | 158174 | 101129 | 134134 | 171215 | 220292 |
| 2004LL | 155194 | 186186 | 105117 | 164184 | 131143 | 244296 |
| 2004LL | 148160 | 194194 | 107113 | 109109 | 131155 | 208296 |
| 2004LL | 116180 | 158178 | 117129 | 176184 | 151171 | 160296 |
| 2004LL | 156202 | 182182 | 109113 | 105176 | 183183 | 292296 |
| 2004LL | 148156 | 158202 | 109133 | 116145 | 147191 | 220296 |
| 2004LL | 140242 | 190198 | 105113 | 105152 | 159191 | 288296 |
| 2004LL | 148202 | 174178 | 121137 | 125160 | 135139 | 248300 |
| 2004LL | 156202 | 162162 | 113125 | 112152 | 151163 | 260300 |
| 2004LL | 210222 | 170178 | 105117 | 117121 | 171175 | 232300 |
| 2004LL | 144172 | 162210 | 113113 | 140156 | 171195 | 156300 |
| 2004LL | 156190 | 170174 | 109113 | 170228 | 159207 | 234300 |
| 2004LL | 160206 | 170186 | 101113 | 109113 | 131131 | 300304 |
| 2004LL | 144176 | 174182 | 121121 | 117156 | 131135 | 280304 |
| 2004LL | 160178 | 162174 | 109113 | 109200 | 147167 | 300304 |
| 2004LL | 116116 | 194202 | 105113 | 117180 | 167175 | 216304 |
| 2004LL | 152152 | 158158 | 109109 | 108112 | 175187 | 292304 |
| 2004LL | 188226 | 190198 | 117117 | 152188 | 155199 | 232304 |
| 2004LL | 140156 | 166170 | 105121 | 104191 | 135159 | 192308 |
| 2004LL | 132192 | 186198 | 113117 | 184200 | 147159 | 211308 |
| 2004LL | 168180 | 178222 | 109125 | 108125 | 123163 | 208308 |
| 2004LL | 152212 | 190198 | 113121 | 170196 | 159171 | 212308 |
| 2004LL | 140172 | 162186 | 101113 | 126198 | 191191 | 172308 |
| 2004LL | 156186 | 170202 | 101109 | 152156 | 123151 | 196312 |
| 2004LL | 168184 | 174198 | 125125 | 113140 | 123151 | 256312 |
| 2004LL | 198222 | 162178 | 117125 | 104108 | 147151 | 312316 |
| 2004LL | 164214 | 162182 | 101105 | 108152 | 135139 | 204320 |
| 2004LL | 140206 | 178182 | 113129 | 108109 | 211255 | 228320 |
| 2004LL | 140156 | 178210 | 105117 | 109140 | 147159 | 220324 |
| 2004LL | 140156 | 198230 | 101101 | 144186 | 143183 | 212324 |
| 2004LL | 168188 | 182206 | 113133 | 139148 | 139159 | 236328 |
| 2004LL | 120176 | 174198 | 105113 | 125142 | 175175 | 296332 |
| 2004LL | 166168 | 178186 | 109113 | 170170 | 135195 | 240376 |
| 2004LL | 168198 | 174182 | 109133 | 140180 | 167171 | 240380 |
| RBB | 176190 | 186186 | 109109 | 128132 | 147147 | 168172 |
| RBB | 136246 | 190214 | 109117 | 112112 | 139179 | 168172 |
| RBB | 152184 | 174178 | 121129 | 123123 | 159159 | 178178 |
| RBB | 148226 | 178190 | 109113 | 120147 | 143159 | 184184 |
| RBB | 136148 | 190190 | 109137 | 146170 | 159163 | 172184 |
| RBB | 158206 | 182190 | 121149 | 122168 | 187191 | 164186 |
| RBB | 82156 | 166190 | 101113 | 144148 | 131163 | 208208 |
| RBB | 140148 | 198206 | 121133 | 113160 | 151183 | 192208 |
| RBB | 164206 | 186198 | 105113 | 117134 | 179183 | 212212 |
| RBB | 130218 | 186210 | 129137 | 129138 | 171203 | 216216 |
| RBB | 120190 | 182210 | 117129 | 112232 | 135147 | 216220 |
| RBB | 180222 | 178178 | 105113 | 138152 | 147175 | 192220 |
| RBB | 148206 | 174178 | 101109 | 138155 | 147175 | 208220 |
| RBB | 144148 | 170182 | 105113 | 152156 | 163179 | 188220 |
| RBB | 156168 | 162186 | 105105 | 88162 | 183183 | 216220 |
| RBB | 116152 | 194202 | 105113 | 113152 | 143179 | 218223 |
| RBB | 156176 | 166190 | 113121 | 144172 | 131147 | 212224 |
| RBB | 152186 | 190214 | 105109 | 148196 | 151163 | 215224 |
| RBB | 168202 | 202222 | 109117 | 113142 | 143175 | 176224 |
| RBB | 152172 | 186190 | 97117 | 136164 | 139195 | 200224 |
| RBB | 128148 | 166178 | 101109 | 117130 | 159167 | 192228 |
| RBB | 156188 | 178198 | 101105 | 113178 | 147187 | 176228 |
| RBB | 148176 | 186198 | 113125 | 128152 | 155195 | 212228 |
| RBB | 168192 | 166198 | 101117 | 130164 | 131147 | 228232 |
| RBB | 176210 | 186186 | 105125 | 140155 | 139167 | 208232 |
| RBB | 156168 | 162182 | 113129 | 134154 | 163171 | 228232 |
| RBB | 132202 | 158170 | 105117 | 144152 | 163183 | 220232 |
| RBB | 116164 | 166190 | 117117 | 130160 | 155191 | 232232 |
| RBB | 140214 | 174202 | 113113 | 109109 | 171171 | 164238 |
| RBB | 164196 | 154194 | 109117 | 110168 | 163187 | 188238 |
| RBB | 132152 | 154182 | 97109 | 134142 | 155155 | 232240 |
| RBB | 148164 | 166178 | 117121 | 130146 | 131147 | 244244 |
| RBB | 140168 | 206206 | 125125 | 117130 | 143171 | 196244 |
| RBB | 136202 | 158174 | 109109 | 108166 | 159199 | 216244 |
| RBB | 176176 | 194226 | 125125 | 109117 | 139167 | 228248 |
| RBB | 116160 | 178206 | 101117 | 121134 | 175191 | 228248 |
| RBB | 152164 | 190218 | 101105 | 104122 | 163175 | 220250 |
| RBB | 190190 | 166194 | 105113 | 113160 | 139191 | 240256 |
| RBB | 190210 | 174174 | 105109 | 100120 | 171203 | 176260 |
| RBB | 136176 | 162182 | 101109 | 109134 | 123183 | 216262 |
| RBB | 152176 | 174206 | 109121 | 138164 | 143147 | 208264 |
| RBB | 152160 | 194202 | 105129 | 125125 | 143155 | 172264 |
| RBB | 140144 | 186198 | 121121 | 104159 | 155175 | 256264 |
| RBB | 168194 | 170182 | 105109 | 109116 | 123167 | 180268 |
| RBB | 156164 | 162186 | 93125 | 104134 | 147179 | 184268 |
| RBB | 144194 | 186186 | 109125 | 121156 | 131199 | 260268 |
| RBB | 128164 | 186198 | 109125 | 138172 | 191191 | 228270 |
| RBB | 160210 | 190206 | 97101 | 126178 | 139155 | 168276 |
| RBB | 174198 | 170174 | 113113 | 130144 | 159183 | 229276 |
| RBB | 160198 | 206210 | 113121 | 113190 | 171183 | 264276 |
| RBB | 170170 | 194206 | 117117 | 121132 | 151163 | 192280 |
| RBB | 144148 | 162210 | 101117 | 130147 | 171179 | 234280 |
| RBB | 164164 | 190202 | 97109 | 113122 | 179195 | 160280 |
| RBB | 162218 | 166174 | 109121 | 151160 | 147195 | 232284 |
| RBB | 156174 | 170178 | 113121 | 108134 | 155159 | 160288 |
| RBB | 140206 | 182190 | 101117 | 134176 | 147171 | 264288 |
| RBB | 135156 | 166182 | 109113 | 134168 | 155175 | 208288 |
| RBB | 164168 | 190194 | 109117 | 126148 | 163175 | 234288 |
| RBB | 148164 | 182186 | 109121 | 97164 | 159187 | 216288 |
| RBB | 218222 | 158202 | 113113 | 144148 | 187195 | 226288 |
| RBB | 148258 | 170186 | 109129 | 116152 | 155163 | 224292 |
| RBB | 164218 | 182194 | 109121 | 142200 | 123207 | 228292 |
| RBB | 206206 | 186198 | 105109 | 102172 | 131147 | 252296 |
| RBB | 174186 | 162182 | 113129 | 117156 | 151159 | 216296 |
| RBB | 152172 | 202250 | 101113 | 113171 | 159171 | 272296 |
| RBB | 164180 | 166170 | 117137 | 129180 | 127183 | 228296 |
| RBB | 160160 | 186190 | 109121 | 113159 | 183183 | 268296 |
| RBB | 152172 | 170218 | 105105 | 124180 | 155183 | 274296 |
| RBB | 135164 | 170174 | 105113 | 106109 | 151155 | 300300 |
| RBB | 152190 | 170202 | 101113 | 113121 | 147167 | 192300 |
| RBB | 144160 | 166170 | 113125 | 126130 | 159183 | 180300 |
| RBB | 198198 | 182186 | 117121 | 126153 | 171183 | 216300 |
| RBB | 166168 | 170186 | 113125 | 146160 | 195199 | 224300 |
| RBB | 132170 | 174174 | 105117 | 148148 | 131187 | 240302 |
| RBB | 148160 | 166202 | 117117 | 100104 | 139155 | 225304 |
| RBB | 148148 | 170206 | 105109 | 144160 | 143159 | 176304 |
| RBB | 148152 | 206206 | 97113 | 112159 | 139159 | 292304 |
| RBB | 147156 | 178178 | 109113 | 146159 | 131175 | 172304 |
| RBB | 116160 | 170186 | 109137 | 121176 | 163175 | 282304 |
| RBB | 168184 | 174194 | 109117 | 136171 | 163191 | 300304 |
| RBB | 170202 | 166214 | 125125 | 109121 | 163163 | 224308 |
| RBB | 152164 | 162214 | 105109 | 139151 | 151175 | 180308 |
| RBB | 148172 | 170178 | 101117 | 104112 | 143179 | 180308 |
| RBB | 148218 | 158178 | 109113 | 126130 | 159163 | 308312 |
| RBB | 156168 | 162190 | 97105 | 142180 | 155167 | 168312 |
| RBB | 132172 | 162166 | 109113 | 168172 | 151151 | 220316 |
| RBB | 120188 | 182202 | 121129 | 132148 | 139167 | 208316 |
| RBB | 160176 | 170198 | 117133 | 117156 | 167171 | 300320 |
| RBB | 170172 | 174178 | 113121 | 108144 | 155191 | 240320 |
| RBB | 152152 | 190198 | 105113 | 120176 | 155183 | 224324 |
| RBB | 116135 | 170194 | 109125 | 134148 | 187199 | 292324 |
| RBB | 144202 | 174186 | 117133 | 112180 | 159159 | 227332 |
| RBB | 132202 | 170190 | 109121 | 156218 | 175179 | 328336 |
| RBB | 152156 | 178206 | 109121 | 164186 | 119167 | 288340 |
| RBB | 116151 | 186190 | 105125 | 126130 | 163171 | 224384 |
| RBB | 140152 | 166182 | 101109 | 117164 | 167191 | 260384 |
| RBB | 176180 | 190210 | 109109 | 104117 | 183199 | 232384 |
| RBB | 156242 | 178186 | 105109 | 108179 | 183207 | 196384 |
| RBB | 148176 | 182190 | 125129 | 117168 | 123167 | 164396 |
| RBB | 156166 | 158206 | 109117 | 147155 | 127159 | 172188 |
| RBB | 116198 | 194218 | 137137 | 119139 | 159175 | 188192 |
| RBB | 152196 | 166190 | 109121 | 107141 | 151151 | 178194 |
| RBB | 168180 | 166182 | 117133 | 111127 | 151155 | 196200 |
| RBB | 172195 | 166218 | 105113 | 103115 | 159159 | 188200 |
| RBB | 172196 | 182190 | 113125 | 147155 | 151151 | 168208 |
| RBB | 160184 | 178194 | 109109 | 91132 | 159167 | 200208 |
| RBB | 196200 | 174174 | 101109 | 142158 | 151155 | 178212 |
| RBB | 160186 | 174206 | 109121 | 121179 | 143163 | 200212 |
| RBB | 152182 | 170178 | 105121 | 119161 | 151171 | 184212 |
| RBB | 200224 | 174194 | 109113 | 120186 | 151175 | 212212 |
| RBB | 84196 | 178186 | 117133 | 110187 | 123163 | 212216 |
| RBB | 140148 | 170198 | 117121 | 118134 | 143171 | 176216 |
| RBB | 214228 | 174194 | 105137 | 127143 | 163179 | 212216 |
| RBB | 164200 | 178194 | 109117 | 115159 | 163187 | 204216 |
| RBB | 144192 | 162162 | 109109 | 103180 | 183187 | 216216 |
| RBB | 160173 | 174206 | 117117 | 123175 | 151171 | 188218 |
| RBB | 140192 | 166178 | 109117 | 147147 | 143171 | 216220 |
| RBB | 152152 | 190194 | 109121 | 139171 | 175203 | 188224 |
| RBB | 140176 | 194194 | 125133 | 158163 | 155171 | 172226 |
| RBB | 128208 | 170170 | 105117 | 118131 | 163175 | 172226 |
| RBB | 140224 | 178190 | 113117 | 115119 | 143147 | 182228 |
| RBB | 116148 | 178194 | 109113 | 127158 | 147167 | 160228 |
| RBB | 144192 | 182210 | 109125 | 106126 | 143183 | 230230 |
| RBB | 156164 | 198210 | 117125 | 106162 | 115147 | 216232 |
| RBB | 152188 | 162166 | 105109 | 106147 | 147179 | 176232 |
| RBB | 144176 | 194206 | 101109 | 175175 | 179227 | 225232 |
| RBB | 132212 | 190198 | 117125 | 107159 | 159167 | 188233 |
| RBB | 186256 | 178226 | 109121 | 181196 | 167179 | 206233 |
| RBB | 164168 | 162174 | 113125 | 103139 | 179183 | 180234 |
| RBB | 140168 | 214214 | 109117 | 137176 | 155187 | 190235 |
| RBB | 132168 | 190202 | 97109 | 130208 | 147179 | 164236 |
| RBB | 156178 | 170174 | 113129 | 110175 | 175179 | 216236 |
| RBB | 168218 | 186202 | 109117 | 123132 | 135151 | 216238 |
| RBB | 152192 | 178182 | 97109 | 103128 | 135179 | 224242 |
| RBB | 140160 | 170174 | 117121 | 154157 | 123187 | 200242 |
| RBB | 152180 | 166206 | 113117 | 150153 | 135155 | 160244 |
| RBB | 170170 | 170198 | 109133 | 123162 | 151171 | 170244 |
| RBB | 220298 | 182206 | 101109 | 155176 | 155159 | 192248 |
| RBB | 116152 | 178202 | 101113 | 150190 | 167179 | 243248 |
| RBB | 174182 | 174190 | 109113 | 122130 | 167167 | 192250 |
| RBB | 144168 | 182198 | 93117 | 119138 | 159175 | 230250 |
| RBB | 166168 | 162174 | 101133 | 103175 | 155159 | 244252 |
| RBB | 172188 | 178186 | 109109 | 150158 | 151171 | 228254 |
| RBB | 144214 | 162198 | 121133 | 218218 | 163179 | 214256 |
| RBB | 144194 | 174206 | 109113 | 131162 | 151151 | 246258 |
| RBB | 174206 | 170214 | 109129 | 99118 | 151163 | 208260 |
| RBB | 176192 | 158182 | 101109 | 127150 | 159171 | 216264 |
| RBB | 160236 | 194206 | 113125 | 123154 | 155171 | 232264 |
| RBB | 116160 | 198218 | 101121 | 131185 | 139195 | 224264 |
| RBB | 148158 | 198214 | 105121 | 127150 | 143171 | 170268 |
| RBB | 144160 | 182206 | 109113 | 151154 | 167187 | 188268 |
| RBB | 148160 | 186194 | 101113 | 115123 | 143163 | 216272 |
| RBB | 160178 | 166166 | 109113 | 106154 | 127131 | 180280 |
| RBB | 148196 | 178194 | 105121 | 154158 | 159167 | 239280 |
| RBB | 136160 | 178190 | 109129 | 142143 | 151199 | 218280 |
| RBB | 152164 | 174194 | 113121 | 118142 | 179215 | 280280 |
| RBB | 144168 | 178182 | 109121 | 120176 | 175199 | 274284 |
| RBB | 172191 | 178182 | 117125 | 123194 | 147167 | 280288 |
| RBB | 156188 | 174214 | 105109 | 127142 | 159171 | 222288 |
| RBB | 152160 | 194202 | 109125 | 103158 | 155203 | 224288 |
| RBB | 116152 | 174202 | 101105 | 134139 | 187207 | 268288 |
| RBB | 156160 | 170186 | 109121 | 99176 | 171171 | 256292 |
| RBB | 152204 | 178186 | 105113 | 138146 | 167203 | 246292 |
| RBB | 144198 | 182194 | 109117 | 135147 | 171175 | 216296 |
| RBB | 136144 | 182214 | 109121 | 126126 | 175187 | 162296 |
| RBB | 80212 | 182186 | 105117 | 102130 | 139203 | 229298 |
| RBB | 156172 | 170190 | 109117 | 110163 | 175183 | 180300 |
| RBB | 140166 | 198198 | 109133 | 109117 | 163187 | 160300 |
| RBB | 116192 | 162170 | 101105 | 136168 | 175195 | 240300 |
| RBB | 156172 | 182186 | 109113 | 134141 | 155211 | 296300 |
| RBB | 156220 | 162210 | 101109 | 115178 | 203223 | 272300 |
| RBB | 164176 | 194194 | 101125 | 91107 | 147159 | 224304 |
| RBB | 116164 | 170198 | 113113 | 176184 | 167171 | 300304 |
| RBB | 148222 | 166174 | 105137 | 128140 | 167183 | 246304 |
| RBB | 116194 | 162198 | 109113 | 107175 | 139143 | 270308 |
| RBB | 168180 | 158162 | 113129 | 157172 | 159183 | 224308 |
| RBB | 116164 | 162182 | 117117 | 119150 | 187187 | 216308 |
| RBB | 144164 | 162170 | 109109 | 124124 | 135191 | 264308 |
| RBB | 164232 | 174190 | 109133 | 141184 | 159227 | 216308 |
| RBB | 116148 | 166170 | 105117 | 142147 | 167171 | 268312 |
| RBB | 116168 | 166174 | 105105 | 145174 | 159183 | 280312 |
| RBB | 158178 | 158162 | 101109 | 115151 | 195223 | 300312 |
| RBB | 152228 | 170186 | 101113 | 123174 | 151183 | 272316 |
| RBB | 160176 | 186190 | 105117 | 143171 | 147147 | 296324 |
| RBB | 152192 | 182210 | 113113 | 137150 | 155227 | 280328 |
| RBB | 116168 | 166170 | 101109 | 187187 | 123159 | 268388 |
| SES | 140144 | 154202 | 121125 | 143151 | 135175 | 160160 |
| SES | 135151 | 174182 | 105109 | 123147 | 167171 | 160172 |
| SES | 166216 | 170190 | 113117 | 164173 | 163191 | 160172 |
| SES | 168190 | 194198 | 113113 | 115135 | 143143 | 162180 |
| SES | 148188 | 178254 | 109117 | 115171 | 151159 | 160188 |
| SES | 212238 | 174178 | 101101 | 139139 | 163203 | 160188 |
| SES | 116206 | 174182 | 109109 | 111161 | 163195 | 166190 |
| SES | 144156 | 158198 | 117129 | 150180 | 123147 | 192192 |
| SES | 172226 | 174194 | 109113 | 164168 | 163187 | 192192 |
| SES | 140186 | 170190 | 89113 | 151151 | 139171 | 188196 |
| SES | 160204 | 182182 | 105109 | 118143 | 123167 | 192200 |
| SES | 84195 | 162174 | 105121 | 134138 | 147147 | 204204 |
| SES | 148168 | 198202 | 101121 | 105139 | 167167 | 192204 |
| SES | 160164 | 170182 | 109129 | 140152 | 159171 | 204204 |
| SES | 200224 | 178194 | 105121 | 127159 | 139163 | 160212 |
| SES | 155168 | 166210 | 105129 | 111115 | 147187 | 212212 |
| SES | 148194 | 174210 | 97121 | 127163 | 151199 | 188212 |
| SES | 148160 | 166178 | 101105 | 127130 | 131179 | 180216 |
| SES | 200204 | 182186 | 117121 | 143147 | 163195 | 168216 |
| SES | 116180 | 178182 | 105105 | 136151 | 183195 | 184216 |
| SES | 80156 | 194222 | 105113 | 123178 | 163179 | 160217 |
| SES | 156176 | 174190 | 109109 | 112131 | 139171 | 192218 |
| SES | 136152 | 170186 | 105129 | 131135 | 119139 | 172220 |
| SES | 204212 | 198222 | 109129 | 110123 | 143159 | 212220 |
| SES | 174230 | 170170 | 113117 | 127132 | 183183 | 200221 |
| SES | 159212 | 198234 | 109129 | 131139 | 147147 | 208224 |
| SES | 188224 | 182202 | 109121 | 115146 | 131187 | 216224 |
| SES | 148172 | 194210 | 125125 | 101172 | 187215 | 214224 |
| SES | 198198 | 170182 | 113129 | 112136 | 151175 | 184228 |
| SES | 152180 | 158166 | 109137 | 98180 | 155187 | 196228 |
| SES | 140152 | 162178 | 121133 | 117139 | 163191 | 208228 |
| SES | 156172 | 174198 | 109109 | 127147 | 151167 | 226229 |
| SES | 116132 | 170194 | 101117 | 160234 | 163167 | 229232 |
| SES | 152174 | 162178 | 105121 | 111173 | 175179 | 172232 |
| SES | 177195 | 166178 | 121125 | 124155 | 155195 | 192232 |
| SES | 156202 | 166174 | 109117 | 110110 | 135171 | 226234 |
| SES | 160168 | 170182 | 109117 | 152174 | 159163 | 216238 |
| SES | 144144 | 182182 | 109113 | 140156 | 163167 | 184238 |
| SES | 152152 | 182202 | 101105 | 122138 | 155191 | 208238 |
| SES | 148164 | 170190 | 105109 | 111143 | 187187 | 211242 |
| SES | 172176 | 186190 | 89109 | 116127 | 159163 | 208244 |
| SES | 136228 | 166190 | 117117 | 136151 | 187191 | 232244 |
| SES | 218244 | 174194 | 109129 | 119138 | 139147 | 236248 |
| SES | 154156 | 194194 | 101133 | 150155 | 127171 | 204248 |
| SES | 152188 | 162198 | 113125 | 112127 | 139199 | 248248 |
| SES | 144144 | 178210 | 117121 | 127180 | 139159 | 234250 |
| SES | 192200 | 162166 | 109129 | 115115 | 147215 | 176250 |
| SES | 144164 | 166170 | 121129 | 120168 | 139139 | 220252 |
| SES | 156172 | 182190 | 109137 | 136155 | 139163 | 230252 |
| SES | 148160 | 162186 | 109133 | 131135 | 147183 | 242254 |
| SES | 120152 | 174174 | 105117 | 139144 | 195215 | 214258 |
| SES | 136140 | 166218 | 117117 | 143214 | 119127 | 184260 |
| SES | 144190 | 174186 | 113129 | 139168 | 151159 | 264264 |
| SES | 144168 | 186194 | 105121 | 114122 | 155175 | 184264 |
| SES | 143166 | 178194 | 101117 | 108116 | 159183 | 256264 |
| SES | 167182 | 166174 | 113117 | 137165 | 163199 | 212264 |
| SES | 160168 | 186214 | 113129 | 127143 | 171175 | 160266 |
| SES | 148168 | 170174 | 113121 | 132132 | 139175 | 264268 |
| SES | 116172 | 174190 | 101109 | 123142 | 155183 | 236268 |
| SES | 192210 | 158174 | 101109 | 115118 | 159159 | 212272 |
| SES | 164180 | 182194 | 105117 | 112120 | 143163 | 184272 |
| SES | 160168 | 178182 | 97113 | 127164 | 167171 | 220274 |
| SES | 148202 | 158166 | 109121 | 132137 | 123159 | 209276 |
| SES | 148156 | 182198 | 97133 | 123142 | 155163 | 212280 |
| SES | 160194 | 194198 | 109113 | 136145 | 151171 | 264280 |
| SES | 156200 | 166214 | 117125 | 168198 | 147195 | 230284 |
| SES | 156186 | 170186 | 109125 | 101142 | 139171 | 282286 |
| SES | 152186 | 166198 | 117129 | 151155 | 135139 | 230288 |
| SES | 148160 | 190190 | 105117 | 116142 | 139179 | 268288 |
| SES | 190214 | 170186 | 109141 | 115131 | 123203 | 238288 |
| SES | 160168 | 174182 | 105113 | 102139 | 123203 | 256288 |
| SES | 148152 | 178182 | 105109 | 117155 | 127143 | 220292 |
| SES | 156178 | 170178 | 113133 | 107189 | 127143 | 280292 |
| SES | 148228 | 182190 | 121125 | 120140 | 155167 | 217292 |
| SES | 148170 | 174194 | 121125 | 105117 | 159175 | 168292 |
| SES | 148160 | 178182 | 105125 | 127140 | 171179 | 168292 |
| SES | 152152 | 166186 | 105109 | 147172 | 135195 | 248292 |
| SES | 148206 | 182190 | 117117 | 131139 | 131147 | 296296 |
| SES | 172188 | 170206 | 117129 | 132132 | 155159 | 292296 |
| SES | 148172 | 178178 | 113117 | 118156 | 163179 | 170296 |
| SES | 132172 | 166186 | 101113 | 102123 | 135179 | 180296 |
| SES | 160176 | 202202 | 101117 | 127147 | 131179 | 288300 |
| SES | 128195 | 166202 | 121125 | 147177 | 139179 | 300300 |
| SES | 212216 | 170182 | 109109 | 110151 | 155223 | 272300 |
| SES | 158174 | 162186 | 109121 | 111142 | 155167 | 268304 |
| SES | 160160 | 162174 | 109121 | 107135 | 153175 | 244304 |
| SES | 136176 | 182190 | 105125 | 105112 | 139155 | 300308 |
| SES | 164182 | 162162 | 105125 | 148159 | 155159 | 268308 |
| SES | 172214 | 162186 | 109117 | 103122 | 163175 | 209308 |
| SES | 144148 | 194194 | 117121 | 115131 | 155163 | 212316 |
| SES | 156208 | 166170 | 113117 | 136180 | 135175 | 226316 |
| SES | 136172 | 166206 | 105117 | 119177 | 143151 | 288320 |
| SES | 144156 | 162198 | 105109 | 123187 | 135151 | 188324 |
| SES | 84152 | 166198 | 101117 | 115147 | 159191 | 184324 |
| SES | 128202 | 166186 | 101113 | 143173 | 131195 | 232324 |
| SES | 184194 | 194202 | 105113 | 134139 | 147199 | 196328 |
| SES | 160196 | 194198 | 117121 | 147155 | 147159 | 306334 |
| SES | 148208 | 186194 | 109113 | 160207 | 123207 | 300384 |
| SES | 164192 | 170226 | 113121 | 127147 | 163195 | 284392 |
| SES | 152156 | 162214 | 113121 | 143143 | 131135 | 204394 |
| SES | 165224 | 178190 | 109133 | 142143 | 115179 | 240394 |
| SES | 136156 | 174194 | 109121 | 127159 | 147163 | 160168 |
| SES | 182196 | 166182 | 101109 | 110200 | 147171 | 172172 |
| SES | 160168 | 178190 | 105129 | 133137 | 131171 | 168184 |
| SES | 168168 | 162190 | 109117 | 123123 | 167171 | 196196 |
| SES | 148164 | 178226 | 101113 | 106155 | 127191 | 196204 |
| SES | 164168 | 162194 | 105109 | 123138 | 139159 | 184208 |
| SES | 168204 | 186194 | 105109 | 110122 | 175231 | 200212 |
| SES | 136156 | 160226 | 97101 | 151166 | 119151 | 212216 |
| SES | 124186 | 166190 | 101113 | 127138 | 127187 | 212218 |
| SES | 148168 | 162214 | 109109 | 119119 | 131159 | 208220 |
| SES | 160178 | 162174 | 105105 | 118119 | 139171 | 220220 |
| SES | 148152 | 162186 | 109117 | 134187 | 147183 | 216220 |
| SES | 188258 | 178190 | 101109 | 106167 | 135203 | 215224 |
| SES | 144164 | 194210 | 113121 | 143143 | 131139 | 184228 |
| SES | 148210 | 178182 | 109125 | 101162 | 167171 | 212228 |
| SES | 152178 | 174198 | 113117 | 118134 | 139187 | 200228 |
| SES | 116176 | 170218 | 109121 | 123141 | 171179 | 188230 |
| SES | 186188 | 162210 | 105105 | 126183 | 143191 | 200230 |
| SES | 116182 | 190198 | 109117 | 127135 | 147199 | 164230 |
| SES | 116176 | 174182 | 109117 | 130141 | 159179 | 208231 |
| SES | 172212 | 178190 | 101109 | 110138 | 143159 | 212232 |
| SES | 140164 | 174210 | 97109 | 139168 | 143167 | 212234 |
| SES | 152160 | 178198 | 117129 | 156174 | 151179 | 208234 |
| SES | 152156 | 194218 | 117125 | 167170 | 183195 | 236236 |
| SES | 144170 | 174182 | 109113 | 110151 | 127171 | 212238 |
| SES | 136148 | 178194 | 109113 | 119122 | 131183 | 184245 |
| SES | 152180 | 166198 | 113117 | 106119 | 163163 | 244248 |
| SES | 152160 | 186198 | 105113 | 111126 | 151171 | 208248 |
| SES | 140164 | 190198 | 121129 | 158179 | 171179 | 164253 |
| SES | 116194 | 166178 | 113125 | 126212 | 191227 | 229256 |
| SES | 160164 | 186186 | 113129 | 115132 | 163175 | 184258 |
| SES | 112148 | 174214 | 113121 | 119141 | 151167 | 188260 |
| SES | 160184 | 162166 | 117125 | 111126 | 123195 | 264264 |
| SES | 174174 | 174190 | 109125 | 138138 | 159159 | 160268 |
| SES | 132176 | 170190 | 109129 | 106119 | 139163 | 172276 |
| SES | 148152 | 162198 | 109121 | 133222 | 171171 | 250276 |
| SES | 136156 | 162182 | 121125 | 110122 | 151155 | 228280 |
| SES | 144196 | 198218 | 113113 | 167230 | 159175 | 234280 |
| SES | 168220 | 170190 | 105117 | 138163 | 155187 | 242280 |
| SES | 120190 | 158202 | 113117 | 122130 | 139167 | 200284 |
| SES | 128182 | 166190 | 105109 | 131181 | 143167 | 234284 |
| SES | 136204 | 178178 | 105137 | 105133 | 163175 | 172284 |
| SES | 164232 | 162186 | 121129 | 119126 | 147175 | 250284 |
| SES | 128140 | 170198 | 105121 | 126142 | 151183 | 246284 |
| SES | 116162 | 178198 | 105113 | 158172 | 143175 | 212288 |
| SES | 168200 | 194202 | 105117 | 105166 | 123163 | 156292 |
| SES | 140188 | 178202 | 109129 | 119148 | 139143 | 219296 |
| SES | 152160 | 186202 | 117121 | 110170 | 143147 | 260296 |
| SES | 160164 | 170182 | 105113 | 114163 | 151187 | 296300 |
| SES | 132212 | 186194 | 109121 | 133147 | 151155 | 284304 |
| SES | 166172 | 194210 | 101101 | 138159 | 143163 | 208304 |
| SES | 160190 | 178190 | 125125 | 102119 | 151179 | 228304 |
| SES | 164180 | 162186 | 105109 | 131155 | 191211 | 260304 |
| SES | 156212 | 162182 | 129149 | 153187 | 123159 | 160308 |
| SES | 144164 | 166186 | 101121 | 127142 | 159183 | 284312 |
| SES | 148236 | 178198 | 113117 | 118135 | 131207 | 200312 |
| SES | 160200 | 162190 | 109113 | 147159 | 163171 | 280316 |
| SES | 140156 | 178202 | 109109 | 110127 | 143159 | 256324 |
| SES | 116172 | 186186 | 109109 | 119123 | 163175 | 304324 |
| SES | 128148 | 166174 | 105121 | 122150 | 159163 | 336340 |
| SES | 144182 | 186194 | 101117 | 114114 | 123159 | 204344 |
| SES | 156224 | 166178 | 109137 | 122127 | 143155 | 208392 |
| SES | 144152 | 170182 | 101109 | 137175 | 167179 | 225392 |
| SES | 144160 | 162194 | 109125 | 122158 | 187187 | 300392 |
| SLL | 136144 | 162174 | 105121 | 116151 | 151159 | 160160 |
| SLL | 162196 | 190190 | 121137 | 203215 | 123179 | 168168 |
| SLL | 143144 | 162194 | 109113 | 127159 | 123147 | 176184 |
| SLL | 144178 | 178202 | 109153 | 150211 | 147151 | 180196 |
| SLL | 140162 | 186186 | 113125 | 96202 | 187187 | 184196 |
| SLL | 148152 | 178182 | 101109 | 126151 | 143207 | 184196 |
| SLL | 148190 | 162174 | 117117 | 151160 | 127183 | 184200 |
| SLL | 156226 | 182198 | 121129 | 119162 | 163203 | 204204 |
| SLL | 156168 | 166190 | 105121 | 118130 | 163175 | 208208 |
| SLL | 116124 | 158194 | 109129 | 103155 | 187199 | 176208 |
| SLL | 116116 | 198206 | 109117 | 102164 | 159167 | 212212 |
| SLL | 144204 | 182186 | 105129 | 113147 | 163179 | 204216 |
| SLL | 116171 | 194210 | 113121 | 127146 | 163175 | 180224 |
| SLL | 148182 | 190198 | 101121 | 110143 | 167187 | 204224 |
| SLL | 164168 | 190230 | 105117 | 108111 | 179195 | 208227 |
| SLL | 156156 | 162190 | 109125 | 102142 | 143155 | 201228 |
| SLL | 180198 | 198214 | 113117 | 139184 | 155175 | 228228 |
| SLL | 144152 | 170230 | 113117 | 144212 | 155155 | 176236 |
| SLL | 148214 | 170186 | 105117 | 114154 | 151159 | 176236 |
| SLL | 160167 | 170182 | 109117 | 151171 | 159163 | 216236 |
| SLL | 128152 | 190202 | 105113 | 141148 | 159195 | 230238 |
| SLL | 152168 | 178186 | 121125 | 115122 | 159167 | 214239 |
| SLL | 140224 | 174186 | 101105 | 102139 | 167171 | 242242 |
| SLL | 116164 | 162190 | 113117 | 102147 | 163175 | 190242 |
| SLL | 156156 | 182190 | 101121 | 143168 | 143183 | 244248 |
| SLL | 164172 | 166198 | 105117 | 134156 | 123187 | 246250 |
| SLL | 152208 | 174178 | 121133 | 127130 | 147159 | 160252 |
| SLL | 172204 | 178210 | 113129 | 116172 | 171219 | 235252 |
| SLL | 184188 | 186210 | 109129 | 130156 | 155171 | 204254 |
| SLL | 152172 | 170190 | 101141 | 131134 | 147159 | 188256 |
| SLL | 148166 | 186194 | 109109 | 131198 | 159183 | 200260 |
| SLL | 112160 | 166182 | 109125 | 129159 | 175187 | 230262 |
| SLL | 166168 | 170178 | 105121 | 119151 | 139147 | 229264 |
| SLL | 156190 | 174186 | 109125 | 101134 | 131163 | 212266 |
| SLL | 148156 | 174198 | 121145 | 147155 | 159195 | 234266 |
| SLL | 168180 | 178186 | 105109 | 116147 | 139159 | 212268 |
| SLL | 152178 | 162178 | 113117 | 151154 | 131163 | 196272 |
| SLL | 160230 | 178186 | 113125 | 131143 | 175203 | 212272 |
| SLL | 148168 | 194210 | 101105 | 129176 | 151175 | 206274 |
| SLL | 116194 | 190194 | 113125 | 94126 | 131155 | 216276 |
| SLL | 148198 | 187194 | 97109 | 111127 | 135159 | 272276 |
| SLL | 140175 | 166186 | 105105 | 123127 | 139175 | 172276 |
| SLL | 156180 | 186206 | 109113 | 117139 | 155183 | 224276 |
| SLL | 141194 | 186202 | 117121 | 122159 | 159159 | 224280 |
| SLL | 160160 | 178186 | 105105 | 143146 | 171171 | 196280 |
| SLL | 80124 | 174186 | 121121 | 135168 | 147171 | 217280 |
| SLL | 148212 | 166170 | 117125 | 126131 | 131171 | 220280 |
| SLL | 148176 | 178186 | 97133 | 216216 | 183195 | 248282 |
| SLL | 174180 | 186198 | 101125 | 138155 | 123139 | 232284 |
| SLL | 116152 | 170194 | 113125 | 123159 | 139143 | 258284 |
| SLL | 164168 | 166190 | 97121 | 135159 | 139163 | 260284 |
| SLL | 172190 | 190198 | 97121 | 127148 | 151179 | 228284 |
| SLL | 216216 | 178206 | 105129 | 106117 | 159207 | 262284 |
| SLL | 152152 | 174190 | 117133 | 119140 | 163195 | 231286 |
| SLL | 116160 | 182214 | 109133 | 122139 | 143159 | 212288 |
| SLL | 144152 | 186190 | 109137 | 137162 | 115163 | 192288 |
| SLL | 133152 | 158162 | 105105 | 114175 | 159167 | 176288 |
| SLL | 116166 | 166182 | 109109 | 119143 | 163175 | 192288 |
| SLL | 116168 | 174190 | 113113 | 110113 | 151179 | 254288 |
| SLL | 116152 | 182190 | 105125 | 114151 | 179183 | 280288 |
| SLL | 152164 | 166206 | 109113 | 126126 | 155159 | 324290 |
| SLL | 116164 | 174186 | 105121 | 119188 | 127151 | 168292 |
| SLL | 144160 | 166210 | 113117 | 135139 | 139151 | 240292 |
| SLL | 152192 | 170170 | 101113 | 110174 | 143155 | 248292 |
| SLL | 152156 | 170182 | 113117 | 102112 | 159159 | 188292 |
| SLL | 156176 | 178182 | 109145 | 124127 | 131171 | 254292 |
| SLL | 156200 | 178186 | 109117 | 135156 | 171179 | 288292 |
| SLL | 164172 | 178246 | 101117 | 113113 | 191207 | 184292 |
| SLL | 168190 | 166178 | 117125 | 135144 | 147167 | 192296 |
| SLL | 148180 | 182202 | 97109 | 130130 | 155179 | 280296 |
| SLL | 152168 | 170194 | 109117 | 142167 | 135155 | 228300 |
| SLL | 148204 | 162182 | 105121 | 151169 | 131167 | 296300 |
| SLL | 148152 | 162178 | 109113 | 139140 | 155175 | 188300 |
| SLL | 155186 | 182206 | 109113 | 110119 | 135179 | 264300 |
| SLL | 148182 | 202206 | 105113 | 127173 | 167179 | 300300 |
| SLL | 148152 | 170190 | 109113 | 110167 | 143151 | 204304 |
| SLL | 132152 | 182190 | 117121 | 122143 | 135159 | 172304 |
| SLL | 160200 | 162198 | 109129 | 118140 | 163171 | 180304 |
| SLL | 144148 | 166214 | 105113 | 110155 | 151171 | 272304 |
| SLL | 156184 | 170190 | 105109 | 125152 | 139155 | 288308 |
| SLL | 156224 | 174202 | 113125 | 114126 | 135163 | 288308 |
| SLL | 200204 | 182190 | 109125 | 119122 | 159179 | 164308 |
| SLL | 140164 | 162214 | 105117 | 112143 | 171171 | 168312 |
| SLL | 128164 | 162166 | 129133 | 114123 | 135179 | 160314 |
| SLL | 190206 | 186194 | 105105 | 142155 | 139163 | 256316 |
| SLL | 152190 | 178186 | 105113 | 114187 | 159175 | 260316 |
| SLL | 200232 | 178178 | 113113 | 120135 | 155203 | 228316 |
| SLL | 160208 | 166174 | 105117 | 102155 | 163167 | 256322 |
| SLL | 156156 | 170174 | 101101 | 147174 | 123179 | 232324 |
| SLL | 116156 | 174174 | 117129 | 91135 | 123163 | 224326 |
| SLL | 116208 | 162186 | 105125 | 103141 | 147203 | 212326 |
| SLL | 160172 | 174186 | 109113 | 98131 | 123135 | 300336 |
| SLL | 144218 | 194198 | 101117 | 150200 | 151167 | 200340 |
| SLL | 164230 | 162190 | 105121 | 135151 | 143183 | 188340 |
| SLL | 164178 | 170202 | 117117 | 106127 | 127135 | 168352 |
| SLL | 152170 | 174186 | 113133 | 110135 | 135155 | 264356 |
| SLL | 182196 | 174202 | 109117 | 153156 | 143151 | 234360 |
| SLL | 144176 | 186202 | 109109 | 135151 | 143179 | 220394 |
| SR | 172176 | 174186 | 109137 | 138151 | 143159 | 160176 |
| SR | 192196 | 186210 | 117121 | 131184 | 187187 | 176176 |
| SR | 148148 | 210214 | 105117 | 137151 | 135187 | 180198 |
| SR | 156180 | 198198 | 125125 | 122133 | 131163 | 192200 |
| SR | 156168 | 166194 | 113125 | 118143 | 127151 | 184204 |
| SR | 120200 | 166174 | 109137 | 114130 | 139179 | 168208 |
| SR | 156184 | 178190 | 105125 | 105122 | 191191 | 180208 |
| SR | 172190 | 170206 | 109121 | 170188 | 159163 | 176215 |
| SR | 168186 | 194202 | 109117 | 150155 | 159163 | 172216 |
| SR | 190190 | 162182 | 105125 | 134143 | 155159 | 204220 |
| SR | 144160 | 178182 | 105113 | 136194 | 151155 | 200222 |
| SR | 148156 | 182206 | 109113 | 111126 | 171187 | 222222 |
| SR | 148164 | 178210 | 109125 | 162214 | 155163 | 212225 |
| SR | 152176 | 162170 | 109113 | 141202 | 155191 | 176225 |
| SR | 180226 | 166190 | 101121 | 118147 | 123147 | 176226 |
| SR | 148164 | 182182 | 105105 | 134151 | 155171 | 208226 |
| SR | 148208 | 186210 | 109113 | 119146 | 151163 | 196228 |
| SR | 152156 | 166186 | 105109 | 114122 | 171171 | 180228 |
| SR | 172180 | 166194 | 109109 | 131150 | 163191 | 204228 |
| SR | 156186 | 174190 | 117125 | 105114 | 143199 | 196228 |
| SR | 148202 | 190218 | 101109 | 162162 | 155199 | 228228 |
| SR | 144208 | 170194 | 121121 | 106134 | 155155 | 226229 |
| SR | 152152 | 162182 | 117129 | 138145 | 131183 | 176229 |
| SR | 156190 | 186222 | 105109 | 155197 | 183183 | 192233 |
| SR | 164212 | 182194 | 113117 | 97101 | 147183 | 215234 |
| SR | 144172 | 170170 | 117125 | 104105 | 147155 | 202236 |
| SR | 156216 | 182190 | 97113 | 107114 | 159163 | 184236 |
| SR | 160220 | 166178 | 105109 | 147147 | 159163 | 216238 |
| SR | 170186 | 194198 | 117141 | 153168 | 139159 | 168248 |
| SR | 202206 | 178182 | 113125 | 107139 | 159159 | 184248 |
| SR | 136210 | 182194 | 105113 | 99110 | 171203 | 205268 |
| SR | 194210 | 178194 | 105121 | 188248 | 127135 | 228272 |
| SR | 156168 | 166170 | 105125 | 101166 | 163187 | 229272 |
| SR | 148148 | 178202 | 109109 | 134134 | 131147 | 272276 |
| SR | 140158 | 174198 | 117117 | 142158 | 147151 | 272276 |
| SR | 156160 | 162186 | 113121 | 109115 | 163175 | 276276 |
| SR | 132164 | 190198 | 121121 | 172226 | 155191 | 232288 |
| SR | 146154 | 186194 | 113125 | 142177 | 135147 | 188292 |
| SR | 198220 | 194218 | 105117 | 100147 | 139151 | 284292 |
| SR | 116168 | 166196 | 105121 | 114150 | 163167 | 196292 |
| SR | 160188 | 186194 | 101121 | 135155 | 151203 | 208292 |
| SR | 148166 | 166174 | 109113 | 114151 | 147207 | 244292 |
| SR | 156190 | 170230 | 109113 | 122130 | 127143 | 284296 |
| SR | 156160 | 182206 | 117125 | 121208 | 139151 | 212296 |
| SR | 164240 | 182202 | 113121 | 118142 | 171171 | 244296 |
| SR | 116172 | 174178 | 101129 | 118149 | 143163 | 168300 |
| SR | 180190 | 174190 | 105113 | 113162 | 135163 | 204300 |
| SR | 144156 | 182210 | 117117 | 102188 | 143143 | 204304 |
| SR | 164164 | 194198 | 113129 | 110110 | 143159 | 180304 |
| SR | 172202 | 186186 | 109117 | 109162 | 155159 | 180304 |
| SR | 80176 | 170210 | 117129 | 115173 | 151171 | 232304 |
| SR | 194206 | 190194 | 109113 | 106168 | 139139 | 284308 |
| SR | 156164 | 182182 | 113121 | 163193 | 139159 | 226308 |
| SR | 160190 | 178198 | 101117 | 103136 | 143167 | 250308 |
| SR | 116186 | 170178 | 105109 | 109142 | 159167 | 296308 |
| SR | 140172 | 178226 | 113113 | 150150 | 151211 | 229308 |
| SR | 128164 | 170182 | 109117 | 107123 | 139163 | 292312 |
| SR | 148166 | 194198 | 117117 | 123155 | 135191 | 184312 |
| SR | 160176 | 198198 | 105109 | 126142 | 163195 | 220312 |
| SR | 166232 | 198206 | 105125 | 151191 | 155183 | 296316 |
| SR | 160168 | 162182 | 105113 | 106138 | 115195 | 164346 |
| SR | 156172 | 186202 | 121129 | 132139 | 143143 | 316388 |
| SS | 156166 | 194198 | 93101 | 107168 | 147143 | 170170 |
| SS | 196212 | 174206 | 109129 | 155155 | 151175 | 158178 |
| SS | 156206 | 166214 | 109121 | 115159 | 151171 | 204204 |
| SS | 140184 | 190194 | 109109 | 119179 | 143207 | 208208 |
| SS | 164172 | 174202 | 101113 | 155159 | 159167 | 200212 |
| SS | 152228 | 186190 | 113137 | 110113 | 187219 | 176212 |
| SS | 148167 | 194198 | 113125 | 115234 | 175191 | 214214 |
| SS | 180182 | 194198 | 113113 | 115155 | 151163 | 212216 |
| SS | 116148 | 174210 | 101117 | 150188 | 139175 | 168216 |
| SS | 176186 | 178202 | 109113 | 107139 | 155175 | 216216 |
| SS | 168180 | 178198 | 101105 | 114118 | 175187 | 166218 |
| SS | 136190 | 182210 | 109121 | 123143 | 155159 | 208224 |
| SS | 140180 | 182194 | 109121 | 147159 | 131167 | 188224 |
| SS | 156216 | 190214 | 101125 | 138155 | 151183 | 224224 |
| SS | 164184 | 186186 | 109133 | 172177 | 123175 | 212228 |
| SS | 164218 | 190198 | 109137 | 149150 | 187195 | 232232 |
| SS | 152156 | 174218 | 105117 | 109132 | 175179 | 212234 |
| SS | 164168 | 186218 | 105113 | 115115 | 163183 | 230234 |
| SS | 168200 | 166194 | 113129 | 97110 | 195207 | 230234 |
| SS | 126147 | 182226 | 105117 | 130194 | 135143 | 224236 |
| SS | 164180 | 170178 | 113133 | 115119 | 123147 | 236236 |
| SS | 116188 | 166186 | 105113 | 119183 | 179183 | 236236 |
| SS | 170172 | 166186 | 109117 | 103180 | 151151 | 236238 |
| SS | 144172 | 178186 | 117121 | 151183 | 155159 | 170240 |
| SS | 160200 | 170238 | 117129 | 147147 | 171183 | 203241 |
| SS | 148192 | 166190 | 109125 | 107134 | 151163 | 228252 |
| SS | 116168 | 214218 | 109121 | 114118 | 147159 | 244256 |
| SS | 160178 | 186190 | 113117 | 107119 | 143183 | 256256 |
| SS | 176176 | 194202 | 125125 | 139147 | 143183 | 256256 |
| SS | 192216 | 170186 | 109121 | 110203 | 147175 | 248268 |
| SS | 148216 | 162178 | 105121 | 119167 | 175187 | 212268 |
| SS | 144170 | 166194 | 117117 | 102167 | 139167 | 178270 |
| SS | 164188 | 174198 | 113117 | 133138 | 151179 | 264270 |
| SS | 160194 | 162186 | 113133 | 163166 | 135163 | 168284 |
| SS | 152172 | 166190 | 113113 | 155208 | 151167 | 208286 |
| SS | 156164 | 174186 | 117117 | 134203 | 151163 | 192288 |
| SS | 148164 | 186190 | 109125 | 103150 | 135167 | 226288 |
| SS | 156180 | 170198 | 113121 | 159180 | 167187 | 196288 |
| SS | 176198 | 174214 | 101109 | 115175 | 151151 | 178292 |
| SS | 148152 | 162202 | 109109 | 135135 | 147171 | 212292 |
| SS | 144200 | 166194 | 105125 | 118131 | 135159 | 170300 |
| SS | 136158 | 162166 | 101105 | 115129 | 159159 | 296300 |
| SS | 160200 | 178186 | 101109 | 128135 | 163163 | 176300 |
| SS | 164198 | 170170 | 109113 | 117118 | 139167 | 184300 |
| SS | 164172 | 166182 | 113129 | 122147 | 163167 | 174304 |
| SS | 204216 | 178182 | 101129 | 138146 | 147175 | 220308 |
| SS | 148206 | 130158 | 105109 | 123144 | 183203 | 166308 |
| SS | 144174 | 166194 | 113129 | 139155 | 147159 | 204316 |
| SS | 172232 | 166170 | 109121 | 130160 | 123139 | 220324 |
| SS | 160164 | 170186 | 109133 | 160161 | 155175 | 240326 |
| SS | 166172 | 178198 | 113113 | 110163 | 155183 | 312328 |
| SS | 147164 | 174198 | 105125 | 122123 | 123163 | 296340 |
| SS | 116116 | 186230 | 109125 | 115172 | 183187 | 268390 |
| SS | 140212 | 186190 | 121133 | 131139 | 171179 | 232394 |
| SS | 202202 | 210210 | 105129 | 127158 | 135163 | 274396 |
| SS | 116144 | 170182 | 113121 | 135143 | 147167 | 180180 |
| SS | 164172 | 170178 | 109117 | 174174 | 151203 | 192200 |
| SS | 148152 | 198202 | 109141 | 143147 | 147151 | 188216 |
| SS | 120148 | 162178 | 109113 | 99159 | 139151 | 212216 |
| SS | 160168 | 166182 | 121121 | 108174 | 143183 | 188216 |
| SS | 184208 | 174198 | 105113 | 127130 | 131147 | 216220 |
| SS | 176194 | 162226 | 109109 | 114118 | 127155 | 214220 |
| SS | 172210 | 166198 | 101125 | 118118 | 179179 | 219220 |
| SS | 144208 | 186190 | 101105 | 126208 | 135187 | 223223 |
| SS | 144172 | 170190 | 109109 | 178178 | 147151 | 180224 |
| SS | 160164 | 162162 | 105121 | 140187 | 135179 | 160224 |
| SS | 140184 | 194202 | 113121 | 106117 | 175227 | 202224 |
| SS | 116183 | 178182 | 121121 | 155158 | 159167 | 212228 |
| SS | 152180 | 174178 | 109121 | 115140 | 143175 | 228228 |
| SS | 160174 | 174190 | 105117 | 119123 | 167171 | 192230 |
| SS | 148164 | 162174 | 113129 | 160194 | 167183 | 184232 |
| SS | 144162 | 158190 | 105133 | 139176 | 159227 | 234234 |
| SS | 162176 | 186186 | 105117 | 143146 | 195231 | 164238 |
| SS | 144156 | 182190 | 101129 | 167167 | 143215 | 184240 |
| SS | 114168 | 182242 | 109125 | 129167 | 147159 | 208242 |
| SS | 172180 | 178210 | 101109 | 114139 | 131175 | 192242 |
| SS | 147156 | 170222 | 93121 | 114122 | 175179 | 168244 |
| SS | 134212 | 170190 | 105109 | 118162 | 143163 | 192248 |
| SS | 151184 | 178186 | 109109 | 135166 | 151167 | 220248 |
| SS | 166184 | 186198 | 109109 | 151159 | 151155 | 249249 |
| SS | 172200 | 166194 | 125125 | 102113 | 151151 | 192252 |
| SS | 116166 | 170174 | 113121 | 91127 | 183187 | 244252 |
| SS | 140170 | 166198 | 109117 | 118151 | 159167 | 180256 |
| SS | 156176 | 178194 | 113125 | 134188 | 159159 | 224264 |
| SS | 140184 | 170198 | 109121 | 131160 | 119143 | 266266 |
| SS | 156176 | 178182 | 105109 | 151206 | 163211 | 230268 |
| SS | 144222 | 182218 | 109113 | 135192 | 127167 | 212270 |
| SS | 192192 | 174178 | 113125 | 150158 | 143171 | 202274 |
| SS | 180184 | 186222 | 101145 | 149162 | 131147 | 180280 |
| SS | 152156 | 166182 | 121125 | 114118 | 143159 | 224280 |
| SS | 160210 | 166210 | 109113 | 220220 | 171207 | 180280 |
| SS | 148152 | 162170 | 113117 | 154184 | 155171 | 224284 |
| SS | 148180 | 202210 | 101113 | 101154 | 147163 | 244288 |
| SS | 144156 | 178218 | 109117 | 131141 | 151163 | 246288 |
| SS | 152172 | 190190 | 101109 | 134147 | 159191 | 172288 |
| SS | 140175 | 210210 | 109113 | 130158 | 135151 | 184292 |
| SS | 80136 | 162182 | 101121 | 118171 | 143155 | 284292 |
| SS | 148216 | 170194 | 97101 | 118137 | 171191 | 188292 |
| SS | 180192 | 186210 | 109125 | 139155 | 183199 | 272292 |
| SS | 202210 | 166178 | 101109 | 114184 | 155199 | 292292 |
| SS | 152192 | 198214 | 113121 | 102147 | 171199 | 280300 |
| SS | 152160 | 162162 | 101109 | 150155 | 143175 | 200304 |
| SS | 172190 | 158166 | 101117 | 127139 | 143179 | 304304 |
| SS | 194202 | 162166 | 105109 | 126146 | 135143 | 208308 |
| SS | 116164 | 162186 | 113117 | 110164 | 163183 | 276316 |
| SS | 140160 | 198198 | 101113 | 106146 | 159175 | 206320 |
| SS | 116148 | 186194 | 101105 | 106169 | 147171 | 264328 |
| SS | 116226 | 162214 | 101113 | 123164 | 131151 | 288342 |
| SS | 116168 | 178198 | 113113 | 135143 | 163183 | 258416 |
| SV | 116206 | 190210 | 105109 | 129160 | 159171 | 164164 |
| SV | 164164 | 178194 | 113121 | 122140 | 155179 | 164164 |
| SV | 116152 | 158166 | 105125 | 114151 | 131155 | 170178 |
| SV | 168178 | 170174 | 109113 | 126168 | 159171 | 188196 |
| SV | 163242 | 186210 | 101129 | 114147 | 179195 | 200200 |
| SV | 158182 | 166202 | 113121 | 160177 | 179247 | 180204 |
| SV | 160160 | 190218 | 85129 | 163167 | 163175 | 160214 |
| SV | 160164 | 178186 | 105121 | 122126 | 147223 | 172214 |
| SV | 124202 | 166170 | 97125 | 122126 | 211211 | 180216 |
| SV | 158164 | 158210 | 113113 | 122152 | 123155 | 192218 |
| SV | 139206 | 158170 | 105109 | 106124 | 123179 | 204218 |
| SV | 164194 | 182190 | 109109 | 129134 | 147155 | 220220 |
| SV | 202218 | 178202 | 117125 | 144148 | 131183 | 220220 |
| SV | 144168 | 178190 | 105109 | 164164 | 159175 | 188222 |
| SV | 148172 | 170182 | 109113 | 122144 | 171179 | 208224 |
| SV | 148202 | 174174 | 101121 | 144168 | 183183 | 160224 |
| SV | 144172 | 182182 | 101117 | 138168 | 179187 | 212224 |
| SV | 176198 | 170190 | 105113 | 181200 | 131159 | 226226 |
| SV | 148172 | 174210 | 113121 | 109113 | 159179 | 196226 |
| SV | 144162 | 174242 | 109129 | 121134 | 155163 | 228228 |
| SV | 148176 | 166174 | 105109 | 117184 | 151183 | 208228 |
| SV | 192194 | 166174 | 105125 | 147163 | 151183 | 224228 |
| SV | 140156 | 190214 | 113129 | 110122 | 171207 | 228228 |
| SV | 166172 | 174194 | 105109 | 118188 | 143147 | 212229 |
| SV | 116116 | 166190 | 109113 | 134163 | 159163 | 228232 |
| SV | 172202 | 154194 | 105125 | 109148 | 163171 | 218232 |
| SV | 140168 | 190198 | 109113 | 117190 | 163179 | 200232 |
| SV | 156156 | 198210 | 109133 | 112150 | 143155 | 196234 |
| SV | 182206 | 166218 | 105133 | 128168 | 163167 | 234234 |
| SV | 190218 | 166186 | 105117 | 122148 | 163183 | 218234 |
| SV | 132180 | 182186 | 113113 | 143154 | 139175 | 178236 |
| SV | 152178 | 178198 | 113113 | 105164 | 155183 | 172238 |
| SV | 132172 | 166190 | 105113 | 144160 | 139159 | 196240 |
| SV | 164186 | 174190 | 113117 | 156156 | 151171 | 228240 |
| SV | 156168 | 174190 | 97117 | 105143 | 163187 | 172240 |
| SV | 160164 | 186202 | 105117 | 143160 | 131183 | 184244 |
| SV | 164172 | 186210 | 105129 | 119119 | 159179 | 242248 |
| SV | 156160 | 158182 | 109113 | 102164 | 187187 | 200248 |
| SV | 164176 | 162162 | 117117 | 122143 | 151167 | 160260 |
| SV | 164176 | 162162 | 117117 | 122143 | 151167 | 160260 |
| SV | 160164 | 182210 | 113125 | 118136 | 159167 | 222260 |
| SV | 184206 | 158194 | 113129 | 113138 | 155207 | 209260 |
| SV | 136148 | 198210 | 105129 | 130180 | 159179 | 240264 |
| SV | 116164 | 182198 | 113121 | 118146 | 131135 | 264268 |
| SV | 144156 | 166190 | 113125 | 117122 | 159179 | 227268 |
| SV | 144156 | 166190 | 113125 | 117122 | 159179 | 228268 |
| SV | 190210 | 170194 | 105129 | 118134 | 167187 | 264268 |
| SV | 182248 | 162198 | 101105 | 118147 | 163171 | 224272 |
| SV | 164172 | 190202 | 109117 | 129134 | 135183 | 232272 |
| SV | 136164 | 174194 | 113125 | 155156 | 155187 | 216272 |
| SV | 184186 | 182182 | 117125 | 192211 | 147171 | 212276 |
| SV | 164198 | 170182 | 129141 | 114126 | 139183 | 252276 |
| SV | 152184 | 186186 | 101109 | 118140 | 147183 | 276276 |
| SV | 180186 | 158190 | 105121 | 106148 | 183187 | 200276 |
| SV | 170198 | 178190 | 109121 | 117150 | 143195 | 276276 |
| SV | 206214 | 186194 | 125125 | 122144 | 151207 | 184276 |
| SV | 148164 | 158174 | 109137 | 121125 | 147203 | 212280 |
| SV | 148160 | 170182 | 113117 | 158175 | 135135 | 228284 |
| SV | 152164 | 162198 | 113121 | 102113 | 127163 | 284288 |
| SV | 148198 | 206210 | 109133 | 134140 | 115175 | 280288 |
| SV | 186190 | 166194 | 113121 | 143176 | 175187 | 232288 |
| SV | 136162 | 166178 | 109117 | 126170 | 159159 | 292292 |
| SV | 136234 | 166170 | 109117 | 134139 | 159159 | 276296 |
| SV | 140159 | 190194 | 105109 | 109113 | 159171 | 219296 |
| SV | 138148 | 162178 | 105109 | 117220 | 167171 | 227296 |
| SV | 144162 | 162194 | 121121 | 112126 | 147191 | 216296 |
| SV | 192202 | 170190 | 113121 | 114192 | 155191 | 292296 |
| SV | 160176 | 158186 | 109117 | 128128 | 159199 | 180296 |
| SV | 136144 | 166174 | 101101 | 140144 | 179211 | 236296 |
| SV | 156174 | 190198 | 129129 | 144150 | 135155 | 232300 |
| SV | 168184 | 182182 | 97113 | 130174 | 151171 | 176300 |
| SV | 144194 | 170190 | 105113 | 113147 | 147175 | 246300 |
| SV | 148160 | 174202 | 109113 | 126139 | 127179 | 226300 |
| SV | 192194 | 166178 | 113117 | 109138 | 147183 | 215300 |
| SV | 128156 | 166194 | 109113 | 130214 | 159187 | 280300 |
| SV | 152160 | 166210 | 117117 | 125168 | 115163 | 192304 |
| SV | 128194 | 162174 | 125125 | 164177 | 163167 | 204304 |
| SV | 168194 | 166170 | 105117 | 113140 | 167171 | 230304 |
| SV | 152164 | 170210 | 105105 | 136156 | 139171 | 282304 |
| SV | 170206 | 162194 | 117121 | 97148 | 123183 | 296304 |
| SV | 148156 | 174182 | 105125 | 130131 | 179235 | 232304 |
| SV | 144164 | 162178 | 105113 | 148163 | 135159 | 308308 |
| SV | 160172 | 170178 | 109113 | 100100 | 155163 | 216308 |
| SV | 160176 | 178198 | 101113 | 207224 | 163171 | 252308 |
| SV | 160176 | 178198 | 101113 | 207224 | 163171 | 252308 |
| SV | 144198 | 186186 | 105113 | 102102 | 155171 | 296308 |
| SV | 160198 | 174198 | 113121 | 152164 | 175247 | 304308 |
| SV | 116164 | 190206 | 105117 | 144151 | 155195 | 300312 |
| SV | 168168 | 198202 | 101125 | 140144 | 167219 | 196312 |
| SV | 164176 | 186214 | 105105 | 103104 | 175195 | 308316 |
| SV | 164206 | 162190 | 117121 | 106139 | 155171 | 264328 |
| SV | 164206 | 162190 | 117121 | 106139 | 155171 | 264328 |
| SV | 156170 | 194194 | 113117 | 130186 | 175183 | 228328 |
| SV | 176214 | 162182 | 109133 | 134140 | 179179 | 264340 |
| SV | 144152 | 166178 | 109129 | 113121 | 175187 | 296348 |
| SV | 176246 | 166186 | 113121 | 117142 | 147151 | 208380 |
| SV | 152168 | 174202 | 101113 | 151177 | 131171 | 260384 |
| SV | 156178 | 190206 | 117129 | 134187 | 147147 | 240388 |
| SV | 144176 | 162194 | 105109 | 108159 | 151155 | 164392 |
| SV | 176180 | 178182 | 105109 | 149152 | 139163 | 160160 |
| SV | 174202 | 166178 | 105125 | 117136 | 171175 | 172172 |
| SV | 164184 | 182186 | 105121 | 164180 | 123215 | 172172 |
| SV | 124132 | 174210 | 109125 | 156164 | 175187 | 160192 |
| SV | 152206 | 170186 | 105117 | 109164 | 147159 | 184196 |
| SV | 164172 | 170190 | 121133 | 117140 | 147179 | 196196 |
| SV | 178184 | 170202 | 109109 | 105192 | 139155 | 184200 |
| SV | 164176 | 166178 | 109109 | 121140 | 135167 | 178208 |
| SV | 176182 | 178178 | 113117 | 135180 | 135171 | 160208 |
| SV | 140168 | 190194 | 105113 | 121180 | 135171 | 172208 |
| SV | 116168 | 166182 | 105125 | 152156 | 119155 | 184212 |
| SV | 156168 | 186190 | 125133 | 101156 | 147187 | 168212 |
| SV | 152168 | 162206 | 109113 | 121178 | 147195 | 208212 |
| SV | 116164 | 158202 | 109121 | 125162 | 127151 | 200216 |
| SV | 148172 | 178206 | 109149 | 121121 | 155163 | 176216 |
| SV | 176206 | 170202 | 117121 | 109121 | 139163 | 180216 |
| SV | 180182 | 166174 | 121129 | 112113 | 151171 | 204216 |
| SV | 160164 | 214218 | 109113 | 130144 | 147183 | 176218 |
| SV | 152178 | 162186 | 101141 | 105113 | 143151 | 152220 |
| SV | 142250 | 166190 | 101105 | 112143 | 171199 | 180220 |
| SV | 172180 | 182190 | 101109 | 108126 | 159175 | 172223 |
| SV | 144144 | 166194 | 105113 | 131140 | 163187 | 216224 |
| SV | 152160 | 162178 | 113133 | 140152 | 171175 | 184226 |
| SV | 136164 | 174186 | 117117 | 117139 | 147147 | 216228 |
| SV | 140144 | 166166 | 105113 | 108172 | 159159 | 160228 |
| SV | 168198 | 174198 | 105109 | 143147 | 143159 | 164228 |
| SV | 172202 | 166178 | 117117 | 140170 | 135163 | 208228 |
| SV | 206218 | 162170 | 113117 | 139156 | 135163 | 224228 |
| SV | 128206 | 162174 | 109121 | 124152 | 143167 | 180228 |
| SV | 176210 | 170174 | 121125 | 134172 | 171179 | 212228 |
| SV | 82164 | 190202 | 109125 | 105105 | 163179 | 220228 |
| SV | 148222 | 174190 | 105121 | 178186 | 159183 | 192228 |
| SV | 164210 | 170210 | 109121 | 117176 | 139183 | 212228 |
| SV | 156168 | 166170 | 109113 | 160172 | 155187 | 216228 |
| SV | 156160 | 162210 | 109129 | 105156 | 163195 | 200228 |
| SV | 152172 | 174174 | 101109 | 126134 | 159163 | 208230 |
| SV | 116144 | 166166 | 109113 | 136192 | 171195 | 230230 |
| SV | 164166 | 166190 | 109117 | 109140 | 155171 | 160232 |
| SV | 144170 | 166218 | 101109 | 112144 | 171199 | 152232 |
| SV | 140144 | 214214 | 109137 | 145168 | 135155 | 224233 |
| SV | 178178 | 170202 | 109109 | 113117 | 159167 | 172236 |
| SV | 152174 | 182202 | 105109 | 109122 | 163167 | 176244 |
| SV | 144230 | 186198 | 105113 | 128152 | 159199 | 168244 |
| SV | 116148 | 182190 | 109117 | 117155 | 159199 | 240244 |
| SV | 152230 | 162206 | 113117 | 135196 | 143211 | 236245 |
| SV | 176180 | 206210 | 109113 | 125140 | 131131 | 212248 |
| SV | 168186 | 178194 | 117125 | 128160 | 163163 | 224248 |
| SV | 140160 | 174182 | 109113 | 125125 | 159159 | 168252 |
| SV | 148200 | 190198 | 109129 | 129186 | 155167 | 168252 |
| SV | 116188 | 170190 | 101113 | 100147 | 123167 | 172252 |
| SV | 136156 | 190190 | 105109 | 97143 | 163163 | 176256 |
| SV | 148156 | 162182 | 109137 | 114128 | 147163 | 236256 |
| SV | 116172 | 178182 | 109109 | 126152 | 179203 | 216256 |
| SV | 156184 | 194202 | 105113 | 120148 | 159203 | 224256 |
| SV | 144168 | 178238 | 109137 | 118130 | 135171 | 176260 |
| SV | 164164 | 162178 | 105113 | 121192 | 143175 | 220260 |
| SV | 144156 | 166170 | 109109 | 139140 | 159167 | 200264 |
| SV | 168188 | 162166 | 109137 | 108124 | 151167 | 212264 |
| SV | 156180 | 170170 | 101109 | 109124 | 139167 | 232266 |
| SV | 116152 | 178186 | 105133 | 101140 | 155167 | 244268 |
| SV | 128200 | 166210 | 109117 | 130136 | 175183 | 232268 |
| SV | 166168 | 186190 | 117125 | 117234 | 183191 | 216268 |
| SV | 160160 | 166190 | 109121 | 113117 | 163167 | 232274 |
| SV | 152170 | 194218 | 109129 | 136136 | 147159 | 192276 |
| SV | 140140 | 174182 | 105109 | 116122 | 163187 | 208276 |
| SV | 156156 | 162198 | 105113 | 96117 | 155163 | 244280 |
| SV | 156200 | 182190 | 113121 | 143172 | 155183 | 168284 |
| SV | 152188 | 182206 | 109121 | 105140 | 143211 | 228284 |
| SV | 230234 | 182182 | 117121 | 134172 | 155159 | 280288 |
| SV | 116136 | 166206 | 109109 | 113180 | 183191 | 216288 |
| SV | 120196 | 178182 | 113117 | 142179 | 163191 | 240288 |
| SV | 180218 | 182202 | 109117 | 116130 | 151203 | 238288 |
| SV | 144226 | 170214 | 109129 | 132136 | 123143 | 228292 |
| SV | 160198 | 190198 | 113117 | 130175 | 155179 | 244292 |
| SV | 122160 | 166182 | 109113 | 140172 | 143151 | 284296 |
| SV | 116144 | 170174 | 105121 | 151160 | 123163 | 232296 |
| SV | 144148 | 170174 | 101113 | 134184 | 155179 | 288296 |
| SV | 152164 | 198210 | 109121 | 149188 | 119183 | 248296 |
| SV | 136156 | 178190 | 113121 | 132140 | 163195 | 172296 |
| SV | 148180 | 170194 | 113117 | 117232 | 179195 | 224296 |
| SV | 152168 | 174178 | 117117 | 135156 | 187203 | 284296 |
| SV | 156168 | 162202 | 113137 | 109130 | 135151 | 180300 |
| SV | 144170 | 186202 | 109125 | 164184 | 131171 | 168300 |
| SV | 168203 | 190210 | 125125 | 121152 | 163183 | 260300 |
| SV | 160172 | 166186 | 101113 | 148152 | 143199 | 216300 |
| SV | 152170 | 162194 | 113113 | 109144 | 123163 | 160304 |
| SV | 132132 | 170170 | 109133 | 144180 | 143163 | 232304 |
| SV | 156156 | 170194 | 101109 | 116130 | 159167 | 245304 |
| SV | 160168 | 166194 | 117125 | 134152 | 183199 | 160308 |
| SV | 132156 | 182218 | 109109 | 113113 | 131183 | 292320 |
| SV | 140148 | 174194 | 105113 | 130130 | 163175 | 328328 |
| SV | 152160 | 166178 | 105113 | 105160 | 139215 | 216328 |
| SV | 160194 | 178178 | 109121 | 135171 | 147171 | 233384 |
| SV | 180206 | 178178 | 105129 | 112139 | 135175 | 208384 |
| SV | 166182 | 182190 | 121129 | 168168 | 167199 | 216384 |
| SV | 160164 | 194202 | 113121 | 116126 | 131203 | 288384 |
| SV | 148166 | 170186 | 101109 | 130151 | 155243 | 220388 |
| SV | 164184 | 170230 | 105113 | 147176 | 143171 | 170170 |
| SV | 159176 | 182190 | 125129 | 138162 | 151159 | 162178 |
| SV | 148156 | 162174 | 117125 | 123138 | 143179 | 180188 |
| SV | 120214 | 170202 | 117129 | 135184 | 159171 | 196196 |
| SV | 144168 | 202214 | 109121 | 147176 | 143179 | 174196 |
| SV | 156156 | 162166 | 105117 | 146180 | 155171 | 178208 |
| SV | 182206 | 170178 | 113117 | 115158 | 163179 | 162208 |
| SV | 138175 | 182186 | 109109 | 112122 | 159167 | 204212 |
| SV | 206242 | 178202 | 101121 | 157163 | 151219 | 216216 |
| SV | 152172 | 186186 | 105129 | 115127 | 127187 | 216218 |
| SV | 80080 | 174194 | 101105 | 107151 | 163171 | 180220 |
| SV | 161172 | 198202 | 109117 | 142164 | 131159 | 222222 |
| SV | 140152 | 178182 | 105121 | 134158 | 139147 | 224224 |
| SV | 160164 | 198202 | 105117 | 127150 | 155171 | 220224 |
| SV | 152152 | 166174 | 121125 | 134177 | 159183 | 210224 |
| SV | 144152 | 170190 | 109133 | 107130 | 131171 | 208226 |
| SV | 164182 | 186190 | 109113 | 147171 | 155191 | 222226 |
| SV | 182190 | 162166 | 117117 | 113147 | 143143 | 224228 |
| SV | 148204 | 174194 | 101109 | 128134 | 163171 | 228228 |
| SV | 152160 | 162170 | 109125 | 115198 | 139143 | 196230 |
| SV | 180180 | 166198 | 113121 | 138168 | 199219 | 212230 |
| SV | 164164 | 186210 | 105113 | 119171 | 135175 | 200236 |
| SV | 156168 | 166214 | 109125 | 126151 | 135183 | 160238 |
| SV | 164194 | 162182 | 105133 | 172188 | 159179 | 166242 |
| SV | 156188 | 170182 | 109117 | 126151 | 139159 | 212246 |
| SV | 152194 | 158162 | 101113 | 99139 | 167175 | 204246 |
| SV | 116172 | 174194 | 113117 | 116116 | 155167 | 236250 |
| SV | 164182 | 178198 | 109117 | 103138 | 135179 | 237258 |
| SV | 152170 | 170214 | 101113 | 148148 | 159191 | 232260 |
| SV | 152204 | 174178 | 101133 | 120142 | 151167 | 234262 |
| SV | 164227 | 158178 | 121121 | 106119 | 171187 | 208262 |
| SV | 185230 | 170214 | 117125 | 137143 | 175215 | 178266 |
| SV | 166166 | 166182 | 109129 | 139176 | 151163 | 224268 |
| SV | 148158 | 162170 | 109109 | 119119 | 143167 | 220268 |
| SV | 152152 | 162162 | 117129 | 147222 | 143191 | 216268 |
| SV | 116160 | 190210 | 105117 | 107158 | 143195 | 168268 |
| SV | 164176 | 190190 | 105113 | 115127 | 143143 | 242270 |
| SV | 116133 | 170186 | 121125 | 119150 | 175187 | 172280 |
| SV | 148160 | 166166 | 109121 | 167186 | 147151 | 226284 |
| SV | 176208 | 190194 | 109129 | 123167 | 151159 | 236284 |
| SV | 116116 | 166186 | 113121 | 134139 | 147167 | 268284 |
| SV | 148238 | 194202 | 105109 | 99175 | 143171 | 232284 |
| SV | 152172 | 198202 | 109121 | 143151 | 143199 | 246284 |
| SV | 136208 | 174178 | 117121 | 108127 | 175231 | 192284 |
| SV | 172214 | 166194 | 129129 | 138171 | 163167 | 236286 |
| SV | 160176 | 174206 | 109117 | 111131 | 131159 | 250292 |
| SV | 116136 | 162182 | 101109 | 119158 | 143171 | 208292 |
| SV | 148167 | 174242 | 105117 | 107110 | 159175 | 232292 |
| SV | 160181 | 158198 | 101109 | 114134 | 135179 | 216292 |
| SV | 164167 | 170170 | 117121 | 102147 | 131191 | 280292 |
| SV | 164178 | 178214 | 105125 | 138200 | 147163 | 188296 |
| SV | 136152 | 182190 | 97129 | 139200 | 139199 | 220296 |
| SV | 160170 | 190202 | 105109 | 151172 | 155199 | 244296 |
| SV | 128168 | 166170 | 109109 | 149149 | 155183 | 242298 |
| SV | 168196 | 178194 | 113125 | 119134 | 159163 | 296300 |
| SV | 159184 | 170178 | 113117 | 168168 | 135163 | 296304 |
| SV | 144184 | 174186 | 105117 | 103147 | 163215 | 304304 |
| SV | 146165 | 190222 | 105105 | 115121 | 139179 | 276306 |
| SV | 160168 | 158166 | 109113 | 116128 | 167191 | 292308 |
| SV | 148170 | 186198 | 97117 | 142146 | 171203 | 224308 |
| SV | 176200 | 174202 | 101101 | 131188 | 163179 | 236312 |
| SV | 164170 | 162186 | 109125 | 130151 | 139179 | 226326 |
| SV | 168176 | 178182 | 109109 | 122126 | 151151 | 184384 |
| SV | 80084 | 162178 | 109129 | 125134 | 163167 | 304394 |
| 2005SV | 160220 | 166178 | 101113 | 137143 | 135159 | 158158 |
| 2005SV | 140140 | 186190 | 113125 | 107111 | 147187 | 158158 |
| 2005SV | 152156 | 186194 | 121133 | 155163 | 163195 | 158158 |
| 2005SV | 152224 | 166174 | 117121 | 111124 | 151155 | 168168 |
| 2005SV | 140156 | 166166 | 105105 | 115135 | 143187 | 184184 |
| 2005SV | 112112 | 186222 | 125129 | 114114 | 147179 | 188188 |
| 2005SV | 168208 | 158178 | 109109 | 119155 | 163203 | 188188 |
| 2005SV | 152163 | 194202 | 109125 | 125131 | 155155 | 192192 |
| 2005SV | 134148 | 170202 | 105125 | 138147 | 155167 | 200200 |
| 2005SV | 160168 | 186198 | 113121 | 119177 | 155167 | 172212 |
| 2005SV | 168188 | 174202 | 101105 | 115115 | 151171 | 156216 |
| 2005SV | 152200 | 170194 | 101105 | 111139 | 211227 | 172216 |
| 2005SV | 156246 | 174198 | 97109 | 157163 | 155175 | 174220 |
| 2005SV | 160232 | 166174 | 101101 | 111159 | 147159 | 182224 |
| 2005SV | 175180 | 210210 | 109121 | 123188 | 123151 | 212228 |
| 2005SV | 176188 | 178182 | 121141 | 121171 | 139159 | 188228 |
| 2005SV | 140192 | 182222 | 113121 | 119151 | 155191 | 204228 |
| 2005SV | 136144 | 162182 | 101121 | 143187 | 163171 | 208230 |
| 2005SV | 140168 | 166174 | 101125 | 134156 | 147151 | 208234 |
| 2005SV | 112148 | 202202 | 121141 | 159159 | 135191 | 192234 |
| 2005SV | 132144 | 162186 | 105109 | 159188 | 151167 | 242242 |
| 2005SV | 144170 | 170194 | 105133 | 111162 | 179183 | 216248 |
| 2005SV | 164196 | 178194 | 105109 | 139168 | 147199 | 158248 |
| 2005SV | 164164 | 230230 | 105109 | 130159 | 147159 | 226262 |
| 2005SV | 147184 | 166178 | 105113 | 151163 | 135183 | 210262 |
| 2005SV | 152180 | 158194 | 105133 | 111128 | 175187 | 266266 |
| 2005SV | 163172 | 178190 | 121125 | 123147 | 163175 | 264272 |
| 2005SV | 136154 | 190214 | 97109 | 111168 | 159163 | 208276 |
| 2005SV | 166226 | 170190 | 113121 | 135159 | 167179 | 158276 |
| 2005SV | 220224 | 170178 | 105121 | 117159 | 151163 | 204280 |
| 2005SV | 116144 | 190194 | 97129 | 132132 | 147163 | 280284 |
| 2005SV | 172212 | 158182 | 113113 | 118167 | 147167 | 242288 |
| 2005SV | 170174 | 182198 | 113121 | 131135 | 171183 | 228288 |
| 2005SV | 148192 | 170178 | 101109 | 106106 | 131191 | 238288 |
| 2005SV | 152192 | 162182 | 113113 | 130159 | 147167 | 170292 |
| 2005SV | 140168 | 174202 | 109113 | 107155 | 159219 | 204296 |
| 2005SV | 156200 | 170190 | 109117 | 127163 | 143159 | 208304 |
| 2005SV | 148176 | 170178 | 105109 | 128130 | 159163 | 234304 |
| 2005SV | 136176 | 174194 | 93113 | 126155 | 159171 | 244320 |
| TW | 148152 | 174198 | 109113 | 117126 | 159159 | 164192 |
| TW | 132144 | 170190 | 113113 | 140140 | 151155 | 196200 |
| TW | 116176 | 174186 | 97105 | 126130 | 155167 | 188204 |
| TW | 164164 | 128178 | 101109 | 163179 | 135183 | 168208 |
| TW | 156160 | 162190 | 105109 | 144148 | 139139 | 216216 |
| TW | 164172 | 158206 | 105113 | 157168 | 131135 | 220220 |
| TW | 135140 | 158182 | 109121 | 117171 | 147155 | 216220 |
| TW | 144178 | 162182 | 129129 | 142156 | 163167 | 168220 |
| TW | 178210 | 158190 | 105121 | 130200 | 179183 | 192220 |
| TW | 168194 | 194202 | 105109 | 121183 | 159183 | 215220 |
| TW | 116190 | 166198 | 109125 | 116148 | 123195 | 208220 |
| TW | 148200 | 162162 | 101109 | 113184 | 143199 | 160224 |
| TW | 160166 | 190198 | 117137 | 125168 | 131167 | 225225 |
| TW | 148168 | 178186 | 113113 | 121179 | 159163 | 220228 |
| TW | 160200 | 194220 | 109121 | 114143 | 167175 | 160228 |
| TW | 160168 | 170186 | 113117 | 113148 | 155175 | 208228 |
| TW | 168198 | 166186 | 105113 | 148224 | 183199 | 180229 |
| TW | 140168 | 174178 | 109125 | 104138 | 135167 | 208230 |
| TW | 166172 | 190206 | 105137 | 142146 | 147163 | 188232 |
| TW | 156206 | 178178 | 105113 | 105140 | 163163 | 232232 |
| TW | 164196 | 186198 | 97105 | 100137 | 139155 | 228235 |
| TW | 148156 | 178182 | 105121 | 141164 | 151167 | 212236 |
| TW | 138140 | 194198 | 109121 | 100148 | 167171 | 200236 |
| TW | 158184 | 182190 | 105109 | 126138 | 155159 | 219240 |
| TW | 167196 | 170174 | 109109 | 119147 | 159191 | 236240 |
| TW | 164170 | 206206 | 105113 | 156156 | 147175 | 220244 |
| TW | 160222 | 182202 | 113113 | 103172 | 151187 | 228244 |
| TW | 180206 | 178202 | 105113 | 138159 | 167231 | 192248 |
| TW | 116152 | 186214 | 105117 | 140144 | 163171 | 192252 |
| TW | 176234 | 174182 | 105105 | 113127 | 135159 | 224254 |
| TW | 116160 | 190198 | 113113 | 125131 | 139159 | 236256 |
| TW | 152152 | 166174 | 101105 | 117130 | 171175 | 252256 |
| TW | 197200 | 166206 | 105129 | 134142 | 135179 | 168257 |
| TW | 120144 | 190194 | 109113 | 113122 | 135167 | 224260 |
| TW | 152160 | 182182 | 113121 | 118171 | 139183 | 225260 |
| TW | 156156 | 178186 | 113113 | 112146 | 143155 | 188268 |
| TW | 144148 | 194214 | 105129 | 109156 | 147171 | 192272 |
| TW | 164184 | 174174 | 105121 | 148156 | 155183 | 216272 |
| TW | 148172 | 174222 | 105121 | 113212 | 147155 | 168276 |
| TW | 152156 | 178198 | 105113 | 98105 | 155175 | 272277 |
| TW | 152164 | 174206 | 121137 | 122155 | 151167 | 212280 |
| TW | 132164 | 178186 | 121129 | 121180 | 163183 | 184280 |
| TW | 124166 | 162190 | 109117 | 129155 | 147187 | 220280 |
| TW | 123172 | 128186 | 113117 | 121159 | 163187 | 241280 |
| TW | 152152 | 178202 | 113117 | 113118 | 163199 | 280280 |
| TW | 148184 | 174198 | 121121 | 105156 | 183211 | 256280 |
| TW | 116180 | 174190 | 105117 | 135246 | 143151 | 220284 |
| TW | 114202 | 170174 | 121125 | 131220 | 167167 | 224284 |
| TW | 164168 | 174182 | 105121 | 97138 | 175179 | 176284 |
| TW | 160194 | 162198 | 117121 | 118118 | 159163 | 160288 |
| TW | 156160 | 190190 | 117117 | 118121 | 175179 | 254288 |
| TW | 152160 | 170182 | 117125 | 109122 | 151183 | 160288 |
| TW | 160202 | 158234 | 121129 | 118122 | 155195 | 192288 |
| TW | 152174 | 166166 | 121129 | 96118 | 120139 | 276292 |
| TW | 140156 | 182210 | 121129 | 113131 | 143163 | 204292 |
| TW | 116160 | 170186 | 101129 | 172172 | 159167 | 188292 |
| TW | 168180 | 174178 | 105105 | 152180 | 147171 | 196292 |
| TW | 116152 | 174190 | 109117 | 168188 | 171175 | 228292 |
| TW | 164168 | 186194 | 133133 | 126126 | 151175 | 276292 |
| TW | 166168 | 194198 | 113113 | 139156 | 175203 | 228292 |
| TW | 148202 | 166194 | 109109 | 113139 | 127131 | 296296 |
| TW | 148172 | 166178 | 121129 | 105188 | 135143 | 296296 |
| TW | 148160 | 174202 | 117121 | 125156 | 139151 | 172296 |
| TW | 182186 | 182198 | 101117 | 103147 | 143159 | 232296 |
| TW | 168173 | 186206 | 109117 | 104122 | 139163 | 260296 |
| TW | 160198 | 162214 | 101125 | 113180 | 135163 | 288296 |
| TW | 182202 | 190190 | 105121 | 127216 | 167167 | 244296 |
| TW | 156198 | 166178 | 101105 | 176176 | 167171 | 172296 |
| TW | 160202 | 162214 | 97133 | 122164 | 131171 | 220296 |
| TW | 176210 | 178194 | 113113 | 122151 | 163179 | 212296 |
| TW | 144202 | 182190 | 117129 | 117168 | 131191 | 252296 |
| TW | 156190 | 182194 | 113121 | 108118 | 139139 | 204300 |
| TW | 116148 | 186198 | 105109 | 130168 | 147151 | 256300 |
| TW | 128147 | 186190 | 109117 | 126171 | 151167 | 264300 |
| TW | 160186 | 162186 | 109109 | 130176 | 147175 | 196300 |
| TW | 140165 | 186186 | 101121 | 96161 | 159175 | 292300 |
| TW | 144180 | 190198 | 117121 | 112163 | 163183 | 200300 |
| TW | 175212 | 182194 | 105109 | 142164 | 151191 | 296300 |
| TW | 116160 | 178182 | 105161 | 125142 | 131131 | 212304 |
| TW | 166332 | 170190 | 105133 | 175206 | 147151 | 184304 |
| TW | 140176 | 178206 | 113117 | 104134 | 131167 | 212304 |
| TW | 136186 | 178214 | 97109 | 125136 | 155183 | 192304 |
| TW | 144156 | 182206 | 105125 | 136136 | 163191 | 223304 |
| TW | 135176 | 206206 | 113137 | 130164 | 151195 | 212304 |
| TW | 116160 | 162218 | 113129 | 112135 | 135167 | 232308 |
| TW | 160174 | 170186 | 117133 | 126126 | 171183 | 240308 |
| TW | 116180 | 170218 | 109121 | 112160 | 115163 | 266310 |
| TW | 136136 | 190194 | 109117 | 118148 | 143155 | 284312 |
| TW | 144168 | 178206 | 109117 | 125164 | 159167 | 168316 |
| TW | 84160 | 170174 | 97101 | 126130 | 163167 | 248316 |
| TW | 160160 | 202206 | 101125 | 109122 | 179183 | 240316 |
| TW | 128148 | 182198 | 117141 | 135151 | 151195 | 276316 |
| TW | 168212 | 178194 | 105113 | 109125 | 135151 | 304320 |
| TW | 140164 | 174202 | 101121 | 113152 | 139151 | 280328 |
| TW | 156196 | 166170 | 105121 | 156187 | 151167 | 192348 |
| TW | 152166 | 182182 | 117125 | 117130 | 127175 | 272352 |
| TW | 148148 | 174206 | 113121 | 113160 | 139155 | 276364 |
| TW | 214218 | 190202 | 105129 | 113164 | 175183 | 223392 |
| TW | 116216 | 182190 | 109117 | 109144 | 139171 | 170174 |
| TW | 136204 | 162206 | 117121 | 114146 | 143151 | 172184 |
| TW | 148170 | 166194 | 97121 | 155155 | 131163 | 176184 |
| TW | 188214 | 166210 | 105125 | 113146 | 175191 | 196196 |
| TW | 152152 | 166174 | 101157 | 123123 | 159223 | 192198 |
| TW | 156164 | 162166 | 113121 | 105134 | 123171 | 208208 |
| TW | 148172 | 182194 | 105121 | 113138 | 167203 | 208208 |
| TW | 172210 | 162178 | 109141 | 123180 | 159167 | 170212 |
| TW | 152184 | 174178 | 121153 | 145155 | 155179 | 174212 |
| TW | 171202 | 174182 | 105117 | 155172 | 143171 | 174215 |
| TW | 168202 | 162178 | 117137 | 128140 | 131159 | 200216 |
| TW | 144172 | 190222 | 109117 | 110155 | 127163 | 216216 |
| TW | 172176 | 194194 | 113129 | 158196 | 151203 | 212220 |
| TW | 156220 | 166182 | 121121 | 122133 | 127163 | 170224 |
| TW | 180202 | 186206 | 109117 | 123172 | 147171 | 220224 |
| TW | 116144 | 182198 | 113113 | 133144 | 167199 | 158224 |
| TW | 140156 | 178202 | 101117 | 143193 | 139239 | 208224 |
| TW | 116202 | 170186 | 105121 | 116147 | 143147 | 188225 |
| TW | 158222 | 162166 | 117121 | 127133 | 167211 | 164228 |
| TW | 148202 | 170178 | 105109 | 147155 | 143171 | 224232 |
| TW | 152176 | 162186 | 105129 | 123147 | 207207 | 223232 |
| TW | 148190 | 158166 | 113121 | 144167 | 151163 | 218236 |
| TW | 156160 | 166178 | 129133 | 139166 | 171187 | 227252 |
| TW | 164172 | 186198 | 109117 | 165182 | 159203 | 166252 |
| TW | 160194 | 174190 | 101117 | 127143 | 151155 | 244254 |
| TW | 148168 | 162202 | 113129 | 117117 | 159159 | 158256 |
| TW | 156196 | 158202 | 105117 | 113117 | 155167 | 166256 |
| TW | 140170 | 162186 | 113113 | 110163 | 139179 | 256256 |
| TW | 164194 | 170182 | 117121 | 91175 | 155191 | 256256 |
| TW | 152164 | 194198 | 117121 | 116146 | 143159 | 178260 |
| TW | 160172 | 166166 | 109117 | 124139 | 163199 | 232260 |
| TW | 144182 | 166174 | 109113 | 125143 | 155159 | 228264 |
| TW | 178186 | 190198 | 101121 | 164168 | 195207 | 222268 |
| TW | 116208 | 162182 | 117125 | 142159 | 151155 | 170276 |
| TW | 124148 | 162170 | 109117 | 110148 | 147155 | 212276 |
| TW | 194234 | 174206 | 101109 | 127147 | 163171 | 192276 |
| TW | 164202 | 178190 | 113113 | 122137 | 147223 | 196276 |
| TW | 116160 | 178186 | 105133 | 97113 | 147163 | 219280 |
| TW | 152156 | 190198 | 105129 | 138171 | 159215 | 160284 |
| TW | 186216 | 170186 | 101121 | 131135 | 147203 | 228288 |
| TW | 148172 | 178194 | 113117 | 113144 | 155155 | 182292 |
| TW | 156164 | 182218 | 105109 | 109140 | 135175 | 250292 |
| TW | 116148 | 178190 | 105121 | 127127 | 191247 | 208292 |
| TW | 176180 | 190210 | 109113 | 136144 | 171171 | 212296 |
| TW | 166180 | 178182 | 105113 | 114138 | 119203 | 272296 |
| TW | 144152 | 174202 | 109109 | 151159 | 175175 | 212300 |
| TW | 148202 | 190190 | 117121 | 113113 | 155195 | 276300 |
| TW | 162190 | 186206 | 105125 | 98115 | 155199 | 220300 |
| TW | 148160 | 170194 | 105117 | 150174 | 195211 | 224300 |
| TW | 144148 | 178218 | 117125 | 134150 | 155155 | 284308 |
| TW | 160168 | 186194 | 109121 | 130135 | 151171 | 160308 |
| TW | 148172 | 170194 | 101113 | 139172 | 139219 | 222308 |
| TW | 138152 | 170182 | 113113 | 113223 | 147167 | 204312 |
| TW | 116168 | 198214 | 105109 | 101130 | 171183 | 284312 |
| TW | 160164 | 162190 | 109109 | 109155 | 131147 | 215316 |
| TW | 152156 | 182206 | 97113 | 155189 | 155191 | 250328 |
| TW | 156164 | 166174 | 109129 | 136140 | 179199 | 216332 |
| TW | 140168 | 170178 | 85109 | 113164 | 139195 | 220394 |
| TW | 160172 | 158174 | 101109 | 143166 | 163191 | 204398 |
| TW | 186206 | 194206 | 109117 | 122163 | 147175 | 184192 |
| TW | 184188 | 174214 | 109109 | 113134 | 175183 | 192192 |
| TW | 116154 | 174182 | 109109 | 123147 | 151159 | 160200 |
| TW | 164222 | 174174 | 113121 | 143171 | 139143 | 208208 |
| TW | 148182 | 190202 | 109117 | 117121 | 151151 | 180212 |
| TW | 136162 | 178198 | 121121 | 122122 | 123191 | 204212 |
| TW | 156166 | 162174 | 109117 | 131164 | 163163 | 212216 |
| TW | 128160 | 166170 | 101105 | 109155 | 167191 | 216216 |
| TW | 196214 | 202210 | 109121 | 102106 | 131143 | 226226 |
| TW | 116160 | 166202 | 105121 | 106121 | 151151 | 216226 |
| TW | 156220 | 182190 | 97109 | 159206 | 183215 | 180228 |
| TW | 160180 | 174182 | 109117 | 134154 | 131163 | 216230 |
| TW | 156176 | 158194 | 93117 | 127150 | 147163 | 230230 |
| TW | 152192 | 192182 | 113117 | 109133 | 155163 | 164231 |
| TW | 168186 | 166170 | 105121 | 119123 | 151155 | 160234 |
| TW | 172184 | 198202 | 121129 | 130159 | 139155 | 176234 |
| TW | 140209 | 182194 | 105109 | 149167 | 135175 | 176234 |
| TW | 116176 | 162182 | 109109 | 121140 | 171187 | 226235 |
| TW | 156214 | 166170 | 117125 | 110130 | 135191 | 160235 |
| TW | 148152 | 170198 | 101109 | 183183 | 155199 | 180236 |
| TW | 168176 | 178202 | 117121 | 106119 | 119223 | 216244 |
| TW | 136152 | 162182 | 101125 | 105137 | 163171 | 204246 |
| TW | 132204 | 162174 | 109121 | 136167 | 179211 | 228246 |
| TW | 116196 | 162202 | 97109 | 128138 | 147191 | 250250 |
| TW | 152198 | 162174 | 105121 | 123127 | 155171 | 196252 |
| TW | 124148 | 170178 | 105117 | 106176 | 131179 | 204252 |
| TW | 156204 | 162162 | 105133 | 175175 | 167179 | 212256 |
| TW | 156226 | 170186 | 101125 | 111136 | 131211 | 256256 |
| TW | 148148 | 194202 | 121133 | 113138 | 139159 | 224260 |
| TW | 200240 | 182182 | 109117 | 107171 | 175187 | 215260 |
| TW | 176204 | 158170 | 113121 | 109166 | 167179 | 230264 |
| TW | 160168 | 186226 | 105113 | 148187 | 155183 | 234264 |
| TW | 152202 | 182194 | 109133 | 135166 | 127171 | 180268 |
| TW | 182184 | 166182 | 133137 | 118154 | 139171 | 196272 |
| TW | 176194 | 166190 | 105117 | 122151 | 143155 | 228280 |
| TW | 132194 | 170190 | 109109 | 138194 | 135143 | 280284 |
| TW | 116196 | 170182 | 105109 | 110126 | 131155 | 225284 |
| TW | 136170 | 186190 | 101121 | 130151 | 163167 | 172292 |
| TW | 154204 | 186194 | 109113 | 111126 | 155171 | 270292 |
| TW | 144164 | 182190 | 105109 | 127127 | 191191 | 216296 |
| TW | 128172 | 182186 | 101129 | 135192 | 163207 | 216296 |
| TW | 184218 | 166190 | 105109 | 150151 | 115163 | 242300 |
| TW | 116160 | 162190 | 125129 | 151182 | 147179 | 258300 |
| TW | 218218 | 166178 | 113117 | 109131 | 171179 | 212304 |
| TW | 164202 | 166186 | 101105 | 118130 | 167187 | 300304 |
| TW | 140168 | 186194 | 117121 | 118183 | 155179 | 296316 |
| TW | 152200 | 178186 | 113125 | 118143 | 151167 | 180320 |
| TW | 168168 | 162210 | 101113 | 98122 | 171171 | 288320 |
| TW | 178180 | 178190 | 105109 | 101125 | 151191 | 316320 |
| TW | 162172 | 182186 | 101117 | 126143 | 143151 | 231324 |
| TW | 160160 | 162190 | 101109 | 151151 | 151155 | 284384 |
| TW | 190232 | 166194 | 109121 | 107163 | 167175 | 264388 |
| TW | 186196 | 182186 | 97105 | 123154 | 159219 | 296388 |
| TW | 162180 | 170210 | 113121 | 131154 | 155163 | 292396 |
| TW | 164214 | 174198 | 117141 | 123167 | 147159 | 176176 |
| TW | 142168 | 166194 | 97097 | 115138 | 127207 | 180188 |
| TW | 172276 | 170190 | 125141 | 107137 | 147203 | 192192 |
| TW | 132168 | 174202 | 101113 | 138175 | 143147 | 168204 |
| TW | 116152 | 170186 | 105117 | 122183 | 127163 | 160204 |
| TW | 164164 | 194198 | 113125 | 118118 | 171175 | 172204 |
| TW | 172194 | 174174 | 105121 | 103130 | 167183 | 172204 |
| TW | 216216 | 190210 | 113113 | 139167 | 123211 | 200208 |
| TW | 178190 | 170178 | 109133 | 121123 | 167183 | 208211 |
| TW | 188238 | 162198 | 117133 | 106150 | 139151 | 212212 |
| TW | 210232 | 178190 | 105117 | 119203 | 159167 | 184212 |
| TW | 144176 | 178218 | 105117 | 106106 | 147179 | 192212 |
| TW | 116148 | 162202 | 109121 | 144180 | 167179 | 212215 |
| TW | 138176 | 166178 | 105113 | 126159 | 123187 | 216216 |
| TW | 144160 | 174182 | 117121 | 139151 | 131159 | 172220 |
| TW | 148214 | 182214 | 105133 | 147151 | 143239 | 168220 |
| TW | 180214 | 162170 | 117117 | 114154 | 143203 | 184222 |
| TW | 156160 | 170190 | 117121 | 119150 | 167167 | 208226 |
| TW | 164164 | 190202 | 101113 | 115159 | 183255 | 200226 |
| TW | 80168 | 166186 | 109129 | 113159 | 163187 | 220228 |
| TW | 144156 | 170186 | 113121 | 118141 | 147163 | 168230 |
| TW | 148178 | 174194 | 113117 | 131151 | 139167 | 230230 |
| TW | 120172 | 186198 | 121141 | 119147 | 123175 | 230230 |
| TW | 164168 | 162178 | 101117 | 153160 | 155179 | 212230 |
| TW | 144144 | 186194 | 101113 | 102186 | 159207 | 180230 |
| TW | 172176 | 162186 | 109113 | 163175 | 131215 | 196234 |
| TW | 140164 | 170194 | 117121 | 118121 | 143183 | 180236 |
| TW | 172176 | 162166 | 97105 | 155171 | 143183 | 229239 |
| TW | 156160 | 166190 | 121125 | 115154 | 151179 | 172248 |
| TW | 160168 | 166198 | 109137 | 167218 | 159167 | 224250 |
| TW | 164206 | 170170 | 109129 | 110130 | 163175 | 172252 |
| TW | 154172 | 182190 | 109121 | 101101 | 135159 | 234254 |
| TW | 152198 | 186202 | 125125 | 119147 | 147163 | 160258 |
| TW | 152186 | 190194 | 105109 | 163258 | 139163 | 208280 |
| TW | 144156 | 158194 | 113137 | 142147 | 143187 | 258280 |
| TW | 186194 | 174206 | 125133 | 107118 | 143187 | 272280 |
| TW | 164184 | 174198 | 109117 | 147154 | 171211 | 248288 |
| TW | 152168 | 166186 | 121129 | 140143 | 147159 | 238292 |
| TW | 156210 | 166194 | 109133 | 134138 | 143167 | 258292 |
| TW | 168174 | 190190 | 117121 | 155155 | 139147 | 226296 |
| TW | 132160 | 194222 | 101105 | 114115 | 147151 | 164300 |
| TW | 148148 | 214214 | 113117 | 130144 | 131167 | 212300 |
| TW | 72218 | 194202 | 117137 | 147163 | 159171 | 280300 |
| TW | 168174 | 186186 | 113117 | 100107 | 179179 | 240300 |
| TW | 116164 | 186190 | 85113 | 155159 | 135191 | 226300 |
| TW | 156238 | 174226 | 97117 | 99099 | 175191 | 292300 |
| TW | 202218 | 170210 | 109121 | 154167 | 135143 | 260304 |
| TW | 152176 | 194226 | 97109 | 115123 | 135135 | 180308 |
| TW | 155202 | 166182 | 113121 | 136183 | 123151 | 280308 |
| TW | 144144 | 174218 | 109125 | 118200 | 163163 | 284308 |
| TW | 172206 | 174222 | 101113 | 131146 | 151179 | 188312 |
| TW | 168178 | 194206 | 113117 | 111125 | 147163 | 280316 |
| TW | 180190 | 170190 | 109121 | 117146 | 151163 | 308324 |
| TW | 214260 | 194210 | 105113 | 115147 | 143171 | 280324 |
| TW | 152178 | 170182 | 113121 | 118127 | 171175 | 216328 |
| TW | 160188 | 158158 | 113125 | 111183 | 135163 | 248332 |
| TW | 152170 | 162206 | 105113 | 123178 | 151183 | 196340 |
| TW | 156268 | 162174 | 109113 | 130185 | 131179 | 292344 |
| TW | 172206 | 158186 | 113137 | 142151 | 171211 | 208387 |
| TW | 116164 | 174210 | 117141 | 163171 | 159215 | 204391 |
| TW | 168172 | 162194 | 105117 | 138154 | 167175 | 216395 |
| UB | 156160 | 158206 | 109113 | 108152 | 171175 | 192196 |
| UB | 164192 | 166174 | 109109 | 130159 | 143195 | 176196 |
| UB | 148206 | 170202 | 113117 | 105105 | 131179 | 200212 |
| UB | 148160 | 166166 | 101129 | 130147 | 155167 | 220220 |
| UB | 180196 | 174214 | 109129 | 147181 | 123187 | 204220 |
| UB | 152152 | 166170 | 121129 | 109160 | 163211 | 204220 |
| UB | 132194 | 166166 | 109129 | 105134 | 131139 | 224224 |
| UB | 170184 | 170174 | 105121 | 140160 | 135163 | 168224 |
| UB | 198202 | 174206 | 117117 | 117162 | 151191 | 184224 |
| UB | 156226 | 178182 | 105121 | 148151 | 143207 | 212224 |
| UB | 148162 | 182198 | 113129 | 105170 | 155227 | 200226 |
| UB | 128156 | 170178 | 101121 | 121140 | 135139 | 212228 |
| UB | 116132 | 198234 | 125129 | 125140 | 175187 | 212228 |
| UB | 152160 | 182222 | 101113 | 125159 | 179191 | 204228 |
| UB | 156168 | 170202 | 109125 | 117139 | 199199 | 160228 |
| UB | 132152 | 178186 | 113113 | 144152 | 151211 | 188228 |
| UB | 164226 | 202206 | 113149 | 117121 | 155167 | 216230 |
| UB | 144164 | 178190 | 113121 | 109109 | 171175 | 176230 |
| UB | 116156 | 162170 | 97113 | 100148 | 147191 | 188230 |
| UB | 152196 | 166174 | 105113 | 146176 | 163175 | 180232 |
| UB | 160168 | 166174 | 113117 | 105148 | 147203 | 160232 |
| UB | 156168 | 166206 | 105121 | 105113 | 143155 | 230240 |
| UB | 160176 | 194198 | 109117 | 152172 | 143183 | 232240 |
| UB | 172182 | 162178 | 109117 | 113160 | 147171 | 228244 |
| UB | 168190 | 166182 | 109121 | 172184 | 167211 | 236244 |
| UB | 140164 | 190206 | 101113 | 113174 | 139179 | 188246 |
| UB | 120210 | 182214 | 117121 | 105117 | 139159 | 184248 |
| UB | 150158 | 162170 | 101121 | 117124 | 163171 | 208248 |
| UB | 186186 | 174202 | 105105 | 152160 | 183195 | 240248 |
| UB | 164184 | 162178 | 109113 | 96188 | 171171 | 196250 |
| UB | 156176 | 162162 | 109113 | 121168 | 159167 | 220252 |
| UB | 156174 | 162178 | 109109 | 108156 | 167175 | 180252 |
| UB | 82214 | 190194 | 113117 | 109159 | 167175 | 236252 |
| UB | 148168 | 174174 | 113113 | 152180 | 183187 | 168256 |
| UB | 148152 | 186202 | 117121 | 109144 | 147155 | 208260 |
| UB | 168196 | 166178 | 121121 | 122142 | 155195 | 236260 |
| UB | 156172 | 186186 | 105117 | 105138 | 151155 | 238264 |
| UB | 180222 | 206206 | 109117 | 142164 | 171179 | 232264 |
| UB | 140214 | 170182 | 105113 | 129160 | 167191 | 196264 |
| UB | 172180 | 186202 | 109129 | 129137 | 159167 | 168268 |
| UB | 148160 | 186198 | 113117 | 108166 | 163171 | 168268 |
| UB | 134206 | 178182 | 113117 | 113171 | 147179 | 234268 |
| UB | 152178 | 158170 | 109121 | 113168 | 155183 | 268268 |
| UB | 132202 | 162170 | 109117 | 138163 | 159191 | 268268 |
| UB | 148178 | 182186 | 113113 | 125125 | 187207 | 220268 |
| UB | 202202 | 158186 | 121125 | 117180 | 143167 | 232272 |
| UB | 170186 | 174194 | 117121 | 129137 | 179187 | 236272 |
| UB | 160182 | 170190 | 109117 | 105230 | 163175 | 184274 |
| UB | 116116 | 190202 | 101117 | 146176 | 159183 | 221274 |
| UB | 120152 | 194202 | 109121 | 100122 | 151151 | 172276 |
| UB | 171172 | 190222 | 105125 | 125158 | 171207 | 256276 |
| UB | 156156 | 210214 | 109125 | 105152 | 155199 | 248278 |
| UB | 132152 | 190190 | 109125 | 170180 | 135151 | 276280 |
| UB | 178202 | 166174 | 117121 | 124136 | 167183 | 272280 |
| UB | 140180 | 182190 | 101109 | 171172 | 135183 | 240284 |
| UB | 152198 | 178194 | 109125 | 140192 | 139203 | 264286 |
| UB | 156160 | 162166 | 117117 | 180184 | 155155 | 224288 |
| UB | 124152 | 190198 | 101149 | 134143 | 159183 | 192288 |
| UB | 156180 | 162186 | 97101 | 134140 | 139219 | 254288 |
| UB | 140164 | 174218 | 125129 | 105130 | 139235 | 268288 |
| UB | 144198 | 170186 | 113117 | 125129 | 163179 | 288292 |
| UB | 116116 | 190202 | 113125 | 152170 | 155195 | 226292 |
| UB | 116164 | 162166 | 109113 | 121153 | 147159 | 268296 |
| UB | 160194 | 162186 | 105121 | 140140 | 131171 | 292296 |
| UB | 152182 | 178186 | 101117 | 81138 | 187195 | 224296 |
| UB | 164196 | 198210 | 113117 | 129138 | 131163 | 220300 |
| UB | 144174 | 162178 | 109117 | 143143 | 147167 | 196300 |
| UB | 168168 | 186190 | 117121 | 121143 | 159167 | 240300 |
| UB | 144168 | 162190 | 101101 | 136144 | 155175 | 180300 |
| UB | 172178 | 178190 | 117129 | 120168 | 151175 | 240300 |
| UB | 172222 | 194206 | 101121 | 113124 | 155187 | 280300 |
| UB | 159168 | 182190 | 105117 | 126140 | 147151 | 236304 |
| UB | 148152 | 166174 | 117125 | 142176 | 139151 | 296304 |
| UB | 148156 | 170206 | 109117 | 129162 | 147163 | 300304 |
| UB | 170206 | 170194 | 97121 | 138152 | 147171 | 180304 |
| UB | 174246 | 182186 | 125129 | 152188 | 187199 | 240304 |
| UB | 152166 | 174202 | 105117 | 130168 | 143167 | 216308 |
| UB | 148190 | 166166 | 121129 | 122176 | 143171 | 236308 |
| UB | 164168 | 174174 | 113121 | 159164 | 167183 | 292308 |
| UB | 180180 | 166166 | 109129 | 125164 | 155159 | 212312 |
| UB | 116182 | 166170 | 113121 | 105160 | 123163 | 200312 |
| UB | 136152 | 162170 | 109109 | 105136 | 187187 | 220312 |
| UB | 162228 | 182198 | 105121 | 92154 | 135139 | 212316 |
| UB | 140188 | 182186 | 117121 | 152175 | 167167 | 238316 |
| UB | 168182 | 174178 | 101117 | 117121 | 171199 | 216332 |
| UB | 152160 | 174198 | 105105 | 128132 | 131159 | 234352 |
| UB | 136160 | 178186 | 109121 | 135200 | 155171 | 212360 |
| UB | 132160 | 178178 | 109113 | 152170 | 159159 | 292380 |
| UB | 144168 | 182190 | 97133 | 146146 | 163183 | 288380 |
| UB | 156156 | 170190 | 101109 | 121159 | 147147 | 288384 |
| UB | 176180 | 174198 | 113121 | 140163 | 139163 | 228384 |
| UB | 152170 | 186198 | 113113 | 112126 | 163163 | 380384 |
| UB | 160222 | 182202 | 105129 | 129180 | 151167 | 196384 |
| UB | 160182 | 162194 | 109109 | 104130 | 159191 | 180384 |
| UB | 160186 | 166166 | 117121 | 164200 | 183199 | 380384 |
| UB | 164172 | 166206 | 109113 | 111133 | 127167 | 170186 |
| UB | 152164 | 166178 | 109121 | 115122 | 167183 | 178194 |
| UB | 114156 | 186206 | 113113 | 134159 | 187199 | 162200 |
| UB | 148156 | 190206 | 93097 | 114174 | 179179 | 180204 |
| UB | 156180 | 170198 | 105109 | 133158 | 139143 | 188206 |
| UB | 148172 | 186190 | 109125 | 143158 | 135183 | 188207 |
| UB | 216216 | 174174 | 105121 | 104117 | 139175 | 158208 |
| UB | 114118 | 170206 | 101117 | 116142 | 127207 | 170208 |
| UB | 120180 | 158166 | 129129 | 114189 | 171175 | 196212 |
| UB | 148160 | 170178 | 109113 | 113159 | 127191 | 212212 |
| UB | 148152 | 194206 | 101121 | 90123 | 147163 | 162216 |
| UB | 180204 | 174186 | 113125 | 107121 | 159175 | 216216 |
| UB | 128152 | 178194 | 105117 | 107134 | 139171 | 220220 |
| UB | 176196 | 190194 | 105105 | 130142 | 171175 | 214222 |
| UB | 156222 | 174186 | 109109 | 146162 | 155155 | 174224 |
| UB | 160168 | 162178 | 105117 | 118156 | 159171 | 208224 |
| UB | 140148 | 174178 | 109133 | 94103 | 171175 | 216224 |
| UB | 164168 | 162162 | 105125 | 132146 | 159183 | 216224 |
| UB | 160172 | 174178 | 101101 | 128160 | 147155 | 216228 |
| UB | 152164 | 166170 | 117121 | 159182 | 131183 | 170228 |
| UB | 152186 | 162182 | 109113 | 107119 | 143211 | 211228 |
| UB | 148172 | 162162 | 105117 | 151166 | 167207 | 212230 |
| UB | 208212 | 182198 | 105105 | 115146 | 147159 | 228232 |
| UB | 144152 | 162170 | 113117 | 130147 | 155167 | 200232 |
| UB | 186208 | 162178 | 113113 | 151151 | 123171 | 200232 |
| UB | 188204 | 166182 | 113121 | 108121 | 139143 | 230234 |
| UB | 148168 | 194210 | 117137 | 104180 | 171207 | 200234 |
| UB | 132156 | 166198 | 101125 | 142143 | 155191 | 160241 |
| UB | 156172 | 170170 | 105105 | 115156 | 167175 | 230242 |
| UB | 124180 | 186194 | 105109 | 119135 | 171179 | 230242 |
| UB | 152212 | 178190 | 117133 | 134176 | 183195 | 182242 |
| UB | 182234 | 202206 | 101129 | 133158 | 115167 | 176248 |
| UB | 116152 | 162182 | 105113 | 134170 | 143167 | 184249 |
| UB | 80180 | 178210 | 105117 | 112130 | 135171 | 220250 |
| UB | 218218 | 198202 | 109113 | 135151 | 187203 | 245250 |
| UB | 148182 | 158186 | 97097 | 147155 | 151155 | 182252 |
| UB | 152244 | 162170 | 109117 | 99103 | 163195 | 224256 |
| UB | 124144 | 174178 | 113121 | 151174 | 135143 | 224260 |
| UB | 116193 | 170210 | 105121 | 121126 | 143167 | 262262 |
| UB | 148190 | 190190 | 121129 | 142159 | 115159 | 174264 |
| UB | 140160 | 186186 | 105109 | 104108 | 139171 | 224264 |
| UB | 144151 | 182190 | 109117 | 102108 | 163171 | 196266 |
| UB | 168198 | 162166 | 101121 | 136155 | 171183 | 266266 |
| UB | 136172 | 178182 | 109129 | 131134 | 119171 | 200268 |
| UB | 132172 | 182202 | 101145 | 146155 | 175187 | 212268 |
| UB | 152152 | 174222 | 109109 | 143159 | 163195 | 180272 |
| UB | 172184 | 166206 | 105109 | 174204 | 143151 | 234276 |
| UB | 140164 | 190194 | 105109 | 133133 | 183199 | 187276 |
| UB | 160218 | 166198 | 101113 | 125125 | 143171 | 208284 |
| UB | 116172 | 190206 | 85109 | 106146 | 151187 | 222284 |
| UB | 152176 | 206210 | 101117 | 110130 | 171215 | 222284 |
| UB | 144172 | 170174 | 113117 | 107159 | 155163 | 158288 |
| UB | 168336 | 166186 | 113129 | 132143 | 163175 | 220288 |
| UB | 172198 | 178186 | 109117 | 94114 | 159199 | 216288 |
| UB | 116124 | 198206 | 113121 | 111111 | 167175 | 260292 |
| UB | 208226 | 166210 | 121141 | 122138 | 143187 | 292292 |
| UB | 178184 | 170218 | 109117 | 139144 | 191191 | 238292 |
| UB | 156172 | 170210 | 117121 | 143155 | 143199 | 186292 |
| UB | 140186 | 162162 | 101125 | 126126 | 195203 | 210292 |
| UB | 140148 | 174198 | 113121 | 138143 | 115163 | 292296 |
| UB | 114160 | 162162 | 105113 | 134148 | 157171 | 220296 |
| UB | 148156 | 158198 | 109133 | 114151 | 147171 | 224296 |
| UB | 114172 | 162178 | 125137 | 100150 | 163175 | 178296 |
| UB | 152188 | 174202 | 113113 | 123228 | 171175 | 288296 |
| UB | 114192 | 170182 | 117125 | 108138 | 147207 | 296296 |
| UB | 140188 | 166178 | 101109 | 116121 | 143171 | 280300 |
| UB | 168200 | 162162 | 121125 | 118158 | 171175 | 204300 |
| UB | 116204 | 162186 | 105117 | 130178 | 163183 | 202300 |
| UB | 212230 | 178186 | 105145 | 102129 | 155183 | 248300 |
| UB | 144230 | 178194 | 109117 | 138170 | 171191 | 180300 |
| UB | 116180 | 182222 | 105133 | 126160 | 159191 | 292300 |
| UB | 116136 | 178182 | 105121 | 114122 | 147163 | 182304 |
| UB | 144168 | 174182 | 105113 | 102178 | 155171 | 232304 |
| UB | 114208 | 162182 | 109113 | 125125 | 175211 | 196304 |
| UB | 148156 | 174194 | 97105 | 107147 | 163231 | 212304 |
| UB | 160192 | 154206 | 109117 | 130150 | 135143 | 308308 |
| UB | 144156 | 170170 | 101145 | 124124 | 139175 | 248308 |
| UB | 164200 | 166206 | 109113 | 116129 | 179223 | 212308 |
| UB | 148174 | 170202 | 97109 | 164170 | 175199 | 198312 |
| UB | 172208 | 166194 | 109113 | 154185 | 139175 | 288316 |
| UB | 172196 | 158186 | 109117 | 117146 | 143183 | 240316 |
| UB | 144152 | 166178 | 117121 | 155155 | 151195 | 224316 |
| UB | 156156 | 178206 | 105113 | 120174 | 139183 | 204328 |
| UB | 144184 | 182194 | 105109 | 155172 | 143227 | 200328 |
| UB | 152204 | 162190 | 113121 | 111130 | 147159 | 266336 |
| UB | 116152 | 158218 | 101109 | 107116 | 179211 | 212340 |
| UB | 144200 | 174198 | 105125 | 107150 | 159183 | 228392 |
| UB | 156186 | 186194 | 133145 | 134134 | 171223 | 222394 |
| UB | 178214 | 170210 | 121125 | 102114 | 139143 | 168184 |
| UB | 144214 | 166170 | 113133 | 139142 | 119175 | 188188 |
| UB | 160208 | 170186 | 125129 | 106235 | 147151 | 180204 |
| UB | 195204 | 166186 | 113113 | 131211 | 139167 | 188204 |
| UB | 160176 | 186190 | 109121 | 115171 | 147175 | 184204 |
| UB | 144182 | 162206 | 109121 | 94106 | 131171 | 184220 |
| UB | 164164 | 162170 | 117129 | 115151 | 135199 | 220220 |
| UB | 176212 | 162202 | 105117 | 111113 | 163211 | 164220 |
| UB | 164194 | 166170 | 101113 | 119170 | 147155 | 172224 |
| UB | 152160 | 174210 | 97101 | 126167 | 163195 | 168224 |
| UB | 116180 | 166174 | 97113 | 147174 | 127167 | 172226 |
| UB | 208212 | 158194 | 105109 | 105130 | 139139 | 184230 |
| UB | 160164 | 178202 | 117129 | 114143 | 139199 | 180230 |
| UB | 152164 | 166182 | 109121 | 102113 | 163203 | 221233 |
| UB | 144144 | 162178 | 105105 | 96171 | 167195 | 212234 |
| UB | 164220 | 178186 | 105121 | 109167 | 151179 | 236236 |
| UB | 117117 | 178202 | 109109 | 116162 | 159159 | 225238 |
| UB | 152166 | 190190 | 109113 | 102154 | 175195 | 204238 |
| UB | 148152 | 170186 | 113117 | 98118 | 139167 | 210241 |
| UB | 156180 | 190190 | 113129 | 115163 | 151195 | 230241 |
| UB | 190194 | 178198 | 105125 | 102143 | 167187 | 192244 |
| UB | 136176 | 218218 | 109125 | 127178 | 143151 | 230250 |
| UB | 152172 | 166210 | 105109 | 142165 | 131163 | 184252 |
| UB | 178180 | 174174 | 101121 | 127158 | 139179 | 220254 |
| UB | 166166 | 166178 | 109109 | 158162 | 147167 | 180256 |
| UB | 152168 | 170182 | 113125 | 113207 | 155175 | 188256 |
| UB | 156158 | 162178 | 101105 | 162178 | 163215 | 164256 |
| UB | 156164 | 166186 | 101117 | 118146 | 179191 | 164264 |
| UB | 178200 | 162186 | 113113 | 114142 | 171175 | 219266 |
| UB | 116152 | 174206 | 105109 | 139143 | 163167 | 234268 |
| UB | 147182 | 182190 | 97125 | 111142 | 151175 | 260272 |
| UB | 132144 | 198202 | 81105 | 151156 | 143159 | 238276 |
| UB | 148176 | 178194 | 105129 | 178200 | 131207 | 228276 |
| UB | 160186 | 166206 | 101117 | 131139 | 139179 | 208280 |
| UB | 148184 | 170178 | 109113 | 135170 | 123139 | 188284 |
| UB | 158216 | 166186 | 101109 | 105122 | 143175 | 280288 |
| UB | 152180 | 154182 | 93125 | 126145 | 131143 | 176296 |
| UB | 152156 | 170186 | 109125 | 147178 | 147155 | 188296 |
| UB | 172216 | 194202 | 121137 | 115122 | 143171 | 216296 |
| UB | 202222 | 166186 | 113125 | 139167 | 147179 | 192296 |
| UB | 156164 | 170170 | 105117 | 130146 | 159191 | 184296 |
| UB | 160164 | 162162 | 105109 | 113143 | 159191 | 176304 |
| UB | 128162 | 182214 | 109121 | 125135 | 155167 | 220308 |
| UB | 164184 | 174190 | 105109 | 137142 | 131171 | 272320 |
| UB | 164186 | 166174 | 93129 | 147161 | 171179 | 292320 |
| UB | 184196 | 186186 | 109113 | 145150 | 175191 | 272340 |
| UB | 152202 | 182194 | 117137 | 140186 | 135163 | 168392 |

Allelic sizes (xxx base pairs) at six microsatellite loci in capelin collected at 15 locations (sample codes in Table 1 of the publication) including samples from 3 locations that were found to be temporally distinct.
