# Supplementary material for: Genetic Structure of Capelin (Mallotus villosus) in the Northwest Atlantic Ocean
Source: PLoS One. 2015 Mar 30;10(3):e0122315. doi: 10.1371/journal.pone.0122315 (PMC4378951; doi:10.1371/journal.pone.0122315)
Supplement: S3 Table — Details of capelin at 6 loci (Mvi) across 18 samples (see Table 1 of the publication for codes) with varying number of individuals in a sample (N) and associated genetic diversity summary statistics that include the number of alleles observed (NA) for each locus, allelic richness (RS) for each locus, the number of private alleles (PS) based on a minimum sample size of 38 individuals, expected heterozygosity (H E), observed heterozygosity (H O) and the inbreeding coefficient (F IS). SD = standard deviation. (DOCX) [file pone.0122315.s005.docx]

**S3 Table. Summary Statistics of Capelin at 6 Loci across 18 Samples Used for Genetic Analyses.**

|  | **Samples** | | | | | | | | | | | | | | | | | |
| --- | --- | --- | --- | --- | --- | --- | --- | --- | --- | --- | --- | --- | --- | --- | --- | --- | --- | --- |
|  | **BB** | **BB61** | **BB65** | **CC** | **2005 CC** | **DRL** | **GSL** | **LL** | **2004 LL** | **RBB** | **SES** | **SLL** | **SR** | **SS** | **SV** | **2005 SV** | **TW** | **UB** |
| N | 654 | 128 | 98 | 480 | 50 | 88 | 122 | 285 | 87 | 186 | 165 | 98 | 62 | 109 | 260 | 39 | 272 | 230 |
| N_A_ *Mv*i2 | 91 | 53 | 54 | 85 | 33 | 48 | 48 | 66 | 45 | 62 | 65 | 51 | 41 | 51 | 68 | 35 | 71 | 64 |
| N_A_ *Mvi*3 | 23 | 20 | 17 | 24 | 17 | 17 | 19 | 24 | 19 | 20 | 22 | 18 | 19 | 22 | 21 | 16 | 22 | 19 |
| N_A_ *Mvi*5 | 19 | 15 | 14 | 19 | 12 | 11 | 14 | 18 | 16 | 13 | 14 | 14 | 11 | 14 | 14 | 12 | 17 | 17 |
| N_A_ *Mvi*9 | 124 | 78 | 72 | 115 | 41 | 61 | 75 | 100 | 55 | 87 | 81 | 73 | 63 | 74 | 94 | 38 | 102 | 95 |
| N_A_ *Mvi*10 | 34 | 26 | 27 | 34 | 24 | 25 | 30 | 29 | 25 | 28 | 30 | 24 | 24 | 28 | 32 | 23 | 33 | 32 |
| N_A_ *Mvi*16 | 115 | 64 | 69 | 114 | 44 | 72 | 76 | 85 | 55 | 84 | 82 | 74 | 47 | 74 | 85 | 38 | 92 | 88 |
| R_S_ *Mvi2* | 14.1 | 14.0 | 14.8 | 13.8 | 13.6 | 14.3 | 13.3 | 14.1 | 14.2 | 14.2 | 14.3 | 13.5 | 13. 7 | 14.2 | 13.9 | 15.0 | 14.1 | 14.2 |
| R_S_ *Mvi*3 | 10.0 | 10.4 | 9.9 | 10.0 | 9.9 | 9.6 | 10.1 | 10.0 | 10.2 | 10.2 | 9.9 | 9.8 | 10.1 | 10.4 | 10.0 | 10.3 | 10.1 | 9.87 |
| R_S_ *Mvi*5 | 7.6 | 7.8 | 7.4 | 7.7 | 7.5 | 7.4 | 7.6 | 7.5 | 7.5 | 7.5 | 7.5 | 7.8 | 7.0 | 7.4 | 7.3 | 7.9 | 7.8 | 7.7 |
| R_S_ *Mvi*9 | 17.2 | 17.0 | 16.5 | 17.1 | 14.9 | 16.3 | 16.7 | 17.3 | 15.7 | 17.4 | 16.5 | 16.6 | 17.1 | 16.7 | 16.9 | 15.3 | 17.0 | 17.3 |
| R_S_ *Mvi*10 | 11.9 | 11.7 | 11.7 | 11.8 | 11.5 | 11.4 | 11.9 | 12.1 | 11.7 | 11.7 | 12.2 | 11.6 | 11.4 | 11.7 | 11.8 | 11.6 | 12.0 | 12.1 |
| R_S_ *Mvi*16 | 16.4 | 16.0 | 16.8 | 16.6 | 15.5 | 17.1 | 16.95 | 16.8 | 15.7 | 16.4 | 16.7 | 17.1 | 15.9 | 16.9 | 16.3 | 15.5 | 16.5 | 16.7 |
| R_S_ All | 12.9 | 12.8 | 12.8 | 12.9 | 12.2 | 12.7 | 12.8 | 13.0 | 12.5 | 12.9 | 12.8 | 12.8 | 12.5 | 12.9 | 12.7 | 12.6 | 12.9 | 13.0 |
| P_S_ *Mv*i2 | 1.08 | 0.89 | 1.53 | 1.17 | 1.43 | 1.85 | 0.70 | 0.96 | 0.91 | 1.08 | 1.21 | 1.01 | 0.69 | 0.87 | 1.19 | 1.85 | 1.11 | 0.99 |
| P_S_ *Mvi*3 | 0.18 | 0.24 | 0.04 | 0.19 | 0.04 | 0.08 | 0.19 | 0.21 | 0.20 | 0.16 | 0.25 | 0.24 | 0.26 | 0.32 | 0.14 | 0.20 | 0.22 | 0.11 |
| P_S_ *Mvi*5 | 0.15 | 0.10 | 0.12 | 0.21 | 0.07 | 0.05 | 0.09 | 0.18 | 0.27 | 0.08 | 0.13 | 0.15 | 0.03 | 0.07 | 0.07 | 0.11 | 0.21 | 0.20 |
| P_S_ *Mvi*9 | 1.59 | 1.32 | 1.64 | 1.48 | 1.83 | 1.30 | 1.48 | 1.40 | 1.53 | 1.57 | 1.37 | 1.57 | 2.02 | 1.58 | 1.31 | 0.84 | 1.46 | 1.51 |
| P_S_ *Mvi*10 | 0.33 | 0.19 | 0.25 | 0.24 | 0.38 | 0.31 | 0.38 | 0.25 | 0.25 | 0.16 | 0.28 | 0.12 | 0.11 | 0.21 | 0.30 | 0.20 | 0.35 | 0.30 |
| P_S_ *Mvi*16 | 1.33 | 1.16 | 1.89 | 1.63 | 1.03 | 1.89 | 1.74 | 1.40 | 0.75 | 1.34 | 1.52 | 2.10 | 1.36 | 1.86 | 1.24 | 1.26 | 1.40 | 1.55 |
| P_S_ All | 0.77 | 0.65 | 0.91 | 0.82 | 0.80 | 0.91 | 0.76 | 0.73 | 0.65 | 0.73 | 0.79 | 0.86 | 0.75 | 0.82 | 0.71 | 0.74 | 0.79 | 0.78 |
| *H_E_* | 0.95 | 0.95 | 0.94 | 0.95 | 0.94 | 0.94 | 0.94 | 0.95 | 0.94 | 0.95 | 0.95 | 0.95 | 0.94 | 0.95 | 0.94 | 0.95 | 0.95 | 0.95 |
| *H_E_* SD | 0.04 | 0.04 | 0.05 | 0.04 | 0.04 | 0.04 | 0.04 | 0.04 | 0.04 | 0.04 | 0.04 | 0.04 | 0.04 | 0.04 | 0.04 | 0.04 | 0.04 | 0.04 |
| *H_O_* | 0.90 | 0.90 | 0.91 | 0.92 | 0.92 | 0.89 | 0.88 | 0.91 | 0.91 | 0.92 | 0.92 | 0.93 | 0.89 | 0.90 | 0.91 | 0.88 | 0.91 | 0.91 |
| *H_O_* SD | 0.03 | 0.04 | 0.04 | 0.03 | 0.02 | 0.04 | 0.06 | 0.04 | 0.04 | 0.03 | 0.02 | 0.03 | 0.04 | 0.05 | 0.03 | 0.09 | 0.02 | 0.03 |
| *F_IS_* | 0.04 | 0.05 | 0.04 | 0.03 | 0.02 | 0.06 | 0.07 | 0.04 | 0.04 | 0.03 | 0.03 | 0.02 | 0.05 | 0.05 | 0.04 | 0.08 | 0.04 | 0.04 |

Details of capelin at 6 loci (*Mvi*) across 18 samples (see Table 1 of the publication for codes) with varying number of individuals in a sample (*N*) and associated genetic diversity summary statistics that include the number of alleles observed (*N_A_*) for each locus, allelic richness (*R_S_*) for each locus, the number of private alleles (*P_S_*) based on a minimum sample size of 38 individuals, expected heterozygosity (*H_E_*), observed heterozygosity (*H_O_*) and the inbreeding coefficient (*F_IS_*). *SD* = standard deviation.
