# Supplementary material for: Genetic Structure of Capelin (Mallotus villosus) in the Northwest Atlantic Ocean
Source: PLoS One. 2015 Mar 30;10(3):e0122315. doi: 10.1371/journal.pone.0122315 (PMC4378951; doi:10.1371/journal.pone.0122315)
Supplement: S4 Table — Re-sampled indices using a jack-knife on the six loci and 18 samples to produce variance estimates and to evaluate the influence of the selection of loci and/or samples on the mean values of parameters. F IS expresses the variation between individuals within populations; F IT expresses the variation within individuals; F ST θ expresses the variation among populations. Jost’s D est is estimated for each locus (no jack-knife) and overall (Chao’s harmonic mean). 95% confidence intervals were constructed through 1,000 bootstraps for each locus. (DOCX) [file pone.0122315.s006.docx]

**S4 Table. Jack-knifed Weir and Cockerham Fixation Indices and Jost’s *D*_est_.**

| **Jack-knife on Loci**  **(18 Samples) for *F*** | ***F*_IS_** | ***F*_IT_** | ***F*_ST_ *θ*** | ***D*_est_** |
| --- | --- | --- | --- | --- |
| Without locus *Mvi*2 | 0.0402 | 0.0409 | 0.0007 | 0.026 (0.097, 0.144) |
| Without locus *Mvi*3 | 0.0434 | 0.0441 | 0.0008 | 0.000 (0.036, 0.072) |
| Without locus *Mvi*5 | 0.0440 | 0.0447 | 0.0007 | 0.002 (0.020, 0.045) |
| Without locus *Mvi*9 | 0.0347 | 0.0350 | 0.0003 | 0.197 (0.311, 0.376) |
| Without locus *Mvi*10 | 0.0433 | 0.0439 | 0.0007 | 0.009 (0.060, 0.101) |
| Without locus *Mvi*16 | 0.0295 | 0.0302 | 0.0007 | 0.035 (0.221, 0.300) |
| Mean | 0.0394 | 0.0400 | 0.0007 |  |
| S. D. | 0.0120 | 0.0121 | 0.0004 |  |
| Multilocus | 0.0392 | 0.0398 | 0.0007 | 0.012 |
| Bootstrap 95% C.I. | (0.0198, 0.0621) | (0.0200, 0.0632) | (0.0002, 0.0014) |  |

|  | ***F*_ST_ *θ*** | | | | | |
| --- | --- | --- | --- | --- | --- | --- |
| **Jack-knife on Samples** | **Mvi2** | **Mvi3** | **Mvi5** | **Mvi9** | **Mvi10** | **Mvi16** |
| Without Sample BB | 0.00056 | -0.00010 | 0.00029 | 0.00273 | 0.00036 | 0.00049 |
| Without Sample BB61 | 0.00056 | -0.00010 | 0.00015 | 0.00251 | 0.00038 | 0.00042 |
| Without Sample BB65 | 0.00049 | -0.00007 | 0.00009 | 0.00235 | 0.00026 | 0.00044 |
| Without Sample CC | 0.00052 | -0.00003 | 0.00019 | 0.00272 | 0.00043 | 0.00043 |
| Without Sample 2005CC | 0.00035 | -0.00010 | 0.00025 | 0.00224 | 0.00035 | 0.00039 |
| Without Sample DRL | 0.00050 | -0.00013 | 0.00012 | 0.00228 | 0.00026 | 0.00046 |
| Without Sample GSL | 0.00047 | -0.00010 | 0.00028 | 0.00240 | 0.00039 | 0.00043 |
| Without Sample LL | 0.00066 | -0.00005 | 0.00012 | 0.00257 | 0.00033 | 0.00046 |
| Without Sample 2004LL | 0.00056 | -0.00020 | 0.00017 | 0.00215 | 0.00021 | 0.00044 |
| Without Sample RBB | 0.00062 | -0.00002 | 0.00014 | 0.00249 | 0.00043 | 0.00042 |
| Without Sample SES | 0.00058 | -0.00005 | 0.00026 | 0.00226 | 0.00035 | 0.00043 |
| Without Sample SLL | 0.00053 | -0.00009 | 0.00029 | 0.00240 | 0.00037 | 0.00047 |
| Without Sample SR | 0.00051 | -0.00013 | 0.00024 | 0.00241 | 0.00024 | 0.00035 |
| Without Sample SS | 0.00053 | -0.00009 | 0.00005 | 0.00235 | 0.00028 | 0.00046 |
| Without Sample SV | 0.00047 | -0.00014 | 0.00021 | 0.00246 | 0.00033 | 0.00043 |
| Without Sample 2005SV | 0.00053 | -0.00003 | 0.00007 | 0.00228 | 0.00038 | 0.00031 |
| Without Sample TW | 0.00062 | -0.00007 | 0.00001 | 0.00258 | 0.00034 | 0.00048 |
| Without Sample UB | 0.00057 | -0.00013 | 0.00030 | 0.00244 | 0.00024 | 0.00043 |
| Mean | 0.00050 | -0.00010 | 0.00016 | 0.00220 | 0.00029 | 0.00039 |
| S. D. | 0.00027 | 0.00020 | 0.00037 | 0.00064 | 0.00027 | 0.00018 |

Re-sampled indices using a jack-knife on the six loci and 18 samples to produce variance estimates and to evaluate the influence of the selection of loci and/or samples on the mean values of parameters. *F*_IS_ expresses the variation between individuals within populations; *F*_IT_ expresses the variation within individuals; *F*_ST_ *θ* expresses the variation among populations. Jost’s *D*_est_ is estimated for each locus (no jack-knife) and overall (Chao’s harmonic mean). 95% confidence intervals were constructed through 1,000 bootstraps for each locus.
