# Supplementary material for: Genetic Structure of Capelin (Mallotus villosus) in the Northwest Atlantic Ocean
Source: PLoS One. 2015 Mar 30;10(3):e0122315. doi: 10.1371/journal.pone.0122315 (PMC4378951; doi:10.1371/journal.pone.0122315)
Supplement: S5 Table — D est for six microsatellite loci applied to 18 capelin samples. Values calculated using all allele frequencies are compared with those calculated after removing rare alleles (alleles with average frequency across samples of less than 0.05% or 0.01% dependent on the frequency distribution). Confidence intervals were generated through 500 permutations of the data. (DOCX) [file pone.0122315.s007.docx]

**S5 Table.** **Effect of Rare Alleles on *D*_est_.**

| **Locus (Number of Alleles, Number of Alleles ≥ 0.05*)** | ***D*_est_ (95% confidence interval) Calculated using All Alleles** | ***D*_est_ (95% confidence interval) Calculated using Alleles with Average Frequency ≥ 0.05*** |
| --- | --- | --- |
| *Mvi*2 (137, 6) | 0.033 (0.007, 0.059) | 0.013 (0.000, 0.051) |
| *Mvi*3 (36, 10) | 0.005 (0.000, 0.026) | 0.000 (0.000, 0.017) |
| *Mvi*5 (25, 7) | 0.002 (0.000, 0.016) | 0.002 (0.000, 0.016) |
| *Mvi*9 (153, 64)* | 0.222 (0.191, 0.255) | 0.222 (0.187, 0.261)* |
| *Mvi*10 (42, 11) | 0.010 (0.000, 0.033) | 0.014 (0.000, 0.036) |
| *Mvi*16 (158, 52)* | 0.075 (0.042, 0.116) | 0.061 (0.025, 0.100)* |
| Harmonic Mean | .007 | .007 |

*D*_est_ for six microsatellite loci applied to 18 capelin samples. Values calculated using all allele frequencies are compared with those calculated after removing rare alleles (alleles with average frequency across samples of less than 0.05% or 0.01% dependent on the frequency distribution). Confidence intervals were generated through 500 permutations of the data. *All average allele frequencies were less than 0.05 for *Mvi*9 and *Mvi*16 therefore a cut off of 0.01 was used for those loci.
