# Supplementary material for: Genetic Structure of Capelin (Mallotus villosus) in the Northwest Atlantic Ocean
Source: PLoS One. 2015 Mar 30;10(3):e0122315. doi: 10.1371/journal.pone.0122315 (PMC4378951; doi:10.1371/journal.pone.0122315)
Supplement: S6 Table — Summary statistics of N replicates (mean, standard deviation) for the log likelihood of the data (Ln P(D)), the degree of admixture (α) and model fit (r) from Markov chain Monte Carlo (MCMC) data collection chains of 500,000 steps each, run under 1 to 15 assumed populations (K). (DOCX) [file pone.0122315.s008.docx]

**S6 Table. Performance of Bayesian Clustering Models.**

| ***K*** | ***N*** | **Mean *Ln P(D)*** | **Std. Dev. *Ln P(D)*** | **Mean *α*** | **Std. Dev. *α*** | ***r*** | **Std. Dev. *r*** |
| --- | --- | --- | --- | --- | --- | --- | --- |
| 1 | 10 | -133377.46 | 0.20 | - | - | 10.2285 | 2.4631 |
| 2 | 10 | -133981.20 | 669.64 | 0.8555 | 0.3033 | 2.0564 | 0.6527 |
| 3 | 10 | -135374.63 | 742.26 | 0.9774 | 0.3330 | 3.5547 | 0.9436 |
| 4 | 10 | -135546.80 | 1899.72 | 0.7342 | 0.4320 | 4.9377 | 0.9962 |
| 5 | 10 | -136176.82 | 1539.11 | 0.5272 | 0.2825 | 5.4195 | 1.6463 |
| 6 | 10 | -135645.43 | 1531.40 | 0.3998 | 0.1942 | 5.5553 | 1.8026 |
| 7 | 5 | -135551.08 | 862.84 | 0.3327 | 0.1417 | 6.7975 | 1.8782 |
| 8 | 6 | -136452.82 | 1466.78 | 0.2684 | 0.1350 | 6.7728 | 1.0623 |
| 9 | 5 | -138391.26 | 978.99 | 0.3492 | 0.2433 | 6.5385 | 1.7716 |
| 10 | 5 | -136681.70 | 683.82 | 0.1792 | 0.0841 | 7.7767 | 1.0108 |
| 11 | 5 | -138280.18 | 2710.53 | 0.2590 | 0.1405 | 6.6099 | 3.3672 |
| 12 | 5 | -137859.02 | 1959.42 | 0.3542 | 0.1252 | 3.8523 | 1.1342 |
| 13 | 4 | -136563.80 | 1167.82 | 0.2994 | 0.1747 | 8.1456 | 2.3874 |
| 14 | 5 | -137259.20 | 940.24 | 0.3386 | 0.0905 | 5.8205 | 2.5362 |
| 15 | 5 | -139460.14 | 3214.36 | 0.4424 | 0.2003 | 7.2881 | 3.4839 |

Summary statistics of *N* replicates (mean, standard deviation) for the log likelihood of the data (*Ln P(D)*), the degree of admixture (*α*) and model fit (*r*) from Markov chain Monte Carlo (MCMC) data collection chains of 500,000 steps each, run under 1 to 15 assumed populations (*K*).
