# Supplementary material for: Genetic Structure of Capelin (Mallotus villosus) in the Northwest Atlantic Ocean
Source: PLoS One. 2015 Mar 30;10(3):e0122315. doi: 10.1371/journal.pone.0122315 (PMC4378951; doi:10.1371/journal.pone.0122315)
Supplement: S1 Text — (DOCX) [file pone.0122315.s009.docx]

**S1 Text. Quality Control of Data.**

To ensure the assigned sizing of the PCR fragments (scoring) was consistent within and among gels and to address potential sources of genotyping error, we used an external size standard and two types of control sample: master control samples and redundant samples. An external ladder consisting of microsatellite PCR fragments of known size (based on sequencing of the cloned PCR fragments) was run every 16 lanes. The same two master control samples (of known genotype) were included in the extraction, PCR and gel electrophoresis for each 96-well plate of samples and were positioned in the first and last wells on the plate (corresponding to the first and last wells on the gel). These two samples were included specifically to address the concern of gel to gel variation of the migration of the samples with respect to the external size standard and allowed for consistent scoring between gels. These same two master control samples were run on every gel run as part of this study. For reference, the same two master control samples were part of the initial 44 samples run in Gordos et al. [1]. In addition to the two master control samples, on each 96 well extraction, we included internal controls (which we refer to as redundants). The redundants were samples where the tissue sample was divided in two with one piece being included later in the plate (last wells). Initially there were six redundants on each plate, but these were increased to ten; one for each row of eight samples with the redundants being grouped in the last two rows. This represents 6.8% (6 redundants out of 88 samples) and 11.4% (10 redundants out of 84 samples) of the samples being re-genotyped. The redundants served two functions, the first was to ensure that there were no inversions (reversal of a row or the whole plate) while processing the plate of samples; the second was as a genotype control. If a sample and its corresponding redundant did not agree, the reason was investigated before the scoring was considered to be complete for that plate. When the reason for the discrepancy was determined (row inversion, artifacts from running the gel (smiling, frowning), genotyping error, transcription error etc.) the genotyping of the locus across the whole gel was reviewed (or the gel was rerun) and corrected as required. Based on this methodology, while we did repeat samples (between 6.8% and 11.4%; see above) they were not scored independently and therefore cannot be used to estimate the genotyping error rate. For all loci, except *Mvi*9, all gels were scored independently by two readers. This was included as an additional level of data control given the lack of internal (to the lane) size standard and manual (as opposed to software based) scoring used with the FMBIOIII scanning platform. The independent genotyping results were compared and discrepancies were reconciled by consensus during re-examination of the original gel. Due to the abundance of one bp allele differences, consensus reconciliation was not used for the *Mvi*9 locus and for consistency the same person scored all *Mvi*9 gels. To help maintain consistency, as this project was undertaken over a number of years, one of the two scorers was the same person throughout the entire study.

Additional validation of the allele sizing for these loci was also undertaken. A subset of samples (n=344) were amplified and size fractionated on an AB3130xl capillary electrophoresis system (Applied Biosystems, Burlington, Canada) as this platform has higher resolution and includes an internal size standard (Gene Scan 500 LIZ (Applied Biosystems, Burlington, Canada)) with each sample. For this analysis, DNA samples were normalized to 20 ng µL^-1^. The PCR reactions were done in 10 µL containing 20 ng of template DNA, 1X Type-it multiplex PCR master mix (Qiagen, Mississauga, Canada) and 1X primer mix (see below). The six microsatellite loci were combined in two panels. The PCR cycles consisted of one cycle of 95° C for 5 minutes, thirty cycles of 95° C for 30 seconds, the annealing temperature for 3 minutes and 72° C for 30 seconds followed by one cycle of 60° C for 30 minutes. The annealing temperature for the first panel was 55° C and for the second panel was 62° C. The primer mix for the first panel was comprised of *Mvi*3 (labeled in PET), *Mvi*5 (labeled in VIC), *Mvi*10 (labeled in NED) and *Mvi*2 (labeled in 6FAM). The primer mix for the second panel included *Mvi*9 (labeled in 6FAM) and *Mvi*16 (labeled in VIC). For *Mvi*9 and *Mvi*16, the forward primers (the unlabeled primers) were synthesized with the dinucleotide + tail option (Applied Biosystems, Burlington, Canada) to encourage non-template nucleotide addition (generally known as adenylation) by *Taq* polymerase to go to completion [2]. This approach was tested with *Mvi*2 as well, but found to have a negative impact on the interpretability of the electrophoresis results for this locus.

The same type of allele size differences (S2 Table) were seen with these amplification and size fractionation procedures for all six loci. This included single base alleles for *Mvi*2, *Mvi*9 and *Mvi*16. The use of the tailed primers for *Mvi*9 and *Mvi*16 suggests that these single base alleles are the result of sequence changes in the PCR fragment itself and not due to incomplete adenylation of PCR products. These results support the fragment size interpretation (scoring) used in this study.

**Supplementary References**

1. Gordos K, Kenchington EL, Hamilton LC, Nakashima BS, Taggart CT. Atlantic capelin (*Mallotus villosus*) tetranucleotide microsatellites. Mol Ecol Notes. 2005; 5: 220-222.

2. Brownstein MJ, Carpten JD, Smith JR. Modulation of Non-Templated Nucleotide Addition by Taq DNA Polymerase: Primer Modifications that Facilitate Genotyping. BioTechniques. 1996; 20: 1004-1010.
