# Supplementary material for: Genetic Structure of Capelin (Mallotus villosus) in the Northwest Atlantic Ocean
Source: PLoS One. 2015 Mar 30;10(3):e0122315. doi: 10.1371/journal.pone.0122315 (PMC4378951; doi:10.1371/journal.pone.0122315)
Supplement: S2 Text — (DOCX) [file pone.0122315.s010.docx]

**S2 Text. Preliminary Genetic Analyses: Methods and Results.**

**Methods**

Linkage disequilibrium (LD) and deviation from Hardy-Weinberg Equilibrium (HWE) in the 45 collections were tested with Genepop version 4.0.10 [1]. The log likelihood ratio statistic (*G*-test) was used to test the LD null hypothesis that genotypes at one locus are independent from genotypes at the other locus. HWE exact tests were performed to test the null hypothesis of random union of gametes. A Markov chain (MC) algorithm run with 1,000 iterations was used to estimate, without bias, the exact *P*-value [2].

Weir and Cockerham’s *F*-statistic (*θ* ) [3] was calculated using Genetix version 4.05.2 [4]. This statistic weighted the allele frequencies according to sample size and was appropriate for our data that reflected widely varying sample sizes. A jack-knife procedure on the loci and samples was used to provide variance estimates, and confidence intervals (C.I.) were generated by bootstrapping over loci. Statistical significance was determined using Fisher’s Exact test as implemented in Genepop with MC simulation and 1,000 iterations. Population differentiation (*θ* ) amongst the 75 temporal, within-location pair-wise comparisons was assessed. Temporal samples with non-significant differentiation were pooled for subsequent analyses following Bekkevold et al. [5]. Four locations (BB65, DRL, SLL and SR) were sampled in a single year; therefore we were unable to directly evaluate the effect of temporal variability among all sample locations and could not rule out the possibility that the single-year samples were atypical for their location. We assumed that the general pattern of no significant genetic differentiation among collection years applied to those locations as well.

**Results**

There was no evidence of linkage among the six loci in the 45 samples collected. Of the 675 tests performed (15 pairs of loci x 45 samples) 78 tables had no information, that is all row or all column marginal sums were one; five were significant at *P* ≤ 0.05 (2003SV: *Mvi*3 and *Mvi*5, *P* = 0.00 ± 0.000; *Mvi*2 and *Mvi*5, P = 0.00 ± 0.001; 2006BB: *Mvi*2 and *Mvi*3, *P* = 0.01 ± 0.007; 2002TW: *Mvi*3 and *Mvi*10, P = 0.03 ± 0.017; 2002CC: *Mvi*5 and *Mvi*10, *P* = 0.03 ± 0.017) and none were significant after correcting for multiple tests. None of these five significant tests occurred within the same locus pair.

Of the 270 multiple tests of HWE, the null hypothesis of random union of gametes was rejected in 50 with *P* ≤ 0.05 (S1 Table). All but one (2007SES) of those showed heterozygote deficiency. After correcting for multiple tests, eleven samples were out of HWE (*P* ≤ 0.0002). These were from ten different samples and involved five of the six loci (*Mvi*3 showed no deviations). One locus, *Mvi*16 was out of HWE in six of those eleven significant deviations. Five of those eleven significant tests were also associated with a large *F*_IS_ ≥ 0.1 with only two from the same sample (GSL collected in 2008 with *Mvi*2 and *Mvi*16). Of the 75 within-location pair-wise combinations of temporal samples, 46 showed significant differences at *P* ≤ 0.05, although only six exhibited genetic differentiation outside the upper 95% confidence interval (C.I.) of the mean of *θ* (estimated at 0.0016; 95% C.I. 0.0002 – 0.0034). These samples were considered to be the most temporally distinct samples. Theta took values of 0.0036 to 0.0049 between the 2005 sample from Chance Cove (CC) and those collected in 2002, 2003 and 2004. Temporal variation was also identified between the 2004 and 2006 samples from Little Lawn (LL) (*θ* = 0.0039) and between the 2005 sample from Straitsview (SV) and those collected in 2003 and 2004 (*θ* = 0.0043 and 0.0044 respectively). Consequently, samples 2005CC, 2004LL, and 2005SV were not pooled with other temporal samples from CC, LL and SV locations, respectively, and most subsequent analyses were performed on 18 samples (15 locations + 2005CC, 2004LL, 2005SV) (S2 Table).

**Supplementary References**

1. Rousset F. Genepop’007: a complete re-implementation of the Genepop software for Windows and Linux. Mol Ecol Res. 2008; 8: 103–106.

2. Guo SW, Thompson EA. Performing the exact test of Hardy-Weinberg proportion for multiple alleles. Biometrics. 1992; 48: 361-372.

3. Weir BS, Cockerham CC. Estimating F-statistics for the analysis of population structure. Evolution. 1984; 38: 1358-1370.

4. Belkhir K, Borsa P, Chikhi L, Raufaste N, Bonhomme F. GENETIX 4.05, logiciel sous Windows TM pour la génétique des populations. Laboratoire Génome, Populations, Interactions, CNRS UMR 5000, Université de Montpellier II, Montpellier (France); 2004.

5. Bekkevold D, André C, Dahlgren TG, Clausen LAW, Torstensen E, Mosegaard H, et al. Environmental correlates of population differentiation in Atlantic herring. Evolution. 2005; 59: 2656–2668.
