# Supplementary material for: Genetic Structure of Capelin (Mallotus villosus) in the Northwest Atlantic Ocean
Source: PLoS One. 2015 Mar 30;10(3):e0122315. doi: 10.1371/journal.pone.0122315 (PMC4378951; doi:10.1371/journal.pone.0122315)
Supplement: S3 Text — (DOCX) [file pone.0122315.s011.docx]

**S3 Text. Individual Assignment Tests: Methods and Results.**

**Methods**

The Præbel et al. study of genetic variation among NEA capelin [1] used assignment tests performed with Geneclass version 2.0 [2] to evaluate the fidelity of individuals to their group of origin. We undertook the same Bayesian individual assignment analyses [2] following Rannala and Mountain [3] to estimate the highest probability of a given individual being drawn from the potential source populations, applying the leave-one-out method. Our data had relatively small genetic distances in *θ* among samples and thus our expectation was one of low self-assignment. To the extent possible, we examined the temporal stability of the self-assignments by comparing results performed separately on samples collected in 2006 (BB, BB61, CC, DRL, GSL, LL, SES, SLL, SS, SV, TW) and in 2007 (BB, CC, GSL, LL, RBB, SES, SR, SS, TW,UB), while acknowledging that these differ in spatial coverage and sample composition. We also used the capelin sampled in 2006 from each of BB, CC, GSL, LL, SES, SS and TW to assign fish sampled at those same locations in 2007 using the same parameters to further examine temporal stability.

**Results**

Assignment tests correctly identified 445 of the 3,433 individuals to their sample location (13%), which was greater than that expected by chance alone (i.e., 5.6%). Separate analyses of the subset of samples collected in 2006 and 2007 showed similar levels of self-identification (11.90 and 12.61% respectively), although lower than that recorded for the composite data. The percentage of correctly identified individuals assessed at locations BB, CC, GSL, LL, SES, SS and TW using fish sampled in 2006 as the reference to assign fish sampled in 2007 was 12.8%.

**Supplementary References**

1. Præbel K, Westgaard JI, Fevolden SE Christiansen JS. Circumpolar genetic population structure of capelin *Mallotus villosus*. Mar Ecol Prog Ser. 2008; 360: 189-199.

2. Piry S, Alapetite A, Cornuet JM, Paetkau D, Baudouin L, Estoup A. GENECLASS2: a software for genetic assignment and first-generation migrant detection. J Heredity. 2004; 95: 536–539.

3. Rannala B, Mountain JL. Detecting immigration by using multilocus genotypes. Proc Natl Acad Sci USA. 1997; 94: 9197-9201.
