# Supplementary material for: Genetic Structure of Capelin (Mallotus villosus) in the Northwest Atlantic Ocean
Source: PLoS One. 2015 Mar 30;10(3):e0122315. doi: 10.1371/journal.pone.0122315 (PMC4378951; doi:10.1371/journal.pone.0122315)
Supplement: S4 Text — (DOCX) [file pone.0122315.s012.docx]

**S4 Text. Bayesian clustering of individuals with Structure: Methods and Results.**

**Methods**

We investigated the genetic population structure of capelin using the Bayesian clustering program in Structure (version 2.3.4) [1]. The 15 sampling locations, excluding the 3 temporally distinct samples, were used as prior information (Locprior option), as recommended for large data sets with weak differentiation [2]. This model ignores the sampling information when the ancestry of individuals is uncorrelated with sampling locations [1]. Clustering was performed under the F-model [3] which assumes admixture of *K* putative parental populations and correlated allele frequencies. Analyses were conducted for a range of *K* values from 1 to 15 using a Markov chain Monte Carlo (MCMC) data collection chain of 500,000 steps to achieve accurate parameter estimates, with a burn-in period of 1,000,000 to reduce the effects of the starting configuration. To evaluate convergence of the MCMC we performed 10 independent simulations of *K* (1–6) and reviewed the consistency of the results. We then ran 4 to 6 simulations for *K* (7–15). The maximal value of the likelihood of the data, *Ln P(D)*, for a given *K* was used to select the optimal *K* [53]. ANOVA tested the assumption of no significant difference in *Ln P(D)* amongst the simulations of *K*. Tukey’s HSD was used as a *post hoc* test to detect homogeneous subsets of *K* containing the optimal *K*. Genetic structure was further evaluated through examination of the degree of admixture (*α*) inferred from the data [1]. Selection of the most likely number of clusters is somewhat *ad hoc* although the smallest value of *K* which explains the structure in the data is considered to be the most parsimonious solution [1]. Admixture coefficients were extracted for each individual and the best alignment across runs within selected *K* was produced with Clumpp [4] and then visualized using Distruct [5] both implemented with Clumpak (<http://clumpak.tau.ac.il> ). These plots were used to visually assess patterns of individual and sample probabilities of cluster membership. To further investigate population subdivision, hierarchical analyses were also completed with reduced data sets. The major clusters identified with Structure under the most likely *K* were reanalyzed using the same settings and simulations of *K* = 2 and 3.

**Results**

Bayesian clustering inferred from the Structure program recovered mean values of *Ln P(D)* through different *K* values from 1–15 (S6 Table) under running conditions indicative of good mixing of the MCMC. Use of sampling location in the Locprior model was uninformative for most *K* , and very poor for *K* = 1, with the exception of *K* = 2 where the *r* statistic was closest to 1 (S1 Fig.).

Overall, signals of structuring were evident in the admixture coefficient, *α*, (S1 Fig.) and in the individual assignment patterns (S2 Fig.). Values of *α* were < 1 and decreased with increasing *K*, plateauing at about *K* = 6. ANOVA rejected the null hypothesis of equal values of *Ln P(D)* across *K* (*P* = 0.000) and a homogenous subset of mean *Ln P(D)* was identified for *K* = 1 to 7 informed by Tukey’s HSD test (*P* = 0.077). The maximal value of the likelihood of the data other than *K* = 1 was achieved at *K* = 2 which based on all parameters was considered to be the most parsimonious number of basal clusters (S6 Table). For each of the two ancestral clusters under that model, hierarchical analyses did not detect further substructure (Cluster I: (*K* = 2: *r* = 10.4734, mean *α* = 1.9755; *K* = 3: *r* = 15.2701, mean *α* = 0.6782); Cluster II: (*K* = 2: *r* = 16.4176, mean *α* = 2.9640; *K* = 3: *r* = 19.3762, mean *α* = 0.8963) and simulations were stopped at *K* = 3.

Assignment of individuals to clusters formed under *K* = 2, 3, 4, 5, 6, 7 (based on mean *Ln P(D)*), *K* = 10 (smallest *α*) and *K* = 12 (low *r*) of the simulation with the highest *Ln P(D)* within each *K*, were illustrated with bar plots ordered by sample location (S2 Fig.). All simulations showed complex admixture patterns. Clusters did not segregate according to sample location but at higher *K* each sample location showed a similarity in the proportion of group membership displayed by their individuals, with distinct patterning evident in the plots (S2 Fig.).

At *K* = 2 the assignments reflected the major relationships identified in the cluster analysis (Figs. 2B, and S2). Individuals from the demersal spawning sites at Grebes Nest (BB65) and Domino Run, Labrador (DRL), the Saguenay River (SR) capelin in the St. Lawrence River estuary, those from the St. Lewis (SLL), Aguanus River (GSL) and Scotian Shelf (SS), and capelin from the Southeast Shoal (SES) all were strongly associated with one of the genetic groups (Cluster I). Within this group, mean membership probability by sample location was greater than 90% in all cases (individual range 71-100%). The second group (Cluster II) was not well resolved with all individuals and sampling locations admixed with Cluster I. On average sample locations in this second group displayed 60% proportional membership to Cluster II and 40% proportional membership to Cluster I. Samples with their highest proportional membership in each of Cluster I and Cluster II exactly correspond to the sample locations separated in the MDS and in the complete linkage dendrogram produced from Jost’s *D*_est_ (Fig. 2B of publication).

# Examination of the bar plots of proportional cluster membership by individuals grouped by sample location, under increasing values of *K*, indicated that the samples with high proportional membership with Cluster I of the *K* = 2 model showed increased distinction from one another with increasing *K*, although these distinctions were not through unique ancestral populations but rather through strongly similar patterns of mixed ancestry (S2 Fig.). This suggests that if hierarchical clusters exist within the data, they are more likely to be found amongst samples associated with Cluster I under the *K* = 2 model.

**Supplementary References**

1. Pritchard JK, Stephens M, Donnelly P. Inference of population structure using multilocus genotype data. Genetics. 2000; 155: 945-959.

2. Husbisz M, Falush D, Stephens M, Pritchard J. Inferring weak population structure with the assistance of sample group information. Mol Ecol Res. 2009; 9: 1322-1332.

3. Falush D, Stephens M, Pritchard JK. Inference of population structure using multilocus genotype data: linked loci and correlated allele frequencies. Genetics. 2003; 164: 1567-1587.

4. Jakobsson M, Rosenberg NA. CLUMPP: a cluster matching and permutation program for dealing with label switching and multimodality in analysis of population structure. Bioinformatics. 2007; 23: 1801–1806.

5. Rosenberg NA. DISTRUCT: a program for the graphical display of population structure. Mol. Ecol. Notes. 2004; 4: 137-138.
